# Supplementary material for: Fifteen Shades of Grey: Combined Analysis of Genome-Wide SNP Data in Steppe and Mediterranean Grey Cattle Sheds New Light on the Molecular Basis of Coat Color
Source: Genes (Basel). 2020 Aug 13;11(8):932. doi: 10.3390/genes11080932 (PMC7464420; doi:10.3390/genes11080932)
Supplement: Supplementary file 1 [file genes-11-00932-s001.zip › Supplementary file S1.docx]

**Supplementary file S1.** Functions of genes in the ± 250 kbp region upstream and downstream the locus Hapmap49624-BTA-47893 on BTA2, detected as significant in 100% of the pair-wise contrasts performed using Limousin as reference breed.

***PMS1*** (PMS1 Homolog 1, Mismatch Repair System Component) encodes a protein belonging to the mutL/hexB family. It is thought to be involved in the repair of DNA mismatches, being considered to be a component of the **DNA mismatch repair** (MMR) complex composed at least of MSH2, MSH3, MSH6, PMS1 and MLH1. An increasing body of evidence indicates that MMR is involved in **UV-induced tumorigenesis of melanoma** and nonmelanoma skin cancer [1,2]. A *PMS1* truncation mutation has been recently described in a patient with **uveal melanoma**, **ocular surface melanosis**, and nevus of Ota [3], a blue **hyperpigmentation** that occurs on the face, most often appearing on the white of the eye. Loss of PMS1 has been identified also in **dermal melanomas** [4-6]. Microsatellite instability and/or mutations in other mismatch repair genes have been also found in uveal melanomas [7-9], in dermal melanoma [10-18] and in benign as well as melanocytic dysplastic nevi [19-21, 14]. A general upregulation of genes involved in the MMR system was observed in **dysplastic nevi** and in the transition from radial growth phase melanomas to vertical growth phase melanomas, confirming that consistent DNA damage occurs during **melanoma** progression [22-24]. Mutations at MMR genes were also observed in hamartoma polyp tissue of patients with the **Peutz-Jeghers syndrome**, a rare disease with clinical manifestations of **pigmented spots on the lips, mucous membranes and extremities**, scattered gastrointestinal polyps, and susceptibility to tumors [25]. In a patient with the Peutz-Jeghers syndrome, Vageli et al. [26] reported a reduction of mRNA expression of *MLH1* in the biopsy from the **oral pigmented area**, and of all the four considered MMR genes in hamartomatous polyps, compared to levels found in peripheral blood. Mutations at MMR genes have been also reported in a syndrome termed constitutional mismatch repair deficiency (CMMR-D), characterized by association between the presence of multiple **cafe´-au-lait spots** and skin-fold freckling, adenomatous colonic polyps with early-onset colorectal carcinoma, and a predisposition to a variety of pediatric malignancies [27]. Also, somatic inactivation of the *NF1* gene through mismatch repair defects has been proposed to explain the occurrence of cafe´-au-lait spots and other features of neurofibromatosis type 1 in affected patients, consistently with the evidence that the *NF1* gene is a mutational target in mismatch repair–deficient cells [28]. MMR genes have been also implicated in **vitiligo**. Indeed, in humans, the 3′-end of the *VIT* cDNA sequence has been shown to be complementary to the 3′-end of hMSH6, a G/T mismatch repair [29]. Preliminary results indicated that decreased levels of VIT1 are associated with increased levels of hMSH6. Complementary stretches of mRNA can hybridize within the cell, and as double-stranded RNA can mediate post-transcriptional gene silencing, the VIT1 transcript potentially modifies the level of G/T mismatch repair protein in the cell [30]. In fact, high levels of hMSH6 may reflect increased DNA damage in vitiligo melanocytes [31].

**ORMDL1** (ORMDL Sphingolipid Biosynthesis Regulator 1) encodes a transmembrane protein anchored in the endoplasmic reticulum that act as a **negative regulator of sphingolipid synthesis** [32]. The sphingolipid metabolic pathway is a fundamental feature of all eukaryotic cells, required to produce complex sphingolipids, such as sphingomyelin and the glycosphingolipid family, that are plasma-membrane building blocks. It also generates bioactive metabolites (such as ceramide, sphingosine, and sphingosine-1-phosphate) that alter cell activities, including growth regulation and apoptosis, through interactions with receptors and enzymes [33]. *ORMDL1* belongs to the evolutionarily conserved ORMDL family [34]. Notably, ORMDL proteins have been shown to directly regulate activity of serine palmitoyl transferase (SPT), the rate-limiting step in **ceramide** biosynthesis [35,36]. Several lines of evidence demonstrated that free-cholesterol induced an autophagic-mediated increase in the turnover of ORMDL1. ORMDL proteins have been recognized as responders to cholesterol excess, exiting the ER to activate SPT and increase sphingomyelin biosynthesis, possibly buffering cellular cholesterol excess [37]. *ORMDL1* was isolated by Araki et al. [38] who showed that it is specifically down‐regulated in association with *PS1* (presenilin 1) mutations. They also showed that *ORMDL1* suppression affects **presenilin-γ‐secretase activity**, with γ‐secretase being responsible for the production of amyloidogenic β‐amyloid protein, whose accumulation in senile plaques appears to play a key role in the pathogenesis of Alzheimer's disease, thus highlighting *ORMDL1* relevance in presenilin‐γ‐secretase function and possibly Alzheimer [38]. Experiments using yeast knockout strains also suggest that ORMDL functions in correct **protein folding** and/or **trafficking in the endoplasmic reticulum** [34]. Miller et al. [39] refers that ORMDL3, a member of the ORMDL family, has been shown to induce expression of genes with potential importance to the pathogenesis of asthma including **metalloproteases** (MMP-9 and ADAM-8), CC chemokines (CCL-20 also known as MIP-3α), and CXC chemokines (IL-8 and CXCL-10) in normal human lung bronchial epithelial cells, and that it activates the ATF6 pathway, one of three branches of the ER localized **unfolded protein response** (UPR). Other studies have suggested that ORMDL3 may impact the PERK/eIF2α pathway, interacts with SERCA-2 and inhibits its function. ORMDL3 mRNA abundance was shown to depend on STAT6. The latter would not be directly regulating ORMDL3 expression, rather through a STAT6-dependent mediator.

Interestingly, zebrafish larvae presenting genome-edited mutations at the **presenilin** gene (i) initially show grossly normal melanotic **skin pigmentation** but subsequently loose this as they grow while retaining pigmentation in the retinal pigmented epithelium, or (ii) retain faint skin melanotic pigmentation once adults [40]. Mammalian presenilins have also been found necessary for **tyrosinase trafficking and melanin formation** by a γ-secretase-dependent mechanism [41,42]. The two tyrosinase-related proteins, tyrosinase-related protein 1 (Tyrp1) and dopachrome tautomerase (DCT) (also known as tyrosinase-related protein 2 (Tyrp2)), are implicated in the activity of the intramembrane protease, γ-secretase. A partial loss-of-function in melanotic pigment formation has been observed in a mouse model of the PSEN1 fAD mutation M146V. In mammals, the protein *silver* (coded by the *SILV* gene, also known as premelanosomal protein, ***PMEL***) can also be cleaved by proteases including γ-secretase to form a natural functional amyloid that facilitates melanin formation. *Silver* is expressed in pigment cells of the eye and skin, which synthesize melanin pigments within melanosomes. After a juxta-membrane cleavage, the C-terminal fragment of SILV is then processed by the γ-secretase complex to release an intracellular domain fragment into endosomal precursors to form amyloid fibrils. These ultimately become melanosomes [40].

As what concerns a possible role of presenilin-activated **unfolded protein response** (UPR), we recall here that tyrosinase, the first and rate-limiting enzyme in melanin biosynthesis, undergoes an extended period of post-translational modification, including N-linked glycosylation and disulfide bond formation-dependent folding in the endoplasmic reticulum (ER) in order to attain a functional tertiary structure. This process requires classical ER chaperones as well as melanocyte-specific factors. Mutations at four loci, encoding melanocyte-specific genes have been shown to result in **tyrosinase misfolding**, namely tyrosinase itself (TYR), the oculocutaneous albinism type 2 gene (OCA2), tyrosinase-related protein 1 (TYRP1) and OCA4. Misfolding results in **peptide retention** in the ER, the primary cellular site for protein synthesis and folding. A complex quality control system ensures that only correctly folded proteins are transported to the Golgi for further modification or to their site of activity. Misfolded proteins are either refolded or targeted for **proteasomal degradation**. Accumulation of unfolded proteins in the ER exerts a stress on the organelle which if unchecked activates the signal cascade known as the unfolded protein response (UPR). In the event that the stress is not resolved, the UPR can induce **apoptosis**. To this regard, **a role for UPR in vitiligo**, a disease that results from localized death of skin melanocytes, has been suggested [43].

Also, in zebrafish, presenilins were shown to influence **Notch signaling** resulting in perturbed neurogenesis and trunk and tail **neural crest development** [44].

Mutations in *PSENEN*, which encodes presenilin enhancer protein 2 (PEN-2), a subunit of the **γ-secretase complex**, was shown to underline a type of Dowling-Degos disease, which is characterized by progressive reticulate **hyperpigmentation** and small, dark-brown hyperkeratotic papules on the trunk, face, flexures, large skin folds, and extremities [45].

***OSGEPL1*** (O-Sialoglycoprotein Endopeptidase Like 1) encodes a protein that is a subunit of the multiprotein KEOPS complex, whose molecular mechanisms of actions remain elusive. *OSGEPL1* is involved in mitochondrial genome maintenance [46] and, hence, proper mitochondrial function. For a discussion on the **close connection between melanosomes and mitochondria function**, please, see Supplementary file S4. Together with YRDC, another subunit of the KEOPS complex, OSGEPL1 is responsible for a key step in the maturation of mitochondrial tRNAs, a N 6-Threonylcarbamoyladenosine (t^6^A) modification (t6A37). *OSGEPL1* knockout cells exhibited respiratory defects and reduced mitochondrial translation, suggesting that t6A37 plays a critical role in this process [47]. *In vivo* impairment of KEOPS proteins has been associated with various pathological conditions, generally characterized by **neurological and neurodegenerative symptoms** [48]. Another diseases associated with *OSGEPL1* is **hemochromatosis** (Type 4). This condition is characterized, among other classical features, by **hyperpigmentation of the skin**, usually most evident on sun-exposed skin, particularly on the face. Hyperpigmentation of external genitalia, flexion folds, scars, nipple areola, buccal mucosa and conjunctiva can also be observed in some patients. The color of skin is generally **slate grey or brownish bronze**. It in not yet clear whether the hyperpigmentation is due to (i) hemosiderin deposition resulting in diffuse, slate-gray darkening or (ii) increased production of melanin in the epidermis [49], or both. Some studies suggested that **hemosiderin**, as well as iron, other bivalent cations, and heavy metals **may stimulate melanogenesis** [50-52] and that melanosomes of dysplastic naevi and melanoma cells contained higher amounts of iron [53]. The *OSGEPL1* paralog, *OSGEP*, has been identified as the glycoprotease responsible for regulating the response of primary acute promyelocytic leukemia (APL) cells to UPR-induced apoptosis through **processing of misfolded** N-CoR (nuclear hormone receptor corepressor) **proteins**. Down-regulation of *OSGEP* was shown to favor **apoptosis** in APL cells [54]. For a discussion of the connection between unfolded protein response (UPR) and pigmentation, please, see the above gene *ORMDL1*. Specific interactions with *OSGEP* and *LAGE3*, which are human orthologues of the KEOPS complex were identified for the PRAME (**preferentially expressed antigen in melanoma**) oncoprotein, a BC-box subunit of a Cullin2-based **E3 ubiquitin ligase** [55], first identified and cloned as the antigen responsible for an anti-tumor immune response in a melanoma patient [56] and reported to repress **retinoic acid signaling** **in melanoma cell lines** [57]. PRAME specifically interacted with the KEOPS complex and recruited Cullin2-based E3 ubiquitin ligases to KEOPS, although it was not possible in this study to define whether KEOPS could be the ubiquitination targets of Cullin2-PRAME [55]. In yeast, inactivation of a KEOPS subunit specifically inhibited telomere recombination, a phenomenon known to elicit genome instability and to accelerate **cellular aging**, thus significantly extending **cell longevity** in both telomerase-positive and pre-senescing telomerase-negative cells [58]. In *Saccharomyces cerevisiae*, the KEOPS complex was shown to play a role in **cell polarity** by contributing to proper localization of a bud-site landmark protein [59]. The KEOPS complex was also identified as a transcription factor [60]. Components of KEOPS are nuclear proteins that associate with chromatin *in vivo* and are important for **inducible gene transcription** [60]. A KEOPS subunit has been shown to functionally interact with **glutaredoxin** [61], which is known to act as a cytosolic iron-sulfur (Fe-S) cluster assembly factor that facilitates (2Fe-2S) cluster insertion into a subset of cytosolic proteins [62,63]. In addition, in *Pyroccocus abyssi*, OSGEP was shown to contain an **iron** (Fe^3+^) atom, that was reduced into Fe^2+^ by treatment with ascorbic acid [64]. Another KEOPS subunit was shown to possess kinase activity toward **p53** and to be activated by the **Akt/PKB** signaling pathway [65]. As p53 is a major protein in the control of genome integrity, a role for the KEOPS complex in **checking DNA damage** has also been proposed [64]. Literature evidences of the roles of **p53 in melanocyte biology and pathology** have been provided [66-70]. Inactivation of **Akt/PKB** has been associated with **reduced cell proliferation** in a melanocyte cell line, as well as **reduced cell pigmentation** by inhibition of the tyrosinase activity [71]. Akt/PKB has been widely shown to be involved in the regulation of melanogenesis [72-76].

***ANKAR*** (Ankyrin and Armadillo Repeat Containing) encodes a protein containing ankyrin and armadillo repeats. Tandem-repeat domains are one of the most abundant classes of protein–protein interaction domains. Some of the most common repeat domains include the ankyrin (ANK) and armadillo (ARM) domains. Tandem-repeat containing proteins are present in all domains of life and function in nearly every cellular process, including cell–cell signaling, cytoskeleton integrity, transcription and cell–cycle regulation, inflammatory response, development, and various transport phenomena [77,78]. The multiple specificities of ankyrin repeats are potentially physiologically significant, through which distinct pathways can be cross-linked or coordinated [79]. Also, ankyrin repeats may play a role as chaperones in the phenomenon of protein folding induced by binding. The *ANKAR* paralog, *ARMC4*, encodes a protein that is thought to be involved in **ciliary organization and function**. This protein has been shown to localize to the ciliary axonemes and at the ciliary base of respiratory cells. Studies indicate that mutations in this gene cause partial **outer dynein arm defects** in respiratory cilia. The cilia of cells with mutations in this gene displayed either reduced ciliary beat frequency and amplitude, or, complete immotility. Some individuals with **primary ciliary dyskensia** (PCD) have been shown to have mutations in this gene. A possible role of primary cilia in pigment disorders has been highlighted in the **Bardet-Biedl Syndrome**, a pleiotropic genetically heterogeneous disorder, characterized, among other features, by **pigmentary retinopathy**. It is caused by impairment of the BBSome complex function. Indeed, the hetero-octameric BBSome complex plays a central role in primary cilia homeostasis. The complex acts as a cargo adapter that recognizes signaling proteins and links them to the intra-flagellar transport machinery. In zebrafish, knockdown of the BBS genes resulted in **delayed retrograde melanosome transport** (i.e. melanosome retraction to the perinuclear region) [80]. The BBSome gene *BBS9* was highlighted as under selection in the semi-feral Sasi-Ardi sheep breed from Western Pyrenees [81]. While the authors emphasize the involvement of the gene in energy metabolism and, as such, they argument it may have been under environmental selection pressure in relation with climate changes, the diluted red phenotype (“cream wool and a fawn face”; “uniform blonde or reddish color”, observed in this breed may, in our opinion be worthwhile of consideration for possible **pigmentation dilution** effects. In addition, primary cilia have been shown to negatively regulate melanogenesis in melanocytes and pigmentation in a human skin model [82]. Moreover, primary cilia have been demonstrated to have a crucial role in **neural crest cell development** [83] and, in zebrafish, cilia dysfunction has been associated with **mis-localization of melanocytes** in the head as well as absence of melanophores in the midline of the trunk [84]. Also, in the **Joubert Syndrome** ciliopathy [85], **retinal pigmentary alterations** are observed features. Impaired **melanosome recruitment** as well as **ciliogenesis defects** were observed in zebrafish mutants for the MCRS1 gene [86]. Ciliobrevins, the first specific small-molecule **antagonists of cytoplasmic dynein** were shown to **perturb protein trafficking within the primary cilium**, leading to their malformation as well as **preventing melanosome aggregation** [87]. Also, please, see Supplementary file 1, notably in what concerns the *PRICKLE2*, *GBF1* and *SUFU* genes.

***ASNSD1*** (Asparagine Synthetase Domain Containing 1) encodes a protein whose Gene Ontology annotations include **asparagine synthase** (glutamine-hydrolyzing) **activity**. Its paralog is indeed *ASNS*, which encodes the enzyme asparagine synthetase, responsible for conversion of aspartate and glutamine to asparagine and glutamate in an ATP-dependent reaction [88]. Studies on *ASNSD1* are very few. An inactivating mutation in the *ASNSD1* gene was shown to be associated with **progressive degenerative myopathy** in mice [89]. Several studies have, instead, been performed on *ASNS* which will be briefly and not exhaustively presented in what follows. *ASNS* has been found to be **up-regulated in various cancer types**, including gastric cancer tissues [90], lung cancer tissues [91], glioblastomas [92], and often associated with poor prognosis [93,94]. Li et al. [94] provided evidence for *ASNS* **involvement in the regulation of melanoma cell growth and cell cycle control**. Indeed, in melanoma cells, inhibition of *ASNS* expression significantly suppressed cell growth and induced a G0/G1 cell cycle arrest. Knockdown of *ASNS* remarkably downregulated the expression levels of cyclins and cyclin-dependent kinases, two kinds of crucial cell cycle regulatory molecules, and upregulated the expression of p21, a potent inhibitor of cyclin-dependent kinases. A marked reduction in proliferation of patient fibroblasts when cultured in asparagine-limited growth medium [95] and arrest of cell cycle progression at G0/G1 phase in lung cancer cells [91] have been also described. The accumulated evidences highlight that maintenance of intracellular asparagine levels is critical for cancer cell growth. Indeed, asparagine regulates mTORC1 complex activity and protein synthesis and, through regulation of serine uptake, it influences serine metabolism and nucleotide synthesis, suggesting that asparagine is involved in coordinating protein and nucleotide synthesis and is an important regulator of cancer cell amino acid homeostasis, anabolic metabolism and proliferation [96]. In colorectal cancer, the expression of *ASNS* was upregulated by mutated *KRAS*, with mutations in the *KRAS* gene being found in various types of cancer, and it was induced by KRAS-activated signaling pathways, in particular the PI3K-AKT-mTOR pathway [97]. Inhibition of *ASNS* was shown to induce p53/p21-dependent **senescence** and cell cycle arrest through a mechanism involving modulation of LKB1 activity [98].

Moreover, asparagine synthethase has been shown to be induced by **unfolded protein response** (UPR) under ER stress [99,100] (please, also see the *ORMDL1* gene, presented above, for a **role of UPR in pigmentation**). Induction of asparagine synthetase by the endoplasmic reticulum stress was shown to associate with inhibition of **lysosome acidification** while knockdown of *ASNS* restored **autophagic flux** [101]. Inhibition of either autophagy or *ASNS* was shown to reduce tumor cell proliferation, migration, and invasion, linking *ASNS* overexpression with poor clinical outcome in multiple cancers [102]. Asparagine synthetase has also been shown to undergoe regulated recruitment to the **mitotic spindles** and that it may have acquired a second role in mitosis similar to other metabolic enzymes that contribute to **metabolic reprogramming in cancer cells** [103].

Asparagine residues plays a role in **N-linked glycosylation** of proteins, a process, in which carbohydrates are attached to asparagine residues (N) residing within a conserved sequence pattern. Mature human **tyrosinase** is a type 1 membrane-bound glycoprotein Genetic changes affecting the conserved patterns near asparagine residues are associated with OCA1 **albinism**. In the cell, N-glycosylation plays an essential role in the **transfer of tyrosinase from ER to the cytoplasm** and in maintaining its **folding, stability** (and hence degradation rate), and **enzymatic activity**. The glycosylation of tyrosinase might regulate the melanin production in different types of cultured melanoma cells. It has been proven in mouse melanoma cells, that inhibition of the early steps of the N-glycosylation process strongly affects tyrosinase activity, thereby changing melanin synthesis. Moreover, the abnormal N-glycosylation process is related to the **depigmented phenotype of human melanomas** [104].

It has been shown that the 5′ UTR of *ASNSD1* holds an **upstream open reading frame** (ORF) capable of producing a cytoplasmic small ORF-encoded peptide whose peptide sequence is conserved across vertebrates, that has been named *ASDURF*. *ASDURF* has been shown to encode a protein with significant sequence and computationally predicted structural homology to the **prefoldin** chaperone family. ASDURF can bind to other prefoldin subunits of the prefoldin-like module (PFDL) which is part of the **PAQosome** complex, essential for the assembly and stabilization of other macromolecular complexes involved in essential cellular functions such as protein synthesis, ribosome biogenesis, transcription, splicing, and others [105,106]. In pathophysiological **stress conditions**, such as ER stress, **UVB exposure**, and others, start codon recognition and translation initiation is generally hindered, resulting in overall translational repression that conversely favors expression of upstream ORF-regulated mRNAs by bypassing these regulatory elements. In other words, upstream ORFs are one of the examples of post-transcriptional strategies employed by the cell to reorganize its protein expression landscape in an effort to restore homeostasis [107]. Whole genome expression profiles of **melanocytes from dysplastic naevi** and adjacent normal skin subjected to Gene Ontology (GO)-based comparative statistical analysis yielded significantly differentially expressed GO classes including “prefoldin complex” and validation of genes from the top GO classes confirmed an heterogeneous **differential expression pattern** [108]. Prefoldins have been shown to play important roles in **cancer development and progression** [109], and in promoting **epithelial-mesenchymal transition** [110], stability of axonemal **dynein** heavy chains [111], biogenesis of cytoskeletal-related proteins and **cytoskeletal assembly** during the folding of actin and tubulin monomers, **disruption of neuroblast polarity** and overgrowth, **development of central nervous system** , androgen and estrogen receptors transcriptional activity [112] and male fertility [113]. The involvement of prefoldin subunits in the cytoplasmic assembly of some non-cytoskeletal complexes has also been established [114]. Prefoldins are also believed to detect proteins that have folded incorrectly and target them to other chaperones that allow for repeat attempts at **refolding** [115]. Prefoldins would play a role in quality control against protein aggregation, and dysfunction of prefoldin is one of the causes of neurodegenerative diseases [116]. A prefoldin-like protein has been shown to be an integral component of the **NF-kB** (see Supplementary file S4 for NF-kB) enhanceosome and to be essential for its nuclear function [117].

***SLC40A1*** (Solute Carrier Family 40 Member 1 alias Ferroportin-1) encodes a protein that may be involved in **iron** ion transmembrane transporter activity. Defects in this gene are a cause of hemochromatosis (see *OSGEPL1* gene above for a link between **hemochromatosis, iron and pigmentation**). *SLC40A1* has been shown to be a manganese-responsive protein that decreases manganese cytotoxicity and accumulation [118] and can also function as a **manganese exporter** [119,120] or also export other **divalent cations** [121]. Manganese is an essential divalent cation that functions as a **cofactor** for the activity of numerous enzymes in cellular processes. For example, the addition of manganese divalent cations resulted in a significant increase in basal adenylate cyclase activity stimulated by a-MSH in melanoma cells was and in enhanced inhibitory powers of adenosine, normally known to be an inhibitor of the hormone-stimulated melanoma adenylate cyclase activity [122]. As another example, the amount of SOD2, a mitochondrial manganese‐dependent superoxide dismutase was found to increase when **melanin synthesis** was inhibited in normal human melanocytes, suggesting that the manganese-dependent induction of *SOD* could be correlated with melanogenesis [123]. Laddha et al. implicated a polymorphism at *SOD2* with **vitiligo** and proposed this polymorphism may be a risk factor for the disease [124], although contrasting reports have been published on this topic [125]. In melanoma patients, activity of SOD2 showed a clear increment with melanoma progression [126] and serum manganese superoxide dismutase had been suggested as a tumour marker for malignant melanoma [127].

**Normal human** **melanocytes** were shown to bind with greater strength to extracellular matrix substrates in the presence of manganese than calcium, presumably through conformational changes in the integrin molecule induced by occupation of the metal-ion-binding site [128]. A similar result was obtained by Searles et al. [129] in normal non-metastatic and **malignant metastatic human melanocytes**. They indeed demonstrated that the **integrin-dependent adhesion** of the above cell types to ECM was modulated by divalent cations, with a stronger effect exerted by manganese, followed by magnesium and calcium. The cation-modulating mechanism of integrin adhesion involves a non-covalent association of the cation molecule with binding sites on the extracellular region of integrins. In addition, manganese was shown to constitutively activate integrins in the absence of ligand, initiating a "post-integrin" cell adhesion cascade through non-integrin mechanisms. In melanoma cells, manganese ions were observed to induce integrin- and Rho kinase-dependent **focal adhesion** and stress fibre formation, as well as reduced melanoma migration [130].

Treatment of **vitiligo** patients with a preparation of an UVB-activated pseudocatalase complex containing manganese stopped the progression of active vitiligo in 95% of cases, even in patients with long lasting disease, induced stable and complete repigmentation and allowed to observe the presence of functioning melanocytes in all patients, possibly derived from a reservoir of a small population of quiescent melanocytes or precursors that has been hypothesized to exist even after many years of disease, since the authors were able to establish melanocyte cell cultures from lesional and nonlesional epidermis from patients with vitiligo of different disease duration [131].

Erway et al. [132] highlighted that *pallid* mice had a dietary manganese requirement few hundred times higher than normal mice. Based on their results, they formulated the hypothesis of a **relationship between melanocytes and trace elements**. Mice homozygous for the *pallid* spontaneous mutation have a light, yellow-brown coat, in combination with slightly abnormal behaviour, abnormal postural responses and head tilting due to the absence of otoliths in many but not all mutant mice. The effect of *pallid* on behaviour and otolith morphology appears to be a result of manganese deficiency. In 1972, Cotzias et al. [133] highlighted that, in pallid mice, **transportation** through the tissues of **manganese**, **L-dopa** (a precursor of melanins) and L-tryptophan was **slower** than observed in black C57Bl/6J mice. Previously, they demonstrated that **melanin granules are very rich in manganese**, and differences in the concentration of manganese were observed in pigmented vs. non-pigmented tissues in adjacent areas of the scalp and skin in human, dog and cattle and in darker vs lighter unicoloured barbs of multicoloured feathers from chicken, turkey and pheasant. In cattle conjunctiva, the **pigmentation also clearly reflected the concentration of manganese** [134]. Pallid mice have prolonged bleeding time due to a platelet storage pool deficiency (SPD) characterized by a normal platelet number but a deficiency in the number of platelet dense granules and in the serotonin, ATP, and ADP content of the granules. Interestingly, two other mouse coat colour mutants, *muted* (Bloc1s5mu) and *mocha* (Ap3dmh), present a similar concatenation of pigment, otolith, and platelet SPD abnormalities, which also occur in human **Hermansky-Pudlak syndrome**, associated with mutations in several different genes implicated in **lysosome-related organelles biogenesis**. Interestingly, interactions of divalent cations other than calcium [135,136] with bilayer lipids have been shown to permit **Rab- and SNARE-dependent** **membrane fusion** [137-147]. **Rab proteins** have been shown to represent **a major component of the melanosome proteome** Chi et al [148]. Expression levels of Rab2A, as well as Rab29, were shown to be **down-regulated by α-MSH** in melanoma cell lines [149]. In addition, several studies demonstrated the role of Rab proteins in melanocyte function. Rab1A and Rab36 were shown to mediate **anterograde and retrograde melanosome transport in melanocytes**, respectively Ishida, Matsui, T. [150,151]. Knock-down of Rab4A was shown to result in defective **melanosome maturation** [152]. Rab7 was shown to regulates **maturation** **of** melanosomal matrix protein **Pmel17** [153]. **Tyrosinase and tyrosinase-related protein 1** were shown to require Rab7 for their **intracellular transport** [154] while, in another study, Rab40C was shown to be a regulator of **Tyrp1 trafficking** in melanocytes [155]. Rab9A was shown to be required for **delivery of cargo from recycling endosomes to melanosomes** [156]. Rab11b was proposed to mediate **melanin transfer between donor melanocytes and acceptor keratinocytes** via coupled exo/endocytosis [157]. Rab17 was shown to regulate **melanocytic filopodia formation and melanosome trafficking** [158]. Rab21 activation was shown to be required for **dendrite formation in melanocytes** [159]. Rab22A was shown to regulate **cargo transport to melanosomes in melanocytes** [160]. Melanocyte cell lines were also shown to employ a Rab27a-regulated exocytic system [161-162]. The Rab27a gene is also implicated in the **Griscelli syndrome**, a rare, autosomal recessive disorder, characterized by **pigmentary dilution of the skin and the hair**, the presence of large clumps of pigment in hair shafts and an accumulation of melanosomes in melanocytes [163]. Finally, activation of Rab32/38 by HPS4 (one of the genes whose mutations have been associated with **Hermansky-Pudlak syndrome**, accompanied by **premature hair greying**) has been shown to be essential for **melanogenesis** of cultured melanocytes [164].

Moreover, several metal ion binding proteins, including *SLC40A1*, together with genes involved in the **Wnt signalling pathway** and in the regulation of gene transcription, were found to be differentially expressed in ERα-positive breast tumours, mutated *vs*. wild-type at the *PIK3CA* gene encoding the catalytic subunit of the **phosphatidylinositol 3-kinase** (PI3K). Deregulation of the **PI3K signalling pathway** is frequent in human cancers. Activation of PI3K activates the serine/threonine kinase **AKT**, which in turn regulates several signalling pathways controlling cell survival, apoptosis, proliferation, motility, and adhesion [165].

Possibly worth of mention the fact that a **manganese-stimulated aminopeptidase** isolated from the bovine cerebrum was shown to inactivate L-prolyl-L-leucyl-glycinamide (PLG) [166], also known as **melanocyte-stimulating hormone release inhibiting factor** or MIF-1, a peptide originated by enzymically-controlled degradation of oxytocin in the hypothalamus [167,168], which has been shown to inhibit the release of the **melanocyte-stimulating hormone (α-MSH)** from the pituitary [169-171]. as well as from the hypothalamus [172], where α-MSH release has been shown to respond to different diurnal rhythms, suggesting independent release mechanisms in the pituitary and hypothalamus [173].

***LOC100848294*** Uncharacterized locus

***WDR75*** (WD Repeat Domain 75, alias UTP17, alias NET16) encodes a **ribosome biogenesis** factor which is part of the ribosomal **small subunit (SSU) processome** required for processing of RNA precursors of the small subunit rRNA (18S) and whose impairment is associated with **cell cycle arrest** [174]. Ribosome biogenesis has been shown to be one of the major gene networks **upregulated in melanoma after UVR exposure** [175] and to be part of the process of **melanocytes reprogramming during tumor transformation** [176,177]. Since nucleolar size is closely related to its function in ribosome biogenesis, enlarged nucleoli observed in melanoma suggests a potential **link between increased ribosome biogenesis** **and melanoma** [178]. Ribosome biogenesis is aberrantly regulated in cancer, with **MAPK** and **PI3K/AKT/mTOR pathways** being linked to these processes [181]. Since the rate of protein synthesis is proportional to the rate of cell proliferation and growth, cancer cells must rely heavily on protein synthesis. This reliance on protein synthesis can be attributed not only to disruption of translational control at the level of mRNA translation but also to accelerated ribosome biogenesis [179]. Recently, **a link between ribosome biogenesis and the tumor suppressor p53** was discovered, characterized by inhibition of p53 ubiquitination thus leading to **cell cycle arrest, apoptosis, or senescence**. Thus, disruption of ribosome biogenesis can impair rDNA transcription, ribosome generation, and mRNA translation, while activating the tumor suppressor p53 to cause cell cycle arrest and apoptosis [180,181].

WDR75 belongs to the WD repeat domain containing protein family. Another WD repeat domain containing protein, WIPI1, a regulator of **vesicle trafficking**, has been demonstrated to be involved in regulating the expression of **MITF** and of **pigmentation** [182]. WD repeats allow WIPI1 to bind to phosphatidylinositol 3-phosphate and phosphatidylinositol 3,5-bisphosphate. Studies suggest that although WIPI1 localizes to multiple different vesicular compartments under normal nutritional conditions, it localizes to the autophagosome under conditions of starvation or TORC1 inhibition [183]. In yeast, it functions to retrieve membrane from the amphisome after fusion of the autophagosome with the lysosome, and also acts as a phosphatidylinositol 3,5-bisphosphate effector to remodel the membrane of vacuoles. Yeast cells deficient in this protein fail to initiate **autophagy** correctly [183]. In COS-7 cells, WIPI1 was shown to regulate **trans-Golgi-endosomal protein trafficking** [184]. Although published studies suggest that WIPI1 controls endosome/autophagosome dynamics in other cell types [185,186], an RNAi screen determined that WIPI1 depletion also significantly **inhibited the accumulation of MITF and TYR mRNA** **in melanoma cells** [182] even though *WIPI1* has no DNA binding domain or nuclear localization signal [184]. A subsequent study revealed that WIPI1 **represses** **TORC1 signaling**, leading to the **increased transcription of MITF target genes and melanosome maturation**. Taken together, these studies define a role for *WIPI1* and MTOR signaling in melanogenesis that is distinct from their role in autophagy [183].

**REFERENCES**

1. Rass, K.; Reichrath, J., UV damage and DNA repair in malignant melanoma and nonmelanoma skin cancer. *Adv Exp Med Biol* **2008,** *624*, 162-78.

2. Seifert, M.; Scherer, S. J.; Edelmann, W.; Bohm, M.; Meineke, V.; Lobrich, M.; Tilgen, W.; Reichrath, J., The DNA-mismatch repair enzyme hMSH2 modulates UV-B-induced cell cycle arrest and apoptosis in melanoma cells. *J Invest Dermatol* **2008,** *128* (1), 203-13.

3. Toomey, C. B.; Fraser, K.; Thorson, J. A.; Goldbaum, M. H.; Lin, J. H., GNAQ and PMS1 Mutations Associated with Uveal Melanoma, Ocular Surface Melanosis, and Nevus of Ota. *Ocul Oncol Pathol* **2019,** *5* (4), 267-272.

4. Korabiowska, M.; Brinck, U.; Kotthaus, I.; Berger, H.; Droese, M., Comparative study of the expression of DNA mismatch repair genes, the adenomatous polyposis coli gene and growth arrest DNA damage genes in melanoma recurrences and metastases. *Melanoma Res* **2000,** *10* (6), 537-44.

5. Korabiowska, M.; Brinck, U.; Dengler, H.; Stachura, J.; Schauer, A.; Droese, M., Analysis of the DNA mismatch repair proteins expression in malignant melanomas. *Anticancer Res* **2000,** *20* (6B), 4499-505.

6. Korabiowska, M.; Dengler, H.; Kellner, S.; Stachura, J.; Schauer, A., Decreased expression of MLH1, MSH2, PMS1 and PMS2 in pigmented lesions indicates accumulation of failed DNA repair along with malignant transformation and tumour progression. *Oncol Rep* **1997,** *4* (3), 653-5.

7. Cross, N. A.; Murray, A. K.; Rennie, I. G.; Ganesh, A.; Sisley, K., Instability of microsatellites is an infrequent event in uveal melanoma. *Melanoma Res* **2003,** *13* (5), 435-40.

8. Hajkova, N.; Hojny, J.; Nemejcova, K.; Dundr, P.; Ulrych, J.; Jirsova, K.; Glezgova, J.; Ticha, I., Germline mutation in the TP53 gene in uveal melanoma. *Sci Rep* **2018,** *8* (1), 7618.

9. Hussein, M. R.; Haemel, A. K.; Albert, D. M.; Wood, G. S., Microsatellite instability and alterations of mismatch repair protein expression in choroidal melanomas. *Arch Ophthalmol* **2005,** *123* (12), 1705-11.

10. Quinn, A. G.; Healy, E.; Rehman, I.; Sikkink, S.; Rees, J. L., Microsatellite instability in human non-melanoma and melanoma skin cancer. *J Invest Dermatol* **1995,** *104* (3), 309-12.

11. Rass, K.; Gutwein, P.; Welter, C.; Meineke, V.; Tilgen, W.; Reichrath, J., DNA mismatch repair enzyme hMSH2 in malignant melanoma: increased immunoreactivity as compared to acquired melanocytic nevi and strong mRNA expression in melanoma cell lines. *Histochem J* **2001,** *33* (8), 459-67.

12. Richetta, A.; Ottini, L.; Falchetti, M.; Innocenzi, D.; Bottoni, U.; Faiola, R.; Mariani-Costantini, R.; Calvieri, S., Instability at sequence repeats in melanocytic tumours. *Melanoma Res* **2001,** *11* (3), 283-9.

13. Alvino, E.; Marra, G.; Pagani, E.; Falcinelli, S.; Pepponi, R.; Perrera, C.; Haider, R.; Castiglia, D.; Ferranti, G.; Bonmassar, E.; Jiricny, J.; Zambruno, G.; D'Atri, S., High-frequency microsatellite instability is associated with defective DNA mismatch repair in human melanoma. *J Invest Dermatol* **2002,** *118* (1), 79-86.

14. Shpitz, B.; Klein, E.; Malinger, P.; Osmolovsky, G.; Gochberg, S.; Bomstein, Y.; Bernheim, J., Altered expression of the DNA mismatch repair proteins hMLH1 and hMSH2 in cutaneous dysplastic nevi and malignant melanoma. *Int J Biol Markers* **2005,** *20* (1), 65-8.

15. Garcia, J. J.; Kramer, M. J.; O'Donnell, R. J.; Horvai, A. E., Mismatch repair protein expression and microsatellite instability: a comparison of clear cell sarcoma of soft parts and metastatic melanoma. *Mod Pathol* **2006,** *19* (7), 950-7.

16. Marani, C.; Alvino, E.; Caporali, S.; Vigili, M. G.; Mancini, G.; Rahimi, S., DNA mismatch repair protein expression and microsatellite instability in primary mucosal melanomas of the head and neck. *Histopathology* **2007,** *50* (6), 780-8.

17. Ponti, G.; Losi, L.; Pellacani, G.; Wannesson, L.; Cesinaro, A. M.; Venesio, T.; Petti, C.; Seidenari, S., Malignant melanoma in patients with hereditary nonpolyposis colorectal cancer. *Br J Dermatol* **2008,** *159* (1), 162-8.

18. Roncati, L., Microsatellite Instability Predicts Response to Anti-PD1 Immunotherapy in Metastatic Melanoma. *Acta Dermatovenerol Croat* **2018,** *26* (4), 341-343.

19. Birindelli, S.; Tragni, G.; Bartoli, C.; Ranzani, G. N.; Rilke, F.; Pierotti, M. A.; Pilotti, S., Detection of microsatellite alterations in the spectrum of melanocytic nevi in patients with or without individual or family history of melanoma. *Int J Cancer* **2000,** *86* (2), 255-61.

20. Hussein, M. R.; Sun, M.; Tuthill, R. J.; Roggero, E.; Monti, J. A.; Sudilovsky, E. C.; Wood, G. S.; Sudilovsky, O., Comprehensive analysis of 112 melanocytic skin lesions demonstrates microsatellite instability in melanomas and dysplastic nevi, but not in benign nevi. *J Cutan Pathol* **2001,** *28* (7), 343-50.

21. Hussein, M. R.; Roggero, E.; Sudilovsky, E. C.; Tuthill, R. J.; Wood, G. S.; Sudilovsky, O., Alterations of mismatch repair protein expression in benign melanocytic nevi, melanocytic dysplastic nevi, and cutaneous malignant melanomas. *Am J Dermatopathol* **2001,** *23* (4), 308-14.

22. Scatolini, M.; Grand, M. M.; Grosso, E.; Venesio, T.; Pisacane, A.; Balsamo, A.; Sirovich, R.; Risio, M.; Chiorino, G., Altered molecular pathways in melanocytic lesions. *Int J Cancer* **2010,** *126* (8), 1869-1881.

23. Palmieri, G.; Ascierto, P. A.; Cossu, A.; Colombino, M.; Casula, M.; Botti, G.; Lissia, A.; Tanda, F.; Castello, G., Assessment of genetic instability in melanocytic skin lesions through microsatellite analysis of benign naevi, dysplastic naevi, and primary melanomas and their metastases. *Melanoma Res* **2003,** *13* (2), 167-70.

24. Kroiss, M. M.; Vogt, T. M.; Schlegel, J.; Landthaler, M.; Stolz, W., Microsatellite instability in malignant melanomas. *Acta Derm Venereol* **2001,** *81* (4), 242-5.

25. Zhang, Z.; Duan, F. X.; Gu, G. L.; Yu, P. F., Mutation analysis of related genes in hamartoma polyp tissue of Peutz-Jeghers syndrome. *World J Gastroenterol* **2020,** *26* (16), 1926-1937.

26. Vageli, D. P.; Doukas, S. G.; Markou, A., Mismatch DNA repair mRNA expression profiles in oral melanin pigmentation lesion and hamartomatous polyp of a child with Peutz-Jeghers syndrome. *Pediatr Blood Cancer* **2013,** *60* (10), E116-7.

27. Wimmer, K.; Etzler, J., Constitutional mismatch repair-deficiency syndrome: have we so far seen only the tip of an iceberg? *Hum Genet* **2008,** *124* (2), 105-22.

28. Wang, Q.; Montmain, G.; Ruano, E.; Upadhyaya, M.; Dudley, S.; Liskay, R. M.; Thibodeau, S. N.; Puisieux, A., Neurofibromatosis type 1 gene as a mutational target in a mismatch repair-deficient cell type. *Hum Genet* **2003,** *112* (2), 117-23.

29. Acharya, S.; Wilson, T.; Gradia, S.; Kane, M. F.; Guerrette, S.; Marsischky, G. T.; Kolodner, R.; Fishel, R., hMSH2 forms specific mispair-binding complexes with hMSH3 and hMSH6. *Proc Natl Acad Sci U S A* **1996,** *93* (24), 13629-34.

30. Hammond, S. M.; Caudy, A. A.; Hannon, G. J., Post-transcriptional gene silencing by double-stranded RNA. *Nat Rev Genet* **2001,** *2* (2), 110-9.

31. Le Poole, I. C.; Sarangarajan, R.; Zhao, Y.; Stennett, L. S.; Brown, T. L.; Sheth, P.; Miki, T.; Boissy, R. E., 'VIT1', a novel gene associated with vitiligo. *Pigment Cell Res* **2001,** *14* (6), 475-84.

32. Breslow, D. K.; Collins, S. R.; Bodenmiller, B.; Aebersold, R.; Simons, K.; Shevchenko, A.; Ejsing, C. S.; Weissman, J. S., Orm family proteins mediate sphingolipid homeostasis. *Nature* **2010,** *463* (7284), 1048-53.

33. Clarke, B. A.; Majumder, S.; Zhu, H.; Lee, Y. T.; Kono, M.; Li, C.; Khanna, C.; Blain, H.; Schwartz, R.; Huso, V. L.; Byrnes, C.; Tuymetova, G.; Dunn, T. M.; Allende, M. L.; Proia, R. L., The Ormdl genes regulate the sphingolipid synthesis pathway to ensure proper myelination and neurologic function in mice. *Elife* **2019,** *8*.

34. Hjelmqvist, L.; Tuson, M.; Marfany, G.; Herrero, E.; Balcells, S.; Gonzalez-Duarte, R., ORMDL proteins are a conserved new family of endoplasmic reticulum membrane proteins. *Genome Biol* **2002,** *3* (6), RESEARCH0027.

35. Siow, D. L.; Wattenberg, B. W., Mammalian ORMDL proteins mediate the feedback response in ceramide biosynthesis. *J Biol Chem* **2012,** *287* (48), 40198-204.

36. Williams, R. D.; Wang, E.; Merrill, A. H., Jr., Enzymology of long-chain base synthesis by liver: characterization of serine palmitoyltransferase in rat liver microsomes. *Arch Biochem Biophys* **1984,** *228* (1), 282-91.

37. Wang, S.; Robinet, P.; Smith, J. D.; Gulshan, K., ORMDL orosomucoid-like proteins are degraded by free-cholesterol-loading-induced autophagy. *Proc Natl Acad Sci U S A* **2015,** *112* (12), 3728-33.

38. Araki, W.; Takahashi-Sasaki, N.; Chui, D. H.; Saito, S.; Takeda, K.; Shirotani, K.; Takahashi, K.; Murayama, K. S.; Kametani, F.; Shiraishi, H.; Komano, H.; Tabira, T., A family of membrane proteins associated with presenilin expression and gamma-secretase function. *FASEB J* **2008,** *22* (3), 819-27.

39. Miller, M.; Tam, A. B.; Cho, J. Y.; Doherty, T. A.; Pham, A.; Khorram, N.; Rosenthal, P.; Mueller, J. L.; Hoffman, H. M.; Suzukawa, M.; Niwa, M.; Broide, D. H., ORMDL3 is an inducible lung epithelial gene regulating metalloproteases, chemokines, OAS, and ATF6. *Proc Natl Acad Sci U S A* **2012,** *109* (41), 16648-53.

40. Jiang, H.; Newman, M.; Lardelli, M., The zebrafish orthologue of familial Alzheimer's disease gene PRESENILIN 2 is required for normal adult melanotic skin pigmentation. *PLoS One* **2018,** *13* (10), e0206155.

41. Wang, R.; Tang, P.; Wang, P.; Boissy, R. E.; Zheng, H., Regulation of tyrosinase trafficking and processing by presenilins: partial loss of function by familial Alzheimer's disease mutation. *Proc Natl Acad Sci U S A* **2006,** *103* (2), 353-8.

42. Haapasalo, A.; Kovacs, D. M., The many substrates of presenilin/gamma-secretase. *J Alzheimers Dis* **2011,** *25* (1), 3-28.

43. Ren, Y.; Yang, S.; Xu, S.; Gao, M.; Huang, W.; Gao, T.; Fang, Q.; Quan, C.; Zhang, C.; Sun, L.; Liang, Y.; Han, J.; Wang, Z.; Zhang, F.; Zhou, Y.; Liu, J.; Zhang, X., Genetic variation of promoter sequence modulates XBP1 expression and genetic risk for vitiligo. *PLoS Genet* **2009,** *5* (6), e1000523.

44. Nornes, S.; Newman, M.; Wells, S.; Verdile, G.; Martins, R. N.; Lardelli, M., Independent and cooperative action of Psen2 with Psen1 in zebrafish embryos. *Exp Cell Res* **2009,** *315* (16), 2791-801.

45. Ralser, D. J.; Basmanav, F. B.; Tafazzoli, A.; Wititsuwannakul, J.; Delker, S.; Danda, S.; Thiele, H.; Wolf, S.; Busch, M.; Pulimood, S. A.; Altmuller, J.; Nurnberg, P.; Lacombe, D.; Hillen, U.; Wenzel, J.; Frank, J.; Odermatt, B.; Betz, R. C., Mutations in gamma-secretase subunit-encoding PSENEN underlie Dowling-Degos disease associated with acne inversa. *J Clin Invest* **2017,** *127* (4), 1485-1490.

46. Oberto, J.; Breuil, N.; Hecker, A.; Farina, F.; Brochier-Armanet, C.; Culetto, E.; Forterre, P., Qri7/OSGEPL, the mitochondrial version of the universal Kae1/YgjD protein, is essential for mitochondrial genome maintenance. *Nucleic Acids Res* **2009,** *37* (16), 5343-52.

47. Lin, H.; Miyauchi, K.; Harada, T.; Okita, R.; Takeshita, E.; Komaki, H.; Fujioka, K.; Yagasaki, H.; Goto, Y. I.; Yanaka, K.; Nakagawa, S.; Sakaguchi, Y.; Suzuki, T., CO2-sensitive tRNA modification associated with human mitochondrial disease. *Nat Commun* **2018,** *9* (1), 1875.

48. Zhou, J. B.; Wang, Y.; Zeng, Q. Y.; Meng, S. X.; Wang, E. D.; Zhou, X. L., Molecular basis for t6A modification in human mitochondria. *Nucleic Acids Res* **2020,** *48* (6), 3181-3194.

49. Chacon, A. H.; Morrison, B.; Hu, S., Acquired hemochromatosis with pronounced pigment deposition of the upper eyelids. *J Clin Aesthet Dermatol* **2013,** *6* (10), 44-6.

50. Granstein, R. D.; Sober, A. J., Drug- and heavy metal--induced hyperpigmentation. *J Am Acad Dermatol* **1981,** *5* (1), 1-18.

51. Chevrant-Breton, J.; Simon, M.; Bourel, M.; Ferrand, B., Cutaneous manifestations of idiopathic hemochromatosis. Study of 100 cases. *Arch Dermatol* **1977,** *113* (2), 161-5.

52. Robert, P.; Zurcher, H.; Schmidli, B., [Studies on pigmentation. IV. Iron and copper content of normal and affected skin, particularly in case of vitiligo and relations to pigmentation]. *Dermatologica* **1953,** *106* (3-5), 200-18.

53. Pavel, S.; van Nieuwpoort, F.; van der Meulen, H.; Out, C.; Pizinger, K.; Cetkovska, P.; Smit, N. P.; Koerten, H. K., Disturbed melanin synthesis and chronic oxidative stress in dysplastic naevi. *Eur J Cancer* **2004,** *40* (9), 1423-30.

54. Ng, A. P.; Howe Fong, J.; Sijin Nin, D.; Hirpara, J. L.; Asou, N.; Chen, C. S.; Pervaiz, S.; Khan, M., Cleavage of misfolded nuclear receptor corepressor confers resistance to unfolded protein response-induced apoptosis. *Cancer Res* **2006,** *66* (20), 9903-12.

55. Costessi, A.; Mahrour, N.; Sharma, V.; Stunnenberg, R.; Stoel, M. A.; Tijchon, E.; Conaway, J. W.; Conaway, R. C.; Stunnenberg, H. G., The human EKC/KEOPS complex is recruited to Cullin2 ubiquitin ligases by the human tumour antigen PRAME. *PLoS One* **2012,** *7* (8), e42822.

56. Ikeda, H.; Lethe, B.; Lehmann, F.; van Baren, N.; Baurain, J. F.; de Smet, C.; Chambost, H.; Vitale, M.; Moretta, A.; Boon, T.; Coulie, P. G., Characterization of an antigen that is recognized on a melanoma showing partial HLA loss by CTL expressing an NK inhibitory receptor. *Immunity* **1997,** *6* (2), 199-208.

57. Epping, M. T.; Wang, L.; Edel, M. J.; Carlee, L.; Hernandez, M.; Bernards, R., The human tumor antigen PRAME is a dominant repressor of retinoic acid receptor signaling. *Cell* **2005,** *122* (6), 835-47.

58. Peng, J.; He, M. H.; Duan, Y. M.; Liu, Y. T.; Zhou, J. Q., Inhibition of telomere recombination by inactivation of KEOPS subunit Cgi121 promotes cell longevity. *PLoS Genet* **2015,** *11* (3), e1005071.

59. Kato, Y.; Kawasaki, H.; Ohyama, Y.; Morishita, T.; Iwasaki, H.; Kokubo, T.; Hirano, H., Cell polarity in Saccharomyces cerevisiae depends on proper localization of the Bud9 landmark protein by the EKC/KEOPS complex. *Genetics* **2011,** *188* (4), 871-82.

60. Kisseleva-Romanova, E.; Lopreiato, R.; Baudin-Baillieu, A.; Rousselle, J. C.; Ilan, L.; Hofmann, K.; Namane, A.; Mann, C.; Libri, D., Yeast homolog of a cancer-testis antigen defines a new transcription complex. *EMBO J* **2006,** *25* (15), 3576-85.

61. Peggion, C.; Lopreiato, R.; Casanova, E.; Ruzzene, M.; Facchin, S.; Pinna, L. A.; Carignani, G.; Sartori, G., Phosphorylation of the Saccharomyces cerevisiae Grx4p glutaredoxin by the Bud32p kinase unveils a novel signaling pathway involving Sch9p, a yeast member of the Akt / PKB subfamily. *FEBS J* **2008,** *275* (23), 5919-33.

62. Banci, L.; Camponeschi, F.; Ciofi-Baffoni, S.; Muzzioli, R., Elucidating the Molecular Function of Human BOLA2 in GRX3-Dependent Anamorsin Maturation Pathway. *J Am Chem Soc* **2015,** *137* (51), 16133-43.

63. Frey, A. G.; Palenchar, D. J.; Wildemann, J. D.; Philpott, C. C., A Glutaredoxin.BolA Complex Serves as an Iron-Sulfur Cluster Chaperone for the Cytosolic Cluster Assembly Machinery. *J Biol Chem* **2016,** *291* (43), 22344-22356.

64. Hecker, A.; Leulliot, N.; Gadelle, D.; Graille, M.; Justome, A.; Dorlet, P.; Brochier, C.; Quevillon-Cheruel, S.; Le Cam, E.; van Tilbeurgh, H.; Forterre, P., An archaeal orthologue of the universal protein Kae1 is an iron metalloprotein which exhibits atypical DNA-binding properties and apurinic-endonuclease activity in vitro. *Nucleic Acids Res* **2007,** *35* (18), 6042-51.

65. Facchin, S.; Ruzzene, M.; Peggion, C.; Sartori, G.; Carignani, G.; Marin, O.; Brustolon, F.; Lopreiato, R.; Pinna, L. A., Phosphorylation and activation of the atypical kinase p53-related protein kinase (PRPK) by Akt/PKB. *Cell Mol Life Sci* **2007,** *64* (19-20), 2680-9.

66. Cui, R.; Widlund, H. R.; Feige, E.; Lin, J. Y.; Wilensky, D. L.; Igras, V. E.; D'Orazio, J.; Fung, C. Y.; Schanbacher, C. F.; Granter, S. R.; Fisher, D. E., Central role of p53 in the suntan response and pathologic hyperpigmentation. *Cell* **2007,** *128* (5), 853-64.

67. Box, N. F.; Terzian, T., The role of p53 in pigmentation, tanning and melanoma. *Pigment Cell Melanoma Res* **2008,** *21* (5), 525-33.

68. Murase, D.; Hachiya, A.; Amano, Y.; Ohuchi, A.; Kitahara, T.; Takema, Y., The essential role of p53 in hyperpigmentation of the skin via regulation of paracrine melanogenic cytokine receptor signaling. *J Biol Chem* **2009,** *284* (7), 4343-53.

69. Hyter, S.; Coleman, D. J.; Ganguli-Indra, G.; Merrill, G. F.; Ma, S.; Yanagisawa, M.; Indra, A. K., Endothelin-1 is a transcriptional target of p53 in epidermal keratinocytes and regulates ultraviolet-induced melanocyte homeostasis. *Pigment Cell Melanoma Res* **2013,** *26* (2), 247-58.

70. Chang, C. H.; Kuo, C. J.; Ito, T.; Su, Y. Y.; Jiang, S. T.; Chiu, M. H.; Lin, Y. H.; Nist, A.; Mernberger, M.; Stiewe, T.; Ito, S.; Wakamatsu, K.; Hsueh, Y. A.; Shieh, S. Y.; Snir-Alkalay, I.; Ben-Neriah, Y., CK1alpha ablation in keratinocytes induces p53-dependent, sunburn-protective skin hyperpigmentation. *Proc Natl Acad Sci U S A* **2017,** *114* (38), E8035-E8044.

71. Kim, D. S.; Kim, S. Y.; Moon, S. J.; Chung, J. H.; Kim, K. H.; Cho, K. H.; Park, K. C., Ceramide inhibits cell proliferation through Akt/PKB inactivation and decreases melanin synthesis in Mel-Ab cells. *Pigment Cell Res* **2001,** *14* (2), 110-5.

72. Oka, M.; Nagai, H.; Ando, H.; Fukunaga, M.; Matsumura, M.; Araki, K.; Ogawa, W.; Miki, T.; Sakaue, M.; Tsukamoto, K.; Konishi, H.; Kikkawa, U.; Ichihashi, M., Regulation of melanogenesis through phosphatidylinositol 3-kinase-Akt pathway in human G361 melanoma cells. *J Invest Dermatol* **2000,** *115* (4), 699-703.

73. Lee, J. H.; Jang, J. Y.; Park, C.; Kim, B. W.; Choi, Y. H.; Choi, B. T., Curcumin suppresses alpha-melanocyte stimulating hormone-stimulated melanogenesis in B16F10 cells. *Int J Mol Med* **2010,** *26* (1), 101-6.

74. Lee, J.; Jung, K.; Kim, Y. S.; Park, D., Diosgenin inhibits melanogenesis through the activation of phosphatidylinositol-3-kinase pathway (PI3K) signaling. *Life Sci* **2007,** *81* (3), 249-54.

75. Kim, J. H.; Baek, S. H.; Kim, D. H.; Choi, T. Y.; Yoon, T. J.; Hwang, J. S.; Kim, M. R.; Kwon, H. J.; Lee, C. H., Downregulation of melanin synthesis by haginin A and its application to in vivo lightening model. *J Invest Dermatol* **2008,** *128* (5), 1227-35.

76. Tu, C. X.; Lin, M.; Lu, S. S.; Qi, X. Y.; Zhang, R. X.; Zhang, Y. Y., Curcumin inhibits melanogenesis in human melanocytes. *Phytother Res* **2012,** *26* (2), 174-9.

77. Mosavi, L. K.; Cammett, T. J.; Desrosiers, D. C.; Peng, Z. Y., The ankyrin repeat as molecular architecture for protein recognition. *Protein Sci* **2004,** *13* (6), 1435-48.

78. Jernigan, K. K.; Bordenstein, S. R., Tandem-repeat protein domains across the tree of life. *PeerJ* **2015,** *3*, e732.

79. Li, J.; Mahajan, A.; Tsai, M. D., Ankyrin repeat: a unique motif mediating protein-protein interactions. *Biochemistry* **2006,** *45* (51), 15168-78.

80. Yen, H. J.; Tayeh, M. K.; Mullins, R. F.; Stone, E. M.; Sheffield, V. C.; Slusarski, D. C., Bardet-Biedl syndrome genes are important in retrograde intracellular trafficking and Kupffer's vesicle cilia function. *Hum Mol Genet* **2006,** *15* (5), 667-77.

81. Ruiz-Larranaga, O.; Langa, J.; Rendo, F.; Manzano, C.; Iriondo, M.; Estonba, A., Genomic selection signatures in sheep from the Western Pyrenees. *Genet Sel Evol* **2018,** *50* (1), 9.

82. Choi, H.; Shin, J. H.; Kim, E. S.; Park, S. J.; Bae, I. H.; Jo, Y. K.; Jeong, I. Y.; Kim, H. J.; Lee, Y.; Park, H. C.; Jeon, H. B.; Kim, K. W.; Lee, T. R.; Cho, D. H., Primary Cilia Negatively Regulate Melanogenesis in Melanocytes and Pigmentation in a Human Skin Model. *PLoS One* **2016,** *11* (12), e0168025.

83. Chang, C. F.; Schock, E. N.; Attia, A. C.; Stottmann, R. W.; Brugmann, S. A., The ciliary baton: orchestrating neural crest cell development. *Curr Top Dev Biol* **2015,** *111*, 97-134.

84. Osborn, D. P.; Roccasecca, R. M.; McMurray, F.; Hernandez-Hernandez, V.; Mukherjee, S.; Barroso, I.; Stemple, D.; Cox, R.; Beales, P. L.; Christou-Savina, S., Loss of FTO antagonises Wnt signaling and leads to developmental defects associated with ciliopathies. *PLoS One* **2014,** *9* (2), e87662.

85. Srivastava, S.; Ramsbottom, S. A.; Molinari, E.; Alkanderi, S.; Filby, A.; White, K.; Henry, C.; Saunier, S.; Miles, C. G.; Sayer, J. A., A human patient-derived cellular model of Joubert syndrome reveals ciliary defects which can be rescued with targeted therapies. *Hum Mol Genet* **2017,** *26* (23), 4657-4667.

86. Lee, S. H.; Lee, M. S.; Choi, T. I.; Hong, H.; Seo, J. Y.; Kim, C. H.; Kim, J., MCRS1 associates with cytoplasmic dynein and mediates pericentrosomal material recruitment. *Sci Rep* **2016,** *6*, 27284.

87. Firestone, A. J.; Weinger, J. S.; Maldonado, M.; Barlan, K.; Langston, L. D.; O'Donnell, M.; Gelfand, V. I.; Kapoor, T. M.; Chen, J. K., Small-molecule inhibitors of the AAA+ ATPase motor cytoplasmic dynein. *Nature* **2012,** *484* (7392), 125-9.

88. Lomelino, C. L.; Andring, J. T.; McKenna, R.; Kilberg, M. S., Asparagine synthetase: Function, structure, and role in disease. *J Biol Chem* **2017,** *292* (49), 19952-19958.

89. Vogel, P.; Ding, Z. M.; Read, R.; DaCosta, C. M.; Hansard, M.; Small, D. L.; Ye, G. L.; Hansen, G.; Brommage, R.; Powell, D. R., Progressive Degenerative Myopathy and Myosteatosis in ASNSD1-Deficient Mice. *Vet Pathol* **2020**, 300985820939251.

90. Yu, Q.; Wang, X.; Wang, L.; Zheng, J.; Wang, J.; Wang, B., Knockdown of asparagine synthetase (ASNS) suppresses cell proliferation and inhibits tumor growth in gastric cancer cells. *Scand J Gastroenterol* **2016,** *51* (10), 1220-6.

91. Xu, Y.; Lv, F.; Zhu, X.; Wu, Y.; Shen, X., Loss of asparagine synthetase suppresses the growth of human lung cancer cells by arresting cell cycle at G0/G1 phase. *Cancer Gene Ther* **2016,** *23* (9), 287-94.

92. Panosyan, E. H.; Lasky, J. L.; Lin, H. J.; Lai, A.; Hai, Y.; Guo, X.; Quinn, M.; Nelson, S. F.; Cloughesy, T. F.; Nghiemphu, P. L., Clinical aggressiveness of malignant gliomas is linked to augmented metabolism of amino acids. *J Neurooncol* **2016,** *128* (1), 57-66.

93. Albert, A. E.; Adua, S. J.; Cai, W. L.; Arnal-Estape, A.; Cline, G. W.; Liu, Z.; Zhao, M.; Cao, P. D.; Mariappan, M.; Nguyen, D. X., Adaptive Protein Translation by the Integrated Stress Response Maintains the Proliferative and Migratory Capacity of Lung Adenocarcinoma Cells. *Mol Cancer Res* **2019,** *17* (12), 2343-2355.

94. Li, H.; Zhou, F.; Du, W.; Dou, J.; Xu, Y.; Gao, W.; Chen, G.; Zuo, X.; Sun, L.; Zhang, X.; Yang, S., Knockdown of asparagine synthetase by RNAi suppresses cell growth in human melanoma cells and epidermoid carcinoma cells. *Biotechnol Appl Biochem* **2016,** *63* (3), 328-33.

95. Palmer, E. E.; Hayner, J.; Sachdev, R.; Cardamone, M.; Kandula, T.; Morris, P.; Dias, K. R.; Tao, J.; Miller, D.; Zhu, Y.; Macintosh, R.; Dinger, M. E.; Cowley, M. J.; Buckley, M. F.; Roscioli, T.; Bye, A.; Kilberg, M. S.; Kirk, E. P., Asparagine Synthetase Deficiency causes reduced proliferation of cells under conditions of limited asparagine. *Mol Genet Metab* **2015,** *116* (3), 178-86.

96. Krall, A. S.; Xu, S.; Graeber, T. G.; Braas, D.; Christofk, H. R., Asparagine promotes cancer cell proliferation through use as an amino acid exchange factor. *Nat Commun* **2016,** *7*, 11457.

97. Toda, K.; Kawada, K.; Iwamoto, M.; Inamoto, S.; Sasazuki, T.; Shirasawa, S.; Hasegawa, S.; Sakai, Y., Metabolic Alterations Caused by KRAS Mutations in Colorectal Cancer Contribute to Cell Adaptation to Glutamine Depletion by Upregulation of Asparagine Synthetase. *Neoplasia* **2016,** *18* (11), 654-665.

98. Deng, L.; Yao, P.; Li, L.; Ji, F.; Zhao, S.; Xu, C.; Lan, X.; Jiang, P., p53-mediated control of aspartate-asparagine homeostasis dictates LKB1 activity and modulates cell survival. *Nat Commun* **2020,** *11* (1), 1755.

99. Barbosa-Tessmann, I. P.; Chen, C.; Zhong, C.; Siu, F.; Schuster, S. M.; Nick, H. S.; Kilberg, M. S., Activation of the human asparagine synthetase gene by the amino acid response and the endoplasmic reticulum stress response pathways occurs by common genomic elements. *J Biol Chem* **2000,** *275* (35), 26976-85.

100. Gjymishka, A.; Su, N.; Kilberg, M. S., Transcriptional induction of the human asparagine synthetase gene during the unfolded protein response does not require the ATF6 and IRE1/XBP1 arms of the pathway. *Biochem J* **2009,** *417* (3), 695-703.

101. Wang, X.; Zhang, X.; Chu, E. S. H.; Chen, X.; Kang, W.; Wu, F.; To, K. F.; Wong, V. W. S.; Chan, H. L. Y.; Chan, M. T. V.; Sung, J. J. Y.; Wu, W. K. K.; Yu, J., Defective lysosomal clearance of autophagosomes and its clinical implications in nonalcoholic steatohepatitis. *FASEB J* **2018,** *32* (1), 37-51.

102. Lin, H. H.; Chung, Y.; Cheng, C. T.; Ouyang, C.; Fu, Y.; Kuo, C. Y.; Chi, K. K.; Sadeghi, M.; Chu, P.; Kung, H. J.; Li, C. F.; Limesand, K. H.; Ann, D. K., Autophagic reliance promotes metabolic reprogramming in oncogenic KRAS-driven tumorigenesis. *Autophagy* **2018,** *14* (9), 1481-1498.

103. Noree, C.; Monfort, E.; Shotelersuk, V., Human asparagine synthetase associates with the mitotic spindle. *Biol Open* **2018,** *7* (12).

104. Dolinska, M. B.; Sergeev, Y. V., The consequences of deglycosylation of recombinant intra-melanosomal domain of human tyrosinase. *Biol Chem* **2017,** *399* (1), 73-77.

105. Coulombe, B.; Cloutier, P.; Gauthier, M. S., How do our cells build their protein interactome? *Nat Commun* **2018,** *9* (1), 2955.

106. Houry, W. A.; Bertrand, E.; Coulombe, B., The PAQosome, an R2TP-Based Chaperone for Quaternary Structure Formation. *Trends Biochem Sci* **2018,** *43* (1), 4-9.

107. Cloutier, P.; Poitras, C.; Faubert, D.; Bouchard, A.; Blanchette, M.; Gauthier, M. S.; Coulombe, B., Upstream ORF-Encoded ASDURF Is a Novel Prefoldin-like Subunit of the PAQosome. *J Proteome Res* **2020,** *19* (1), 18-27.

108. Gao, L.; van Nieuwpoort, F. A.; Out-Luiting, J. J.; Hensbergen, P. J.; de Snoo, F. A.; Bergman, W.; van Doorn, R.; Gruis, N. A., Genome-wide analysis of gene and protein expression of dysplastic naevus cells. *J Skin Cancer* **2012,** *2012*, 981308.

109. Zhou, C.; Guo, Z.; Xu, L.; Jiang, H.; Sun, P.; Zhu, X.; Mu, X., PFND1 Predicts Poor Prognosis of Gastric Cancer and Promotes Cell Metastasis by Activating the Wnt/beta-Catenin Pathway. *Onco Targets Ther* **2020,** *13*, 3177-3186.

110. Penate, X.; Praena-Fernandez, J. M.; Romero Pareja, P.; Enguix-Riego, M. D. V.; Payan-Bravo, L.; Vieites, B.; Gomez-Izquierdo, L.; Jaen Olasolo, J.; Rivin Del Campo, E.; Reyes, J. C.; Chavez, S.; Lopez Guerra, J. L., Overexpression of Canonical Prefoldin Associates with the Risk of Mortality and Metastasis in Non-Small Cell Lung Cancer. *Cancers (Basel)* **2020,** *12* (4).

111. Patel-King, R. S.; Sakato-Antoku, M.; Yankova, M.; King, S. M., WDR92 is required for axonemal dynein heavy chain stability in cytoplasm. *Mol Biol Cell* **2019,** *30* (15), 1834-1845.

112. Sanchez-Morgan, N.; Kirsch, K. H.; Trackman, P. C.; Sonenshein, G. E., UXT Is a LOX-PP Interacting Protein That Modulates Estrogen Receptor Alpha Activity in Breast Cancer Cells. *J Cell Biochem* **2017,** *118* (8), 2347-2356.

113. Yesseyeva, G.; Aikemu, B.; Hong, H.; Yu, C.; Dong, F.; Sun, J.; Zang, L.; Zheng, M.; Ma, J., Prefoldin subunits (PFDN1-6) serve as poor prognostic markers in gastric cancer. *Biosci Rep* **2020,** *40* (2).

114. Millan-Zambrano, G.; Chavez, S., Nuclear functions of prefoldin. *Open Biol* **2014,** *4* (7).

115. Patel-King, R. S.; King, S. M., A prefoldin-associated WD-repeat protein (WDR92) is required for the correct architectural assembly of motile cilia. *Mol Biol Cell* **2016,** *27* (8), 1204-9.

116. Abe, A.; Takahashi-Niki, K.; Takekoshi, Y.; Shimizu, T.; Kitaura, H.; Maita, H.; Iguchi-Ariga, S. M.; Ariga, H., Prefoldin plays a role as a clearance factor in preventing proteasome inhibitor-induced protein aggregation. *J Biol Chem* **2013,** *288* (39), 27764-76.

117. Sun, S.; Tang, Y.; Lou, X.; Zhu, L.; Yang, K.; Zhang, B.; Shi, H.; Wang, C., UXT is a novel and essential cofactor in the NF-kappaB transcriptional enhanceosome. *J Cell Biol* **2007,** *178* (2), 231-44.

118. Yin, Z.; Jiang, H.; Lee, E. S.; Ni, M.; Erikson, K. M.; Milatovic, D.; Bowman, A. B.; Aschner, M., Ferroportin is a manganese-responsive protein that decreases manganese cytotoxicity and accumulation. *J Neurochem* **2010,** *112* (5), 1190-8.

119. Choi, E. K.; Nguyen, T. T.; Iwase, S.; Seo, Y. A., Ferroportin disease mutations influence manganese accumulation and cytotoxicity. *FASEB J* **2019,** *33* (2), 2228-2240.

120. Madejczyk, M. S.; Ballatori, N., The iron transporter ferroportin can also function as a manganese exporter. *Biochim Biophys Acta* **2012,** *1818* (3), 651-7.

121. Troadec, M. B.; Ward, D. M.; Lo, E.; Kaplan, J.; De Domenico, I., Induction of FPN1 transcription by MTF-1 reveals a role for ferroportin in transition metal efflux. *Blood* **2010,** *116* (22), 4657-64.

122. Bregman, M. D.; Sawyer, T. K.; Hadley, M. E.; Hruby, V. J., Adenosine and divalent cation effects on S-91 melanoma adenylate cyclase. *Arch Biochem Biophys* **1980,** *201* (1), 1-7.

123. Yoshimoto, S.; Ohagi, Y.; Yoshida, M.; Yanagi, H.; Hibino, S.; Ichihashi, M.; Ando, H., Placental extracts regulate melanin synthesis in normal human melanocytes with alterations of mitochondrial respiration. *Exp Dermatol* **2019,** *28 Suppl 1*, 50-54.

124. Laddha, N. C.; Dwivedi, M.; Gani, A. R.; Shajil, E. M.; Begum, R., Involvement of superoxide dismutase isoenzymes and their genetic variants in progression of and higher susceptibility to vitiligo. *Free Radic Biol Med* **2013,** *65*, 1110-1125.

125. Seckin, H. Y.; Kalkan, G.; Butun, I.; Akbas, A.; Bas, Y.; Karakus, N.; Benli, I., Analysis of Manganese Superoxide Dismutase and Glutathione Peroxidase 1 Gene Polymorphisms in Vitiligo. *Biochem Genet* **2016,** *54* (4), 438-447.

126. Bisevac, J. P.; Djukic, M.; Stanojevic, I.; Stevanovic, I.; Mijuskovic, Z.; Djuric, A.; Gobeljic, B.; Banovic, T.; Vojvodic, D., Association Between Oxidative Stress and Melanoma Progression. *J Med Biochem* **2018,** *37* (1), 12-20.

127. Schadendorf, D.; Zuberbier, T.; Diehl, S.; Schadendorf, C.; Czarnetzki, B. M., Serum manganese superoxide dismutase is a new tumour marker for malignant melanoma. *Melanoma Res* **1995,** *5* (5), 351-3.

128. Hara, M.; Yaar, M.; Tang, A.; Eller, M. S.; Reenstra, W.; Gilchrest, B. A., Role of integrins in melanocyte attachment and dendricity. *J Cell Sci* **1994,** *107 ( Pt 10)*, 2739-48.

129. Searles, G. E.; Dixon, W. T.; Thomas, P. D.; Jimbow, K., Divalent cations control cell-substrate adhesion and laminin expression in normal and malignant human melanocytes in early and late stages of cellular differentiation. *J Invest Dermatol* **1995,** *105* (2), 301-8.

130. Lydolph, M. C.; Morgan-Fisher, M.; Hoye, A. M.; Couchman, J. R.; Wewer, U. M.; Yoneda, A., Alpha9beta1 integrin in melanoma cells can signal different adhesion states for migration and anchorage. *Exp Cell Res* **2009,** *315* (19), 3312-24.

131. Schallreuter, K. U.; Moore, J.; Wood, J. M.; Beazley, W. D.; Gaze, D. C.; Tobin, D. J.; Marshall, H. S.; Panske, A.; Panzig, E.; Hibberts, N. A., In vivo and in vitro evidence for hydrogen peroxide (H2O2) accumulation in the epidermis of patients with vitiligo and its successful removal by a UVB-activated pseudocatalase. *J Investig Dermatol Symp Proc* **1999,** *4* (1), 91-6.

132. Erway, L. C.; Fraser, A. S.; Hurley, L. S., Prevention of congenital otolith defect in pallid mutant mice by manganese supplementation. *Genetics* **1971,** *67* (1), 97-108.

133. Cotzias, G. C.; Tang, L. C.; Miller, S. T.; Sladic-Simic, D.; Hurley, L. S., A mutation influencing the transportation of manganese, L-dopa, and L-tryptophan. *Science* **1972,** *176* (4033), 410-2.

134. Cotzias, G. C.; Papavasiliou, P. S.; Miller, S. T., Manganese in Melanin. *Nature* **1964,** *201*, 1228-9.

135. Parkinson, K.; Baines, A. E.; Keller, T.; Gruenheit, N.; Bragg, L.; North, R. A.; Thompson, C. R., Calcium-dependent regulation of Rab activation and vesicle fusion by an intracellular P2X ion channel. *Nat Cell Biol* **2014,** *16* (1), 87-98.

136. Li, X.; Garrity, A. G.; Xu, H., Regulation of membrane trafficking by signalling on endosomal and lysosomal membranes. *J Physiol* **2013,** *591* (18), 4389-401.

137. Aballay, A.; Sarrouf, M. N.; Colombo, M. I.; Stahl, P. D.; Mayorga, L. S., Zn2+ depletion blocks endosome fusion. *Biochem J* **1995,** *312 ( Pt 3)*, 919-23.

138. Tomsig, J. L.; Suszkiw, J. B., Metal selectivity of exocytosis in alpha-toxin-permeabilized bovine chromaffin cells. *J Neurochem* **1996,** *66* (2), 644-50.

139. Capogna, M.; McKinney, R. A.; O’Connor, V.; Gähwiler, B.H.; Thompson S.M., Ca2+ or Sr2+ Partially Rescues Synaptic Transmission in Hippocampal Cultures Treated with Botulinum Toxin A and C, But Not Tetanus Toxin. *J Neurosci.* **1997**, 17(19), 7190–7202.

140. Xu-Friedman, M. A.; Regehr, W. G., Probing fundamental aspects of synaptic transmission with strontium. *J Neurosci* **2000,** *20* (12), 4414-22.

141. Kishimoto, T.; Liu, T. T.; Ninomiya, Y.; Takagi, H.; Yoshioka, T.; Ellis-Davies, G. C.; Miyashita, Y.; Kasai, H., Ion selectivities of the Ca(2+) sensors for exocytosis in rat phaeochromocytoma cells. *J Physiol* **2001,** *533* (Pt 3), 627-37.

142. Neves, G.; Neef, A.; Lagnado, L., The actions of barium and strontium on exocytosis and endocytosis in the synaptic terminal of goldfish bipolar cells. *J Physiol* **2001,** *535* (Pt 3), 809-24.

143. Searl, T. J.; Silinsky, E. M., Evidence for two distinct processes in the final stages of neurotransmitter release as detected by binomial analysis in calcium and strontium solutions. *J Physiol* **2002,** *539* (Pt 3), 693-705.

144. Fix, M.; Melia, T. J.; Jaiswal, J. K.; Rappoport, J. Z.; You, D.; Sollner, T. H.; Rothman, J. E.; Simon, S. M., Imaging single membrane fusion events mediated by SNARE proteins. *Proc Natl Acad Sci U S A* **2004,** *101* (19), 7311-6.

145. Bhalla, A.; Tucker, W. C.; Chapman, E. R., Synaptotagmin isoforms couple distinct ranges of Ca2+, Ba2+, and Sr2+ concentration to SNARE-mediated membrane fusion. *Mol Biol Cell* **2005,** *16* (10), 4755-64.

146. Starai, V. J.; Thorngren, N.; Fratti, R. A.; Wickner, W., Ion regulation of homotypic vacuole fusion in Saccharomyces cerevisiae. *J Biol Chem* **2005,** *280* (17), 16754-62.

147. Hay, J. C., Calcium: a fundamental regulator of intracellular membrane fusion? *EMBO Rep* **2007,** *8* (3), 236-40.

148. Chi, A.; Valencia, J. C.; Hu, Z. Z.; Watabe, H.; Yamaguchi, H.; Mangini, N. J.; Huang, H.; Canfield, V. A.; Cheng, K. C.; Yang, F.; Abe, R.; Yamagishi, S.; Shabanowitz, J.; Hearing, V. J.; Wu, C.; Appella, E.; Hunt, D. F., Proteomic and bioinformatic characterization of the biogenesis and function of melanosomes. *J Proteome Res* **2006,** *5* (11), 3135-44.

149. Ding, J.; Du, K., ClipR-59 interacts with Akt and regulates Akt cellular compartmentalization. *Mol Cell Biol* **2009,** *29* (6), 1459-71.

150. Ishida, M.; Ohbayashi, N.; Fukuda, M., Rab1A regulates anterograde melanosome transport by recruiting kinesin-1 to melanosomes through interaction with SKIP. *Sci Rep* **2015,** *5*, 8238.

151. Matsui, T.; Ohbayashi, N.; Fukuda, M., The Rab interacting lysosomal protein (RILP) homology domain functions as a novel effector domain for small GTPase Rab36: Rab36 regulates retrograde melanosome transport in melanocytes. *J Biol Chem* **2012,** *287* (34), 28619-31.

152. Nag, S.; Rani, S.; Mahanty, S.; Bissig, C.; Arora, P.; Azevedo, C.; Saiardi, A.; van der Sluijs, P.; Delevoye, C.; van Niel, G.; Raposo, G.; Setty, S. R. G., Rab4A organizes endosomal domains for sorting cargo to lysosome-related organelles. *J Cell Sci* **2018,** *131* (18).

153. Kawakami, A.; Sakane, F.; Imai, S.; Yasuda, S.; Kai, M.; Kanoh, H.; Jin, H. Y.; Hirosaki, K.; Yamashita, T.; Fisher, D. E.; Jimbow, K., Rab7 regulates maturation of melanosomal matrix protein gp100/Pmel17/Silv. *J Invest Dermatol* **2008,** *128* (1), 143-50.

154. Hirosaki, K.; Yamashita, T.; Wada, I.; Jin, H. Y.; Jimbow, K., Tyrosinase and tyrosinase-related protein 1 require Rab7 for their intracellular transport. *J Invest Dermatol* **2002,** *119* (2), 475-80.

155. Yatsu, A.; Shimada, H.; Ohbayashi, N.; Fukuda, M., Rab40C is a novel Varp-binding protein that promotes proteasomal degradation of Varp in melanocytes. *Biol Open* **2015,** *4* (3), 267-75.

156. Mahanty, S.; Ravichandran, K.; Chitirala, P.; Prabha, J.; Jani, R. A.; Setty, S. R., Rab9A is required for delivery of cargo from recycling endosomes to melanosomes. *Pigment Cell Melanoma Res* **2016,** *29* (1), 43-59.

157. Tarafder, A. K.; Bolasco, G.; Correia, M. S.; Pereira, F. J. C.; Iannone, L.; Hume, A. N.; Kirkpatrick, N.; Picardo, M.; Torrisi, M. R.; Rodrigues, I. P.; Ramalho, J. S.; Futter, C. E.; Barral, D. C.; Seabra, M. C., Rab11b mediates melanin transfer between donor melanocytes and acceptor keratinocytes via coupled exo/endocytosis. *J Invest Dermatol* **2014,** *134* (4), 1056-1066.

158. Beaumont, K. A.; Hamilton, N. A.; Moores, M. T.; Brown, D. L.; Ohbayashi, N.; Cairncross, O.; Cook, A. L.; Smith, A. G.; Misaki, R.; Fukuda, M.; Taguchi, T.; Sturm, R. A.; Stow, J. L., The recycling endosome protein Rab17 regulates melanocytic filopodia formation and melanosome trafficking. *Traffic* **2011,** *12* (5), 627-43.

159. Ohbayashi, N.; Yatsu, A.; Tamura, K.; Fukuda, M., The Rab21-GEF activity of Varp, but not its Rab32/38 effector function, is required for dendrite formation in melanocytes. *Mol Biol Cell* **2012,** *23* (4), 669-78.

160. Shakya, S.; Sharma, P.; Bhatt, A. M.; Jani, R. A.; Delevoye, C.; Setty, S. R., Rab22A recruits BLOC-1 and BLOC-2 to promote the biogenesis of recycling endosomes. *EMBO Rep* **2018,** *19* (12).

161. Bahadoran, P.; Aberdam, E.; Mantoux, F.; Busca, R.; Bille, K.; Yalman, N.; de Saint-Basile, G.; Casaroli-Marano, R.; Ortonne, J. P.; Ballotti, R., Rab27a: A key to melanosome transport in human melanocytes. *J Cell Biol* **2001,** *152* (4), 843-50.

162. Yu, M.; Kasai, K.; Nagashima, K.; Torii, S.; Yokota-Hashimoto, H.; Okamoto, K.; Takeuchi, T.; Gomi, H.; Izumi, T., Exophilin4/Slp2-a targets glucagon granules to the plasma membrane through unique Ca2+-inhibitory phospholipid-binding activity of the C2A domain. *Mol Biol Cell* **2007,** *18* (2), 688-96.

163. Menasche, G.; Pastural, E.; Feldmann, J.; Certain, S.; Ersoy, F.; Dupuis, S.; Wulffraat, N.; Bianchi, D.; Fischer, A.; Le Deist, F.; de Saint Basile, G., Mutations in RAB27A cause Griscelli syndrome associated with haemophagocytic syndrome. *Nat Genet* **2000,** *25* (2), 173-6.

164. Ohishi, Y.; Kinoshita, R.; Marubashi, S.; Ishida, M.; Fukuda, M., The BLOC-3 subunit HPS4 is required for activation of Rab32/38 GTPases in melanogenesis, but its Rab9 activity is dispensable for melanogenesis. *J Biol Chem* **2019,** *294* (17), 6912-6922.

165. Cizkova, M.; Cizeron-Clairac, G.; Vacher, S.; Susini, A.; Andrieu, C.; Lidereau, R.; Bieche, I., Gene expression profiling reveals new aspects of PIK3CA mutation in ERalpha-positive breast cancer: major implication of the Wnt signaling pathway. *PLoS One* **2010,** *5* (12), e15647.

166. Simmons, W. H.; Brecher, A. S., Inactivation of melanocyte-stimulating hormone release-inhibiting factor by a manganese-stimulated bovine brain aminopeptidase. *J Biol Chem* **1973,** *248* (16), 5780-4.

167. Celis, M. E.; Taleisnik, S., Formation of a melanocyte-stimulating hormone-release inhibiting factor by hypothalamic extracts from rats. *Int J Neurosci* **1971,** *1* (4), 223-30.

168. Nair, R. M.; Kastin, A. J.; Schally, A. V., Isolation and structure of hypothalamic MSH release-inhibition hormone. *Biochem Biophys Res Commun* **1971,** *43* (6), 1376-81.

169. Celis, M. E.; Taleisnik, S.; Walter, R., Regulation of formation and proposed structure of the factor inhibiting the release of melanocyte-stimulating hormone. *Proc Natl Acad Sci U S A* **1971,** *68* (7), 1428-33.

170. Celis, M. E., Hypothalamic peptides involved in the control of MSH secretion: identity, biosynthesis and regulation of their release. *Front Horm Res* **1977,** *4*, 69-79.

171. Celis, M. E.; Thody, A. J.; Fisher, C., Effect of Pro-Leu-Gly-NH(2) on plasma levels of ?-MSH in the rat. *Neurochem Int* **1982,** *4* (1), 27-31.

172. Scimonelli, T.; Celis, M. E., Inhibition by L-prolyl-L-leucyl-glycinamide (PLG) of alpha-melanocyte stimulating hormone release from hypothalamic slices. *Peptides* **1982,** *3* (6), 885-9.

173. Monnet, F.; Reubi, J. C.; Eberle, A.; Lichtensteiger, W., Diurnal variation in the release of alpha-MSH from rat hypothalamus and pituitary. *Neuroendocrinology* **1981,** *33* (5), 284-7.

174. Bernstein, K. A.; Baserga, S. J., The small subunit processome is required for cell cycle progression at G1. *Mol Biol Cell* **2004,** *15* (11), 5038-46.

175. Ferguson, B.; Handoko, H. Y.; Mukhopadhyay, P.; Chitsazan, A.; Balmer, L.; Morahan, G.; Walker, G. J., Different genetic mechanisms mediate spontaneous versus UVR-induced malignant melanoma. *Elife* **2019,** *8*.

176. Maurus, K.; Hufnagel, A.; Geiger, F.; Graf, S.; Berking, C.; Heinemann, A.; Paschen, A.; Kneitz, S.; Stigloher, C.; Geissinger, E.; Otto, C.; Bosserhoff, A.; Schartl, M.; Meierjohann, S., The AP-1 transcription factor FOSL1 causes melanocyte reprogramming and transformation. *Oncogene* **2017,** *36* (36), 5110-5121.

177. Hoja-Lukowicz, D.; Szwed, S.; Laidler, P.; Litynska, A., Proteomic analysis of Tn-bearing glycoproteins from different stages of melanoma cells reveals new biomarkers. *Biochimie* **2018,** *151*, 14-26.

178. Nakamoto, K.; Ito, A.; Watabe, K.; Koma, Y.; Asada, H.; Yoshikawa, K.; Shinomura, Y.; Matsuzawa, Y.; Nojima, H.; Kitamura, Y., Increased expression of a nucleolar Nop5/Sik family member in metastatic melanoma cells: evidence for its role in nucleolar sizing and function. *Am J Pathol* **2001,** *159* (4), 1363-74.

179. Xu, G.; Zhang, W.; Bertram, P.; Zheng, X. F.; McLeod, H., Pharmacogenomic profiling of the PI3K/PTEN-AKT-mTOR pathway in common human tumors. *Int J Oncol* **2004,** *24* (4), 893-900.

180. Kardos, G. R.; Dai, M. S.; Robertson, G. P., Growth inhibitory effects of large subunit ribosomal proteins in melanoma. *Pigment Cell Melanoma Res* **2014,** *27* (5), 801-12.

181. Kardos, G. R.; Robertson, G. P., Therapeutic interventions to disrupt the protein synthetic machinery in melanoma. *Pigment Cell Melanoma Res* **2015,** *28* (5), 501-19.

182. Ganesan, A. K.; Ho, H.; Bodemann, B.; Petersen, S.; Aruri, J.; Koshy, S.; Richardson, Z.; Le, L. Q.; Krasieva, T.; Roth, M. G.; Farmer, P.; White, M. A., Genome-wide siRNA-based functional genomics of pigmentation identifies novel genes and pathways that impact melanogenesis in human cells. *PLoS Genet* **2008,** *4* (12), e1000298.

183. Ho, H.; Kapadia, R.; Al-Tahan, S.; Ahmad, S.; Ganesan, A. K., WIPI1 coordinates melanogenic gene transcription and melanosome formation via TORC1 inhibition. *J Biol Chem* **2011,** *286* (14), 12509-23.

184. Jeffries, T. R.; Dove, S. K.; Michell, R. H.; Parker, P. J., PtdIns-specific MPR pathway association of a novel WD40 repeat protein, WIPI49. *Mol Biol Cell* **2004,** *15* (6), 2652-63.

185. Proikas-Cezanne, T.; Ruckerbauer, S.; Stierhof, Y. D.; Berg, C.; Nordheim, A., Human WIPI-1 puncta-formation: a novel assay to assess mammalian autophagy. *FEBS Lett* **2007,** *581* (18), 3396-404.

186. Proikas-Cezanne, T.; Waddell, S.; Gaugel, A.; Frickey, T.; Lupas, A.; Nordheim, A., WIPI-1alpha (WIPI49), a member of the novel 7-bladed WIPI protein family, is aberrantly expressed in human cancer and is linked to starvation-induced autophagy. *Oncogene* **2004,** *23* (58), 9314-25.
