# Supplementary material for: Fifteen Shades of Grey: Combined Analysis of Genome-Wide SNP Data in Steppe and Mediterranean Grey Cattle Sheds New Light on the Molecular Basis of Coat Color
Source: Genes (Basel). 2020 Aug 13;11(8):932. doi: 10.3390/genes11080932 (PMC7464420; doi:10.3390/genes11080932)
Supplement: Supplementary file 1 [file genes-11-00932-s001.zip › Supplementary file S2.docx]

**Supplementary file S2.** Functions of genes in the ± 250 kbp region upstream and downstream the locus Hapmap53144-ss46525999 on BTA4, detected as significant in 100% of the pair-wise contrasts performed using Holstein as reference breed.

***MYO1G*** (Myosin IG) belongs to the myosins, class I, single-headed, membrane-associated unconventional members of the myosin superfamily found in most eukaryotic cells. They bind actin filaments in an ATP-regulated manner. Because of their association with membranes, they have traditionally been viewed as motors that function primarily to transport membranous organelles along actin filaments. More recently, however, a wealth of roles for unconventional myosins that are not obviously related to organelle transport have been uncovered, including **membrane dynamics, cytoskeletal structure, mechanical signal-transduction, endosome processing**, **mitotic spindle regulation** and **gene transcription** [1,2].

Notably, *MYO1G* has been shown to encodes the human **minor histocompatibility antigen** HA-2 [3]. Also, it has been shown to **associates with the plasma membrane** in lymphocytes [4] and in Jurkat T‐cells where it has been shown to **contribute to the cell elasticity** [5,6]. To mount an immune response, T lymphocytes must successfully search for foreign material presented by major histocompatibility complex (MHC) molecules on antigen-presenting cells (APC). This search has been described as having features of a random-like motility pattern in lymph nodes. To optimize initial detection of antigens, a T cell must **balance migration speed with the need to dwell in a given location** for long enough to detect signaling complexes and become activated. *Myo1g* was indeed shown to transiently accumulate in discrete areas at the plasma membrane of migrating cells or when membranes are deformed and *Myo1g* deficient cells were shown to move faster and straighter [7], which ultimately proved to be a deficit, specifically for detection of rare antigens. This highlights that random walk motility is tuned and generates optimal combinations of speed and local dwell time. *Myo1g* contributes to this regulation by generating membrane tension and **modulating** cell-intrinsic meandering search and **interactions** **with** MHC-presenting **dendritic cells** during lymph-node surveillance [8].

*Myo1G* has been shown to be a key regulator of the phagocytic cups formed for internalization of the bound particle during opsonin receptor-mediated **phagocytosis** [9] and it has been identified as a **cytoskeleton-associated protein** whose depletion induces lysosomal cell death. Inactivation of *Myo1G* significantly increased the proportion of cells with an **enlarged endo-lysosomal (acidic) compartment,** **increased lysosomal protease activity**, and increased the number of both initial autophagic vacuoles and degradative autophagic vacuoles [10]. Maravillas-Montero et al. [11] reported the localization of Myo1g in B-cell membrane compartments such as lipid rafts, microvilli, and **membrane extensions formed during spreading**, detected abnormalities in the adhesion ability and chemokine-induced directed migration of lymphocytes after *Myo1g* inactivation and assessed a role for *Myo1g* in **phagocytosis** and **exocytosis** processes. More recently, Lopez-Ortega and Santos-Argumedo [12] highlighted that *Myo1g* contributes to **cell adhesion** and **cell migration** through modulation of lipid rafts and CD44 **recycling** in B lymphocytes. They indeed demonstrated that the lack of *Myo1g* decreases the cell-surface levels of CD44 and of a lipid raft surrogate, and that, in cells depleted of *Myo1g*, the recycling of CD44 was delayed, the delay seeming to be caused at the level of **formation of recycling complex and entry into recycling endosomes**, with impact on cell migration.

No direct evidence for involvement of *Myo1g* in the pigmentation process was found in the restricted literature on this target, and we hypothesize that the initial observation by Olety et al. [6], reporting protein expression exclusively in hematopoietic tissues and cells may have biased the following researches toward this compartment, while subsequent larger transcriptomic and proteomics projects revealed a much wider *Myo1g* expression (GeneCards ID: GC07M044962) that still deserve to be investigated for its functional significance. However, other class I unconventional myosins have been clearly implicated in **pigmentation**, as exemplified below, in a not exhaustive manner.

*MYO1B* (Myosin IB) encodes a motor protein that may participate in processes, particularly endosomal trafficking, critical to **neuronal** development and function (**cell migration, neurite outgrowth, vesicular transport**). Myosin IB has been shown to modulate the morphology and the protein transport within multi-vesicular sorting endosomes in a **human pigmented cell line** [13].

*MYO1E* codes for one of the several unconventional myosin heavy chains that may provide force for movement of membranes along actin filaments, and has been proposed to **correspond to the mouse *dilute* locus** [14] producing a **lightening of coat color caused by an abnormal adendritic melanocyte morphology**.

Finally, another member of the myosin family, *MYO5A*, has been shown to be **target of *MITF*** in **melanocytes** [15] and to be a major **actin-based vesicle transport motor** [16,17] that binds to one of its cargos, the **melanosome**. When one of the members of this receptor-motor complex is mutated, the melanosomes clump in the perinuclear region of the melanocyte and are transferred unevenly to the developing hair [18], leading to a **dilution of coat color** [19]. In humans, mutations in the *MYO5A* gene are associated with the **Griscelli syndrome**, characterized by **pigmentary dilution of the skin and the hair**, the presence of large clumps of pigment in hair shafts and an accumulation of melanosomes in melanocytes [20].

***LOC112446527*** (U6 spliceosomal RNA) small nuclear RNA

***PURB*** (Purine Rich Element Binding Protein B) encodes a nuclear protein implicated in several biological processes. It has been shown to have high level of expression in the cytoplasm of **neuronal cells** [21]. PUR proteins are single-stranded nucleic acid-binding proteins implicated in several processes such as injury-induced **repression** of genes encoding certain muscle-restricted isoforms of **actin and myosin** expressed in the heart, skeletal muscle, and vasculature [22-32], **transcriptional regulation** of other genes [33-38], **mRNA translation** [39,40,30], and **RNA transport** [41,42], initiation and regulation of **DNA replication** [43-45] and, in the case of the founding member of this family, PURA, a role in control of **cell cycle progression** [46-49] and in **cell proliferation** necessary for normal brain and myeloid cell development [50] has also been suggested. Simultaneous deletion of the two closely related gene family members, *PURA* and *PURB*, has been suggested to have consequences related to progression to **acute myelogenous leukemia** [51]. PUR proteins have been shown to attenuate baseline expression of the gene encoding the cyto-contractile protein **smooth muscle α-actin** in quiescent fibroblasts essential for myofibroblast-mediated **wound contraction** following tissue injury and wound healing. PUR α and β transcriptional repressors were shown to govern the DNA-binding activity of serum response factor (SRF) and phosphorylated Smad3 transcriptional activators during induction of smooth muscle α-actin gene expression in human pulmonary myofibroblasts, in a dynamic interplay between transcriptional activators and repressors in regulating smooth muscle α-actin gene output during fibroblast to **myofibroblast differentiation** [52]. Consistent with the role of PURB proteins as suppressor of myofibroblast differentiation, inactivation of PURB proteins in mouse embryo fibroblasts promoted **changes in cell morphology, actin isoform expression, and cell migration** indicative of conversion to a myofibroblast-like phenotype [53]. Transition of stromal cardiac fibroblasts into smooth muscle-like myofibroblasts is an early aspect of wound healing and fibrosis during native heart disease and after cardiac transplant. Perivascular fibrosis has been described as a significant pathobiologic feature of chronic rejection in heart transplant recipients that may limit long-term graft survival. Zhang et al. [25] highlighted that activation of the normally silent smooth muscle α-actin gene, mediated by PUR proteins, is part of the reprogramming process in cardiomyocytes from mouse hearts subjected to repeated transplant surgery and ischemia-reperfusion injury. Based on the above features, PUR proteins have been regarded as **transcriptional reprogramming proteins** [54].

PURB proteins were also shown to acts as **repressors of the *GnRH1* gene transcription** [55] through promoter binding in *GnRH1*-expressing neurons in the preoptic area of the hypothalamus (see Supplementary file S1 concerning the release of melanocyte-stimulating hormone from the hypothalamus, discussed by Scimonelli and Celis, 1982).

PUR proteins have been shown to bind the dendrite‐targeting motifs present in BC1 RNA [42,56] and have been hypothesized to be involved in the **transport** of BC1 RNA **along dendritic microtubules** and possibly co-operate in anchoring of BC1 RNA within these dendritic compartments. The BC1 RNA is a small **neuronal** non-coding RNA that can form **ribonucleoprotein particles** (RNP) in combination with RNA-binding proteins. BC1 RNAs are mediators of local translational control [57,58] through interaction with translation initiation factors [59] and are involved in the local regulation of protein synthesis in the synaptodendritic compartment that underlies **neuronal plasticity** [60,61].

More recently, PUR proteins were implicated in livestock traits of economic relevance. In particular, they were found to fall in a cluster of genes positively-correlated with female-biased **sexual size dimorphism** in fishes of the *Cynoglossus* species, a typical female heterogamete species in which female-biased sexual size dimorphism has severely hindered its sustainable development in aquaculture [62]. Also, PURB proteins have been shown to act as **positive regulators of amino acid-induced milk synthesis** in bovine mammary epithelial cells [63]. Indeed, PURB has been shown to be required for amino acids to stimulate **mTOR** and SREBP-1c gene expression, and to be a positive regulator of amino acid-induced **PI3K**-regulated milk protein and fat synthesis in bovine mammary epithelial cells.

***MIR4657*** Identified in 2011 as a new microRNAs by next generation sequencing of paired normal and tumor human breast tissue [64]. Detected as enriched in cancer drug-resistant HEK293tTS cells identified after cisplatin treatment [65]. Using a comparative genomic approach, it has been suggested to bind *MyD88* which is known to activate the immune response-inducing nuclear transcriptional factor **NF-kB** [66]. In a study aiming at deciphering a potential role for miRNAs in innate immune ontogeny, MIR4657 was found to be one of the primary transcripts (pri-miRNAs) induced in adult monocytes stimulated with LPS for 1 and for 6 hours compared to unstimulated adult monocytes, as well as in umbilical cord blood monocytes stimulated with LPS for 1 and for 6 hours compared to unstimulated umbilical cord blood monocytes [67]. Downregulated in serous ovarian carcinoma [68]. It was found in exosome isolated from conditioned media from two breast cancer cell populations (T47D and BT-200) while could not be detected in conditioned media from two other breast cancer cell populations (SKBR3 and MDA-MB-231) [69]. Downregulated in metformin-treated cholangiocarcinoma tumor cell lines [70]. Upregulated in rheumatic heart disease [71]. Upregulated in non-lesional compared with lesional psoriatic skin, with **TNF** being known to play an important role in the pathogenesis of **psoriasis** [72]. Upregulated in serum of alcohol-consuming pregnant women [73]. It was shown to be a differentially expressed miRNA in human gingival epithelial cells transfected with the miR-H1 viral microRNA from herpes simplex virus-1 [74]. It was among the miRNAs showing differential expression that were associated with survival for rectal cancer [75]. It was found to be under-expressed in “side population” (stem) cells sorted from human hepatocellular tissues compared to non-side population cells [76]. In a study carried out by Ohzawa et al. [77], HER2 positive breast cancer patients who underwent neoadjuvant chemotherapy with trastuzumab were sorted into pathological complete response (pCR) and non-pCR groups. Differential miRNA expression was observed between these pCR and non-pCR groups, with MIR4657 being observed as downregulated. It was shown to be one of the dysregulated microRNAs among those highly correlated with intervertebral disc degeneration grade [78]. By data mining of a pharmaco-clinical variants database, it was found to have FOS, a **psoriasis** associated gene, as target gene whose inferred transcription factors were ATF1, ATF2, ATF7, BACH2, BATF, BCL3, CEBPB, CEBPG, COBRA1, CREB1, DDIT3, EGR1, EGR2, ELK1, ELK3, ELK4, ESR1, ESR2, ETS1, ETS2, ETV4 and FLI1 [79]. In a study aiming to determine whether extracellular vesicle (EV)-derived miRNAs are differentially expressed in follicular fluid from individual mature follicles from subjects with and without polycystic ovarian syndrome, and if these differences are vesicle-specific and adiposity-dependent, microRNAs from lean subjects resulted to be associated with the regulation of transcription and cell apoptosis and, among them, MIR4657 was found in EV-depleted follicular fluid. It was also upregulated by transient overexpression of HNRNPA2/B1 in breast cancer cells at 48 h, but not at 72 h [80]. It was shown to be among the top downregulated exosomal miRNAs in patients with myocardial infarction [81]. In the bovine species, it was detected in the *Bos taurus* genome by using genes associated with bovine trypanosomosis as targets. It resulted to target the coding sequence of the following trypanosomosis associated genes: *CD14*, *ICAM-1*, *LBP*, *TLR-2*, ***TNF***. From annotated data in the human species, MIR4657, whose annotated Gene Ontology process was “Cellular protein modification process”, resulted to target the following genes: *CD14*, *ITGAM*, TLR-2, TLR-4, **TNF** [82]. It was shown to be among the microRNA differentially expressed in murine lung alveolar type II (ATII) cells 2 days post-infection with influenza A virus relative to ATII cells from uninfected mice [83]. By exploring the transcriptome regulated by a deletion mutation in exon 19 of the EGFR gene in lung carcinoma patients, they found it only slightly under-expressed in deleted vs non deleted patients [84].

Wang et al. [85] showed that **psoriasis TNF cytokines disrupt the pigment production in patients' melanocytes**. Decreased expression of the genes involved in pigmentation and, consistently, a suppressed melanin production was observed in normal human melanocytes treated with IL-17 and **TNF**. Also, see Supplementary file S4 for a link between **TNF/NF-κB signaling** and **pigmentation**.

In an *in silico* analysis of non-synonymous single nucleotide polymorphisms (SNPs) of *BMPR2* gene, MIR4657 was found as one of the microRNAs whose target binding was affected by the presence of SNPs in the *BMPR2* coding sequence [86]. *BMPR2* encodes a bone morphogenetic protein receptor 2 which is a serine/threonine kinase that plays a role in **cellular growth and differentiation** through phosphorylation and recruitment of BMPR1 receptor which phosphorylates **SMAD** transcription factors leading to gene regulation in nucleus. Bone morphogenetic proteins are the ligands for BMPR2. See Supplementary file S4 for a link between **BMP signaling** and embryonic **neural crest** and **pattern formation** (*FBXL15* gene). In addition, several studies have provided evidence for an involvement of BMP signaling in physiological **biogenesis of melanocytes, melanogenesis and pigmentation** [87-92] as well as in **melanoma** [93-97] and **psoriasis** [98]. Using mutant mice, Han et al. [99] showed that experimental reduction of BMPR2 and ACVR2A (Activin A Receptor Type 2A) function is reduced in melanocytes, **gray hair develops**, as melanosomes differentiate but **fail to grow**, resulting in organelle miniaturization.

***H2AFV*** Long non-coding RNA

***PPIA*** (Peptidylprolyl Isomerase A, alias *CYPA,* Cyclophilin A) encodes a peptidyl-prolyl isomerase A (PPIA), also known as cyclophilin A, an abundant, ubiquitously expressed protein, with the highest concentration in the central nervous system, that has been used in several studies as housekeeping gene for gene expression normalization. It is the intracellular ligand of the immunosuppressive drug cyclosporin A and has peptidyl-prolyl cis-trans isomerase (PPIase) activity, which is linked to its role in **protein folding and assembly**. Shieh et al. [100] described a reduction of **rhodopsin** in flies mutant at the *ninA* gene, which was shown to share relevant sequence identity with *PPIA*. Since the Rh1 opsin gene was found to be expressed at normal levels in *ninA* mutants, the authors suggested that the reduction of rhodopsin could be a post-transcriptional or a post-translational event, thus supporting the hypothesis that PPIA activity may be necessary for the correct **folding** and stability of rhodopsin. Besides the role as a folding catalyst, PPIA has been reported to act as a **molecular chaperone** [101]. PPIA has been linked to a number of human diseases, but its role in pathogenesis is still unknown [102]. The cyclophilin A protein was found to be **over-expressed in two melanoma cell lines as compared to melanocytes** [103,104] identified peptidylprolyl isomerase A as a differentially-expressed protein contrasting early submucosal non-invasive and invasive colorectal cancers. In the same tumor type, Yamamoto et al. [105] found that knockdown of *PPIA* significantly inhibited **cell migration and invasion** but had no effect on cell proliferation. In addition, knockdown of *PPIA* was associated with upregulation of E-cadherin and downregulation of N-cadherin and Snail expression, suggesting that *PPIA* knockdown inhibited cell migration and invasion by suppressing **epithelial-mesenchymal transition**.

In osteoblasts, PPIA has been shown to be necessary for **BMP-2** (Bone Morphogenetic Protein-2)-**induced** **SMAD** **phosphorylation**, exerting dual (pro-osteogenic and anti-osteoclastic) effects [106]. Downregulation of peptidylprolyl isomerase A has been shown to promote **cell death** and enhance doxorubicin-induced **apoptosis** in hepatocellular carcinoma [107]. It has been associated with **nervous system degeneration** [108,109] and identified as hallmark of familial **amyotrophic lateral sclerosis** (ALS) already at a presymptomatic stage in spinal cord of mutant *SOD1* animal models [108-110]. *SOD1* is a well-known cytosolic anti-oxidant enzyme that has been largely studied in connection with ALS. Mutant and/or oxidized **misfolded SOD1** is thought to escape the cell degradation machinery and impair the **proteasomal system and autophagy** [111,112], inducing a stress response by interfering with various cellular functions. TARDBP (also known as TDP-43) is an RNA binding protein normally localized in the nucleus, belonging to the heterogeneous nuclear ribonucleoprotein (hnRNP) family, and available evidence suggests that it has multiple roles in RNA processing and gene expression regulation [113]. Lauranzano et al. [114] provided evidence that *PPIA* is a molecular link between *TARDBP* and *SOD1* pathologies. Notably, they showed that peptidylprolyl isomerase A governs TARDBP function and assembly in heterogeneous nuclear ribonucleoprotein complexes and that the PPIA/TARDBP interaction was impaired in amyotrophic lateral sclerosis. As mentioned in Supplementary file S4, we recall here that **amyotrophic lateral sclerosis** is a **neurodegenerative disease** that is characterized by **axonal retraction** and subsequent loss of motor neurons [115].

Li et al. [116] found that cisplatin, a drug used in tumor therapy, could induce a **senescence-like phenotype** and growth arrest in a mouse neuroblastoma and rat glioma hybrid cell line. Using 2-dimension electrophoresis and comparing normal and senescent cells, they hence detected five differentially expressed proteins, including peptidylprolyl isomerase A (PPIA). In normal cells, terminal proliferative arrest may result from terminal differentiation or replicative senescence. Treating normal cells with **DNA-damaging** drugs rapidly induces terminal proliferative arrest, which is accompanied by an **accelerated senescence** phenotype. In addition to normal cells, cultures of human cancer cells derived from solid tumors tend to undergo accelerated senescence following exposure to low doses of DNA-damaging drugs, such as cisplatin. Accelerated senescence, associated with **proliferative arrest**, may be hence interpreted as a physiological mechanism of the **DNA damage response** that occurs during tumor therapy.

Yu et al. [117] showed that knock-down of PPIA and PPIA specific inhibitor treatment partially inhibited levels of CD147, the cellular receptor for cyclophilin A, which is also a multifunctional transmembrane glycoprotein playing a critical role in many pathological and physiological processes involving a variety of cell types such as various cancer cells, leukocytes, fibroblasts, and endothelial cells and able to stimulates fibroblast and endothelial cells to facilitate tumor invasion, metastasis, and angiogenesis. Decreased PPIA expression also significantly inhibited **STAT3** activity and expanded **estrogen responsiveness**.

In a human macrophage cell line, stimulation of CD147 (the cellular receptor for cyclophilin A) with its specific monoclonal antibody was shown to induce the expression of a matrix metalloproteinase, the phosphorylation of ERK, the phosphorylation-associated degradation of IκB, and the nuclear translocation of **NF-κB** p65 and p50 subunits [118]. The interconnection between cyclophilin A and **NF-κB** has been reported, among others, also in the studies by Bahmed et al. [119], Sun et al. [120] and Pasetto et al. [121]. The interconnection between the **NF-κB** pathway and the (JAK)-**STAT** signaling pathway has been reported, among others, in the works by Squarize et al. [122], Grivennikov and Karin [123], Olavarria et al. [124], McFarland et al. [125], Ahmad et al. [126], Czerkies et al. [127] and Haselager et al. [128]. Also, see Supplementary file S4 for the cross-talk between **NF-κB pathway** and **STAT** signaling. The STAT1 protein can be activated by various ligands including interferon-gamma and EGF. Persistent exposure to interferon-gamma has been shown to induce senescence in normal human melanocytes. Because interferon-gamma is present in various inflammatory conditions and is found to be elevated in the vitiliginous skin, it is possible that depigmentation in vitiligo arises from localized inflammation, where interferon-gamma interferes with the cell viability of surrounding melanocytes, leading to the senescence-driven melanocyte detachment [129]. A role for EGF, via its receptor, in **melanocyte biology and pathology** has been highlighted [130-133]. Also, STAT1 has been implicated in **UVB-induced melanocyte resistance to DNA damage and apoptosis** [134] and loss of sensitivity to interferon in melanoma cells [135].

***ZMIZ2*** (Zinc Finger MIZ-Type Containing 2, alias *ZIMP7*) together with *ZMIZ1* is a member of a PIAS (protein inhibitor of activated STAT)-like family of proteins that interact with nuclear hormone receptors. *ZMIZ2* **interacts with androgen receptors** (AR) and **enhances AR-mediated transcription** [136]. An interaction between ZMIZ2 and PIAS proteins, with higher preference for PIAS3, was reported by Peng et al. [137] who also demonstrated a role for PIAS proteins in modulating the activity of ZMIZ2 in androgen receptor-mediated transcription.

Interestingly, Rodríguez-Magadán et al. [138], by analyzing the gene expression pattern of the PIAS-like proteins, *ZMIZ1* and *ZMIZ2* in early gonadal development and during spermatogenesis, highlighted their **sexually dimorphic expression**, demonstrating that both genes are **up-regulated in male gonads** relative to fetal ovaries. Both genes were detectable in male gonads starting from 12.5 days post coitum (dpc), corresponding to the step characterized by the surrounding of the germ cells by Sertoli cells to form epithelial aggregates called the testis cords. At this stage, expression of *ZMIZ1* was limited, in the male, at the mesonephros, while, in female embryos, it was no longer evident in this compartment where it could be detected earlier. From 12.5 dpc to 14.5 dpc, *ZMIZ1* transcripts were increasingly detected in male gonads. Concerning *ZMIZ2*, in female gonads a very faint signal could be detected at 12.5–14.5 dpc compared to the more marked signal detectable in the same time-frame in the male gonad. Testis expression of *ZMIZ2* was also detected in the adult mouse. ***ZMIZ2****,* but not *ZMIZ1*, **expression** **is re-initiated prior to puberty**, after a period of silencing that occurs during the latter embryonic stages, and the ZMIZ2 protein co-localizes at the XY body in pachytene spermatocytes where they probably play a role in the meiotic process. Indeed, ZMIZ1 and ZMIZ2 have been included among the **components of the sumoylation machinery** [139] and ZMIZ1 expression was found to be deregulated in the majority of the papillary thyroid cancers (PTC) tissues screened by Tuccilli and co-workers, likely contributing to the PTC phenotype. Small Ubiquitin-like MOdifier (SUMO) proteins are small protein modifiers capable of regulating cellular localization and function of target proteins. Over the last few years, a relevant role has been demonstrated for **SUMOylation** in the modulation of important cellular processes, including **gene transcription, DNA repair, cell-cycle regulation and apoptosis**. Taylor and Labonne [140] highlighted that, during vertebrate development, the activities of individual group E **SOX** factors are well conserved and are regulated by **SUMOylation**. Group E SOX factors such as SOX9, and the closely related SOX10, are essential for the **formation of neural crest precursor cells**. In addition, upon interaction with EGR2, SOX10 has been shown to activate downstream genes [141] in Schwann cells, much alike the **SOX10-dependent cascade in melanocytes**, where SOX10 partners with the **MITF** protein to activate a downstream melanocyte-specific gene, ***DCT*** [142]. Based on these results, Adameyko et al. [143]formulated a general outline for the **development of Schwann cell precursors into myelinating Schwann cells or melanocytes** in which interactions of SOX genes with either EGR2 or MITF result in a commitment to myelinating Schwann cells or melanocytes, respectively. Mutations in *SOX10* can cause **Waardenburg syndrome**, a disorder that manifests with sensorineural deafness, **pigmentation defects of the skin, hair and iris** and various **defects of neural crest-derived tissues** [144]. Bertolotto et al. [145] described in **melanoma** patients a germline missense substitution in ***MITF***, which has been proposed to act as a melanoma oncogene, located in a small-ubiquitin-like modifier (SUMO) consensus site that **severely impaired SUMOylation of MITF**, increasing its transcriptional activity compared to wild-type MITF and enhancing **melanocytic clonogenicity, migration and invasion**, consistent with a gain-of-function role in tumorigenesis. Subsequently, Bonet et al. [146] demonstrated that the germline missense substitution in *MITF* **impaired the ability of human melanocytes to undergo senescence**.

ZMIZ1 has been suggested to act as a cofactor of Notch1 and to heterogeneously regulate **Notch** target genes [147,148] while ZIMZ2 has been shown to be required for **mesoderm development and dorsoventral patterning** [149]. Activation of signaling pathways including **Notch** are considered fundamental events in **epithelial to mesenchymal transition** (EMT) [150]. Consistently, Notch signaling was shown to play a role in neurogenesis and trunk and tail **neural crest development** [151]. Literature evidences of the roles of the Notch receptor in **melanocyte biology and pathology** have been provided [152,153]. Also, see the discussion concerning the *SUFU* gene in Supplementary file S4.

Zhu et al. [154] identified ZMIZ2 as one of the members of the LINC00265-ZMIZ2-β-catenin axis which they showed indispensable for colorectal tumorigenesis. LINC00265 is a long non coding RNA found to be upregulated and highly correlated with poor clinical outcome in human patients with colorectal cancer. LINC00265 acts as a miRNA sponge to enhance ZMIZ2 expression. Furthermore, ZMIZ2 recruits USP7 to stabilize **β-catenin**, which facilitates colorectal tumorigenesis. The **WNT/β-catenin signaling** is highly activated in colorectal cancer. Without WNT stimulation, cytoplasmic β-catenin is degraded, while, upon WNT activation, stabilized β-catenin may subsequently translocate to the nucleus where it binds to transcription factors and activates its transcriptional program. A role for ZMIZ2 in regulating the activity of the Wnt/β-catenin signaling pathway had been previously described also by Lee et al. [155]. Activation of signaling pathways including **Wnt/beta-catenin** are considered fundamental events in **epithelial to mesenchymal transition** (EMT) [150]. Also, see the discussion concerning the *PSD* gene and the *MIR146B* microRNA in Supplementary file S4.

ZMIZ1 has been shown to interact with **p53** and to transcriptionally activate it [156]. Literature evidences of a role for p53 in melanocyte biology and pathology have been provded [157-161]. ZMIZ1 has been shown also to enhance **SMAD** transcriptional activity and to play a role in regulating the **TGF-beta/SMAD signaling** pathway [162]. Literature evidences of the roles of SMAD in **melanocyte biology and pathology** have been provided Yang [163], Singh [89]. Also, see discussion concerning *PURB* and *PPIA* genes and the *MIR4657* microRNA above, as well as *MIR146B* microRNA in Supplementary file S4

***LOC112446406*** Uncharacterized locus

***OGDH*** (Oxoglutarate Dehydrogenase) encodes one subunit (E1) of the 2-oxoglutarate dehydrogenase complex. The complex is composed of E1 (2-oxoglutarate decarboxylase), E2 (dihydrolipoamide succinyl transferase), and E3 (dihydrolipoamide dehydrogenase) which couple 2-oxoglutarate oxidation to NADH formation. This complex catalyzes the overall conversion of 2-oxoglutarate (alpha-ketoglutarate) to succinyl-CoA and CO_2_ during the Krebs cycle. Liberated electrons are transferred through FAD to reduce NAD+ forming NADH. The NADH generated is utilized to drive the oxidative phosphorylation of ADP producing ATP. The protein is located in the mitochondrial matrix and uses thiamine pyrophosphate as a cofactor. The 2‐oxoglutarate substrate is generated both within the tricarboxylic acid cycle and through **glutamate transamination and oxidative deamination**. Hence, ***OGDH* sits at a major metabolic hub in the Krebs cycle linking carbon flux to the biosynthesis and degradation of amino acids** [164]. The ensuing role of OGDHC in the **degradation of glutamate**, which is **neurotoxic in excess**, is in accordance with the known association between reduced *OGDHC* activity and **neurodegeneration** [165-168]. For a discussion of the link between **glutamate metabolism and pigmentation**, see the section concerning the *ASNSD1* gene in Supplementary file S1. Furthermore, 2‐oxoglutarate takes part in metabolic signaling [169-173], and therefore its degradation by OGDHC may affect **metabolic plasticity** [174]. Duan et al. [175] highlighted that metabolites downstream of glycolysis (alpha ketoglutarate) could regulate **autophagy** and influence **apoptosis**. Notably, they proposed that glycolysis inhibition may increase apoptosis by reducing alpha ketoglutarate levels, which are regulated by **p53** and **OGDH**, thus reducing pro-survival autophagy. Indeed, autophagy is a cell survival mechanism that can become activated when nutrient and energy levels are limiting. During autophagy, cells degrade internal organelles and damaged proteins in auto-phagolysosomes. The degradation products are then recycled into metabolic pathways in order to maintain or restore nutrient and energy levels required for survival. Autophagy inhibition has been shown to contribute to apoptosis [175]. In **melanocytes**, **suppression of autophagy** was shown to causes **premature senescence** [176]. Autophagy has been shown to be active also in hair keratinocytes [177] and mesenchymal cells surrounding hair follicles [178]. However, **long-lived skin cells such as neurons and melanocytes may more strictly depend on autophagy for cellular homeostasis and normal execution of their functions during aging while rapidly renewing epidermal epithelium may better tolerate suppression of autophagy** [179]. Moreover, several of the steps in melanin production yield H_2_O_2_ and other free radicals. This places melanocytes under a higher **oxidative stress** **load** than for example keratinocytes, which is most prominent in hair follicle melanocytes because they produce large quantities of melanin constitutively throughout the anagen phase of the hair cycle. **Oxidative stress** generated outside hair follicle melanocytes, e.g., by UV-light induced, psycho-emotional, or inflammatory stress may add to this endogenous oxidative stress, **overwhelm the hair follicle melanocyte antioxidant capacity**, and speed up terminal damage accumulating for example in the aging hair follicle [180]. Consistently, a protective effect of superoxide dismutase against hair graying in a mouse model had been reported by Emerit et al. [181]. **The above evidences could explain why depigmentation in our cattle model is observed in hairs but not in skin.** Anatomical and functional differences between pigmentation in the skin and that of hair should also be taken into consideration to this regard. We recall here that each melanocyte is associated with five keratinocytes in the hair bulb forming a “hair follicle-melanin unit” while each melanocyte in the skin is associated with 36 keratinocytes constituting an “epidermal-melanin unit.”. Unlike in the skin where pigment production is continuous, melanogenesis in the hair is closely associated with stages of the hair cycle. Hair is actively pigmented in the anagen phase and is “turned off” during the catagen phase and absent during telogen. During anagen, a marked reduction in the number of melanocytes in the hair follicles through **autophagolysosomal degeneration** leading to pigment loss is thought to be **central in the pathogenesis of graying** [182].

2‐oxoglutarate degradation by OGDHC may also affect **signal transduction**, including **mitochondrial retrograde regulation** [183], broadly defined as the set of cellular **adaptive responses** to altered mitochondrial states that culminate in changes in nuclear gene expression whose outcome is usually a **reprogramming of metabolic, regulatory, or stress-related pathways**. Retrograde signaling has been involved in **aging**. In addition, mitochondria also appear to have a **stress response pathway** superficially **similar to the unfolded protein response in the endoplasmic reticulum** [184,185], Retrograde signaling has been shown to activate **NFκB** [186]. Retrograde signaling induces the expression of a number of **tumor-specific marker genes**, such as cathepsin L, an extracellular matrix protease, **TGFβ**, epiregulin, and **mouse melanoma antigen**. The retrograde signaling also induces changes in cell shape and **development of pseudopod-like structures** often seen in invasive tumor cells. *OGDH* is at the intercept of not only energy production and glutamate turnover, but also **mitochondrial production/scavenging of reactive oxygen species** (**ROS**) [187]. It is considered a **mitochondrial redox sensor** and is modulated by changes in **ROS**. It can produce **superoxide**, that is then rapidly dismutated by superoxide dismutase (SOD) yielding **hydrogen peroxide**. Thus, OGDH not only serves as a redox sensor but through ROS formation **directly influences the state of the surrounding redox environment [164].**

Wang et al. [188] showed that the signaling pathway of *AHR* (aryl hydrocarbon receptor), a ligand-activated transcription factor known to play pivotal roles in protecting cells against oxidative stress through regulating mitochondrial homeostasis, might have a major role in **protecting melanocytes against oxidative damage** via **inducing mitochondrial biogenesis**, while impaired AHR activation could cause defective repair of mitochondria and exacerbate oxidative damage-induced apoptosis in melanocytes.

Restoration of *OGDHL* expression in cervical cancer cells lacking endogenous *OGDHL* expression suppressed **cell proliferation, invasion** and soft agar **colony formation** *in vitro*. Knockdown of *OGDHL* expression in cervical cancer cells expressing endogenous *OGDHL* had the opposite effect. Forced expression of *OGDHL* increased the **production of reactive oxygen species (ROS)** **leading to apoptosis** through caspase 3 mediated **down-regulation of the AKT signaling cascade and decreased NF-κB phosphorylation**. Conversely, silencing *OGDHL* stimulated the signaling pathway via increased AKT phosphorylation. Moreover, the addition of caspase 3 or ROS inhibitors in the presence of OGDHL increased AKT signaling and cervical cancer cell proliferation [189].

**Reactive oxygen intermediates** have been shown to be a common denominator of **NF-kB activating signals**. More specifically, **hydrogen peroxide (H2O2)** might be used as **second messenger in the NF-kB system**, despite its cytotoxicity. Analysis of pathways leading to NF-kappa B activation in the nervous system has identified a number of ROI-dependent pathways such as cytokine- and neurotrophin-mediated activation, **glutamatergic** signal transduction, and various diseases with crucial ROI involvement (e.g., Alzheimer's disease, Parkinson's disease, multiple sclerosis, amyotrophic lateral sclerosis) [190].

Overexpression of *OGDH* in human gastric **cancer** (GC) **cells** resulted in the downregulation of the “**epithelial to mesenchymal transition**” molecular markers E-cadherin and *ZO-1*, the upregulation of N-cadherin and claudin-1. *OGDH* knockdown cells showed decreased mitochondrial membrane potential, oxygen consumption rate, intracellular ATP product, and **increased ROS level** and NADP+/NADPH ratio. Consistently, overexpression of *OGDH* enhanced the mitochondrial function in GC cells. Furthermore, *OGDH* knockdown reduced the expressions of **β-catenin**, slug and TCF8/ZEB1, and the downstream targets cyclin D1 and MMP9. OGDH overexpression facilitated the activation of **Wnt/β-catenin signal pathway**. Additionally, overexpression of OGDH promoted tumorigenesis of GC cells in nude mice [191]. In another study on the same cell type, OGDH was shown to mediate the inhibition of SIRT5 on **cell proliferation and migration** [191]. *OGDHC* was found to be differentially expressed in the retina of wild-type and homozygous **EGR1** knockout mice [192], with EGR1 (Early growth response protein 1) being a tumor protein **p53-responsive stress transcription factor** known to have a function in a variety of biologic processes (e.g., cell proliferation, brain plasticity and learning, apoptosis), also including **pigmentation**. Indeed, EGR1 is a transcription factor that is **upregulated by α-MSH** in hypothalamic neurons [193] and in human **epidermal melanocytes**. It has been shown to be upregulated after treatment of neuroblastoma cells with **melanin-concentrating hormone** (MCH) peptide which also induced a transient phosphorylation of MAPKinases, expression of phosphorylated **p53** proteins (an effect dependent upon MAPKinase activity) and increased phosphorylation of Elk-1 that, together with EGR1, is a transcriptional factors targeted by the MAPKinase pathway. Finally, MCH provoked **neurite outgrowth** after 24 h-treatment of neuroblastoma cells [194]. EGR1 induces **STAT3** gene expression [195] and the EGR1-STAT3 transcription factor axis regulates α-melanocyte-stimulating hormone-induced **tyrosinase gene transcription** **in melanocytes** [196]. Moreover, upregulation of **oxidative** and genotoxic **stress** **response genes**, including EGR1, coupled with **p53** activational phosphorylation, was observed in cultured human **metastatic melanoma cells** exposed to dihydroartemisinin (DHA), a major class of antimalarials that kill plasmodium parasites through induction of iron-dependent oxidative stress [197]. EGR1 expression was also found to be significantly increased in chicken fibroblasts overexpressing chicken Opsin 5, an UV-sensor, after **UV-A irradiation** for 30 min, suggesting that UV-A absorption by opsin 5 can upregulate the expression levels of EGR1 [198]. Perer et al. (2020), by examining the cutaneous effects of acute exposure of epidermal reconstructs to dihydroxyacetone (DHA), an agent responsible for **chemical (sunless) tanning** as a consequence of the formation of melanin-mimetic cutaneous pigments (**'melanoidins'**), observed a pronounced **cellular stress se** that was associated with overexpression, among other genes, of EGR1. Increase of EGR1 was also observed among the early molecular responses to quantified levels of serial oxidative stress (OS) in the human retinal pigment epithelium (RPE) [199]. During **long-term cultivation** of multipotent mesenchymal stromal cells, transcript levels of *EGR1*, a gene known to promote the **activation of proliferation** through **β-catenin**, **NF-κB**, and AP-1 signaling pathways [200,201], were found to be decreased compared to short-term cultivation [202] thus highlighting a possible role for this gene in **cell senescence**. Expression of EGR1 was found to be upregulated in zebrafish after inactivation of the MALAT-1 gene, that encodes a long non-coding RNA, whose knockdown was found to induce various phenotypic alterations including **reduced pigmentation** [203]. *EGR1* was found to be upregulated in **melanocytes exposed to oxidative stress** [204], a factor that is known to induce **melanocyte death in vitiligo** [205]. Furthermore, *OGDH* has been shown to be the source of the main **autoantigen** associated with the **autoimmune disease** “primary biliary cirrhosis” [206], in which an involvement of **mitophagy**, a **lysosomal-mediated degradation pathway**, as possible pathogenetic mechanism has been suggested [207]. **Mitophagy** serves as a crucial **quality control mechanism**. In line with this, it plays emerging critical roles in **senescence** (defective mitochondrial clearance is exacerbated in aged cells) and accumulation of unhealthy mitochondrion associates – or is possibly causative – to crucial pathological conditions, like diabetes mellitus [208], **neurodegeneration** (e.g. Parkinson and Alzheimer) [209,210], and **cancer**, including **melanoma** [211].

NRD1 was identified as a mitochondrial co-chaperone for *OGDH*, and provided a mechanistic link between **mitochondrial metabolic dysfunction**, **mTORC1 signaling**, and **impaired autophagy in neurodegeneration.** Indeed, loss of NRD1 or OGDH caused an increase in α-ketoglutarate, a substrate for OGDH, which in turn elicited mTORC1 activation and a subsequent **reduction in autophagy** [212].

Mitophagy may occur through Parkin‐dependent and Parkin‐independent processes. Parkin, a **ubiquitin E3 ligase**, adds ubiquitin to many substrates leading to interactions with LC3, a key component of autophagosomes. The process of Parkin‐dependent mitophagy is initiated by PINK1 accumulation in the outer mitochondrial membrane **in response to mitochondrial depolarization and damage**. PINK1 phosphorylates ubiquitin to activate the E3 ligase function of Parkin as well as the outer mitochondrial membrane protein MFN2 to serve as a Parkin receptor. Once recruited, Parkin ubiquitinates multiple outer mitochondrial membrane proteins (e.g., VDAC1, HDAC6, and mitofusin) leading to interaction with LC3 and initiation of mitophagy [208]. The mitochondrial matrix protein methionine sulfoxide reductase has also been shown to play a role in switching on mitophagy by reducing Parkin methionine oxidation [208]. Interestingly, reduced methionine sulfoxide reductase correlate with **senile hair graying** [213], **epidermal damage** [214], and **vitiligo** [215]. In the normal aging process, the most important effect of melanocyte dysfunction is graying of the hair. The follicular melanocytes which supply pigment to the hair respond to signals of the hair growth cycle, and their age-related **depletion** and **change in shape** [216] together with an **increase in redox stress** are implicated in age-related dysfunction of the hair follicle pigmentary unit [217].

An increasing number of studies indicate that autophagy in melanocytes may affect **age-related changes in melanogenesis, melanosome transfer, melanocyte cell death, melanocyte redox stress control, melanocyte proliferation rate, melanocyte senescence and inflammatory signaling** [179]. One of the possible connectors of **autophagy**, cell cycle control and **aging** in **melanocytes and melanoma** is the **p53 activating protein ARF**, which acts as emergency control of superoxide levels in mitochondrial dysfunction. See Supplementary file S4 for the role of the protein ***PSD*** in regulating signal transduction by activating ADP-ribosylation factor 6 (**ARF6**).

Also, patients with **xeroderma pigmentosum**, known for their increased risk for melanoma [218], have recently been reported to display **defective mitophagy** [219]. Mitophagy has also been suggested to occur post simulated **sunlight exposure** in cultured human skin amelanotic melanoma cells [220], and **glutamine and melanin** supplementation were shown to **inhibit mitophagy** **in melanoma cells** following simulated sunlight exposure. Evidences that increased **ROS** are required for stimulation of **mitophagy** have been accumulating [221]. As the case of mitochondrial respiration, melanin production and maturation in melanosomes also involves an inherent production of various free radicals. In normal melanocytes, **melanin** acts as a **scavenger** of UV‐ and intracellularly-generated **ROS**, preventing potentially hazardous **DNA damage** and protein oxidation. Notably, **autophagy-deficient keratinocytes** were shown to display **increased DNA damage, senescence and aberrant lipid composition after oxidative stress** in vitro and in vivo [222]. The melanin protective functions may be lost in **melanoma** cells, at least in part, resulting from excessive oxidation of melanin itself. Moreover, the production of ROS in melanoma cells is further enhanced by oncogenic activation, inflammation, glycolytic respiration, and deregulation of oxidoreductases or the **mitochondrial manganese-dependent SOD** (see the *SLC40A1* gene, Supplementary file S1) among others. In addition, a variety of melanoma‐associated defects on signaling cascades depending on **MAPK**, **AKT**, **NOTCH1**, and **NF‐κB** affect or are modulated by **ROS** production [223]. A theory on the **role of oxidative stress in graying of hair** was formulated by the above-mentioned work by Arck et al. [180].

The degradation of melanosome by autophagy is largely unknown. A functional genome screening study revealed that some **autophagy regulatory genes play a role in pigment accumulation** while another study showed that **autophagy affects skin color** determination by the regulation of melanin degradation in keratinocytes [224,225] occasionally found autophagosomes in **melanocytes from perilesional halo nevi skin, from stable vitiligo skin and from healthy control skin.** In contrast, in active vitiligo, autophagosomes could not be found anywhere. This result was interpreted by the authors as a sign of a dysfunction of mitophagy in melanocytes from perilesional vitiligo skin, with possible accumulation of damaged mitochondria, which may then expand the oxidant imbalance. However, the authors did not provide a direct evidence that the described autophagic mechanism was specifically targeting mitochondria. Evidences have suggested that **autophagy may play a role also in biogenesis/destruction of melanosomes** [226,227]. Small interfering RNA-based screens identified **autophagy genes** as having an **impact on melanogenesis** and **maturation of melanosomes,** and heterozygosity for the autophagy regulator beclin 1 results in altered fur color of mice [228]. Kim et al. [224], by using ARP101, a known inhibitor of Matrix Metallopeptidase 2, previously known to induce autophagy in various cancer cell types, provided a direct evidence that **autophagosomes engulf melanin and melanosomes**. They showed that ARP101 inhibits α-MSH-stimulated melanin synthesis by down-regulation of melanogenesis regulators (tyrosinase, TRP1, and MITF) in melanocytes, it induces autophagy in melanocytes, and that its anti-melanogenic effect is strictly dependent on autophagy activation. Finally, electron microscopy confirmed that autophagosomes engulfed melanin or melanosomes following combined treatment with α-MSH and ARP101. The results of this study suggest that **autophagy** is dispensable for melanogenesis but **important for the control of the stress response** and **sustained proliferation of melanocytes**. In 2019, Xiao et al. [229] reported that, while mice fetal hair follicle melanocytes were shown to occasionally contain autophagosomes, in cortical hair follicle keratinocytes, melanosome degradation clearly occurred in the phagolysosomes. Zhang et al. [176] demonstrate that the disruption of autophagy in melanocytes does not prevent melanogenesis, although it leads to a slight but significant **reduction in melanin** in both mice hair and tail epidermis. Cultured **autophagy-deficient melanocytes** showed a strongly **reduced proliferative capacity** and became **prematurely senescent**. At the molecular level, the lack of autophagy was associated with the **accumulation** of p62/SQSTM1 both *in vivo* and *in vitro*, the upregulation of nuclear factor E2–related factor 2 (Nrf2) signaling but also reactive oxygen species (**ROS**) and **lipid oxidation**. Mitochondrial autophagy has been shown to further increase cellular **ROS** levels that stimulate **lipid peroxidation and glutathione depletion**, leading to combined necroptotic and **ferroptotic cell death** [221]. Whether a similar phenomenon may occur as a consequence of dysregulated autophagy of melanin/melanosomes it is not known yet. Ferroptosis is a type of programmed cell death dependent on **iron** and characterized by the accumulation of lipid peroxides and is genetically and biochemically distinct from other forms of regulated cell death such as apoptosis. **Iron dysregulation** is associated with aberrant redox metabolism and **ROS accumulation**, feeding the enzymatic lipid peroxidation. See Supplementary file S1 for **iron** **homeostasis** (genes *SLC40A1* and *OSGEPL1*) and **pigmentation**. Intricate and complicated **interplay between ferroptosis, ionizing radiation** (IR), ATM (ataxia-telangiectasia mutated)/ATR (ATM and Rad3-related), and **tumor suppressor p53** have been described, which signifies the participation of the **DNA damage response** (DDR) **in iron-related cell death** [84]. **AKT** activation has been shown to be needed in cardiomyocytes to lower the tumor-induced mitophagy and autophagy, as well as **UPS** activity [230]. See the discussion concerning the *PMS1* and the *OSGEPL1* genes, and the role of **DNA-damage response in melanoma and vitiligo**, in Supplementary file S1.

***TMED4*** (Transmembrane P24 Trafficking Protein 4, alias Putative NF-Kappa-B-Activating Protein 156, alias Endoplasmic Reticulum Stress-Response Protein 25, alias P24 Family Protein Alpha-3) encodes a member of the Transmembrane p24 trafficking protein family, which includes four sub-families, alpha, beta, gamma and delta, with TMED4 belonging to sub-family alpha. These proteins play an important but poorly understood role in the **selective transport processes at the endoplasmic reticulum (ER)-Golgi interface**. The p24 proteins are abundantly present in **early secretory pathway membranes** and cycle continuously between these compartments. Proposed functions range from a role as **receptor for specific secretory cargoes** or as **machinery for COPI vesicle budding**, to the **supply of machinery proteins to secretory pathway sub-compartments** or in the **quality control of protein transport out of the ER,** and they have been suggested to be involved in **endoplasmic reticulum stress response** [231,232]. By using *Xenopus laevis* intermediate pituitary transgenic neuroendocrine **melanotrope cells** as a physiologically relevant, inducible cell model to study the p24 proteins, these authors (Strating et al., 2011) demonstrated that even p24 proteins from the same sub-family may have non-redundant roles in secretory cargo biosynthesis and processing of their major secretory cargo protein **proopiomelanocortin** (POMC), which proteolytically yields the bioactive **α-melanophore-stimulating hormone** (MSH). Rötter et al. [233] had previously demonstrated that TMED4, together with other but not all subunits of the p24 family, is **coordinately expressed with POMC.** Strating et al. [231] provided evidence that the transgene expression of TMED4 greatly **reduces POMC transport** and **leads to accumulation of the prohormone in large, ER-localized electron-dense structures**, as well as to an abnormal structural organization of the Golgi apparatus. In *C. elegans*, mutated p24 proteins damaged the supply of machinery cargo involved in the quality control system, **allowing the transport of a mutant cargo protein out of the ER** [234]. Another member of the p24 protein family, TMED5, was shown to be up-regulated by the MIR-G-1 microRNA in cervical cancer cells where increased TMED5 expression was shown to promote **cell proliferation, migration, invasion and EMT progression**. TMED5 was shown to interact with WNT7B activating the **WNT-CTNNB1/β-catenin pathway**. MIR-G-1 over-expression was shown to promote nuclear **autophagy** [235]. The Alzheimer disease associated TMED10 protein was shown to negatively **regulate autophagy** by inhibiting *ATG4B* activity [196].

Diseases associated with **TMED4** include Neuronal Ceroid Lipofuscinosis 8. See Supplementary files S1 and S4 for additional direct/indirect implications of candidate genes detected in our study (*ORMDL1, SLC40A1, ELOVL3, MFSD13A*) in pathogenesis of **neuronal ceroid lipofuscinosis**

***DDX56*** (DEAD-Box Helicase 56, alias *DDX21*) encodes a member of the DEAD box protein family. DEAD box proteins, characterized by the conserved motif Asp-Glu-Ala-Asp (DEAD), act principally as ATP-dependent RNA helicases. However, it is now thought that rather than being processive RNA helicases, several of these proteins may be acting as RNA ‘chaperones’, promoting the formation of optimal RNA structures through local RNA unwinding, or as RNPases by mediating RNA–protein association/dissociation. These proteins are now of major interest because they are known to play **important roles in virtually all aspects of RNA synthesis and function,** including transcriptional regulation, pre-mRNA processing, ribosome biogenesis, RNA turnover, RNA export and translation, processes that involve multi-step association/dissociation of large RNP complexes as well as the modulation of complex RNA structures [236,237]. Notably, *DDX56* is a nucleolar protein involved in the control of the two transcriptional arms of ribosome biogenesis: (i) synthesis and processing of the rRNA in the nucleolus, and (ii) transcription of ribosomal protein genes in the nucleoplasm. It has been shown to indirectly respond to dysfunction of *TCOF1*, a gene encoding a nucleolar protein that acts as a regulator of RNA polymerase I by connecting RNA polymerase I with enzymes responsible for ribosomal processing and modification. *TCOF1* mutations have been associated with abnormal craniofacial development primarily originating from **diminished allocation of cranial neural crest cells** into the first and second pharyngeal arches. This study nicely exemplifies how **nucleolar stress and rDNA damage may affect embryo development in a tissue–selective fashion**. Mutations in factors involved in ribosome biogenesis can lead to ribosomopathies, a collection of congenital disorders typically displaying tissue-selective defects, despite the broad requirement for ribosomes across growing tissues [238]. *DDX56* has been also shown to cooperate with SIRT7 in preventing accumulation of R-loops and the deriving **DNA damage**, thus safeguarding **genome integrity**. Moreover, DDX56 resolves **estrogen-induced R loops on estrogen-responsive genes** in breast cancer cells, which prevents the blocking of transcription elongation on these genes [222]. ***DDX56*** n**ucleolar compartmentalization** and **rRNA binding** was shown to be regulated by c-Jun, a well-established transcriptional regulator of AP-1-mediated gene expression that play an important role in the positive regulation of G1/S **cell cycle progression**, cancer cell **proliferation**, and **survival** [239].

The protein encoded by *DDX56* shows **ATPase activity** in the presence of polynucleotides and associates with nucleoplasmic 65S preribosomal particles. In colorectal cancer, *DDX56* expression was upregulated and high *DDX56* expression was associated with lymphatic **invasion** and distant **metastasis** and was an independent poor prognostic factor. DDX56 was shown to promote **proliferation** ability through **regulating the cell cycle**. *DDX56* knockdown reduced **intron retention** and expression of the tumor suppressor WEE1, which functions as a G2-M **DNA damage checkpoint** [240]. A role in cell proliferation for this gene was also reported in gastric cancer tissues. Indeed, *DDX21* was significantly up-regulated in gastric cancer tissues compared to paired adjacent normal tissues. The expression of DDX21 was closely related to the pathological stage of gastric cancer. *In vitro* and *in vivo* studies had shown that knockdown of *DDX21* inhibited gastric cancer cell **proliferation**, **colony formation**, G1/S **cell cycle** transition and xenograft growth, while ectopic expression of DDX21 promoted these cell functions. Mechanically, DDX21 induced gastric cancer cell growth by up-regulating levels of Cyclin D1 and CDK2 [241].

An interesting recent study by Santoriello et al. [242] demonstrated that DDX21/DDX56 **mediates nucleotide stress responses in neural crest and melanoma cells**. First, they showed that **progesterone**, which is an activator of the progesterone receptor PGR, rescued expression of neural crest markers crestin, SOX10, PAX3 and FOXD3 and **rescued elongation defects** observed in zebrafish treated with leflunomide. Leflunomide is an inhibitor of DHODH, which is an enzyme involved in *de novo* nucleotide biosynthesis, whose inhibition was previously shown to lower pyrimidine levels and block transcription elongation in neural crest and melanoma cells. Based on these evidences, a role for **progesterone** signaling in **overcoming transcriptional defects** was been proposed. Progesterone was also shown to reduce PGR levels by almost twofold in zebrafish embryos, possibly explaining why both exposure to progesterone and loss of its receptor yield the same biological effect *in vivo*. They found interactions between the progesterone receptor and proteins from two major biological processes. First, the progesterone receptor associated with enzymes involved in nucleotide metabolism, highlighting a function for the progesterone receptor in the regulation of nucleotide levels. They hence identified DDX21/DDX56 as a major interactor of the progesterone receptor and showed that **knock-down of DDX21/DDX56 rescued the neural crest following leflunomide** treatment. In addition, they observed that crestin rescue **recovered in vivo melanocyte differentiation potential**. **Pigmentation defects** characterizing leflunomide-treated embryos **were overcome by DDX21/DDX56 knockdown** (Fig. 3c). This established that **knockdown of DDX21/DDX56 confers resistance to nucleotide depletion**. Given the dual function of DDX21 in regulating mRNA transcription and rRNA transcription, processing and modifications, the authors addressed the issue whether DDX21/DDX56 may respond to changes in nucleotide levels and observed that its binding to rRNA was reduced by **nucleotide stress**, accompanied by a simultaneous increase in the binding of DDX21/DDX56 to mRNA. The shift in DDX21/DDX56 interaction from rRNA to mRNA was reversed by nucleotide supplementation, indicating that this effect is specific to a reduction of nucleotide pools. Based on their observation that DDX21/DDX56 engages mRNAs when there are low nucleotide levels, they investigated whether DHODH inhibition affected DDX21/DDX56 localization, observing partial **relocalization of DDX21/DDX56 from the nucleolus to the nucleoplasm**, which was reversed by nucleotide supplementation. These observations revealed that DDX21/DDX56 can interpret nucleotide levels and relocalize to the nucleoplasm when nucleotide pools are limited. They also showed that nucleotide stress negatively affects DDX21/DDX56 binding to chromatin, while chromatin binding was re-established by the addition of nucleotides. The reduced genomic occupancy of DDX21/DDX56 was found to correlate with gene expression changes, being associated with 32% of the genome that was at least twofold downregulated and 17% of genes showing significant upregulation. Gene set analysis of downregulated DDX21/DDX56 targets uncovered an enrichment in genes associated with RNA metabolism and the cell cycle. These observations could explain the transcriptional and proliferative defects seen in neural crest and melanoma cells after DHODH inhibition. In summary, limited nucleotide pools reduce the expression of DDX21/DDX56-bound genes.

Another recent work [243] highlighted that the metastasis-associated phosphatase of regenerating liver 3 (PRL3), a **p53 target gene**, binds to the RNA helicase DDX21/DDX56 impairing its binding and distribution on chromatin, thereby restricting productive transcription by RNA polymerase II. This PRL3-DDX21 transcriptional regulation was shown to be active in selectively downregulating **MITF-dependent endolysosomal genes** and represents a new regulatory mechanism of MITF-controlled gene expression, with MITF being essential for the proliferation and differentiation of melanocyte stem cell-derived melanocyte populations. In zebrafish, **this mechanism was shown to control premature melanoblast expansion and differentiation from melanocyte stem cells into melanocytes.** With this work, the authors hence provided evidence that **regulation of transcription elongation may prevent premature melanocyte stem cell differentiation** and reveal a new mechanism that regulates endolysosomal target genes via PRL3-DDX21-mediated inhibition. Since transcription through gene bodies is subject to dynamic changes in elongation rate and is a highly regulated process in development and differentiation, they proposed that the PRL3-DDX21 regulation of productive transcription elongation may function as a **fine-tuning mechanism for matching the regenerative response with tissue needs.** Unexpectedly, they did not identify enriched genes that are specific to the melanosome or to melanin synthesis. Rather, the endolysosomal genes regulated by PRL3 are associated with lysosomal-related acidic organelle **biosynthesis and trafficking** and with **autophagosome formation**. Thus, it seems likely that PRL3-dependent restrained transcriptional elongation of endolysosomal components leads to **more than simply delaying melanosome biogenesis** and points to a function in **maintaining a stem cell state**. The function of these vesicles in melanocyte stem cells differentiation is not known but may involve regulation of signaling pathways, such as the Notch pathway that is regulated by endomembrane vesicles and **inhibits premature differentiation of the melanocyte stem cells in the hair follicle niche.**

The DDX56 paralog *EIF-4A1* was shown to be consistently overexpressed in human melanoma cells *in vitro* and to contribute to the control of **melanoma cell proliferation** [244]. By investigating age-dependent alternative splicing (ADAS) in the context of postnatal organ growth in mice, mRNA of EIF4A2, another DDX56 paralog, was shown to undergo alternative splicing at high significance, with exon 11 included in the juvenile form and excluded from the adult form throughout the three studied compartments (cerebral cortex, cardiomyocytes and hepatocytes). The alternative splicing was mediated by SRSF7, a juvenile-specifically expressed splicing factor. The authors hence suggested that SRSF7 mediates juvenile patterns of alternative splicing, thus giving rise to the **juvenile transcriptome**. Mutation of SRSF7 causes **a premature switch from the juvenile to adult isoforms** of EIF4A2 both *in vitro* and *in vivo*, resulting in altered cellular juvenescence, which is instead characterized by the cellular capacities for growth, maturation, differentiation, anabolic metabolism, and **resistance to premature senescence**.

***NPC1L1*** (NPC1 Like Intracellular Cholesterol Transporter 1) is a p53 target gene [245] that encodes a multi-pass membrane protein. It contains a conserved N-terminal Niemann-Pick C1 (NPC1) domain and a putative **sterol-sensing domain** (SSD) functioning as a **plasma membrane to trans-Golgi network transport signal** in other proteins. NPC1L1 proteins **traffic** between the plasma membrane and intracellular compartments through the **endocytic recycling pathway**, which is regulated by cellular cholesterol availability. Acute cholesterol depletion **relocates** NPC1L1 **to the cell surface**, resulting in an increased uptake of free cholesterol through NPC1L1 [246,247]. Most of the **vesicular trafficking** events are dependent on the **cytoskeleton and motor proteins**. Chu et al. [248] provided evidence that the transport of NPC1L1 to the plasma membrane is dependent on the microfilament-associated myosin Vb/Rab11a/Rab11-FIP2 triple complex. Hence, this protein **takes up free cholesterol into cells through vesicular endocytosis.** As such it plays a critical role in the absorption of intestinal **cholesterol**. Studies are missing to show a possible role of NPC1L1 in melanocytes. Schallreuter et al. [249] highlighted that **epidermal melanocytes** hold the capacity for **autocrine cholesterol synthesis** as well as its **uptake** by the LDL receptor⁄Apo-B100 signal and showed the presence of cholesterol in the plasma membrane of melanocytes including the melanocyte-specific organelle (melanosome). Furthermore, they demonstrate that **cholesterol increases melanogenesis in epidermal melanocytes and melanoma cells**. Moreover, they demonstrated that **early stage melanosomes have significantly higher cholesterol content** compared with mature melanosomes.

In addition, cholesterol has been shown to play an important role in **dendrite differentiation** [250] and **synaptic activity** [251]. Cholesterol can be detected in detergent-insoluble membrane microdomains such as caveolae. **Caveolae** are specialized, invaginated plasma membrane domains that are defined morphologically and by the expression of signature proteins called, **caveolins**, which can bind cholesterol and requires cholesterol for oligomerization. Caveolae and caveolins are abundant in a variety of cell types where they play critical roles in **endocytosis** and transcytosis, mechano-transduction and mechano-protective role [252], cell fate (proliferation, survival, and differentiation), membrane lipid homeostasis, and signal transduction [253]. A recent work by Domingues et al. [254] reported an additional function for caveolae in **controlling melanin transfer to keratinocytes** and **epidermis pigmentation**. Interestingly, they found that caveolae distribute asymmetrically in melanocytes and the number of cultured melanocytes showing caveolae asymmetrically distributed doubled when co-cultured with keratinocytes, whereas co-culture with HeLa cells had no effect. Caveolae were shown to be preferentially located at the melanocyte–keratinocyte interface in human epidermis and to be **more abundant in melanocytes during UV-induced tissue pigmentation**. Most interesting, caveolin-1 was shown to **regulate cAMP production in melanocytes**, thus enhancing the activity of protein kinase A (PKA), through phosphorylation of the cAMP responsive element binding protein (CREB) and therefore upregulating the transcription of genes associated with pigmentation. Notably, caveolin-1 was shown to control **early signaling events in melanocytes** that affect the **transcriptional regulation of melanin-synthesizing enzymes, melanin production and melanosome maturation.** Caveolin 1 depleted melanocytes displayed an elongated shape and formed **fewer dendritic projections** and were mostly **unresponsive to** any contact made by **keratinocytes**. Hence, **caveolae in melanocytes have a key role in melanocyte dendrite outgrowth and the establishment and maintenance of contact with keratinocytes**. In particular, caveolae were shown to control the cAMP-dependent changes in shape and dendricity of melanocytes **by modulating the contractile force generated by the actomyosin subcortical network**. Loss of caveolae was shown to **impair melanin transfer** in 2D co-cultures of keratinocytes with Caveolin 1 depleted melanocytes and in a 3D model of skin epidermis. Consistently with the scenario depicted above, Schmitz et al [255] highlighted that, in oligodendrocytes, process formation is differentially affected by modulating the intra- and extracellular cholesterol content and that this cell type express NPC1L1.

**Cholesterol depletion** has been shown to **increase the expression of autophagy related genes and initiated autophagy**. Yu et al. [256] further investigated the **connection between cholesterol and autophagy** after brain ischemia using the specific inhibitor of the NPC1L1 protein. Inhibition of NPC1L1 induced autophagy through an AMPK-dependent mechanism, attenuating neuronal apoptosis. A role for *NPC1L1* in the regulation of autophagy was also suggested by Wang et al. [188] in a mouse model expressing human *NPC1L1* in the liver under conditions of alcohol consumption. It was concluded that NPC1L1 expression reduced hepatic autophagy.

An alternative pathway for the role of cholesterol in melanogenesis could be because of its central role as the **precursor for steroid hormone synthesis**. Here it seems important to remember that **cholesterol is the precursor for cortisol**, which is the main mediator for the hypothalamus–pituitary–adrenal (HPA)-axis. An **equivalent of this axis** has been identified in the human **skin** where **melanocytes** hold the capacity for **autocrine proopiomelanocortin (POMC) processing, catecholamine and acetylcholine synthesis** as well as the CRH⁄CRHR-1 signal. In addition, the influence of 17β-estradiol and other **estrogens** on **melanogenesis** has been the subject of many studies. Today it is well documented that **melanocytes and pigmented melanoma cells synthesize 17β-oestradiol** [249]. Natale et al. (2018) transiently exposed primary human **melanocytes** to estrogen or progesterone. Continuous **estrogen** exposure drove **increases in melanin production**, while **progesterone** had **opposite** **effects**. After hormone withdrawal, progesterone treated cells quickly returned to their baseline level of melanin production. In contrast, estrogen treated cells stably produced more melanin through continual cell divisions over the subsequent 50 days. A subset of cells that were exposed to transient estrogen were subsequently treated with progesterone. This reversed the estrogen effects, and melanin production decreased to the sub-baseline level seen upon initial progesterone treatment. Remarkably, after progesterone withdrawal, these cells fully returned back to the heightened level of melanin production induced by the initial estrogen exposure. In addition to increased melanin production, transient estrogen exposure was associated with stable **increases in well-established melanocyte differentiation proteins** including tyrosinase (TYR), p-CREB and MC1R. These results indicate that **estrogen signaling, even transiently, induces durable, long-lasting effects in melanocytes associated with markers of a more fully differentiated cell state**. Consistent results were obtained in melanoma cells, both when treating cells with estrogen or with the specific agonist of GPER (G protein-coupled estrogen receptor), previously demonstrated as the sole mediator of the estrogen and of the GPER agonist effects in normal primary human melanocytes [257]. In addition, treatment with the GPER agonist resulted in a dose-dependent **inhibition of melanoma proliferation**. To test whether transient GPER signaling may induce a persistent state in melanoma cells that affects subsequent tumor growth *in vivo*, they treated melanoma cells with estrogen, G-1, or vehicle *in vitro*, and subsequently injected equal numbers of treated cells into host mice. **Pretreatment with estrogen or G-1 markedly reduced subsequent tumor size**, indicating that transient GPER activation has durable, long-lasting effects on melanoma cells that limit tumor growth *in vivo*. GPER signaling in melanoma cells was shown to stably **deplete c-Myc** protein, a transcription factor that antagonizes differentiation and promotes proliferation and survival, and induced a relative **growth arrest**. A study suggests that **hormonal factors may upregulate GPER expression** [258]. See Supplementary files S1 and S4 for additional candidate genes (*ORMDL1, ELOVL3*) related with cholesterol synthesis/metabolism.

*NPC1L1* has also the ability to transport alpha-tocopherol (vitamin E) [259]. In addition, this protein may play a critical role in regulating **lipid metabolism**. Lack of *NPC1L1* activity leads to multiple lipid transport defects.

NPC1L1 has been associated with the **Niemann-Pick Disease** (NPD), a **lysosomal storage disease**. NPD disease is an autosomal recessive human lipidosis characterized by the accumulation of unesterified cholesterol in lysosomes. Studies using NPD fibroblasts demonstrated the inability of these cells to esterify endocytosed cholesterol, confirming the observation that cholesterol is **unable to exit the endosomal/lysosomal system.**

Hanson et al. [260] highlighted that in a certain number of NPD patients, **dermal melanocytosis** can be observed and may represent a cutaneous sign of the underlying lysosomal storage disease. They also describe two young patients with extensive dermal melanocytosis in association with two **lysosomal storage diseases**, GM1 **gangliosidosis** type 1 and **Hurler syndrome**, respectively. In addition, Schmuth et al. [261] highlighted that a fraction of patients with **NPD** showing grayish-brown **hyperpigmentation of the skin** as a cutaneous sign of the disease, also showed **abnormal permeability barrier homeostasis**, i.e., delayed recovery kinetics following acute barrier disruption by cellophane tape-stripping and found this being associated with severe acid-sphingomyelinase deficiency, thus demonstrating an important role for **enzymatic processing of sphingomyelin-to-ceramide** by acid-sphingomyelinase as a mechanism for generating a portion of the stratum corneum ceramides for permeability barrier homeostasis in mammalian skin.

Melanosomes are lysosome-related organelles (LROs). Cells that harbor these LROs must exploit specific mechanisms to **divert cargoes from conventional endocytic organelles** and deliver them to nascent LROs. Failure of these mechanisms can lead to LRO deficiencies and consequent disease [262]. The early endosomal network, comprising sorting and recycling endosomes, consists of vacuolar and tubulovesicular domains that promote trafficking to and from the cell surface or toward lysosomes or the trans-Golgi network [263]. Pre-melanosomes derive from **early endosomes** undergoing a specialization toward the biosynthesis and storage of melanins [264]. Biogenesis of melanosomes requires the transport of melanin-synthesizing enzymes **from tubular recycling endosomes to maturing melanosomes**. PMEL17 has been shown to enter melanosomes after internalization from the plasma membrane with kinetics consistent with receptor-mediated **endocytosis**. Melanosomal delivery of TYRP1 employs tubular **recycling endosomal transport** [265]. The **subversion of the endocytic pathway to form melanosomes in melanocytes** parallels similar processes in other cell types possessing specialized organelles and functions, including the modification of the early endosomal system for synaptic vesicle biogenesis in neurons and the development of secretory lysosomes [266]. Melanin transfer between donor melanocytes and acceptor keratinocytes occurs via coupled exo/**endocytosis** [267]. Depletion of syntaxin 13, a recycling endosomal Qa-SNARE, inhibits pigment granule maturation in melanocytes by rerouting the melanosomal proteins such as TYR and TYRP1 to lysosomes [268]. Based on the above, a role for NPC1L1 in pigmentation seems reasonable, although not yet investigated.

***NUDCD3*** (NudC Domain Containing 3, alias *NUDCL*) The product of this gene functions to **maintain the stability of dynein intermediate chain**. Depletion of this gene product results in aggregation and degradation of dynein intermediate chain, **mislocalization of the dynein complex** from kinetochores, spindle microtubules, and spindle poles, and loss of gamma-tubulin from spindle poles. The protein localizes to the Golgi apparatus during interphase, and levels of the protein increase after the G1/S transition.

A number of studies in *Aspergillus nidulans* have demonstrated that genes in the nuclear distribution (NUD) pathway are homologous to components or regulators of the cytoplasmic **dynein** complex. *NUDC* orthologs have been identified from fungi to mammals, which show high sequence and structure conservation. *NUDC* was originally identified as a **prolactin-inducible gene** in rat T cells [269]. Two paralogs of the mammalian NUDC, NudC-like (*NUDCL*), and NudC-like 2 (*NUDCL2*) genes were also identified, which are present in vertebrates. All NUDC gene products share a similar conserved **p23 domain**. p23 protein is a cochaperone of heat shock protein 90 (Hsp90) and partici**pates in the folding of various client proteins, such as progesterone receptor and estrogen receptor** [270]. Accumulating data have demonstrated that NUDC plays multiple roles in **cell cycle progression**, **neuronal migration**, inflammatory response, platelet production, and **ciliogenesis**. Either depletion or overexpression of NUDC has been shown to induce cytokinesis defects [271,272]. It was shown to play essential roles in regulating **actin dynamics** and ciliogenesis by stabilizing **cofilin 1** [273]. Depletion of NUDC causes the accumulation of bundled stress fibers and inhibits cell spreading and lamellipodia formation, indicating that NUDC is a crucial regulator of actin dynamics. Further results show that NudC colocalizes with cofilin 1 especially at the leading edge and influences the stability of cofilin 1 via an Hsp90-independent pathway. Knockdown of NUDC promotes **cilia elongation** and increases the percentage of ciliated cells, which is similar to those by actin cytoskeleton disruption or cofilin 1 depletion [273]. NUDCL has been reported to be essential for **cell cycle progression** and **cell viability** by stabilizing the dynein intermediate chain [274]. Depletion of NUDCL was found to induce **multiple mitotic defects**, including chromosome misalignment, multipolar spindles, failure of chromosome segregation, formation of dumbbell-like DNA structures, and accumulation of micronuclei, leading to cell death. Moreover, NUDCL was shown to bind to the dynein intermediate chain, and knockdown of NUDCL promoted degradation of the dynein intermediate chain. Additionally, overexpression of NUDCL that is localized to the centrosome and midbody results in **cytokinesis defects** and **inhibits cell proliferation**. NUDCL has been found to play a role in **retrograde mitochondrial motility** **in axons** [275]. Depletion of both NUDCL and NDEL1 almost blocks retrograde mitochondrial transport, suggesting these proteins may work together to regulate retrograde mitochondrial transport, possibly by linking the LIS1/ dynein complex [275]. Previous studies showed that the cytoplasmic dynein 2 complex is the motor for retrograde intraflagellar transport to drive the transport of activated components **from the cilia tip to the cell body** [276]. Depletion of dynein 2 components was shown to diminish the ability of cells to **generate primary cilia** [277]. Interestingly, NUDCL associates with dynein 2, and knockdown of NUDCL exhibits similar cilia phenotypes to that of dynein 2 depletion [278,279]. We recall here that cytoplasmic **dynein** is a well-known minus-end-directed microtubule motor present in most eukaryotic cells. Cytoplasmic dynein plays essential roles in many cellular processes, as it can hydrolysis ATP to generate force to move on and towards the minus end of the microtubule. Dynein is able to power the trafficking of various cargo towards microtubule minus ends including endosomes, lysosomes, components of the centrosomes, and mRNAs. It also contributes to Golgi positioning in the perinuclear region, nuclear rotation and positioning, centrosome separation, and nuclear envelope breakdown for entry into mitosis. During mitosis, dynein localizes to the kinetochore where it is thought to remove spindle-assembly checkpoint proteins by transporting them towards the spindle poles [280,282].

***LOC104972146*** Uncharacterized locus

***CAMK2B*** (Calcium/Calmodulin Dependent Protein Kinase II Beta) encodes the beta chain of a calcium/calmodulin dependent protein kinase, belonging to the serine/threonine protein kinase family, and composed of four different chains: alpha, beta, gamma, and delta. Calmodulin-dependent kinases (CaMK) mediate many of the second messenger effects of Ca^2+^. At basal Ca^2+^ levels, CaMKs are maintained in a dormant state through autoinhibition, which can be relieved by increases in Ca^2+^ levels. Calcium signaling is crucial for several aspects of **plasticity at glutamatergic synapses**. A link between **glutamate**, calcium/calmodulin-dependent protein kinases and **dendrite spines and synapses morphology** in brain neurons has been established [282,283]. Glutamate activates NMDA (N-methyl-d-aspartate) receptors, leading to large calcium influx which can in turn activate CaMKII. Activated CaMKII phosphorylates and activates calmodulin-dependent kinase kinase (CaMKK, CaMK1). These kinases form a multimolecular complex with the guanine-nucleotide exchange factor bPIX. Phosphorylation of this results in activation of Rac1 and Pak1. Pak1-mediated phosphorylation then activates LIM kinase (LIMK), which inactivates actin-depolymerizing protein **cofilin** (see above, the role of NUDC genes in stabilizing cofilin), hence promoting **stabilization of F-actin** and giving spine enlargement. In developing hippocampal neurons, promotes **arborization of the dendritic tree** and in mature neurons, promotes **dendritic remodeling**. Also regulates the **migration of developing neurons**. The CaMK pathway, alike the MAPK, PKC and PLC pathways, all converging on transcription factors like CREB or MEF-2, has been shown to be activated by **oxytocin** [284]. CAMK2B is involved in the **neurotrophin** signaling pathway [285], with neurotrophins known to affect melanocyte maturation [286] and pigmentation [287-290]. In a recent paper, Jiang et al. highlighted that, under **ER-stress conditions**, activated CAMK2B phosphorylates FAM134B, which enhances FAM134B oligomerization and activity in membrane fragmentation and **selective ER autophagy**. The endoplasmic reticulum (ER) is the largest intracellular organelle, which constitutes a continuous intracellular network of sheet and tubular membrane structures. ER plays essential roles in protein and lipid synthesis, calcium homeostasis, organelle communication and innate immunity. The oscillation of ER size and shape in response to varying environmental cues is crucial to cell homeostasis. Elimination of redundant ER is mediated by a selective autophagy pathway, which is coined as ER-phagy. Selective autophagy is a cellular quality control pathway through which a variety of autophagy cargoes are specifically engulfed by autophagosomes and delivered to lysosomes for degradation. The specificity of this process is governed by autophagy receptors that simultaneously bind to cargoes and the LC3 family members on the expanding autophagosomal membranes.

***YKT6*** (YKT6 V-SNARE Homolog) encodes a vesicular soluble NSF attachment protein receptor (v-SNARE) protein **implicated in vesicular transport** between secretory compartments. It is a membrane associated, isoprenylated protein that mediates vesicle docking and fusion to a specific acceptor cellular compartment. YKT6 is the most conserved SNARE protein in eukaryotes. It shows lipid-regulated conformational changes. Crucial SNARE activities, including vesicle-target protein recognition, association, and dissociation, are governed in part by conformational changes of SNARE proteins. In yeast, YKT6 functions in homotypic fusion of ER and vacuolar membranes, **retrograde Golgi trafficking and autophagosome formation** [291-295]. In higher eukaryotes, it seems to play a role in **non-canonical autophagosome formation** under starvation conditions. Loss of YKT6 was shown to lead to **large-scale accumulation of autophagosomes that are unable to fuse with lysosomes to form autolysosomes** [296] in Drosophila. It has been shown to regulate **epithelial cell migration** [297]. It is highly expressed in brain **neurons**. It was shown to have a conserved function in endosomal **Wnt trafficking** in Drosophila and in human cells, possibly a mechanism for fine-tuning of Wnt secretion in endosomes [298]. Cytosolic YKT6 is normally autoinhibited by a unique farnesyl-mediated regulatory mechanism; however, during **lysosomal stress**, it has been shown to activate and redistribute into membranes to preferentially promote hydrolase trafficking and enhance cellular clearance. α-Synuclein has been shown to aberrantly bind and deactivate YKT6 in Parkinson's patient-derived neurons, thereby **disabling the lysosomal stress response** and facilitating protein accumulation. Activating YKT6 by small-molecule farnesyltransferase inhibitors was shown to restore lysosomal activity and reduce α-synuclein in patient-derived neurons and mice [299]. We finally recall here that **syntaxins** also belongs to **SNAREs**, including ***STX17*** whose mutation has been shown to be responsible for the **grey color in horse breeds** [300].

**REFERENCES**

1. Woolner, S.; Bement, W. M., Unconventional myosins acting unconventionally. *Trends Cell Biol* **2009,** *19* (6), 245-52.

2. De La Cruz, E. M.; Ostap, E. M., Relating biochemistry and function in the myosin superfamily. *Curr Opin Cell Biol* **2004,** *16* (1), 61-7.

3. Pierce, R. A.; Field, E. D.; Mutis, T.; Golovina, T. N.; Von Kap-Herr, C.; Wilke, M.; Pool, J.; Shabanowitz, J.; Pettenati, M. J.; Eisenlohr, L. C.; Hunt, D. F.; Goulmy, E.; Engelhard, V. H., The HA-2 minor histocompatibility antigen is derived from a diallelic gene encoding a novel human class I myosin protein. *J Immunol* **2001,** *167* (6), 3223-30.

4. Patino-Lopez, G.; Aravind, L.; Dong, X.; Kruhlak, M. J.; Ostap, E. M.; Shaw, S., Myosin 1G is an abundant class I myosin in lymphocytes whose localization at the plasma membrane depends on its ancient divergent pleckstrin homology (PH) domain (Myo1PH). *J Biol Chem* **2010,** *285* (12), 8675-86.

5. Lopez-Ortega, O.; Ovalle-Garcia, E.; Ortega-Blake, I.; Antillon, A.; Chavez-Munguia, B.; Patino-Lopez, G.; Fragoso-Soriano, R.; Santos-Argumedo, L., Myo1g is an active player in maintaining cell stiffness in B-lymphocytes. *Cytoskeleton (Hoboken)* **2016,** *73* (5), 258-68.

6. Olety, B.; Walte, M.; Honnert, U.; Schillers, H.; Bahler, M., Myosin 1G (Myo1G) is a haematopoietic specific myosin that localises to the plasma membrane and regulates cell elasticity. *FEBS Lett* **2010,** *584* (3), 493-9.

7. Gerard, A.; Patino-Lopez, G.; Beemiller, P.; Nambiar, R.; Ben-Aissa, K.; Liu, Y.; Totah, F. J.; Tyska, M. J.; Shaw, S.; Krummel, M. F., Detection of rare antigen-presenting cells through T cell-intrinsic meandering motility, mediated by Myo1g. *Cell* **2014,** *158* (3), 492-505.

8. Stein, J. V., T Cell Motility as Modulator of Interactions with Dendritic Cells. *Front Immunol* **2015,** *6*, 559.

9. Dart, A. E.; Tollis, S.; Bright, M. D.; Frankel, G.; Endres, R. G., The motor protein myosin 1G functions in FcgammaR-mediated phagocytosis. *J Cell Sci* **2012,** *125* (Pt 24), 6020-9.

10. Groth-Pedersen, L.; Aits, S.; Corcelle-Termeau, E.; Petersen, N. H.; Nylandsted, J.; Jaattela, M., Identification of cytoskeleton-associated proteins essential for lysosomal stability and survival of human cancer cells. *PLoS One* **2012,** *7* (10), e45381.

11. Maravillas-Montero, J. L.; Lopez-Ortega, O.; Patino-Lopez, G.; Santos-Argumedo, L., Myosin 1g regulates cytoskeleton plasticity, cell migration, exocytosis, and endocytosis in B lymphocytes. *Eur J Immunol* **2014,** *44* (3), 877-86.

12. Lopez-Ortega, O.; Santos-Argumedo, L., Myosin 1g Contributes to CD44 Adhesion Protein and Lipid Rafts Recycling and Controls CD44 Capping and Cell Migration in B Lymphocytes. *Front Immunol* **2017,** *8*, 1731.

13. Salas-Cortes, L.; Ye, F.; Tenza, D.; Wilhelm, C.; Theos, A.; Louvard, D.; Raposo, G.; Coudrier, E., Myosin Ib modulates the morphology and the protein transport within multi-vesicular sorting endosomes. *J Cell Sci* **2005,** *118* (Pt 20), 4823-32.

14. Whitmer, J. D.; Koslovsky, J. S.; Bahler, M.; Mercer, J. A., Chromosomal location of three unconventional myosin heavy chain genes in the mouse. *Genomics* **1996,** *38* (2), 235-7.

15. Alves, C. P.; Yokoyama, S.; Goedert, L.; Pontes, C. L. S.; Sousa, J. F.; Fisher, D. E.; Espreafico, E. M., MYO5A Gene Is a Target of MITF in Melanocytes. *J Invest Dermatol* **2017,** *137* (4), 985-989.

16. Hume, A. N.; Ushakov, D. S.; Tarafder, A. K.; Ferenczi, M. A.; Seabra, M. C., Rab27a and MyoVa are the primary Mlph interactors regulating melanosome transport in melanocytes. *J Cell Sci* **2007,** *120* (Pt 17), 3111-22.

17. Robinson, C. L.; Evans, R. D.; Sivarasa, K.; Ramalho, J. S.; Briggs, D. A.; Hume, A. N., The adaptor protein melanophilin regulates dynamic myosin-Va:cargo interaction and dendrite development in melanocytes. *Mol Biol Cell* **2019,** *30* (6), 742-752.

18. Van Gele, M.; Geusens, B.; Schmitt, A. M.; Aguilar, L.; Lambert, J., Knockdown of myosin Va isoforms by RNAi as a tool to block melanosome transport in primary human melanocytes. *J Invest Dermatol* **2008,** *128* (10), 2474-84.

19. O'Sullivan, T. N.; Wu, X. S.; Rachel, R. A.; Huang, J. D.; Swing, D. A.; Matesic, L. E.; Hammer, J. A., 3rd; Copeland, N. G.; Jenkins, N. A., dsu functions in a MYO5A-independent pathway to suppress the coat color of dilute mice. *Proc Natl Acad Sci U S A* **2004,** *101* (48), 16831-6.

20. Menasche, G.; Pastural, E.; Feldmann, J.; Certain, S.; Ersoy, F.; Dupuis, S.; Wulffraat, N.; Bianchi, D.; Fischer, A.; Le Deist, F.; de Saint Basile, G., Mutations in RAB27A cause Griscelli syndrome associated with haemophagocytic syndrome. *Nat Genet* **2000,** *25* (2), 173-6.

21. Osugi, T.; Ding, Y.; Tanaka, H.; Kuo, C. H.; Do, E.; Irie, Y.; Miki, N., Involvement of a single-stranded DNA binding protein, ssCRE-BP/Pur alpha, in morphine dependence. *FEBS Lett* **1996,** *391* (1-2), 11-6.

22. Ferris, L. A.; Kelm, R. J., Jr., Structural and functional analysis of single-nucleotide polymorphic variants of purine-rich element-binding protein B. *J Cell Biochem* **2019,** *120* (4), 5835-5851.

23. Rumora, A. E.; Steere, A. N.; Ramsey, J. E.; Knapp, A. M.; Ballif, B. A.; Kelm, R. J., Jr., Isolation and characterization of the core single-stranded DNA-binding domain of purine-rich element binding protein B (Purbeta). *Biochem Biophys Res Commun* **2010,** *400* (3), 340-5.

24. Ramsey, J. E.; Kelm, R. J., Jr., Mechanism of strand-specific smooth muscle alpha-actin enhancer interaction by purine-rich element binding protein B (Purbeta). *Biochemistry* **2009,** *48* (27), 6348-60.

25. Zhang, A.; David, J. J.; Subramanian, S. V.; Liu, X.; Fuerst, M. D.; Zhao, X.; Leier, C. V.; Orosz, C. G.; Kelm, R. J., Jr.; Strauch, A. R., Serum response factor neutralizes Pur alpha- and Pur beta-mediated repression of the fetal vascular smooth muscle alpha-actin gene in stressed adult cardiomyocytes. *Am J Physiol Cell Physiol* **2008,** *294* (3), C702-14.

26. Ji, J.; Tsika, G. L.; Rindt, H.; Schreiber, K. L.; McCarthy, J. J.; Kelm, R. J., Jr.; Tsika, R., Puralpha and Purbeta collaborate with Sp3 to negatively regulate beta-myosin heavy chain gene expression during skeletal muscle inactivity. *Mol Cell Biol* **2007,** *27* (4), 1531-43.

27. Knapp, A. M.; Ramsey, J. E.; Wang, S. X.; Strauch, A. R.; Kelm, R. J., Jr., Structure-function analysis of mouse Pur beta II. Conformation altering mutations disrupt single-stranded DNA and protein interactions crucial to smooth muscle alpha-actin gene repression. *J Biol Chem* **2007,** *282* (49), 35899-909.

28. Subramanian, S. V.; Polikandriotis, J. A.; Kelm, R. J., Jr.; David, J. J.; Orosz, C. G.; Strauch, A. R., Induction of vascular smooth muscle alpha-actin gene transcription in transforming growth factor beta1-activated myofibroblasts mediated by dynamic interplay between the Pur repressor proteins and Sp1/Smad coactivators. *Mol Biol Cell* **2004,** *15* (10), 4532-43.

29. Gupta, M.; Sueblinvong, V.; Gupta, M. P., The single-strand DNA/RNA-binding protein, Purbeta, regulates serum response factor (SRF)-mediated cardiac muscle gene expression. *Can J Physiol Pharmacol* **2007,** *85* (3-4), 349-59.

30. Gupta, M.; Sueblinvong, V.; Raman, J.; Jeevanandam, V.; Gupta, M. P., Single-stranded DNA-binding proteins PURalpha and PURbeta bind to a purine-rich negative regulatory element of the alpha-myosin heavy chain gene and control transcriptional and translational regulation of the gene expression. Implications in the repression of alpha-myosin heavy chain during heart failure. *J Biol Chem* **2003,** *278* (45), 44935-48.

31. Kelm, R. J., Jr.; Wang, S. X.; Polikandriotis, J. A.; Strauch, A. R., Structure/function analysis of mouse Purbeta, a single-stranded DNA-binding repressor of vascular smooth muscle alpha-actin gene transcription. *J Biol Chem* **2003,** *278* (40), 38749-57.

32. Carlini, L. E.; Getz, M. J.; Strauch, A. R.; Kelm, R. J., Jr., Cryptic MCAT enhancer regulation in fibroblasts and smooth muscle cells. Suppression of TEF-1 mediated activation by the single-stranded DNA-binding proteins, Pur alpha, Pur beta, and MSY1. *J Biol Chem* **2002,** *277* (10), 8682-92.

33. Pandey, P. R.; Yang, J. H.; Tsitsipatis, D.; Panda, A. C.; Noh, J. H.; Kim, K. M.; Munk, R.; Nicholson, T.; Hanniford, D.; Argibay, D.; Yang, X.; Martindale, J. L.; Chang, M. W.; Jones, S. W.; Hernando, E.; Sen, P.; De, S.; Abdelmohsen, K.; Gorospe, M., circSamd4 represses myogenic transcriptional activity of PUR proteins. *Nucleic Acids Res* **2020,** *48* (7), 3789-3805.

34. Johnson, E. M., The Pur protein family: clues to function from recent studies on cancer and AIDS. *Anticancer Res* **2003,** *23* (3A), 2093-100.

35. Gallia, G. L.; Johnson, E. M.; Khalili, K., Puralpha: a multifunctional single-stranded DNA- and RNA-binding protein. *Nucleic Acids Res* **2000,** *28* (17), 3197-205.

36. Tretiakova, A.; Steplewski, A.; Johnson, E. M.; Khalili, K.; Amini, S., Regulation of myelin basic protein gene transcription by Sp1 and Puralpha: evidence for association of Sp1 and Puralpha in brain. *J Cell Physiol* **1999,** *181* (1), 160-8.

37. Du, Q.; Tomkinson, A. E.; Gardner, P. D., Transcriptional regulation of neuronal nicotinic acetylcholine receptor genes. A possible role for the DNA-binding protein Puralpha. *J Biol Chem* **1997,** *272* (23), 14990-5.

38. Haas, S.; Thatikunta, P.; Steplewski, A.; Johnson, E. M.; Khalili, K.; Amini, S., A 39-kD DNA-binding protein from mouse brain stimulates transcription of myelin basic protein gene in oligodendrocytic cells. *J Cell Biol* **1995,** *130* (5), 1171-9.

39. Kelm, R. J., Jr.; Elder, P. K.; Getz, M. J., The single-stranded DNA-binding proteins, Puralpha, Purbeta, and MSY1 specifically interact with an exon 3-derived mouse vascular smooth muscle alpha-actin messenger RNA sequence. *J Biol Chem* **1999,** *274* (53), 38268-75.

40. Gallia, G. L.; Darbinian, N.; Jaffe, N.; Khalili, K., Single-stranded nucleic acid-binding protein, Pur alpha, interacts with RNA homologous to 18S ribosomal RNA and inhibits translation in vitro. *J Cell Biochem* **2001,** *83* (3), 355-63.

41. Kanai, Y.; Dohmae, N.; Hirokawa, N., Kinesin transports RNA: isolation and characterization of an RNA-transporting granule. *Neuron* **2004,** *43* (4), 513-25.

42. Johnson, E. M.; Kinoshita, Y.; Weinreb, D. B.; Wortman, M. J.; Simon, R.; Khalili, K.; Winckler, B.; Gordon, J., Role of Pur alpha in targeting mRNA to sites of translation in hippocampal neuronal dendrites. *J Neurosci Res* **2006,** *83* (6), 929-43.

43. Shimotai, Y.; Minami, H.; Saitoh, Y.; Onodera, Y.; Mishima, Y.; Kelm, R. J., Jr.; Tsutsumi, K., A binding site for Pur alpha and Pur beta is structurally unstable and is required for replication in vivo from the rat aldolase B origin. *Biochem Biophys Res Commun* **2006,** *340* (2), 517-25.

44. Chang, C. F.; Gallia, G. L.; Muralidharan, V.; Chen, N. N.; Zoltick, P.; Johnson, E.; Khalili, K., Evidence that replication of human neurotropic JC virus DNA in glial cells is regulated by the sequence-specific single-stranded DNA-binding protein Pur alpha. *J Virol* **1996,** *70* (6), 4150-6.

45. Jurk, M.; Weissinger, F.; Lottspeich, F.; Schwarz, U.; Winnacker, E. L., Characterization of the single-strand-specific BPV-1 origin binding protein, SPSF I, as the HeLa Pur alpha factor. *Nucleic Acids Res* **1996,** *24* (14), 2799-806.

46. Itoh, H.; Wortman, M. J.; Kanovsky, M.; Uson, R. R.; Gordon, R. E.; Alfano, N.; Johnson, E. M., Alterations in Pur(alpha) levels and intracellular localization in the CV-1 cell cycle. *Cell Growth Differ* **1998,** *9* (8), 651-65.

47. Stacey, D. W.; Hitomi, M.; Kanovsky, M.; Gan, L.; Johnson, E. M., Cell cycle arrest and morphological alterations following microinjection of NIH3T3 cells with Pur alpha. *Oncogene* **1999,** *18* (29), 4254-61.

48. Barr, S. M.; Johnson, E. M., Ras-induced colony formation and anchorage-independent growth inhibited by elevated expression of Puralpha in NIH3T3 cells. *J Cell Biochem* **2001,** *81* (4), 621-38.

49. Liu, H.; Barr, S. M.; Chu, C.; Kohtz, D. S.; Kinoshita, Y.; Johnson, E. M., Functional interaction of Puralpha with the Cdk2 moiety of cyclin A/Cdk2. *Biochem Biophys Res Commun* **2005,** *328* (4), 851-7.

50. Khalili, K.; Del Valle, L.; Muralidharan, V.; Gault, W. J.; Darbinian, N.; Otte, J.; Meier, E.; Johnson, E. M.; Daniel, D. C.; Kinoshita, Y.; Amini, S.; Gordon, J., Puralpha is essential for postnatal brain development and developmentally coupled cellular proliferation as revealed by genetic inactivation in the mouse. *Mol Cell Biol* **2003,** *23* (19), 6857-75.

51. Lezon-Geyda, K.; Najfeld, V.; Johnson, E. M., Deletions of PURA, at 5q31, and PURB, at 7p13, in myelodysplastic syndrome and progression to acute myelogenous leukemia. *Leukemia* **2001,** *15* (6), 954-62.

52. Hariharan, S.; Kelm, R. J., Jr.; Strauch, A. R., The Puralpha/Purbeta single-strand DNA-binding proteins attenuate smooth-muscle actin gene transactivation in myofibroblasts. *J Cell Physiol* **2014,** *229* (9), 1256-71.

53. Rumora, A. E.; Wang, S. X.; Ferris, L. A.; Everse, S. J.; Kelm, R. J., Jr., Structural basis of multisite single-stranded DNA recognition and ACTA2 repression by purine-rich element binding protein B (Purbeta). *Biochemistry* **2013,** *52* (26), 4439-50.

54. Subramanian, S. V.; Kelm, R. J.; Polikandriotis, J. A.; Orosz, C. G.; Strauch, A. R., Reprogramming of vascular smooth muscle alpha-actin gene expression as an early indicator of dysfunctional remodeling following heart transplant. *Cardiovasc Res* **2002,** *54* (3), 539-48.

55. Zhao, S.; Kelm, R. J., Jr.; Fernald, R. D., Regulation of gonadotropin-releasing hormone-1 gene transcription by members of the purine-rich element-binding protein family. *Am J Physiol Endocrinol Metab* **2010,** *298* (3), E524-33.

56. Ohashi, S.; Kobayashi, S.; Omori, A.; Ohara, S.; Omae, A.; Muramatsu, T.; Li, Y.; Anzai, K., The single-stranded DNA- and RNA-binding proteins pur alpha and pur beta link BC1 RNA to microtubules through binding to the dendrite-targeting RNA motifs. *J Neurochem* **2000,** *75* (5), 1781-90.

57. Muslimov, I. A.; Iacoangeli, A.; Brosius, J.; Tiedge, H., Spatial codes in dendritic BC1 RNA. *J Cell Biol* **2006,** *175* (3), 427-39.

58. Ohashi, S.; Koike, K.; Omori, A.; Ichinose, S.; Ohara, S.; Kobayashi, S.; Sato, T. A.; Anzai, K., Identification of mRNA/protein (mRNP) complexes containing Puralpha, mStaufen, fragile X protein, and myosin Va and their association with rough endoplasmic reticulum equipped with a kinesin motor. *J Biol Chem* **2002,** *277* (40), 37804-10.

59. Cristofanilli, M.; Iacoangeli, A.; Muslimov, I. A.; Tiedge, H., Neuronal BC1 RNA: microtubule-dependent dendritic delivery. *J Mol Biol* **2006,** *356* (5), 1118-23.

60. Robeck, T.; Skryabin, B. V.; Rozhdestvensky, T. S.; Skryabin, A. B.; Brosius, J., BC1 RNA motifs required for dendritic transport in vivo. *Sci Rep* **2016,** *6*, 28300.

61. Nimmrich, V.; Hargreaves, E. L.; Muslimov, I. A.; Bianchi, R.; Tiedge, H., Dendritic BC1 RNA: modulation by kindling-induced afterdischarges. *Brain Res Mol Brain Res* **2005,** *133* (1), 110-8.

62. Wang, N.; Wang, R.; Wang, R.; Chen, S., Transcriptomics analysis revealing candidate networks and genes for the body size sexual dimorphism of Chinese tongue sole (Cynoglossus semilaevis). *Funct Integr Genomics* **2018,** *18* (3), 327-339.

63. Huo, N.; Yu, M.; Li, X.; Zhou, C.; Jin, X.; Gao, X., PURB is a positive regulator of amino acid-induced milk synthesis in bovine mammary epithelial cells. *J Cell Physiol* **2019,** *234* (5), 6992-7003.

64. Persson, H.; Kvist, A.; Rego, N.; Staaf, J.; Vallon-Christersson, J.; Luts, L.; Loman, N.; Jonsson, G.; Naya, H.; Hoglund, M.; Borg, A.; Rovira, C., Identification of new microRNAs in paired normal and tumor breast tissue suggests a dual role for the ERBB2/Her2 gene. *Cancer Res* **2011,** *71* (1), 78-86.

65. Radamas, R., Identification of modulators of chemotherapeutic resistance using a randon shRNA library. (Doctoral dissertation) **2012**. http://unsworks.unsw.edu.au/fapi/datastream/unsworks:10948/SOURCE01

66. Iannniello, F., Identification and characterization of antimicrobial agents to control bacterial infection. (Doctoral dissertation) **2018**. <http://www.fedoa.unina.it/11933/2/Ianniello_Flora_29_Parziale.pdf>

67. Cho, P., The vrole of microRNAs in the early life human innate immune response. (Doctoral dissertation) **2013**. <https://open.library.ubc.ca/cIRcle/collections/ubctheses/24/items/1.0073937>

68. Li, Y.; Yao, L.; Liu, F.; Hong, J.; Chen, L.; Zhang, B.; Zhang, W., Characterization of microRNA expression in serous ovarian carcinoma. *Int J Mol Med* **2014,** *34* (2), 491-8.

69. Glynn, C., microRNA-mediated intercellular communication in the primary breast tumour microenvironment. (Doctoral dissertation) **2014**. <https://aran.library.nuigalway.ie/xmlui/bitstream/handle/10379/4702/2014GlynnPhD.pdf?sequence=1>

70. Fujimori, T.; Kato, K.; Fujihara, S.; Iwama, H.; Yamashita, T.; Kobayashi, K.; Kamada, H.; Morishita, A.; Kobara, H.; Mori, H.; Okano, K.; Suzuki, Y.; Masaki, T., Antitumor effect of metformin on cholangiocarcinoma: In vitro and in vivo studies. *Oncol Rep* **2015,** *34* (6), 2987-96.

71. Li, N.; Lian, J.; Zhao, S.; Zheng, D.; Yang, X.; Huang, X.; Shi, X.; Sun, L.; Zhou, Q.; Shi, H.; Xu, G.; Incoom, E. K.; Zhou, J.; Shao, G., Detection of Differentially Expressed MicroRNAs in Rheumatic Heart Disease: miR-1183 and miR-1299 as Potential Diagnostic Biomarkers. *Biomed Res Int* **2015,** *2015*, 524519.

72. Raaby, L.; Langkilde, A.; Kjellerup, R. B.; Vinter, H.; Khatib, S. H.; Hjuler, K. F.; Johansen, C.; Iversen, L., Changes in mRNA expression precede changes in microRNA expression in lesional psoriatic skin during treatment with adalimumab. *Br J Dermatol* **2015,** *173* (2), 436-47.

73. Gardiner, A. S.; Gutierrez, H. L.; Luo, L.; Davies, S.; Savage, D. D.; Bakhireva, L. N.; Perrone-Bizzozero, N. I., Alcohol Use During Pregnancy is Associated with Specific Alterations in MicroRNA Levels in Maternal Serum. *Alcohol Clin Exp Res* **2016,** *40* (4), 826-37.

74. Shango, J.G., Modulation of oral immunity by viral microRNA: impact on macrophage & epithelial cell microRNA expression. (Doctoral dissertation) **2016**. https://indigo.uic.edu/articles/Modulation_of_Oral_Immunity_by_Viral_MicroRNA_Impact_on_Macrophage_Epithelial_Cell_MicroRNA_Expression/10793891/files/19305953.pdf

75. Slattery, M. L.; Herrick, J. S.; Pellatt, D. F.; Mullany, L. E.; Stevens, J. R.; Wolff, E.; Hoffman, M. D.; Wolff, R. K.; Samowitz, W., Site-specific associations between miRNA expression and survival in colorectal cancer cases. *Oncotarget* **2016,** *7* (37), 60193-60205.

76. Jiang, Y.; Gao, H.; Liu, M.; Mao, Q., Sorting and biological characteristics analysis for side population cells in human primary hepatocellular carcinoma. *Am J Cancer Res* **2016,** *6* (9), 1890-1905.

77. Ohzawa, H.; Miki, A.; Teratani, T.; Shiba, S.; Sakuma, Y.; Nishimura, W.; Noda, Y.; Fukushima, N.; Fujii, H.; Hozumi, Y.; Mukai, H.; Yasuda, Y., Usefulness of miRNA profiles for predicting pathological responses to neoadjuvant chemotherapy in patients with human epidermal growth factor receptor 2-positive breast cancer. *Oncol Lett* **2017,** *13* (3), 1731-1740.

78. Zhang, B.; Guo, W.; Sun, C.; Duan, H. Q.; Yu, B. B.; Mu, K.; Guan, Y. Y.; Li, Y.; Liu, S.; Liu, Y.; Ban, D. X.; Ruan, W. D.; Kong, X. H.; Xing, C.; Ning, G. Z.; Feng, S. Q., Dysregulated MiR-3150a-3p Promotes Lumbar Intervertebral Disc Degeneration by Targeting Aggrecan. *Cell Physiol Biochem* **2018,** *45* (6), 2506-2515.

79. Anandaram, H., A computational approach to identify microRNA (miRNA) based biomarker of Pharmacovariant from the regulation of disease pathology. *MOJ Proteomics & Bioinformatics* **2018**, 7 (3**)** 152-170.

80. Klinge, C. M.; Piell, K. M.; Tooley, C. S.; Rouchka, E. C., HNRNPA2/B1 is upregulated in endocrine-resistant LCC9 breast cancer cells and alters the miRNA transcriptome when overexpressed in MCF-7 cells. *Sci Rep* **2019,** *9* (1), 9430.

81. Zhao, X.; Jia, Y.; Chen, H.; Yao, H.; Guo, W., Plasma-derived exosomal miR-183 associates with protein kinase activity and may serve as a novel predictive biomarker of myocardial ischemic injury. *Exp Ther Med* **2019,** *18* (1), 179-187.

82. Morenikeji, O. B.; Hawkes, M. E.; Hudson, A. O.; Thomas, B. N., Computational Network Analysis Identifies Evolutionarily Conserved miRNA Gene Interactions Potentially Regulating Immune Response in Bovine Trypanosomosis. *Front Microbiol* **2019,** *10*, 2010.

83. Woods, P. S.; Doolittle, L. M.; Rosas, L. E.; Nana-Sinkam, S. P.; Tili, E.; Davis, I. C., Increased expression of microRNA-155-5p by alveolar type II cells contributes to development of lethal ARDS in H1N1 influenza A virus-infected mice. *Virology* **2020,** *545*, 40-52.

84. Chen, P. H.; Tseng, W. H.; Chi, J. T., The Intersection of DNA Damage Response and Ferroptosis-A Rationale for Combination Therapeutics. *Biology (Basel)* **2020,** *9* (8).

85. Wang, C. Q. F.; Akalu, Y. T.; Suarez-Farinas, M.; Gonzalez, J.; Mitsui, H.; Lowes, M. A.; Orlow, S. J.; Manga, P.; Krueger, J. G., IL-17 and TNF synergistically modulate cytokine expression while suppressing melanogenesis: potential relevance to psoriasis. *J Invest Dermatol* **2013,** *133* (12), 2741-2752.

86. Taha, M.A.; Ahmed, S.; Elnair, R.; Basher, E.; Abdelghani, A.; Mohamed Ali, A.; Dafaalla, M.D.; Alfaki, M.M.; Abdelrahim, M.A.; Abdalla, A.A.; Mohammed, M.I.; Elsheikh, M.; Hussein, A.; Hassan, M., In silico analysis of non-synonymous Single nucleotide polymorphisms of BMBR2 (PPH1) gene and demonstration of gene’s network interactions. *International Journal of Computational Bioinformatics and In Silico Modeling* **2015**, 4(5) 724-734.

87. Gramann, A. K.; Venkatesan, A. M.; Guerin, M.; Ceol, C. J., Regulation of zebrafish melanocyte development by ligand-dependent BMP signaling. *Elife* **2019,** *8*.

88. Yang, J.; Wang, J.; Pan, L.; Li, H.; Rao, C.; Zhang, X.; Niu, G.; Qu, J.; Hou, L., BMP4 is required for the initial expression of MITF in melanocyte precursor differentiation from embryonic stem cells. *Exp Cell Res* **2014,** *320* (1), 54-61.

89. Singh, S. K.; Abbas, W. A.; Tobin, D. J., Bone morphogenetic proteins differentially regulate pigmentation in human skin cells. *J Cell Sci* **2012,** *125* (Pt 18), 4306-19.

90. Park, H. Y.; Wu, C.; Yaar, M.; Stachur, C. M.; Kosmadaki, M.; Gilchrest, B. A., Role of BMP-4 and Its Signaling Pathways in Cultured Human Melanocytes. *Int J Cell Biol* **2009,** *2009*, 750482.

91. Yaar, M.; Wu, C.; Park, H. Y.; Panova, I.; Schutz, G.; Gilchrest, B. A., Bone morphogenetic protein-4, a novel modulator of melanogenesis. *J Biol Chem* **2006,** *281* (35), 25307-14.

92. Bilodeau, M. L.; Greulich, J. D.; Hullinger, R. L.; Bertolotto, C.; Ballotti, R.; Andrisani, O. M., BMP-2 stimulates tyrosinase gene expression and melanogenesis in differentiated melanocytes. *Pigment Cell Res* **2001,** *14* (5), 328-36.

93. Sinnberg, T.; Niessner, H.; Levesque, M. P.; Dettweiler, C.; Garbe, C.; Busch, C., Embryonic bone morphogenetic protein and nodal induce invasion in melanocytes and melanoma cells. *Biol Open* **2018,** *7* (6).

94. Venkatesan, A. M.; Vyas, R.; Gramann, A. K.; Dresser, K.; Gujja, S.; Bhatnagar, S.; Chhangawala, S.; Gomes, C. B. F.; Xi, H. S.; Lian, C. G.; Houvras, Y.; Edwards, Y. J. K.; Deng, A.; Green, M.; Ceol, C. J., Ligand-activated BMP signaling inhibits cell differentiation and death to promote melanoma. *J Clin Invest* **2018,** *128* (1), 294-308.

95. Braig, S.; Bosserhoff, A. K., Death inducer-obliterator 1 (Dido1) is a BMP target gene and promotes BMP-induced melanoma progression. *Oncogene* **2013,** *32* (7), 837-48.

96. Hsu, M. Y.; Rovinsky, S. A.; Lai, C. Y.; Qasem, S.; Liu, X.; How, J.; Engelhardt, J. F.; Murphy, G. F., Aggressive melanoma cells escape from BMP7-mediated autocrine growth inhibition through coordinated Noggin upregulation. *Lab Invest* **2008,** *88* (8), 842-55.

97. Rothhammer, T.; Poser, I.; Soncin, F.; Bataille, F.; Moser, M.; Bosserhoff, A. K., Bone morphogenic proteins are overexpressed in malignant melanoma and promote cell invasion and migration. *Cancer Res* **2005,** *65* (2), 448-56.

98. Di Costanzo, L.; Scala, E.; Caiazzo, G.; Lembo, S.; Marino, R.; Megna, M.; Patri, A.; Di Caprio, R.; Balato, A., Possible role of BMP-4 in the hyper-pigmentation of psoriatic plaques after anti-TNF-alpha treatment. *Exp Ther Med* **2019,** *18* (5), 4120-4124.

99. Han, R.; Beppu, H.; Lee, Y. K.; Georgopoulos, K.; Larue, L.; Li, E.; Weiner, L.; Brissette, J. L., A pair of transmembrane receptors essential for the retention and pigmentation of hair. *Genesis* **2012,** *50* (11), 783-800.

100. Shieh, B. H.; Stamnes, M. A.; Seavello, S.; Harris, G. L.; Zuker, C. S., The ninaA gene required for visual transduction in Drosophila encodes a homologue of cyclosporin A-binding protein. *Nature* **1989,** *338* (6210), 67-70.

101. Freskgard, P. O.; Bergenhem, N.; Jonsson, B. H.; Svensson, M.; Carlsson, U., Isomerase and chaperone activity of prolyl isomerase in the folding of carbonic anhydrase. *Science* **1992,** *258* (5081), 466-8.

102. Nigro, P.; Pompilio, G.; Capogrossi, M. C., Cyclophilin A: a key player for human disease. *Cell Death Dis* **2013,** *4*, e888.

103. Caputo, E.; Maiorana, L.; Vasta, V.; Pezzino, F. M.; Sunkara, S.; Wynne, K.; Elia, G.; Marincola, F. M.; McCubrey, J. A.; Libra, M.; Travali, S.; Kane, M., Characterization of human melanoma cell lines and melanocytes by proteome analysis. *Cell Cycle* **2011,** *10* (17), 2924-36.

104. Zhang, J.; Song, M. Q.; Zhu, J. S.; Zhou, Z.; Xu, Z. P.; Chen, W. X.; Chen, N. W., Identification of differentially-expressed proteins between early submucosal non-invasive and invasive colorectal cancer using 2D-DIGE and mass spectrometry. *Int J Immunopathol Pharmacol* **2011,** *24* (4), 849-59.

105. Yamamoto, T.; Takakura, H.; Mitamura, K.; Taga, A., Cyclophilin a knokdown inhibits cell migration and invasion through the suppression of epithelial-mesenchymal transition in colorectal cancer cells. *Biochem Biophys Res Commun* **2020,** *526* (1), 55-61.

106. Guo, M.; James, A. W.; Kwak, J. H.; Shen, J.; Yokoyama, K. K.; Ting, K.; Soo, C. B.; Chiu, R. H., Cyclophilin A (CypA) Plays Dual Roles in Regulation of Bone Anabolism and Resorption. *Sci Rep* **2016,** *6*, 22378.

107. Cheng, S.; Luo, M.; Ding, C.; Peng, C.; Lv, Z.; Tong, R.; Xiao, H.; Xie, H.; Zhou, L.; Wu, J.; Zheng, S., Downregulation of Peptidylprolyl isomerase A promotes cell death and enhances doxorubicin-induced apoptosis in hepatocellular carcinoma. *Gene* **2016,** *591* (1), 236-244.

108. Massignan, T.; Casoni, F.; Basso, M.; Stefanazzi, P.; Biasini, E.; Tortarolo, M.; Salmona, M.; Gianazza, E.; Bendotti, C.; Bonetto, V., Proteomic analysis of spinal cord of presymptomatic amyotrophic lateral sclerosis G93A SOD1 mouse. *Biochem Biophys Res Commun* **2007,** *353* (3), 719-25.

109. Nardo, G.; Pozzi, S.; Pignataro, M.; Lauranzano, E.; Spano, G.; Garbelli, S.; Mantovani, S.; Marinou, K.; Papetti, L.; Monteforte, M.; Torri, V.; Paris, L.; Bazzoni, G.; Lunetta, C.; Corbo, M.; Mora, G.; Bendotti, C.; Bonetto, V., Amyotrophic lateral sclerosis multiprotein biomarkers in peripheral blood mononuclear cells. *PLoS One* **2011,** *6* (10), e25545.

110. Marino, M.; Papa, S.; Crippa, V.; Nardo, G.; Peviani, M.; Cheroni, C.; Trolese, M. C.; Lauranzano, E.; Bonetto, V.; Poletti, A.; DeBiasi, S.; Ferraiuolo, L.; Shaw, P. J.; Bendotti, C., Differences in protein quality control correlate with phenotype variability in 2 mouse models of familial amyotrophic lateral sclerosis. *Neurobiol Aging* **2015,** *36* (1), 492-504.

111. Bendotti, C.; Marino, M.; Cheroni, C.; Fontana, E.; Crippa, V.; Poletti, A.; De Biasi, S., Dysfunction of constitutive and inducible ubiquitin-proteasome system in amyotrophic lateral sclerosis: implication for protein aggregation and immune response. *Prog Neurobiol* **2012,** *97* (2), 101-26.

112. Chen, S.; Zhang, X.; Song, L.; Le, W., Autophagy dysregulation in amyotrophic lateral sclerosis. *Brain Pathol* **2012,** *22* (1), 110-6.

113. Buratti, E.; Baralle, F. E., The multiple roles of TDP-43 in pre-mRNA processing and gene expression regulation. *RNA Biol* **2010,** *7* (4), 420-9.

114. Lauranzano, E.; Pozzi, S.; Pasetto, L.; Stucchi, R.; Massignan, T.; Paolella, K.; Mombrini, M.; Nardo, G.; Lunetta, C.; Corbo, M.; Mora, G.; Bendotti, C.; Bonetto, V., Peptidylprolyl isomerase A governs TARDBP function and assembly in heterogeneous nuclear ribonucleoprotein complexes. *Brain* **2015,** *138* (Pt 4), 974-91.

115. Otake, K.; Kamiguchi, H.; Hirozane, Y., Identification of biomarkers for amyotrophic lateral sclerosis by comprehensive analysis of exosomal mRNAs in human cerebrospinal fluid. *BMC Med Genomics* **2019,** *12* (1), 7.

116. Li, W.; Wang, W.; Li, Y.; Wang, W.; Wang, T.; Li, L.; Han, Z.; Wang, S.; Ma, D.; Wang, H., Proteomics analysis of normal and senescent NG108-15 cells: GRP78 plays a negative role in cisplatin-induced senescence in the NG108-15 cell line. *PLoS One* **2014,** *9* (3), e90114.

117. Yu, T.; Lin, S.; Xu, R.; Du, T. X.; Li, Y.; Gao, H.; Diao, H. L.; Zhang, X. H., Cyclophilin A plays an important role in embryo implantation through activating Stat3. *Reproduction* **2020,** *160* (3), 343-351.

118. Kim, J. Y.; Kim, W. J.; Kim, H.; Suk, K.; Lee, W. H., The Stimulation of CD147 Induces MMP-9 Expression through ERK and NF-kappaB in Macrophages: Implication for Atherosclerosis. *Immune Netw* **2009,** *9* (3), 90-7.

119. Bahmed, K.; Henry, C.; Holliday, M.; Redzic, J.; Ciobanu, M.; Zhang, F.; Weekes, C.; Sclafani, R.; Degregori, J.; Eisenmesser, E., Extracellular cyclophilin-A stimulates ERK1/2 phosphorylation in a cell-dependent manner but broadly stimulates nuclear factor kappa B. *Cancer Cell Int* **2012,** *12* (1), 19.

120. Sun, S.; Guo, M.; Zhang, J. B.; Ha, A.; Yokoyama, K. K.; Chiu, R. H., Cyclophilin A (CypA) interacts with NF-kappaB subunit, p65/RelA, and contributes to NF-kappaB activation signaling. *PLoS One* **2014,** *9* (8), e96211.

121. Pasetto, L.; Pozzi, S.; Castelnovo, M.; Basso, M.; Estevez, A. G.; Fumagalli, S.; De Simoni, M. G.; Castellaneta, V.; Bigini, P.; Restelli, E.; Chiesa, R.; Trojsi, F.; Monsurro, M. R.; Callea, L.; Malesevic, M.; Fischer, G.; Freschi, M.; Tortarolo, M.; Bendotti, C.; Bonetto, V., Targeting Extracellular Cyclophilin A Reduces Neuroinflammation and Extends Survival in a Mouse Model of Amyotrophic Lateral Sclerosis. *J Neurosci* **2017,** *37* (6), 1413-1427.

122. Squarize, C. H.; Castilho, R. M.; Sriuranpong, V.; Pinto, D. S., Jr.; Gutkind, J. S., Molecular cross-talk between the NFkappaB and STAT3 signaling pathways in head and neck squamous cell carcinoma. *Neoplasia* **2006,** *8* (9), 733-46.

123. Grivennikov, S. I.; Karin, M., Dangerous liaisons: STAT3 and NF-kappaB collaboration and crosstalk in cancer. *Cytokine Growth Factor Rev* **2010,** *21* (1), 11-9.

124. Olavarria, V. H.; Sepulcre, M. P.; Figueroa, J. E.; Mulero, V., Prolactin-induced production of reactive oxygen species and IL-1beta in leukocytes from the bony fish gilthead seabream involves Jak/Stat and NF-kappaB signaling pathways. *J Immunol* **2010,** *185* (7), 3873-83.

125. McFarland, B. C.; Gray, G. K.; Nozell, S. E.; Hong, S. W.; Benveniste, E. N., Activation of the NF-kappaB pathway by the STAT3 inhibitor JSI-124 in human glioblastoma cells. *Mol Cancer Res* **2013,** *11* (5), 494-505.

126. Ahmad, S. F.; Ansari, M. A.; Zoheir, K. M.; Bakheet, S. A.; Korashy, H. M.; Nadeem, A.; Ashour, A. E.; Attia, S. M., Regulation of TNF-alpha and NF-kappaB activation through the JAK/STAT signaling pathway downstream of histamine 4 receptor in a rat model of LPS-induced joint inflammation. *Immunobiology* **2015,** *220* (7), 889-98.

127. Czerkies, M.; Korwek, Z.; Prus, W.; Kochanczyk, M.; Jaruszewicz-Blonska, J.; Tudelska, K.; Blonski, S.; Kimmel, M.; Brasier, A. R.; Lipniacki, T., Cell fate in antiviral response arises in the crosstalk of IRF, NF-kappaB and JAK/STAT pathways. *Nat Commun* **2018,** *9* (1), 493.

128. Haselager, M.; Thijssen, R.; Kater, A. P.; Eldering, E., Cross-Talk between cytokine and NF-kb signaling in the CLL microenvironment can affect sensitivity for Venetoclax. *Blood* **2019,** 134 (suppl.1) 5449.

129. Wang, S.; Zhou, M.; Lin, F.; Liu, D.; Hong, W.; Lu, L.; Zhu, Y.; Xu, A., Interferon-gamma induces senescence in normal human melanocytes. *PLoS One* **2014,** *9* (3), e93232.

130. Gordon-Thomson, C.; Mason, R. S.; Moore, G. P., Regulation of epidermal growth factor receptor expression in human melanocytes. *Exp Dermatol* **2001,** *10* (5), 321-8.

131. Mirmohammadsadegh, A.; Hassan, M.; Gustrau, A.; Doroudi, R.; Schmittner, N.; Nambiar, S.; Tannapfel, A.; Ruzicka, T.; Hengge, U. R., Constitutive expression of epidermal growth factor receptors on normal human melanocytes. *J Invest Dermatol* **2005,** *125* (2), 392-4.

132. Szabad, G.; Kormos, B.; Pivarcsi, A.; Szell, M.; Kis, K.; Kenderessy Szabo, A.; Dobozy, A.; Kemeny, L.; Bata-Csorgo, Z., Human adult epidermal melanocytes cultured without chemical mitogens express the EGF receptor and respond to EGF. *Arch Dermatol Res* **2007,** *299* (4), 191-200.

133. Kormos, B.; Belso, N.; Bebes, A.; Szabad, G.; Bacsa, S.; Szell, M.; Kemeny, L.; Bata-Csorgo, Z., In vitro dedifferentiation of melanocytes from adult epidermis. *PLoS One* **2011,** *6* (2), e17197.

134. Zhang, K.; Anumanthan, G.; Scheaffer, S.; Cornelius, L. A., HMGB1/RAGE Mediates UVB-Induced Secretory Inflammatory Response and Resistance to Apoptosis in Human Melanocytes. *J Invest Dermatol* **2019,** *139* (1), 202-212.

135. Wong, L. H.; Krauer, K. G.; Hatzinisiriou, I.; Estcourt, M. J.; Hersey, P.; Tam, N. D.; Edmondson, S.; Devenish, R. J.; Ralph, S. J., Interferon-resistant human melanoma cells are deficient in ISGF3 components, STAT1, STAT2, and p48-ISGF3gamma. *J Biol Chem* **1997,** *272* (45), 28779-85.

136. Beliakoff, J.; Sun, Z., Zimp7 and Zimp10, two novel PIAS-like proteins, function as androgen receptor coregulators. *Nucl Recept Signal* **2006,** *4*, e017.

137. Peng, Y.; Lee, J.; Zhu, C.; Sun, Z., A novel role for protein inhibitor of activated STAT (PIAS) proteins in modulating the activity of Zimp7, a novel PIAS-like protein, in androgen receptor-mediated transcription. *J Biol Chem* **2010,** *285* (15), 11465-75.

138. Rodriguez-Magadan, H.; Ramirez, L.; Schnabel, D.; Vazquez, M.; Lomeli, H., Sexually dimorphic gene expression of the Zimp7 and Zimp10 genes in embryonic gonads. *Gene Expr Patterns* **2010,** *10* (1), 16-23.

139. Tuccilli, C.; Baldini, E.; Sorrenti, S.; Di Gioia, C.; Bosco, D.; Ascoli, V.; Mian, C.; Barollo, S.; Rendina, R.; Coccaro, C.; Pepe, M.; Catania, A.; Bononi, M.; Tartaglia, F.; De Antoni, E.; D'Armiento, M.; Ulisse, S., Papillary Thyroid Cancer Is Characterized by Altered Expression of Genes Involved in the Sumoylation Process. *J Biol Regul Homeost Agents* **2015,** *29* (3), 655-62.

140. Taylor, K. M.; Labonne, C., SoxE factors function equivalently during neural crest and inner ear development and their activity is regulated by SUMOylation. *Dev Cell* **2005,** *9* (5), 593-603.

141. Harris, M. L.; Baxter, L. L.; Loftus, S. K.; Pavan, W. J., Sox proteins in melanocyte development and melanoma. *Pigment Cell Melanoma Res* **2010,** *23* (4), 496-513.

142. Kondoh, H.; Kamachi, Y., SOX-partner code for cell specification: Regulatory target selection and underlying molecular mechanisms. *Int J Biochem Cell Biol* **2010,** *42* (3), 391-9.

143. Adameyko, I.; Lallemend, F.; Furlan, A.; Zinin, N.; Aranda, S.; Kitambi, S. S.; Blanchart, A.; Favaro, R.; Nicolis, S.; Lubke, M.; Muller, T.; Birchmeier, C.; Suter, U.; Zaitoun, I.; Takahashi, Y.; Ernfors, P., Sox2 and Mitf cross-regulatory interactions consolidate progenitor and melanocyte lineages in the cranial neural crest. *Development* **2012,** *139* (2), 397-410.

144. Sharma, K.; Arora, A., Waardenburg Syndrome: A Case Study of Two Patients. *Indian J Otolaryngol Head Neck Surg* **2015,** *67* (3), 324-8.

145. Bertolotto, C.; Lesueur, F.; Giuliano, S.; Strub, T.; de Lichy, M.; Bille, K.; Dessen, P.; d'Hayer, B.; Mohamdi, H.; Remenieras, A.; Maubec, E.; de la Fouchardiere, A.; Molinie, V.; Vabres, P.; Dalle, S.; Poulalhon, N.; Martin-Denavit, T.; Thomas, L.; Andry-Benzaquen, P.; Dupin, N.; Boitier, F.; Rossi, A.; Perrot, J. L.; Labeille, B.; Robert, C.; Escudier, B.; Caron, O.; Brugieres, L.; Saule, S.; Gardie, B.; Gad, S.; Richard, S.; Couturier, J.; Teh, B. T.; Ghiorzo, P.; Pastorino, L.; Puig, S.; Badenas, C.; Olsson, H.; Ingvar, C.; Rouleau, E.; Lidereau, R.; Bahadoran, P.; Vielh, P.; Corda, E.; Blanche, H.; Zelenika, D.; Galan, P.; French Familial Melanoma Study, G.; Aubin, F.; Bachollet, B.; Becuwe, C.; Berthet, P.; Bignon, Y. J.; Bonadona, V.; Bonafe, J. L.; Bonnet-Dupeyron, M. N.; Cambazard, F.; Chevrant-Breton, J.; Coupier, I.; Dalac, S.; Demange, L.; d'Incan, M.; Dugast, C.; Faivre, L.; Vincent-Fetita, L.; Gauthier-Villars, M.; Gilbert, B.; Grange, F.; Grob, J. J.; Humbert, P.; Janin, N.; Joly, P.; Kerob, D.; Lasset, C.; Leroux, D.; Levang, J.; Limacher, J. M.; Livideanu, C.; Longy, M.; Lortholary, A.; Stoppa-Lyonnet, D.; Mansard, S.; Mansuy, L.; Marrou, K.; Mateus, C.; Maugard, C.; Meyer, N.; Nogues, C.; Souteyrand, P.; Venat-Bouvet, L.; Zattara, H.; Chaudru, V.; Lenoir, G. M.; Lathrop, M.; Davidson, I.; Avril, M. F.; Demenais, F.; Ballotti, R.; Bressac-de Paillerets, B., A SUMOylation-defective MITF germline mutation predisposes to melanoma and renal carcinoma. *Nature* **2011,** *480* (7375), 94-8.

146. Bonet, C.; Luciani, F.; Ottavi, J. F.; Leclerc, J.; Jouenne, F. M.; Boncompagni, M.; Bille, K.; Hofman, V.; Bossis, G.; Marco de Donatis, G.; Strub, T.; Cheli, Y.; Ohanna, M.; Luciano, F.; Marchetti, S.; Rocchi, S.; Birling, M. C.; Avril, M. F.; Poulalhon, N.; Luc, T.; Hofman, P.; Lacour, J. P.; Davidson, I.; Bressac-de Paillerets, B.; Ballotti, R.; Marine, J. C.; Bertolotto, C., Deciphering the Role of Oncogenic MITFE318K in Senescence Delay and Melanoma Progression. *J Natl Cancer Inst* **2017,** *109* (8).

147. Pinnell, N.; Yan, R.; Cho, H. J.; Keeley, T.; Murai, M. J.; Liu, Y.; Alarcon, A. S.; Qin, J.; Wang, Q.; Kuick, R.; Elenitoba-Johnson, K. S.; Maillard, I.; Samuelson, L. C.; Cierpicki, T.; Chiang, M. Y., The PIAS-like Coactivator Zmiz1 Is a Direct and Selective Cofactor of Notch1 in T Cell Development and Leukemia. *Immunity* **2015,** *43* (5), 870-83.

148. Rakowski, L. A.; Garagiola, D. D.; Li, C. M.; Decker, M.; Caruso, S.; Jones, M.; Kuick, R.; Cierpicki, T.; Maillard, I.; Chiang, M. Y., Convergence of the ZMIZ1 and NOTCH1 pathways at C-MYC in acute T lymphoblastic leukemias. *Cancer Res* **2013,** *73* (2), 930-41.

149. Moreno-Ayala, R.; Schnabel, D.; Salas-Vidal, E.; Lomeli, H., PIAS-like protein Zimp7 is required for the restriction of the zebrafish organizer and mesoderm development. *Dev Biol* **2015,** *403* (1), 89-100.

150. York, J. R.; McCauley, D. W., The origin and evolution of vertebrate neural crest cells. *Open Biol* **2020,** *10* (1), 190285.

151. Nornes, S.; Newman, M.; Wells, S.; Verdile, G.; Martins, R. N.; Lardelli, M., Independent and cooperative action of Psen2 with Psen1 in zebrafish embryos. *Exp Cell Res* **2009,** *315* (16), 2791-801.

152. Massi, D.; Tarantini, F.; Franchi, A.; Paglierani, M.; Di Serio, C.; Pellerito, S.; Leoncini, G.; Cirino, G.; Geppetti, P.; Santucci, M., Evidence for differential expression of Notch receptors and their ligands in melanocytic nevi and cutaneous malignant melanoma. *Mod Pathol* **2006,** *19* (2), 246-54.

153. Schouwey, K.; Delmas, V.; Larue, L.; Zimber-Strobl, U.; Strobl, L. J.; Radtke, F.; Beermann, F., Notch1 and Notch2 receptors influence progressive hair graying in a dose-dependent manner. *Dev Dyn* **2007,** *236* (1), 282-9.

154. Zhu, Y.; Gu, L.; Lin, X.; Cui, K.; Liu, C.; Lu, B.; Zhou, F.; Zhao, Q.; Shen, H.; Li, Y., LINC00265 promotes colorectal tumorigenesis via ZMIZ2 and USP7-mediated stabilization of beta-catenin. *Cell Death Differ* **2020,** *27* (4), 1316-1327.

155. Lee, S. H.; Zhu, C.; Peng, Y.; Johnson, D. T.; Lehmann, L.; Sun, Z., Identification of a novel role of ZMIZ2 protein in regulating the activity of the Wnt/beta-catenin signaling pathway. *J Biol Chem* **2013,** *288* (50), 35913-24.

156. Lee, J.; Beliakoff, J.; Sun, Z., The novel PIAS-like protein hZimp10 is a transcriptional co-activator of the p53 tumor suppressor. *Nucleic Acids Res* **2007,** *35* (13), 4523-34.

157. Cui, R.; Widlund, H. R.; Feige, E.; Lin, J. Y.; Wilensky, D. L.; Igras, V. E.; D'Orazio, J.; Fung, C. Y.; Schanbacher, C. F.; Granter, S. R.; Fisher, D. E., Central role of p53 in the suntan response and pathologic hyperpigmentation. *Cell* **2007,** *128* (5), 853-64.

158. Box, N. F.; Terzian, T., The role of p53 in pigmentation, tanning and melanoma. *Pigment Cell Melanoma Res* **2008,** *21* (5), 525-33.

159. Murase, D.; Hachiya, A.; Amano, Y.; Ohuchi, A.; Kitahara, T.; Takema, Y., The essential role of p53 in hyperpigmentation of the skin via regulation of paracrine melanogenic cytokine receptor signaling. *J Biol Chem* **2009,** *284* (7), 4343-53.

160. Hyter, S.; Coleman, D. J.; Ganguli-Indra, G.; Merrill, G. F.; Ma, S.; Yanagisawa, M.; Indra, A. K., Endothelin-1 is a transcriptional target of p53 in epidermal keratinocytes and regulates ultraviolet-induced melanocyte homeostasis. *Pigment Cell Melanoma Res* **2013,** *26* (2), 247-58.

161. Chang, C. H.; Kuo, C. J.; Ito, T.; Su, Y. Y.; Jiang, S. T.; Chiu, M. H.; Lin, Y. H.; Nist, A.; Mernberger, M.; Stiewe, T.; Ito, S.; Wakamatsu, K.; Hsueh, Y. A.; Shieh, S. Y.; Snir-Alkalay, I.; Ben-Neriah, Y., CK1alpha ablation in keratinocytes induces p53-dependent, sunburn-protective skin hyperpigmentation. *Proc Natl Acad Sci U S A* **2017,** *114* (38), E8035-E8044.

162. Li, X.; Thyssen, G.; Beliakoff, J.; Sun, Z., The novel PIAS-like protein hZimp10 enhances Smad transcriptional activity. *J Biol Chem* **2006,** *281* (33), 23748-56.

163. Yang, G.; Li, Y.; Nishimura, E. K.; Xin, H.; Zhou, A.; Guo, Y.; Dong, L.; Denning, M. F.; Nickoloff, B. J.; Cui, R., Inhibition of PAX3 by TGF-beta modulates melanocyte viability. *Mol Cell* **2008,** *32* (4), 554-63.

164. Mailloux, R. J.; Craig Ayre, D.; Christian, S. L., Induction of mitochondrial reactive oxygen species production by GSH mediated S-glutathionylation of 2-oxoglutarate dehydrogenase. *Redox Biol* **2016,** *8*, 285-97.

165. Bunik, V. I.; Kabysheva, M. S.; Klimuk, E. I.; Storozhevykh, T. P.; Pinelis, V. G., Phosphono analogues of 2-oxoglutarate protect cerebellar granule neurons upon glutamate excitotoxicity. *Ann N Y Acad Sci* **2009,** *1171*, 521-9.

166. Gibson, G. E.; Blass, J. P.; Beal, M. F.; Bunik, V., The alpha-ketoglutarate-dehydrogenase complex: a mediator between mitochondria and oxidative stress in neurodegeneration. *Mol Neurobiol* **2005,** *31* (1-3), 43-63.

167. Amsterdam, A.; Nissen, R. M.; Sun, Z.; Swindell, E. C.; Farrington, S.; Hopkins, N., Identification of 315 genes essential for early zebrafish development. *Proc Natl Acad Sci U S A* **2004,** *101* (35), 12792-7.

168. Dunckelmann, R. J.; Ebinger, F.; Schulze, A.; Wanders, R. J.; Rating, D.; Mayatepek, E., 2-ketoglutarate dehydrogenase deficiency with intermittent 2-ketoglutaric aciduria. *Neuropediatrics* **2000,** *31* (1), 35-8.

169. Scaduto, R. C., Jr., Calcium and 2-oxoglutarate-mediated control of aspartate formation by rat heart mitochondria. *Eur J Biochem* **1994,** *223* (3), 751-8.

170. O'Donnell, J. M.; Doumen, C.; LaNoue, K. F.; White, L. T.; Yu, X.; Alpert, N. M.; Lewandowski, E. D., Dehydrogenase regulation of metabolite oxidation and efflux from mitochondria in intact hearts. *Am J Physiol* **1998,** *274* (2), H467-76.

171. Rubi, B.; del Arco, A.; Bartley, C.; Satrustegui, J.; Maechler, P., The malate-aspartate NADH shuttle member Aralar1 determines glucose metabolic fate, mitochondrial activity, and insulin secretion in beta cells. *J Biol Chem* **2004,** *279* (53), 55659-66.

172. Pardo, B.; Contreras, L.; Serrano, A.; Ramos, M.; Kobayashi, K.; Iijima, M.; Saheki, T.; Satrustegui, J., Essential role of aralar in the transduction of small Ca2+ signals to neuronal mitochondria. *J Biol Chem* **2006,** *281* (2), 1039-47.

173. MacKenzie, E. D.; Selak, M. A.; Tennant, D. A.; Payne, L. J.; Crosby, S.; Frederiksen, C. M.; Watson, D. G.; Gottlieb, E., Cell-permeating alpha-ketoglutarate derivatives alleviate pseudohypoxia in succinate dehydrogenase-deficient cells. *Mol Cell Biol* **2007,** *27* (9), 3282-9.

174. Sharma, A.; Kumar, V., Metabolic plasticity mediates differential responses to spring and autumn migrations: Evidence from gene expression patterns in migratory buntings. *Exp Physiol* **2019,** *104* (12), 1841-1857.

175. Duan, L.; Perez, R. E.; Maki, C. G., Alpha ketoglutarate levels, regulated by p53 and OGDH, determine autophagy and cell fate/apoptosis in response to Nutlin-3a. *Cancer Biol Ther* **2019,** *20* (3), 252-260.

176. Zhang, J.; Liu, F.; Cao, J.; Liu, X., Skin transcriptome profiles associated with skin color in chickens. *PLoS One* **2015,** *10* (6), e0127301.

177. Parodi, C.; Hardman, J. A.; Allavena, G.; Marotta, R.; Catelani, T.; Bertolini, M.; Paus, R.; Grimaldi, B., Autophagy is essential for maintaining the growth of a human (mini-)organ: Evidence from scalp hair follicle organ culture. *PLoS Biol* **2018,** *16* (3), e2002864.

178. Nicu, C.; Hardman, J. A.; Pople, J.; Paus, R., Do human dermal adipocytes switch from lipogenesis in anagen to lipophagy and lipolysis during catagen in the human hair cycle? *Exp Dermatol* **2019,** *28* (4), 432-435.

179. Eckhart, L.; Tschachler, E.; Gruber, F., Autophagic Control of Skin Aging. *Front Cell Dev Biol* **2019,** *7*, 143.

180. Arck, P. C.; Overall, R.; Spatz, K.; Liezman, C.; Handjiski, B.; Klapp, B. F.; Birch-Machin, M. A.; Peters, E. M., Towards a "free radical theory of graying": melanocyte apoptosis in the aging human hair follicle is an indicator of oxidative stress induced tissue damage. *FASEB J* **2006,** *20* (9), 1567-9.

181. Emerit, I.; Filipe, P.; Freitas, J.; Vassy, J., Protective effect of superoxide dismutase against hair graying in a mouse model. *Photochem Photobiol* **2004,** *80* (3), 579-82.

182. Kumar, A. B.; Shamim, H.; Nagaraju, U., Premature Graying of Hair: Review with Updates. *Int J Trichology* **2018,** *10* (5), 198-203.

183. Butow, R. A.; Avadhani, N. G., Mitochondrial signaling: the retrograde response. *Mol Cell* **2004,** *14* (1), 1-15.

184. Mori, K.; Ma, W.; Gething, M. J.; Sambrook, J., A transmembrane protein with a cdc2+/CDC28-related kinase activity is required for signaling from the ER to the nucleus. *Cell* **1993,** *74* (4), 743-56.

185. Travers, K. J.; Patil, C. K.; Wodicka, L.; Lockhart, D. J.; Weissman, J. S.; Walter, P., Functional and genomic analyses reveal an essential coordination between the unfolded protein response and ER-associated degradation. *Cell* **2000,** *101* (3), 249-58.

186. Biswas, G.; Anandatheerthavarada, H. K.; Zaidi, M.; Avadhani, N. G., Mitochondria to nucleus stress signaling: a distinctive mechanism of NFkappaB/Rel activation through calcineurin-mediated inactivation of IkappaBbeta. *J Cell Biol* **2003,** *161* (3), 507-19.

187. Bunik, V.; Kaehne, T.; Degtyarev, D.; Shcherbakova, T.; Reiser, G., Novel isoenzyme of 2-oxoglutarate dehydrogenase is identified in brain, but not in heart. *FEBS J* **2008,** *275* (20), 4990-5006.

188. Wang, Y.; Yang, P.; Zhang, B.; Ding, Y.; Lei, S.; Hou, Y.; Guan, X.; Li, Q., Hepatic NPC1L1 overexpression attenuates alcoholic autophagy in mice. *Mol Med Rep* **2019,** *20* (4), 3224-3232.

189. Sen, T.; Sen, N.; Noordhuis, M. G.; Ravi, R.; Wu, T. C.; Ha, P. K.; Sidransky, D.; Hoque, M. O., OGDHL is a modifier of AKT-dependent signaling and NF-kappaB function. *PLoS One* **2012,** *7* (11), e48770.

190. Kaltschmidt, B.; Sparna, T.; Kaltschmidt, C., Activation of NF-kappa B by reactive oxygen intermediates in the nervous system. *Antioxid Redox Signal* **1999,** *1* (2), 129-44.

191. Lu, X.; Yang, P.; Zhao, X.; Jiang, M.; Hu, S.; Ouyang, Y.; Zeng, L.; Wu, J., OGDH mediates the inhibition of SIRT5 on cell proliferation and migration of gastric cancer. *Exp Cell Res* **2019,** *382* (2), 111483.

192. Schippert, R.; Schaeffel, F.; Feldkaemper, M. P., Microarray analysis of retinal gene expression in Egr-1 knockout mice. *Mol Vis* **2009,** *15*, 2720-39.

193. Millington, G. W.; Tung, Y. C.; Hewson, A. K.; O'Rahilly, S.; Dickson, S. L., Differential effects of alpha-, beta- and gamma(2)-melanocyte-stimulating hormones on hypothalamic neuronal activation and feeding in the fasted rat. *Neuroscience* **2001,** *108* (3), 437-45.

194. Cotta-Grand, N.; Rovere, C.; Guyon, A.; Cervantes, A.; Brau, F.; Nahon, J. L., Melanin-concentrating hormone induces neurite outgrowth in human neuroblastoma SH-SY5Y cells through p53 and MAPKinase signaling pathways. *Peptides* **2009,** *30* (11), 2014-24.

195. Travers, J. B.; Weyerbacher, J.; Ocana, J. A.; Borchers, C.; Rapp, C. M.; Sahu, R. P., Acute Ethanol Exposure Augments Low-Dose UVB-Mediated Systemic Immunosuppression via Enhanced Production of Platelet-Activating Factor Receptor Agonists. *J Invest Dermatol* **2019,** *139* (7), 1619-1622.

196. Shin, S. Y.; Choi, J. H.; Jung, E.; Gil, H. N.; Lim, Y.; Lee, Y. H., The EGR1-STAT3 Transcription Factor Axis Regulates alpha-Melanocyte-Stimulating Hormone-Induced Tyrosinase Gene Transcription in Melanocytes. *J Invest Dermatol* **2019,** *139* (7), 1616-1619.

197. Cabello, C. M.; Lamore, S. D.; Bair, W. B., 3rd; Qiao, S.; Azimian, S.; Lesson, J. L.; Wondrak, G. T., The redox antimalarial dihydroartemisinin targets human metastatic melanoma cells but not primary melanocytes with induction of NOXA-dependent apoptosis. *Invest New Drugs* **2012,** *30* (4), 1289-301.

198. Kato, M.; Sato, K.; Habuta, M.; Fujita, H.; Bando, T.; Morizane, Y.; Shiraga, F.; Miyaishi, S.; Ohuchi, H., Localization of the ultraviolet-sensor Opn5m and its effect on myopia-related gene expression in the late-embryonic chick eye. *Biochem Biophys Rep* **2019,** *19*, 100665.

199. Chaum, E.; Yin, J.; Lang, J. C., Molecular responses transduced by serial oxidative stress in the retinal pigment epithelium: feedback control modeling of gene expression. *Neurochem Res* **2011,** *36* (4), 574-82.

200. Sun, T.; Tian, H.; Feng, Y. G.; Zhu, Y. Q.; Zhang, W. Q., Egr-1 promotes cell proliferation and invasion by increasing beta-catenin expression in gastric cancer. *Dig Dis Sci* **2013,** *58* (2), 423-30.

201. Parra, E.; Ferreira, J.; Saenz, L., Inhibition of Egr-1 by siRNA in prostate carcinoma cell lines is associated with decreased expression of AP-1 and NF-kappaB. *Int J Mol Med* **2011,** *28* (5), 847-53.

202. Lobanova, M. V.; Ratushnyy, A. Y.; Buravkova, L. B., Expression of senescence-associated genes in multipotent mesenchymal stromal cells during long-term cultivation at various hypoxic levels. *Dokl Biochem Biophys* **2016,** *470* (1), 326-328.

203. Wu, M.; Zhang, S.; Chen, X.; Xu, H.; Li, X., Expression and function of lncRNA MALAT-1 in the embryonic development of zebrafish. *Gene* **2019,** *680*, 65-71.

204. Sastry, K. S.; Naeem, H.; Mokrab, Y.; Chouchane, A. I., RNA-seq Reveals Dysregulation of Novel Melanocyte Genes upon Oxidative Stress: Implications in Vitiligo Pathogenesis. *Oxid Med Cell Longev* **2019,** *2019*, 2841814.

205. Denat, L.; Kadekaro, A. L.; Marrot, L.; Leachman, S. A.; Abdel-Malek, Z. A., Melanocytes as instigators and victims of oxidative stress. *J Invest Dermatol* **2014,** *134* (6), 1512-1518.

206. Hirschfield, G. M.; Gershwin, M. E., The immunobiology and pathophysiology of primary biliary cirrhosis. *Annu Rev Pathol* **2013,** *8*, 303-30.

207. Xu, Y.; Shen, J.; Ran, Z., Emerging views of mitophagy in immunity and autoimmune diseases. *Autophagy* **2020,** *16* (1), 3-17.

208. Lee, S. H.; Lee, S.; Du, J.; Jain, K.; Ding, M.; Kadado, A. J.; Atteya, G.; Jaji, Z.; Tyagi, T.; Kim, W. H.; Herzog, R. I.; Patel, A.; Ionescu, C. N.; Martin, K. A.; Hwa, J., Mitochondrial MsrB2 serves as a switch and transducer for mitophagy. *EMBO Mol Med* **2019,** *11* (8), e10409.

209. Liu, J.; Liu, W.; Li, R.; Yang, H., Mitophagy in Parkinson's Disease: From Pathogenesis to Treatment. *Cells* **2019,** *8* (7).

210. Chakravorty, A.; Jetto, C. T.; Manjithaya, R., Dysfunctional Mitochondria and Mitophagy as Drivers of Alzheimer's Disease Pathogenesis. *Front Aging Neurosci* **2019,** *11*, 311.

211. Maes, H.; Agostinis, P., Autophagy and mitophagy interplay in melanoma progression. *Mitochondrion* **2014,** *19 Pt A*, 58-68.

212. Yoon, W. H.; Sandoval, H.; Nagarkar-Jaiswal, S.; Jaiswal, M.; Yamamoto, S.; Haelterman, N. A.; Putluri, N.; Putluri, V.; Sreekumar, A.; Tos, T.; Aksoy, A.; Donti, T.; Graham, B. H.; Ohno, M.; Nishi, E.; Hunter, J.; Muzny, D. M.; Carmichael, J.; Shen, J.; Arboleda, V. A.; Nelson, S. F.; Wangler, M. F.; Karaca, E.; Lupski, J. R.; Bellen, H. J., Loss of Nardilysin, a Mitochondrial Co-chaperone for alpha-Ketoglutarate Dehydrogenase, Promotes mTORC1 Activation and Neurodegeneration. *Neuron* **2017,** *93* (1), 115-131.

213. Wood, J. M.; Decker, H.; Hartmann, H.; Chavan, B.; Rokos, H.; Spencer, J. D.; Hasse, S.; Thornton, M. J.; Shalbaf, M.; Paus, R.; Schallreuter, K. U., Senile hair graying: H2O2-mediated oxidative stress affects human hair color by blunting methionine sulfoxide repair. *FASEB J* **2009,** *23* (7), 2065-75.

214. Schallreuter, K. U.; Rubsam, K.; Chavan, B.; Zothner, C.; Gillbro, J. M.; Spencer, J. D.; Wood, J. M., Functioning methionine sulfoxide reductases A and B are present in human epidermal melanocytes in the cytosol and in the nucleus. *Biochem Biophys Res Commun* **2006,** *342* (1), 145-52.

215. Zhou, Z.; Li, C. Y.; Li, K.; Wang, T.; Zhang, B.; Gao, T. W., Decreased methionine sulphoxide reductase A expression renders melanocytes more sensitive to oxidative stress: a possible cause for melanocyte loss in vitiligo. *Br J Dermatol* **2009,** *161* (3), 504-9.

216. Itou, T., Morphological changes in hair melanosomes by aging. *Pigment Cell Melanoma Res* **2018,** *31* (5), 630-635.

217. Tobin, D. J., Age-related hair pigment loss. *Curr Probl Dermatol* **2015,** *47*, 128-38.

218. DiGiovanna, J. J.; Kraemer, K. H., Shining a light on xeroderma pigmentosum. *J Invest Dermatol* **2012,** *132* (3 Pt 2), 785-96.

219. Fang, E. F.; Scheibye-Knudsen, M.; Brace, L. E.; Kassahun, H.; SenGupta, T.; Nilsen, H.; Mitchell, J. R.; Croteau, D. L.; Bohr, V. A., Defective mitophagy in XPA via PARP-1 hyperactivation and NAD(+)/SIRT1 reduction. *Cell* **2014,** *157* (4), 882-896.

220. Zanchetta, L. M.; Garcia, A.; Lyng, F.; Walsh, J.; Murphy, J. E., Mitophagy and mitochondrial morphology in human melanoma-derived cells post exposure to simulated sunlight. *Int J Radiat Biol* **2011,** *87* (5), 506-17.

221. Basit, F.; van Oppen, L. M.; Schockel, L.; Bossenbroek, H. M.; van Emst-de Vries, S. E.; Hermeling, J. C.; Grefte, S.; Kopitz, C.; Heroult, M.; Hgm Willems, P.; Koopman, W. J., Mitochondrial complex I inhibition triggers a mitophagy-dependent ROS increase leading to necroptosis and ferroptosis in melanoma cells. *Cell Death Dis* **2017,** *8* (3), e2716.

222. Song, C.; Hotz-Wagenblatt, A.; Voit, R.; Grummt, I., SIRT7 and the DEAD-box helicase DDX21 cooperate to resolve genomic R loops and safeguard genome stability. *Genes Dev* **2017,** *31* (13), 1370-1381.

223. Soengas, M. S., Mitophagy or how to control the Jekyll and Hyde embedded in mitochondrial metabolism: implications for melanoma progression and drug resistance. *Pigment Cell Melanoma Res* **2012,** *25* (6), 721-31.

224. Kim, E. S.; Jo, Y. K.; Park, S. J.; Chang, H.; Shin, J. H.; Choi, E. S.; Kim, J. B.; Seok, S. H.; Kim, J. S.; Oh, J. S.; Kim, M. H.; Lee, E. H.; Cho, D. H., ARP101 inhibits alpha-MSH-stimulated melanogenesis by regulation of autophagy in melanocytes. *FEBS Lett* **2013,** *587* (24), 3955-60.

225. Ding, G. Z.; Zhao, W. E.; Li, X.; Gong, Q. L.; Lu, Y., A comparative study of mitochondrial ultrastructure in melanocytes from perilesional vitiligo skin and perilesional halo nevi skin. *Arch Dermatol Res* **2015,** *307* (3), 281-9.

226. Ho, H.; Kapadia, R.; Al-Tahan, S.; Ahmad, S.; Ganesan, A. K., WIPI1 coordinates melanogenic gene transcription and melanosome formation via TORC1 inhibition. *J Biol Chem* **2011,** *286* (14), 12509-23.

227. van den Boorn, J. G.; Picavet, D. I.; van Swieten, P. F.; van Veen, H. A.; Konijnenberg, D.; van Veelen, P. A.; van Capel, T.; Jong, E. C.; Reits, E. A.; Drijfhout, J. W.; Bos, J. D.; Melief, C. J.; Luiten, R. M., Skin-depigmenting agent monobenzone induces potent T-cell autoimmunity toward pigmented cells by tyrosinase haptenation and melanosome autophagy. *J Invest Dermatol* **2011,** *131* (6), 1240-51.

228. Ganesan, A. K.; Ho, H.; Bodemann, B.; Petersen, S.; Aruri, J.; Koshy, S.; Richardson, Z.; Le, L. Q.; Krasieva, T.; Roth, M. G.; Farmer, P.; White, M. A., Genome-wide siRNA-based functional genomics of pigmentation identifies novel genes and pathways that impact melanogenesis in human cells. *PLoS Genet* **2008,** *4* (12), e1000298.

229. Xiao, L.; Zhang, R. Z.; Zhu, W. Y., The distribution of melanocytes and the degradation of melanosomes in fetal hair follicles. *Micron* **2019,** *119*, 109-116.

230. Pietzsch, S.; Ricke-Hoch, M.; Stapel, B.; Hilfiker-Kleiner, D., Modulation of cardiac AKT and STAT3 signalling in preclinical cancer models and their impact on the heart. *Biochim Biophys Acta Mol Cell Res* **2020,** *1867* (3), 118519.

231. Strating, J. R.; Bouw, G.; Hafmans, T. G.; Martens, G. J., p24 Proteins from the same subfamily are functionally nonredundant. *Biochimie* **2011,** *93* (3), 528-32.

232. Strating, J. R.; Hafmans, T. G.; Martens, G. J., Functional diversity among p24 subfamily members. *Biol Cell* **2009,** *101* (4), 207-19.

233. Rotter, J.; Kuiper, R. P.; Bouw, G.; Martens, G. J., Cell-type-specific and selectively induced expression of members of the p24 family of putative cargo receptors. *J Cell Sci* **2002,** *115* (Pt 5), 1049-58.

234. Wen, C.; Greenwald, I., p24 proteins and quality control of LIN-12 and GLP-1 trafficking in Caenorhabditis elegans. *J Cell Biol* **1999,** *145* (6), 1165-75.

235. Yang, Z.; Sun, Q.; Guo, J.; Wang, S.; Song, G.; Liu, W.; Liu, M.; Tang, H., GRSF1-mediated MIR-G-1 promotes malignant behavior and nuclear autophagy by directly upregulating TMED5 and LMNB1 in cervical cancer cells. *Autophagy* **2019,** *15* (4), 668-685.

236. Fuller-Pace, F. V., DExD/H box RNA helicases: multifunctional proteins with important roles in transcriptional regulation. *Nucleic Acids Res* **2006,** *34* (15), 4206-15.

237. Yang, H.; Henning, D.; Valdez, B. C., Functional interaction between RNA helicase II/Gu(alpha) and ribosomal protein L4. *FEBS J* **2005,** *272* (15), 3788-802.

238. Calo, E.; Gu, B.; Bowen, M. E.; Aryan, F.; Zalc, A.; Liang, J.; Flynn, R. A.; Swigut, T.; Chang, H. Y.; Attardi, L. D.; Wysocka, J., Tissue-selective effects of nucleolar stress and rDNA damage in developmental disorders. *Nature* **2018,** *554* (7690), 112-117.

239. Holmstrom, T. H.; Mialon, A.; Kallio, M.; Nymalm, Y.; Mannermaa, L.; Holm, T.; Johansson, H.; Black, E.; Gillespie, D.; Salminen, T. A.; Langel, U.; Valdez, B. C.; Westermarck, J., c-Jun supports ribosomal RNA processing and nucleolar localization of RNA helicase DDX21. *J Biol Chem* **2008,** *283* (11), 7046-53.

240. Kouyama, Y.; Masuda, T.; Fujii, A.; Ogawa, Y.; Sato, K.; Tobo, T.; Wakiyama, H.; Yoshikawa, Y.; Noda, M.; Tsuruda, Y.; Kuroda, Y.; Eguchi, H.; Ishida, F.; Kudo, S. E.; Mimori, K., Oncogenic splicing abnormalities induced by DEAD-Box Helicase 56 amplification in colorectal cancer. *Cancer Sci* **2019,** *110* (10), 3132-3144.

241. Cao, J.; Wu, N.; Han, Y.; Hou, Q.; Zhao, Y.; Pan, Y.; Xie, X.; Chen, F., DDX21 promotes gastric cancer proliferation by regulating cell cycle. *Biochem Biophys Res Commun* **2018,** *505* (4), 1189-1194.

242. Santoriello, C.; Sporrij, A.; Yang, S.; Flynn, R. A.; Henriques, T.; Dorjsuren, B.; Custo Greig, E.; McCall, W.; Stanhope, M. E.; Fazio, M.; Superdock, M.; Lichtig, A.; Adatto, I.; Abraham, B. J.; Kalocsay, M.; Jurynec, M.; Zhou, Y.; Adelman, K.; Calo, E.; Zon, L. I., RNA helicase DDX21 mediates nucleotide stress responses in neural crest and melanoma cells. *Nat Cell Biol* **2020,** *22* (4), 372-379.

243. Johansson, J. A.; Marie, K. L.; Lu, Y.; Brombin, A.; Santoriello, C.; Zeng, Z.; Zich, J.; Gautier, P.; von Kriegsheim, A.; Brunsdon, H.; Wheeler, A. P.; Dreger, M.; Houston, D. R.; Dooley, C. M.; Sims, A. H.; Busch-Nentwich, E. M.; Zon, L. I.; Illingworth, R. S.; Patton, E. E., PRL3-DDX21 Transcriptional Control of Endolysosomal Genes Restricts Melanocyte Stem Cell Differentiation. *Dev Cell* **2020**.

244. Eberle, J.; Fecker, L. F.; Bittner, J. U.; Orfanos, C. E.; Geilen, C. C., Decreased proliferation of human melanoma cell lines caused by antisense RNA against translation factor eIF-4A1. *Br J Cancer* **2002,** *86* (12), 1957-62.

245. Kung, C. P.; Leu, J. I.; Basu, S.; Khaku, S.; Anokye-Danso, F.; Liu, Q.; George, D. L.; Ahima, R. S.; Murphy, M. E., The P72R Polymorphism of p53 Predisposes to Obesity and Metabolic Dysfunction. *Cell Rep* **2016,** *14* (10), 2413-25.

246. Yu, L.; Bharadwaj, S.; Brown, J. M.; Ma, Y.; Du, W.; Davis, M. A.; Michaely, P.; Liu, P.; Willingham, M. C.; Rudel, L. L., Cholesterol-regulated translocation of NPC1L1 to the cell surface facilitates free cholesterol uptake. *J Biol Chem* **2006,** *281* (10), 6616-24.

247. Davies, J. P.; Levy, B.; Ioannou, Y. A., Evidence for a Niemann-pick C (NPC) gene family: identification and characterization of NPC1L1. *Genomics* **2000,** *65* (2), 137-45.

248. Chu, B. B.; Ge, L.; Xie, C.; Zhao, Y.; Miao, H. H.; Wang, J.; Li, B. L.; Song, B. L., Requirement of myosin Vb.Rab11a.Rab11-FIP2 complex in cholesterol-regulated translocation of NPC1L1 to the cell surface. *J Biol Chem* **2009,** *284* (33), 22481-90.

249. Schallreuter, K. U.; Hasse, S.; Rokos, H.; Chavan, B.; Shalbaf, M.; Spencer, J. D.; Wood, J. M., Cholesterol regulates melanogenesis in human epidermal melanocytes and melanoma cells. *Exp Dermatol* **2009,** *18* (8), 680-8.

250. Goritz, C.; Mauch, D. H.; Pfrieger, F. W., Multiple mechanisms mediate cholesterol-induced synaptogenesis in a CNS neuron. *Mol Cell Neurosci* **2005,** *29* (2), 190-201.

251. Mauch, D. H.; Nagler, K.; Schumacher, S.; Goritz, C.; Muller, E. C.; Otto, A.; Pfrieger, F. W., CNS synaptogenesis promoted by glia-derived cholesterol. *Science* **2001,** *294* (5545), 1354-7.

252. Cheng, J. P. X.; Nichols, B. J., Caveolae: One Function or Many? *Trends Cell Biol* **2016,** *26* (3), 177-189.

253. Gu, X.; Reagan, A. M.; McClellan, M. E.; Elliott, M. H., Caveolins and caveolae in ocular physiology and pathophysiology. *Prog Retin Eye Res* **2017,** *56*, 84-106.

254. Domingues, L.; Hurbain, I.; Gilles-Marsens, F.; Sires-Campos, J.; Andre, N.; Dewulf, M.; Romao, M.; Viaris de Lesegno, C.; Mace, A. S.; Blouin, C.; Guere, C.; Vie, K.; Raposo, G.; Lamaze, C.; Delevoye, C., Coupling of melanocyte signaling and mechanics by caveolae is required for human skin pigmentation. *Nat Commun* **2020,** *11* (1), 2988.

255. Schmitz, M.; Signore, S. C.; Zerr, I.; Althaus, H. H., Oligodendroglial process formation is differentially affected by modulating the intra- and extracellular cholesterol content. *J Mol Neurosci* **2013,** *49* (3), 457-69.

256. Yu, J.; Li, X.; Matei, N.; McBride, D.; Tang, J.; Yan, M.; Zhang, J. H., Ezetimibe, a NPC1L1 inhibitor, attenuates neuronal apoptosis through AMPK dependent autophagy activation after MCAO in rats. *Exp Neurol* **2018,** *307*, 12-23.

257. Natale, C. A.; Duperret, E. K.; Zhang, J.; Sadeghi, R.; Dahal, A.; O'Brien, K. T.; Cookson, R.; Winkler, J. D.; Ridky, T. W., Sex steroids regulate skin pigmentation through nonclassical membrane-bound receptors. *Elife* **2016,** *5*.

258. Fabian, M.; Rencz, F.; Krenacs, T.; Brodszky, V.; Harsing, J.; Nemeth, K.; Balogh, P.; Karpati, S., Expression of G protein-coupled oestrogen receptor in melanoma and in pregnancy-associated melanoma. *J Eur Acad Dermatol Venereol* **2017,** *31* (9), 1453-1461.

259. Narushima, K.; Takada, T.; Yamanashi, Y.; Suzuki, H., Niemann-pick C1-like 1 mediates alpha-tocopherol transport. *Mol Pharmacol* **2008,** *74* (1), 42-9.

260. Hanson, M.; Lupski, J. R.; Hicks, J.; Metry, D., Association of dermal melanocytosis with lysosomal storage disease: clinical features and hypotheses regarding pathogenesis. *Arch Dermatol* **2003,** *139* (7), 916-20.

261. Schmuth, M.; Man, M. Q.; Weber, F.; Gao, W.; Feingold, K. R.; Fritsch, P.; Elias, P. M.; Holleran, W. M., Permeability barrier disorder in Niemann-Pick disease: sphingomyelin-ceramide processing required for normal barrier homeostasis. *J Invest Dermatol* **2000,** *115* (3), 459-66.

262. Sitaram, A.; Marks, M. S., Mechanisms of protein delivery to melanosomes in pigment cells. *Physiology (Bethesda)* **2012,** *27* (2), 85-99.

263. Delevoye, C.; Hurbain, I.; Tenza, D.; Sibarita, J. B.; Uzan-Gafsou, S.; Ohno, H.; Geerts, W. J.; Verkleij, A. J.; Salamero, J.; Marks, M. S.; Raposo, G., AP-1 and KIF13A coordinate endosomal sorting and positioning during melanosome biogenesis. *J Cell Biol* **2009,** *187* (2), 247-64.

264. Raposo, G.; Marks, M. S., Melanosomes--dark organelles enlighten endosomal membrane transport. *Nat Rev Mol Cell Biol* **2007,** *8* (10), 786-97.

265. Dennis, M. K.; Mantegazza, A. R.; Snir, O. L.; Tenza, D.; Acosta-Ruiz, A.; Delevoye, C.; Zorger, R.; Sitaram, A.; de Jesus-Rojas, W.; Ravichandran, K.; Rux, J.; Sviderskaya, E. V.; Bennett, D. C.; Raposo, G.; Marks, M. S.; Setty, S. R., BLOC-2 targets recycling endosomal tubules to melanosomes for cargo delivery. *J Cell Biol* **2015,** *209* (4), 563-77.

266. Raposo, G.; Marks, M. S., The dark side of lysosome-related organelles: specialization of the endocytic pathway for melanosome biogenesis. *Traffic* **2002,** *3* (4), 237-48.

267. Tarafder, A. K.; Bolasco, G.; Correia, M. S.; Pereira, F. J. C.; Iannone, L.; Hume, A. N.; Kirkpatrick, N.; Picardo, M.; Torrisi, M. R.; Rodrigues, I. P.; Ramalho, J. S.; Futter, C. E.; Barral, D. C.; Seabra, M. C., Rab11b mediates melanin transfer between donor melanocytes and acceptor keratinocytes via coupled exo/endocytosis. *J Invest Dermatol* **2014,** *134* (4), 1056-1066.

268. Jani, R. A.; Purushothaman, L. K.; Rani, S.; Bergam, P.; Setty, S. R., STX13 regulates cargo delivery from recycling endosomes during melanosome biogenesis. *J Cell Sci* **2015,** *128* (17), 3263-76.

269. Morris, S. M.; Anaya, P.; Xiang, X.; Morris, N. R.; May, G. S.; Yu-Lee, L., A prolactin-inducible T cell gene product is structurally similar to the Aspergillus nidulans nuclear movement protein NUDC. *Mol Endocrinol* **1997,** *11* (2), 229-36.

270. Echtenkamp, F. J.; Zelin, E.; Oxelmark, E.; Woo, J. I.; Andrews, B. J.; Garabedian, M.; Freeman, B. C., Global functional map of the p23 molecular chaperone reveals an extensive cellular network. *Mol Cell* **2011,** *43* (2), 229-41.

271. Zhou, T.; Aumais, J. P.; Liu, X.; Yu-Lee, L. Y.; Erikson, R. L., A role for Plk1 phosphorylation of NudC in cytokinesis. *Dev Cell* **2003,** *5* (1), 127-38.

272. Aumais, J. P.; Williams, S. N.; Luo, W.; Nishino, M.; Caldwell, K. A.; Caldwell, G. A.; Lin, S. H.; Yu-Lee, L. Y., Role for NudC, a dynein-associated nuclear movement protein, in mitosis and cytokinesis. *J Cell Sci* **2003,** *116* (Pt 10), 1991-2003.

273. Zhang, C.; Zhang, W.; Lu, Y.; Yan, X.; Yan, X.; Zhu, X.; Liu, W.; Yang, Y.; Zhou, T., NudC regulates actin dynamics and ciliogenesis by stabilizing cofilin 1. *Cell Res* **2016,** *26* (2), 239-53.

274. Zhou, T.; Zimmerman, W.; Liu, X.; Erikson, R. L., A mammalian NudC-like protein essential for dynein stability and cell viability. *Proc Natl Acad Sci U S A* **2006,** *103* (24), 9039-44.

275. Shao, C. Y.; Zhu, J.; Xie, Y. J.; Wang, Z.; Wang, Y. N.; Wang, Y.; Su, L. D.; Zhou, L.; Zhou, T. H.; Shen, Y., Distinct functions of nuclear distribution proteins LIS1, Ndel1 and NudCL in regulating axonal mitochondrial transport. *Traffic* **2013,** *14* (7), 785-97.

276. Liu, A.; Wang, B.; Niswander, L. A., Mouse intraflagellar transport proteins regulate both the activator and repressor functions of Gli transcription factors. *Development* **2005,** *132* (13), 3103-11.

277. Asante, D.; Stevenson, N. L.; Stephens, D. J., Subunit composition of the human cytoplasmic dynein-2 complex. *J Cell Sci* **2014,** *127* (Pt 21), 4774-87.

278. Fu, Q.; Wang, W.; Zhou, T.; Yang, Y., Emerging roles of NudC family: from molecular regulation to clinical implications. *Sci China Life Sci* **2016,** *59* (5), 455-62.

279. Riera, J.; Lazo, P. S., The mammalian NudC-like genes: a family with functions other than regulating nuclear distribution. *Cell Mol Life Sci* **2009,** *66* (14), 2383-90.

280. Chen, X. J.; Xu, H.; Cooper, H. M.; Liu, Y., Cytoplasmic dynein: a key player in neurodegenerative and neurodevelopmental diseases. *Sci China Life Sci* **2014,** *57* (4), 372-7.

281. Roberts, A. J.; Kon, T.; Knight, P. J.; Sutoh, K.; Burgess, S. A., Functions and mechanics of dynein motor proteins. *Nat Rev Mol Cell Biol* **2013,** *14* (11), 713-26.

282. Bennett, M. R.; Lagopoulos, J., Stress and trauma: BDNF control of dendritic-spine formation and regression. *Prog Neurobiol* **2014,** *112*, 80-99.

283. Ciani, L.; Salinas, P. C., From neuronal activity to the actin cytoskeleton: a role for CaMKKs and betaPIX in spine morphogenesis. *Neuron* **2008,** *57* (1), 3-4.

284. Jurek, B.; Neumann, I. D., The Oxytocin Receptor: From Intracellular Signaling to Behavior. *Physiol Rev* **2018,** *98* (3), 1805-1908.

285. Su, Y.; Yang, L.; Li, Z.; Wang, W.; Xing, M.; Fang, Y.; Cheng, Y.; Lin, G. N.; Cui, D., The interaction of ASAH1 and NGF gene involving in neurotrophin signaling pathway contributes to schizophrenia susceptibility and psychopathology. *Prog Neuropsychopharmacol Biol Psychiatry* **2020,** *104*, 110015.

286. Buac, K.; Xu, M.; Cronin, J.; Weeraratna, A. T.; Hewitt, S. M.; Pavan, W. J., NRG1 / ERBB3 signaling in melanocyte development and melanoma: inhibition of differentiation and promotion of proliferation. *Pigment Cell Melanoma Res* **2009,** *22* (6), 773-84.

287. Yamaguchi, Y.; Hearing, V. J., Physiological factors that regulate skin pigmentation. *Biofactors* **2009,** *35* (2), 193-9.

288. Yaar, M.; Park, H. Y., Melanocytes: a window into the nervous system. *J Invest Dermatol* **2012,** *132* (3 Pt 2), 835-45.

289. Marconi, A.; Panza, M. C.; Bonnet-Duquennoy, M.; Lazou, K.; Kurfurst, R.; Truzzi, F.; Lotti, R.; De Santis, G.; Dumas, M.; Bonte, F.; Pincelli, C., Expression and function of neurotrophins and their receptors in human melanocytes. *Int J Cosmet Sci* **2006,** *28* (4), 255-61.

290. Bothwell, M., Neurotrophin function in skin. *J Investig Dermatol Symp Proc* **1997,** *2* (1), 27-30.

291. Kriegenburg, F.; Bas, L.; Gao, J.; Ungermann, C.; Kraft, C., The multi-functional SNARE protein Ykt6 in autophagosomal fusion processes. *Cell Cycle* **2019,** *18* (6-7), 639-651.

292. Mizushima, N.; Matsui, T.; Yamamoto, H., YKT6 as a second SNARE protein of mammalian autophagosomes. *Autophagy* **2019,** *15* (1), 176-177.

293. Yong, C. Q. Y.; Tang, B. L., Another longin SNARE for autophagosome-lysosome fusion-how does Ykt6 work? *Autophagy* **2019,** *15* (2), 352-357.

294. Bas, L.; Papinski, D.; Kraft, C., Ykt6 mediates autophagosome-vacuole fusion. *Mol Cell Oncol* **2018,** *5* (6), e1526006.

295. Gao, J.; Reggiori, F.; Ungermann, C., A novel in vitro assay reveals SNARE topology and the role of Ykt6 in autophagosome fusion with vacuoles. *J Cell Biol* **2018,** *217* (10), 3670-3682.

296. Takats, S.; Glatz, G.; Szenci, G.; Boda, A.; Horvath, G. V.; Hegedus, K.; Kovacs, A. L.; Juhasz, G., Non-canonical role of the SNARE protein Ykt6 in autophagosome-lysosome fusion. *PLoS Genet* **2018,** *14* (4), e1007359.

297. Naydenov, N. G.; Joshi, S.; Feygin, A.; Saini, S.; Litovchick, L.; Ivanov, A. I., A membrane fusion protein, Ykt6, regulates epithelial cell migration via microRNA-mediated suppression of Junctional Adhesion Molecule A. *Cell Cycle* **2018,** *17* (14), 1812-1831.

298. Linnemannstons, K.; Witte, L.; Karuna, P.; Kittel, J.; Danieli, A.; Muller, D.; Nitsch, L.; Honemann-Capito, M.; Grawe, F.; Wodarz, A.; Gross, J. C., Ykt6-dependent endosomal recycling is required for Wnt secretion in the Drosophila wing epithelium. *Development* **2020**.

299. Cuddy, L. K.; Wani, W. Y.; Morella, M. L.; Pitcairn, C.; Tsutsumi, K.; Fredriksen, K.; Justman, C. J.; Grammatopoulos, T. N.; Belur, N. R.; Zunke, F.; Subramanian, A.; Affaneh, A.; Lansbury, P. T., Jr.; Mazzulli, J. R., Stress-Induced Cellular Clearance Is Mediated by the SNARE Protein ykt6 and Disrupted by alpha-Synuclein. *Neuron* **2019,** *104* (5), 869-884 e11.

300. Rosengren Pielberg, G.; Golovko, A.; Sundstrom, E.; Curik, I.; Lennartsson, J.; Seltenhammer, M. H.; Druml, T.; Binns, M.; Fitzsimmons, C.; Lindgren, G.; Sandberg, K.; Baumung, R.; Vetterlein, M.; Stromberg, S.; Grabherr, M.; Wade, C.; Lindblad-Toh, K.; Ponten, F.; Heldin, C. H.; Solkner, J.; Andersson, L., A cis-acting regulatory mutation causes premature hair graying and susceptibility to melanoma in the horse. *Nat Genet* **2008,** *40* (8), 1004-9.
