# Supplementary material for: Fifteen Shades of Grey: Combined Analysis of Genome-Wide SNP Data in Steppe and Mediterranean Grey Cattle Sheds New Light on the Molecular Basis of Coat Color
Source: Genes (Basel). 2020 Aug 13;11(8):932. doi: 10.3390/genes11080932 (PMC7464420; doi:10.3390/genes11080932)
Supplement: Supplementary file 1 [file genes-11-00932-s001.zip › Supplementary file S3.docx]

**Supplementary file S3.** Functions of genes in the ± 250 kbp regions upstream and downstream the nine SNP loci on BTA14, detected as significant in a number of pair-wise comparisons ranging between 15 and 36, and, in all instances, at least for 2 different reference breeds.

***XKR4*** (XK Related 4) encodes a protein that has been shown to be expressed in the brain and suggested to **promote exposure of phosphatidylserine by apoptotic cells** [1]. It belongs to the XKR family that consists of 9 different family members, XKR1-9. A role in promoting exposure of phosphatidylserine (scramblase activity) when cells undergo apoptosis has been shown also for XKR8 [2-4] that has been also shown to be involved in exposure of phosphatidylserine by **senescent** neutrophils [5]. Moreover, the findings of Kim et al. [6] suggest that XKR8 may be required for phosphatidylserine exposure in myoblasts in preparation for fusion and may plays an active role during **myoblast differentiation**. The asymmetry of phosphatidylserine across the plasma membrane plays a crucial signaling role in numerous physiological processes. Many peripheral and integral membrane proteins have phosphatidylserine-binding domains on their cytoplasmic surfaces which either provide a membrane anchor or affect activity. These domains can also determine **trafficking within the cell**. As an example, exophilin4/Slp2-a localizes on **phosphatidylserine**-enriched plasma membrane, and its N-terminal Rab27-binding domain (RBD27) specifically recognizes Rab27 on the surfaces of melanosomes and secretory granules prior to docking and fusion, with Rab27 dysfunction causing the **Griscelli syndrome** [7].

Interestingly, *XKR4* was found in a candidate region for differential selection by contrasting three **Korean cattle breeds displaying different coat color phenotypes** [8]. In a genome-wide association study including taurine, indicine and composite breeds, five SNPs in and around the *XKR4* gene were found to be significantly associated with **rump fat thickness** This gene and its immediate surrounding genes are also interesting because gene frequencies in the region show **evidence of population genetic selection** [9]. Using a large sample of taurine–indicine composite cattle, Porto Neto et al. [9] highlighted that the relatively large quantitative trait locus effect detected at this locus appears to be a **result of the variation in indicine and taurine–indicine composite cattle.** It was detected within a **selection signature in cattle with various indicine introgression levels** [10].

Sun et al. [11] demonstrate that *XKR5* is a novel negative **regulator of KIT signaling**. Interaction of XKR5 with KIT leads to phosphorylation of XKR5. Tyrosine phosphorylated XKR5 acts as a negative regulator of KIT signaling, which leads to downregulation of phosphorylation of ERK, AKT, and p38. The biological effects are **reduced proliferation and reduced colony forming capacity** in semi-solid medium. The mechanism by which XKR5 exerts its effect is not known.

By exploring the role of **somatic synonymous mutations in regulatory elements in melanoma** samples from The Cancer Genome Atlas, Zhang [12] identified 12 genes that were hit by recurrent potentially functional synonymous mutations, which showed statistical significance in the pathogenic mutations. Among them, *XKR6* had been previously reported to be mutated in melanoma [13]. SNPs at the gene *XKR6* were shown to be significantly associated with the auto-immune disease **systemic lupus erythematosus** (SLE), or to be correlated with increased **serum triglycerides levels** in patients with coronary and heart disease and **total cholesterol levels** in patients with ischemic stroke and a high risk of coronary and heart disease and ischemic stroke [14].

The expression of the KX blood group system is critical to normal red blood cells morphology, and null mutations are associated with the McLeod neuroacanthocytosis syndrome, characterized by hematological and **neurological abnormalities**. XK is a predicted membrane transporter expressed ubiquitously. In erythrocytes, it is linked to the endothelin-3-converting enzyme Kell via a single disulfide bond. Linked to Kell, XK binds to protein 4.1R, which itself binds to β-spectrin at the binding junction of spectrin and actin, forming a 4.1R–spectrin–actin ternary complex of **cytoskeleton** in erythrocytes At neurological level, the most striking change in mice lacking XK is the presence of **giant axons** [15].

***TRNAT-AGU*** (Transfer RNA threonine) tRNA

***TMEM68*** (Transmembrane Protein 68) encodes a protein that has been suggested to belong to the glycerophospholipid acyltransferase family. Chang et al. [16] described it as an integral **ER membrane protein** and a putative acyltransferase **involved in brain glycerolipid metabolism**. Alike *XKR4* (see above), *TMEM68* was found in a candidate region for differential selection by contrasting three Korean cattle breeds displaying **different coat color phenotypes** [8]. In addition, it was found associated with various phenotypic traits in cattle of indicine origin [17,18].

***TGS1*** (Trimethylguanosine Synthase 1) encodes an essential cytoplasmic methyltransferase. Methyltransferases are involved in many different cellular processes including the post-transcriptional modification of RNAs. TGS1 catalyzes the 2 serial methylation steps for the conversion of the 7-monomethylguanosine (m7G) caps of snRNAs (small nuclear RNAs), including spliceosomal uridyl-rich small nuclear RNAs (UsnRNAs), and snoRNAs (small nucleolar RNAs) to a 2,2,7-trimethylguanosine (m(2,2,7)G) cap structure. The enzyme is specific for guanine, and N7 methylation must precede N2 methylation. Hypermethylation of the m7G cap of U snRNAs leads to their concentration in nuclear foci, their colocalization with coilin and the formation of canonical Cajal bodies (CBs). It plays a role in **transcriptional regulation**.

UsnRNA 5’-cap hypermethylation plays an important role during the biogenesis of UsnRNPs (uridyl-rich small nuclear ribonucleoprotein particles). In higher eukaryotes, the maturation of UsnRNPs comprises a **nucleo-cytoplasmic transport** cycle. Newly transcribed snRNAs U1, U2, U4 and U5 are exported to the cytoplasm in an m7 G cap-dependent manner, where assembly with seven Sm proteins occurs. This assembly process is mediated by the survival of motor neuron complex (SMN complex), a large multiprotein complex. The m7 G cap is subsequently hypermethylated by TGS1 and the resulting m3G cap is recognized by the nuclear import adaptor snurportin1, which binds to the general nuclear import receptor importin. Hence, the m3G cap serves as nuclear import signal that indicates the completed assembly of the core UsnRNP particle. Therefore, the interaction of TGS1 with UsnRNP proteins SmB/B0 and D1 as well as with the SMN complex appears to correlate with the ordered process of **ribonucleoprotein particles assembly** and subsequent cap hypermethylation [19]. Borg et al. [20] highlighted that TGS1 is required for normal [motor behavior](https://www.sciencedirect.com/topics/medicine-and-dentistry/locomotion) and [neuromuscular activity](https://www.sciencedirect.com/topics/medicine-and-dentistry/neuromuscular-function), and that its **disruption induces phenotypes that mirror spinal muscular atrophy**, a neuromuscular disorder, resulting, in the majority of cases, from insufficient levels of the ubiquitously-expressed survival motor neuron (SMN) protein. *TGS1* was detected within a **selection signature in cattle with various indicine introgression levels** [10].

***LYN*** (LYN Proto-Oncogene, Src Family Tyrosine Kinase) encodes a non-receptor tyrosine protein kinase belonging to the Src family tyrosine kinases (SFK). Src family kinases interact with many cellular cytosolic, nuclear and membrane proteins, modifying these proteins by phosphorylation of tyrosine residues. As such, SFKs are involved in the modulation and regulation of many different processes such as **proliferation, differentiation, migration, metabolism and apoptosis, responses to DNA damage and genotoxic agents**. In **melanoma**, *LYN* was shown to play an **oncogenic** role by its contribution to the regulation of cell **proliferation, migration, invasion, apoptosis and autophagy** via the **PI3K/Akt pathway** [21]. Pro-oncogenic activity of *LYN* was also observed in gastric cancer cells [22] where *LYN* knockdown was found to **induce apoptosis** and inhibit both **migration** and **invasion**. *LYN* knockdown resulted in the **activation of the mitochondrial apoptotic pathway**. Likewise, the **Wnt/β-catenin pathway** was inactivated by *LYN* knockdown and **epithelial-mesenchymal transition** mesenchymal markers (including N-cadherin and vimentin) were also found to be downregulated. Finally, the **AKT/mTOR pathway** was found to be downregulated by LYN knockdown in gastric cancer cells.

It has been shown to interact and induce autophosphorylation of **KIT**, to play a role in the control of **KIT expression**, to change intracellular localization in response to stimulation of the receptor tyrosine kinases, ALK and KIT and to be involved in **endosomes and lipid rafts** [23]. It has also been shown to activate a variety of signaling components including Jnks, Erks, **PI3K**, the **JAK-STAT** pathway [24]. It was demonstrated to be involved in the **NF-κB** pathway [25,26]. Inhibition of Src-family kinases was shown to impaire phosphorylation and accumulation of **c-Jun**, leading to reduced formation of **AP-1 complexes** upon LPS stimulation [27].

*LYN* has been shown to be activated via cell detachment through decreased membrane **cholesterol** levels during the change in its membrane distribution [28]. Furthermore, cholesterol incorporation was shown to decreases *LYN* activity, suggesting that the localization and the function of the Src-family kinase *LYN* are critically regulated by its membrane anchorage through lipid modifications. Cholesterol, a major component of the plasma membrane, determines the physical properties of biological membranes and plays a critical role in the assembly of membrane microdomains. Enrichment or deprivation of membrane cholesterol affects the activities of many signaling molecules at the plasma membrane. Cell detachment changes the structure of the plasma membrane and influences the localizations of lipids, including cholesterol [29].

In dendritic cells, the membrane raft-associated LYN Src family kinase is used by CD40, whose receptors associate with **sphingolipid- and cholesterol-rich plasma membrane microdomains**, to initiate CD40 signaling via tyrosine phosphorylation of intracellular substrates, activating a pathway implicating ERK activation and **cytokines (interleukins) production** [30].

Also, *LYN* has been shown to be activated by ligand binding to the neutral glycosphingolipid **lactosylceramide**, that has been shown to form lipid rafts (membrane microdomains) coupled with the Src family kinase LYN on the plasma membranes of human neutrophils. *LYN* activation in neutrophils was associated with neutrophil functions, such as **superoxide generation and migration** [31].

A role for *LYN* and **glucosylceramides** in the **migratory activity** of immortalized human endometrial stromal cells has also been shown [32].

**Cleavage** of LYN during induction of apoptosis may represent another mechanism for the regulation of the Src kinase [33]. Notably, expression of the **caspase-cleaved** form of LYN (LynDeltaN) in mice was shown to mediate a chronic inflammatory syndrome resembling **psoriasis**, altering **NF-kB** activity [34]. In B cells, the mitochondrion-dependent caspase-mediated **cleavage of LYN impair apoptosis** likely through the modulation of **c-Myc** expression [35].

In oligodendrocytes, knockdown of *LYN* resulted in **apoptosis** with concomitant accumulation of C(16)-**ceramide** due to activation of acid **sphingomyelinase** (ASMase) and sphingomyelin hydrolysis. Thus, LYN kinase was suggested to promote cell survival by suppressing acid sphingomyelinase activity [36].

*LYN* has been shown to be a mediator of **epithelial-mesenchymal transition** in breast cancer [37] and to regulate **epithelial-mesenchymal transition** in mice treated with cigarette smoking extracts, through **Smad2/3 signaling** [38].

Also, *LYN* has been shown to be involved in **radiation-induced signal transduction** [39] and to regulate the cell death response to **ultraviolet radiation** through c-Jun N terminal kinase-dependent FAS ligand activation [40]. It has been shown to be among the intracellular signaling molecules activated by **genotoxic stresses**, playing a role in regulating **DNA damage-induced apoptosis** [41].

The Golgi-associated LYN tyrosine kinase has been involved in the **translocation** of annexin II to the **endoplasmic reticulum** under **oxidative stress** [42]. Various findings indicate that Src family members interact in a regulated manner with the **cytoskeleton** [43]. LYN has been shown to interact with phosphoproteins which **regulate cell cycle and the cytoskeleton** [44] and is involved in **phagocytosis**. In glioblastoma cells, **under nutrient-deprived conditions**, constitutively-active *LYN* expression enhanced enhanced AMPK activity and survival and promoted **autophagy** as well as inhibiting cell death and promoting proliferation while expression of dominant-negative *LYN* promoted cell death. The observed findings suggest that on nutrient deprivation in vitro, as well as in xenograft tumors in vivo, LYN acts to enhance the survival of glioblastoma cells by promoting autophagy and proliferation as well as inhibiting cell death [45]. LYN has been shown to be involved in the **polarization of neurons**, with the **differentiation of** **axons and dendrites** [46]. It was shown to contribute to coordination of **motor activity in the brain** through regulation of the pathway of N-methyl-D-aspartate (**NMDA**) receptor, an ionotropic **glutamate receptor** [47] and to be among the signals possibly controlling **migration and differentiation** of the **neural crest cells** during development [23]. *LYN* mutations have been implicated in the ability by estrogen receptor-positive breast cancers **to adapt to estrogen deprivation** and become resistant to antiestrogen therapy [48]. Exposure of differentiating human osteoclasts to **17β-estradiol** induced upregulation of *LYN*. In control condition, estrogen treatment increased the apoptosis rate and suppressed the calcium signaling, resulting in reduced osteoclastogenesis. These osteoclasts were smaller in size with reduced extent of multinuclearity and produced significantly low levels of bone resorbing enzymes. They also exhibited disrupted sealing zone formation with low podosome density, impaired cell polarization and reduced resorption of dentine slices. In *LYN* knockdown condition, **estrogen failed to induce apoptosis**. *LYN* knockdown osteoclasts did not show reduction in production of bone resorbing enzymes and had defined sealing zone formation with high podosome density with no impairment in cell polarization. Thus, the inhibitory action of estrogen on osteoclast was severely restrained in *LYN* knockdown condition, demonstrating the importance of **LYN as a key mediator of the effect of estrogen** on osteoclastogenesis [49]. LYN tyrosine kinase expression was shown to be upregulated in castrate-resistant prostate cancer compared with normal tissue. Castrate-resistant prostate cancer progression is a complex process by which prostate cells acquire the **ability to survive and proliferate in the absence or under very low levels of androgens**. Most castrate-resistant prostate cancer tumors continue to express androgen receptors as well as androgen-responsive genes owing to reactivation of androgen receptors. LYN expression in castrate-resistant prostate cancer was shown to enhance **androgen receptor** transcriptional activity while targeting LYN kinase was shown to induce dissociation of androgen receptors from the molecular chaperone Hsp90, leading to their ubiquitination and proteasomal degradation, indicating a mechanism of regulation of androgen receptor stability and transcriptional activity by LYN [50]. Finally, *LYN* was detected within a selection signature in cattle with various **indicine** introgression levels [10].

***CHCHD7*** (Coiled-Coil-Helix-Coiled-Coil-Helix Domain Containing 7) encodes a protein of the mitochondrial intermembrane space, which is a member of the CHCH-containing twin CX_9_C proteins [51]. This protein functions in **mitochondrial copper homeostasis** and is essential for the expression of functional **cytochrome oxidase** [52]. Mitochondria are cellular organelles that are essential to aerobic ATP production and, as such, energy homeostasis. ATP is generated by the coordinate activity of five multimeric enzyme complexes, most of which rely on iron–sulfur, heme, or copper centers for their catalytic competence. Mitochondria therefore have a vested interest in the cellular metabolism of metal ions. It is perhaps not surprising then that bioactive pools of several metals, including iron and copper, are localized to the mitochondrial matrix, and are crucial to the sustained synthesis and delivery of prosthetic groups that are required by these enzymes for their assembly and proper function. As mitochondria contain bioactive pools of copper and iron, mechanisms must exist to acquire, store, and mobilize these elements. Many of the proteins that mediate the uptake of iron into mitochondria and its subsequent assimilation into heme and iron–sulfur clusters have been identified; however, those critical to mitochondrial copper metabolism remain unknown. What is clear is that copper export from the matrix to the mitochondrial intermembrane space is required for its use in metallating cytochrome c oxidase (COX) subunits I and II during the biogenesis of COX, and in maturing the small fraction of the total cellular copper–zinc **superoxide dismutase** (SOD1) that resides in this compartment. Delivery and insertion of copper into each of these enzymes is surprisingly complex. To date, 12 accessory proteins with essential roles in the maturation of COX, SOD1 or both enzymes have been identified in yeast. Each factor has a human orthologue and contains highly conserved cysteines that are organized as Cx_3_C or Cx_9_C motifs. While it is known that these motifs are used to oxidatively trap soluble accessory proteins within the mitochondrial intermembrane space, thereby ensuring their appropriate localization, most of these proteins have yet to be functionally characterized. However, studies of COX17, SCO1, and SCO2 in a number of in vivo and in vitro systems have provided robust evidence that **redox regulation** of their cysteine residues is crucial to their **metallochaperone functions**. Data also support an important role for redox regulation of the copper-binding cysteines of SCO1 in the transduction of a SCO-dependent, mitochondrial signal that modulates cellular copper homeostasis [53].

It was detected within a number of selection signatures studies and GWAS for body weight and milk characteristics in cattle [54], bovine stature [55], carcass meat yield [56], carcass weight and stature in Japanese Black cattle [57], weight gain and carcass traits in Japanese Black steers [58], birth weight in **indicine** (Nellore) cattle [59], knuckle, biceps and shank weight in Simmental beef cattle (Song ^60^ et al., 2016), beef quality traits in Angus cattle [61] as well as in a selection signature in cattle with various **indicine** introgression levels [10].

***SDR16C5*** (Short Chain Dehydrogenase/Reductase Family 16C Member 5, alias *RDHE2*, epidermal retinol dehydrogenase 2) encodes a member of the short-chain alcohol dehydrogenase/reductase superfamily of proteins and is involved in the **oxidation of retinol to retinaldehyde** [62], one of the steps necessary for conversion of retinol into retinoic acid, the major bioactive form of vitamin A, which influences a broad spectrum of physiological processes during embryogenesis and adulthood, regulating, through interactions with nuclear transcription factors, the retinoic acid receptors, the expression of over 500 genes. Diseases associated with SDR16C5 include **psoriasis**. Interestingly, by screening key genes and possible signaling pathways involved in the **differentiation and proliferation of human melanocytes**, Mei et al. [63] found SDR16C5, as well as the DHRS3, DHRS9, and RDH10 genes in retinol metabolic pathway, continuously down-regulated at different culture time points, suggesting that the differentiation and proliferation of melanocytes may be accompanied by an increase in the expression of genes involved in **retinoic acid catabolism** and a decrease in the expression of synthesis-related genes. As a result, the catabolism of retinoic acid may contribute to the differentiation, proliferation and maturation of cultured melanocytes. Wu et al. [64] showed that mice lacking the epidermal retinol dehydrogenases SDR16C5 display **accelerated hair growth**. A role for retinoic acid signaling in **melanoma cell lines** has been reported by Epping et al. [65] (see Supplementary file S1). Retinoic acid has been shown to work as a **morphogen in embryos** [66]. Deletion of the Notch pathway co-factor RBP-J in mouse hair follicle stem cells was shown to trigger adjacent **melanocyte stem cells to** **precociously differentiate** in their shared niche. Transcriptome screen and in vivo functional studies revealed that the **elevated level of retinoic acid** caused by de-repression of retinoic acid metabolic process genes as a result of RBP-J deletion in hair follicle stem cells triggers **ectopic melanocyte stem cells differentiation** in the niche. Hence, RBP-J functions as a repressor in hair follicle stem cells to suppress retinoid metabolic process genes and consequently the level of retinoic acid in the niche shared by hair follicle stem cells and melanocyte stem cells. Elevated levels of retinoic acid were shown to be both necessary and sufficient to induce **ectopic melanocyte stem cells pigmentation** in the niche in hair cycle dependent fashion, as well as to lead to increased level of c-Kit protein on melanocyte stem cells. The increased level of c-Kit on melanocyte stem cells was shown to be required for their ectopic differentiation and this phenomenon occurred via the c-Kit-MEK pathway. The study showed that it is the KIT-ligand originated from hair follicle stem cells that activates the c-Kit-MEK pathway to induce melanocyte stem cells ectopic differentiation in the mutant niche. Overall, **melanocyte stem cells were shown to maintain self-renewal ability by expressing low level of c-Kit to remain insensitive to the differentiation signals. Their neighboring hair follicle stem cells are not just passive receiver of signaling inputs from the niche, as they actively suppress differentiation-sensitizing signals in the niche, such as retinoid acid level, to help maintain a differentiation refractory niche environment** [67]. The addition of retinoic acid to the medium was shown to significantly **decrease the proliferation of normal human adult epidermal melanocytes** as well as of human undifferentiated epidermal **melanoblasts**, in a dose-dependent manner. It also significantly decreased both MITF and TRP-1 mRNA expression level in normal human adult epidermal melanocytes while MITF and TRP-1 mRNA expression levels in human melanoblasts were significantly increased following the addition of retinoic acid, thus suggesting that retinoic acid has significantly different effects on melanocytes depending on their stage of differentiation. More specifically, **retinoic acid promotes differentiation in earlier stages, in which embryonic stem cells become melanoblasts via neural crest cells, and inhibits differentiation in later stages, in which melanoblasts become melanocytes**. EDNRB was suggested as the key molecule involved in the mechanism of retinoic acid in human melanocytes [68].

It was detected within a GWAS for birth weight in **indicine** (Nellore) cattle [59] as well as in a candidate region for differential selection by contrasting three Korean cattle breeds displaying **different coat color phenotypes** [8].

***SDR16C6*** (Short Chain Dehydrogenase/Reductase Family 16C Member 6) Pseudogene

***PENK*** (Proenkephalin) is an endogenous opioid polypeptide hormone and a negative regulator of **cell proliferation** and tissue organization. A number of immunocytochemical and biochemical studies have shown that the peptides are concentrated heavily in the **hypothalamus** of various species. All hypothalamic structures except the mammillary bodies have been shown to contain PENK neurons. The massive numbers of PENK neurons throughout hypothalamic structures suggest that PENK neurons participate in numerous homeostatic functions. However, the chiasmatic region has the greatest number of neurons, especially in the intermediate nucleus, but also in the suprachiasmatic nucleus and scattered throughout the medial and lateral preoptic regions. There was also robust labeling of neurons by the pre-PENK probe in the bed nucleus of the stria terminalis and in the central nucleus of the amygdala. **Several of these hypothalamic structures have been reported to be sexually dimorphic** [69]. High levels of PENK mRNA and PENK-derived peptides are expressed in **embryonic mesenchymal tissues during differentiation** into mature tissues and organs. Shortly after birth, as development progresses, PENK expression drops in those tissues to undetectable levels [70]. In the rat cardiac muscle, PENK was shown to rise five- to six-fold between 3 and 27 months of age. Elevated levels of peptide and mRNA in the aging heart suggest that enkephalins contribute to age-associated cardiac adaptations [71]. PENK is processed through the action of proprotein convertase (PC)1and PC2 to produce the enkephalin peptides, met-enkephalin and leu-enkephalin. Opioid peptides act on specific opiate receptors found on nervous and mucosal cells, and on various cells in the immune system. They link the neuroendocrine and immune systems to control immunological functions. The receptors for enkephalins include mu, delta and kappa opioid receptors, which are expressed on skin cells, and their activation can **regulate keratinocyte and melanocyte activities**. There is evidence that the endogenous opioids modulate **stress-induced effects**, including environmental stresses such as **solar radiation**, antigenic stimuli, and physical, chemical and biological insults. The skin responds to these insults through complex biological and biochemical activities, including PENK, which is coordinated by local neuroendocrine and immune systems. [72]. **Reactive oxygen species** (ROS) were shown to dramatically induce the expression of the opioid proenkephalin (PENK) in primary astrocytes [73]. Following **acute stress**, PENK transcription was shown to increase in the swine caudate putamen at the base of the forebrain [74]. It was detected as a **differentially methylated** gene by comparing **prenatally stressed** with non-stressed young Brahman bull calves [75]. Mild **maternal undernutrition** in early pregnancy was shown to induce greater levels of PENK mRNA in restricted fetuses [76]. *PENK* is a **Hedgehog**-responsive gene [77]. It belongs to the **opioid/orphanin gene family**, together with, prodynorphin, nociceptin/proorphanin and proopiomelanocortin, the latter being a complex precursor that comprises several peptidic hormones, including **melanocyte-stimulating hormones** (MSHs), adrenocorticotropic hormone (ACTH), and β-endorphin [78]. It has been included in the **Immediate-Early Genes** group, that includes genes for which the activation of transcription is fast (within minutes) and transient after a given cellular stimulus and undetectable in quiescent cells. Immediate-Early Genes do not require de novo protein synthesis to be activated and are grouped into ‘regulatory transcription factors’ and ‘effector factors. PENK expression was shown to be suppressed in **neural crest derived cells** by CaMK2 antagonist [79]. See Supplementary file S2 for the function of *CAMK2B*. The expression of PENK has been shown to be increased by pituitary adenylate cyclase activating peptide38 (PACAP38), i.e., a neuropeptide of the secretin-glucagon-VIP superfamily [80]. Also, PENK has been shown to be regulated by PKA, PKC and tyrosine kinases [81,82], and **glutamate receptors of the NMDA type** were involved in the maintenance of the expression of the *PENK* gene in neostriatal neurons [83] and in the neocortex [84]. PENK expression has been found to be increased in keratinocytes in **psoriatic skin**, suggesting a role for PENK in the pathogenesis of the skin disease [72]. PENK depletion substantially derepresses transcription of a range of antiapoptotic gene targets previously implicated in repression-mediated apoptosis induction by NF-κB and p53. It has been hence suggested to assist **stress-activated apoptosis** through transcriptional repression of **NF-kappaB- and p53-regulated gene targets** [85] and to be differentially expressed in cell lines with different sensitivity to a small organic molecule that has been shown to restore tumor-suppressor function to mutant p53 [86]. In osteosarcoma, PENK was shown to promote **cell migration** by regulating the phosphatidylinositol 3-kinase (**PI3K)/Akt** signaling pathway [87]. CpG sites in the PENK gene have been frequently reported in DNA methylation studies investigating **aging-associated changes** [88-91]. Evidence gathered from neurochemical and pharmacological studies point to an important role of opioid peptides in the balanced and/or coordinated activity of the brain striatal output pathways in pathological conditions such as **Parkinson’s disease** [92,93]. PENK expression in an *in vitro* model of **Huntington's disease**, a polyglutamine neurodegenerative disorder, comprised of primary striatal neurons expressing N-terminal fragments of mutant huntingtin reproduced the gene expression pattern seen in human Huntington's disease [94]. *PENK* was upregulated after **neurogenic induction** of bone marrow mesenchymal stomal cells and was involved in **synaptic transmission/long term potentialization** [95]. In cultured neocortical interneurons, endogenous **glutamate** enhanced N-methyl-D-aspartate-induced gene expression by acting via Group I metabotropic glutamate receptors. In contrast, stimulation of Group I metabotropic glutamate receptors by endogenous glutamate decreased the CREB phosphorylation and **PENK gene expression** induced by the activation of (S)-α-amino-3-hydroxy-5-methyl-4-isoxazolpropionic acid receptors [96]. **Steroids**, including **glucocorticoid**, the major mammalian response to **stress**, are associated with the regulation of the *PENK* gene. *PENK* gene expression was shown to be regulated by glucocorticoids via a mechanism that **varies with age** and **tissue** and **functions** during the induction of the Penk gene and not to maintain the basal gene expression [97]. In turn, PENK has been shown to represent a possible stimulatory input for ACTH release [98]. **Cortisol** has been suggested to play an important role in the regulation of fetal adrenal PENK mRNA levels and enkephalin synthesis by the adrenal gland of the fetal sheep [99]. Enkephalin levels are **responsive to gonadal steroids** [100]. *PENK* was identified among the genes that were significantly regulated during progesterone-induced endometrium maturation [101]. Proenkephalin mRNA was shown to be regulated by **17 beta-estradiol** in the endometrium and this effect of estradiol was antagonized by progesterone. Borthwick et al [102] and Carson et al [103] also found decreased endometrial expression of PENK during the secretory phase using microarray analysis. Differential expression of PDYN and PENK during the estrus cycle generally correlates with distinct differences in the degree of colocalization of **estrogen and progesterone receptors** mRNA in PDYN and PENK mRNA-containing neurons in the anteroventral periventricular nucleus of the preoptic region, which represents an essential component of neural pathways regulating gonadotropin secretion, and **contains sexually dimorphic populations of neurons** that express dynorphin or enkephalin [104]. All three major classes of endogenous opioid peptides have been implicated in **mediating the actions of estrogens and progesterone** on GnRH release. Sex- and steroid-associated differences in enkephalin neurons in the ovine paraventricular nucleus have been reported and suggest that opioid peptides may have an important role in the control of LH release. In rodents, enkephalin neurons have been shown to project to GnRH neurons in the preoptic area, further supporting a role for these neurons in the control of the reproductive axis [105]. The authors confirmed that the inhibitory actions of progesterone are associated with an increase in opioid gene expression in specific hypothalamic nuclei. An increase in progesterone was associated with increased POMC gene expression in the arcuate nucleus and increased PENK expression in the paraventricular nucleus. In addition to **androgen receptors** (ARs) and testosterone, opioid neuropeptides in the medial preoptic nucleus influence **sexually-motivated song production** in male European starlings, and it has been proposed that testosterone may in part regulate song by modifying opioid systems. Results support the hypothesis that **ARs may alter opioid gene expression** in medial preoptic nucleus to fine-tune singing to reflect a male's motivational state [106]. A substantial proportion of the PENK-expressing cells in the anterior part of the mice medial preoptic area were positive for estrogen receptor α and androgen receptor and may hence be **sensitive to gonadal steroid hormones** [107]. *PENK* was detected within a GWAS for birth weight in **indicine** (Nellore) cattle [59], for body measurement traits in Chinese Wagyu beef cattle [108]. In a whole-genome gene expression study that compared **prepubertal and postpubertal Brangus** (***Bos indicus***-influenced composite cattle) heifers, PENK was one of the most differentially expressed genes, with its expression being higher in post-puberty heifers in five tissues: uterus, endometrium, hypothalamus, pituitary gland, and live [109]. In sheep, increased progesterone was associated with increased POMC and PENK gene expression [105], with increased progesterone levels occurring in the transitional peripubertal phase before mature estrus cycles and fertility are achieved [110]. The PENK locus has been also detected in several GWAS-QTL efforts involving **Bos indicus-influenced** cattle in Australia and the United States [111,112]. This locus was also found to be a QTL in GWAS of bull fertility traits also indicative of **puberty** in Brahman bulls, which are a ***Bos indicus*** breed [111]. This gene was also reported to be differentially expressed in the pituitary gland of **Brangus** heifers [113,114]. Finally, PENK was validated in a tropical composite cattle population, as a marker for sexual precocity traits [115].

***LOC112449660*** Uncharacterized locus

***IMPAD1*** (Inositol Monophosphatase Domain-Containing Protein 1, alias 3'(2'), 5'-Bisphosphate Nucleotidase 2, *BPNT2,* alias *IMPase*) encodes a member of the inositol monophosphatase family. Is a Golgi-resident **phosphatase** responsible for the hydrolysis of phosphoadenosine phosphate (PAP), the byproduct of sulfotransferase reactions, to adenosine monophosphate (AMP). **Sulfur** is an essential element in all lifeforms used in the **synthesis of sulfur-containing amino acids**, **maintenance of cellular redox states** and detoxifying toxic compounds. 3'-phosphoadenosine 5'-phosphosulfate (PAPS) is the active form of sulfur used in these reactions, which consume PAPS, producing 3'-phosphoadenosine 5'-phosphate (PAP). PAP is degraded to 5′-AMP (AMP) by 3′-nucleotidase family. Mammals encode two 3′-nucleotidases, the Golgi-resident inositol monophosphatase 3 (IMPAD3 aka PAP phosphatase, gPAPP) and the cytosolic bisphosphate 3′-nucleotidase 1 (BPNT1, described in its own reac tion). Both require Mg2+ as cofactor and both are inhibited by lithium [116]. *BPNT2* drives the **sulfation of glycosaminoglycans**, which are then secreted from the Golgi and form the cartilaginous extracellular matrix. In humans, loss-of-function of the enzyme is associated with diseases involving bone deformities, and chondrodysplasia. *IMPAD1* inactivation in mice has been shown to produce chondrodysplasia with abnormal joint formation and impaired proteoglycan sulfation. The human chondrodysplasia associated with IMPAD1 deficiency joins a growing number of skeletoarticular conditions associated with **defective synthesis of sulfated proteoglycans**, highlighting the importance of proteoglycans in the development of skeletal elements and joints [117].

Glycosaminoglycans which are major constituents of the cell surface and extracellular matrix, but are also found in intracellular compartments, are believed to be important in modifying the growth of cells both in vitro and in vivo. These molecules may be involved in the adhesion of cells to each other and to the substratum or membranes. Several examples of the **role of glycosaminoglycans** (such as dermatan sulfate, keratan sulfate, chondroitin sulfate and heparan sulfate) **in pigment biology** **and pathology** are available in the literature, briefly and un-exhaustively addressed in what follows.

Dermatan sulfate has been shown to be required for **neural crest cells to migrate** and adhere to fibronectin [118]. A decline in the proportion of hyaluronic acid was found in **pigmented cultures obtained from neural crest cells**, possibly related to pigmentation. The overall pattern of glycosaminoglycans synthesis by **melanocytes** differentiated *in vitro* was relatively enriched in sulfated glycosaminoglycans, as is the environment that they colonize *in vivo* [119]. Whereas both **melanocyte and melanoma** cultures were shown to produce hyaluronic acid, chondroitin 4-sulfate, and heparan sulfates, with quantitative differences between the two cell cultures, only the melanocyte culture produced chondroitin 6-sulfate [120]. Melanoma cells were confirmed to present an altered glycosaminoglycan profile compared to that of melanocytes [121,122]. In metastatic melanoma, amount and composition of glycosaminoglycans are altered compared with nonmetastatic melanoma, indicating their involvement in **melanocytic tumor progression** [123,124]. Treatment of human metastatic melanoma cells with fibroblast growth factor-2 (FGF-2) was shown to strongly reduce the expression levels of the heparan sulfate-containing proteoglycan, **syndecan-4**, a focal adhesion component in a range of cell types, and to significantly enhance **migration** ability [125]. Cultured **keratinocytes** were shown to produce substantially more sulfated glycans than do the **melanocytes**; heparan sulfate was shown to be quantitatively the major sulfated glycosaminoglycan of keratinocytes and chondroitin sulfate the major product of melanocytes; during the transition from active growth to growth inhibition, heparan sulfates as proteoglycans and free chains increased in keratinocyte cultures, whereas no such transition was apparent for the melanocyte heparan sulfates; total production of chondroitin sulfate proteoglycans was down-regulated in both cell lineages with growth inhibition. The contrasts suggested that the synthetic patterns have evolved divergently as the two cell types exploited proteoglycans differently for regulating molecular pathways of growth and differentiation [126,127] cultured mouse **neural crest cells** in the presence of stem cell factor (SCF) in individual wells coated with extracellular matrix (fibronectin, collagen I, chondroitin sulphate, dermatan sulphate). More KIT-positive cells and DOPA-positive cells were detected in the presence of SCF on ECM-coated wells than on non-coated wells. A statistically significant increase in DOPA-positive cells was evident in fibronectin and collagen I wells. In contrast, in the absence of SCF, few DOPA-positive cells and KIT-positive cells were detected on either the ECM-coated or non-coated wells. The authors concluded that ECM affect melanocyte proliferation and development in the presence of SCF. Cell adhesion studies on micropatterned surfaces on silanised glass substrate, obtained through a photo-immobilisation procedure, highlighted that the sulphated hyaluronan derivative is capable to stimulate **melanocyte proliferation** when added to the culture medium more than the native hyaluronan polysaccharide. Melanocytes adhered on the sulphated hyaluronan derivative coated stripes while on the hyaluronan-coated micropatterned surfaces they spread around the structured area, resulting in the exclusion of the topographic pattern [128].

Takabe et al. [129] highlighted that melanocytes carry a thick and dynamic **hyaluronan coat** and that the **UVB-induced expression of proinflammatory mediators** in melanocytes depends on this coat. Removal of the pericellular hyaluronan coat by hyaluronidase further promotes the expression of the proinflammatory mediators in UVB-exposed melanocytes. The signaling pathways leading to the up-regulation of the studied cytokine and chemokines depends on the activation of TLR-4, p38, and **NF-kB**. Heparin, a highly sulfated glycosaminoglycan and an analogue of heparan sulfate chains of proteoglycans, frequently used for exploring the role of heparan sulfate in binding and interacting with various growth factors, chemokines, extracellular matrix proteins, and enzymes, was shown to **interfere with keratinocyte phagocytosis** through blocking **PI3k/Akt** and MEK/ERK signaling pathways, suggesting that heparin may serve as a potent antiphagocytic and anti-inflammation agent in epidermis [130].

In the context of **cancer**, *IMPAD1* undergoes mRNA upregulation and is co-amplified with oncogenes like MYC. Bajaj et al. [131] identified previously unknown functions of IMPAD1 in enhancing **Golgi-mediated secretion of matrix metalloproteases** (MMPs) to drive **cellular invasion and metastasis**. A role of IMPAD1 in promotion of lung cancer **metastasis** has been proposed by Yang et al. [132] who showed it mediated metastasis through **mitochondria dysfunction** by inhibiting mitochondrial Complex I activity to reduce **ROS production**, leading to AMPK phosphorylation, which in turn induced **Notch1**-mediated upregulation of HEY1. The gene expressions of IMPAD1 (gPAPP) and BPNT1 were shown to be remarkably down-regulated in **glioma**. IMPAD-1 hydrolyzes the Golgi PAP yielding AMP and phosphate, BPNT-1 hydrolyzes cytoplasm PAP to AMP and phosphate. Therefore, it is reasonable to speculate that the deficiencies of IMPAD1 and BPNT1 function would lead to a lower degradation rate of PAP and hence promote its accumulation in glioma [133]. *IMPAD1* was found in a **genome-wide association study** on body measurement traits in Chinese **Wagyu** beef cattle [108].

Inhibition of the *IMPAD1* **paralog**, *BPNT1*, in the nervous system, can cause **selective toxicity to specific neurons** [134]. In *Caenorhabditis elegans*, activity of XRN2, a conserved 5’→3’ exoribonuclease that recognizes RNA with a 5’ monophosphate and degrades it to mononucleotides, is promoted by BPNT1 through hydrolysis of the endogenous XRN inhibitor, PAP [135]. Mice lacking *BPNT1* develop **iron deficiency anemia**, due to accumulation of PAP. The anemia is rescued in *BPNT1* null mice harboring hypomorphic mutations in PAPSS2 (3'-Phosphoadenosine 5'-Phosphosulfate Synthase 2) causing decreased PAP metabolic toxicity [116,136]. In mice lacking BPNT1 at the level of the intestinal epithelium, tissue specific metabolic toxicity (elevated PAP levels in the duodenal epithelium) was observed, that cause **reduced expression of** **key iron homeostatic genes** involved in dietary iron reduction via duodenal cytochrome reductase, apical iron import, transcriptional coordination of iron regulatory genes, and iron export to the blood via **ferroportin**. Inhibition of intestinal BPNT1 was identified as a strategy to overcome **hemochromatosis** in mice. The study provided insights into the role of sulfur assimilation metabolism in mediating disorders of iron deficiency and overload in mice [137]. For a role of **iron in pigmentation**, see Supplementary file S1 (*OSGEPL1* and *SLC40A1* genes).

BPNT1 was found in a genomic region associated with **puberty traits** segregating across **tropically adapted cattle breeds** [115].

Other **paralogs** of this gene are *INPP1*, which encodes an inositol polyphosphate-1-phosphatase, one of the enzymes involved in **phosphatidylinositol signaling pathways**, *IMPA1*, which encodes the inositol monophosphatase 1, that dephosphorylates myo-inositol monophosphate to generate free myo-inositol, a **precursor of phosphatidylinositol**, and is therefore an important modulator of intracellular signal transduction via the production of the second messengers myoinositol 1,4,5-trisphosphate and diacylglycerol, and IMPA2, encoding the inositol monophosphatase 2, that catalyzes the dephosphoylration of inositol monophosphate and plays an important role in **phosphatidylinositol signaling**.

In the phosphatidylinositol signaling pathway, activated phospholipase C (PLC) by the binding extracellular signal molecule to the receptor on the cell surface catalyzes the hydrolysis of phosphatidylinositol-(4,5)-bisphosphate (PIP2) into two secondary messengers IP3 (inositol triphosphate) and (diacylglycerol) DAG, which both **convert extracellular signal into intracellular signal**. The derived phosphoinositides are involved in many signaling pathways such as the **PI3K-Akt pathway** that mediates **cell proliferation, survival, and metabolism**. In addition, phosphoinositide signaling also mediates many cell activities like cell **migration**, **endocytosis**, **membrane dynamics**.

**PI3K pathway and its effects on melanocytes** have been addressed in various studies [138]. In particular, Mosca et al. [138] showed that αMSH-dependent PI3K/AKT signaling play a key role in the **control of both melanogenesis and extracellular release of pigment**. In agreement with previous literature data, they showed that an alteration of this axis can lead to an **imbalance in redox equilibrium** and, consequently, interference with **genomic integrity**. These aspects become relevant in the control of cell survival.

***LOC107133116*** Uncharacterized locus

***TOX*** (Thymocyte Selection Associated High Mobility Group Box) encodes a protein that contains a HMG (high mobility group) box DNA binding domain. HMG boxes are found in many eukaryotic proteins involved in **chromatin assembly, transcription and replication**. It is generally thought that HMG proteins act as architectural factors that regulates gene expression by altering local chromatin structure and modulating the formation of multi-protein regulatory complexes. TOX is a **transcriptional regulator** with a major role in **neural stem cell commitment** and corticogenesis as well as in lymphoid cell development and lymphoid tissue organogenesis. TOX binds to GC-rich DNA sequences in the proximity of transcription start sites and may alter chromatin structure, modifying access of transcription factors to DNA. During cortical development, **controls the neural stem cell pool by inhibiting the switch from proliferative to differentiating progenitors**. Beyond progenitor cells, **promotes neurite outgrowth** in newborn neurons migrating to reach the cortical plate. May activate or repress critical genes for **neural stem cell fate** such as SOX2, EOMES and ROBO2. Acts as a **developmental checkpoint** and regulates thymocyte positive selection toward T cell lineage commitment. May regulate the **NOTCH-mediated gene program**. May regulate the expression of genes encoding inhibitory receptors such as PDCD1 and induce the **exhaustion program** (a reversible process), to prevent the overstimulation of T cells and activation-induced cell death.

During T cell development, expression of TOX has been shown to be sufficient to induce changes in coreceptor gene expression associated with β-selection. TOX expression was also shown to be sufficient to initiate positive selection to the CD8 lineage in the absence of MHC–TCR interactions [139]. The authors further demonstrated that up-regulation of TOX in double positive thymocytes is **calcineurin dependent**, linking this critical signaling pathway to nuclear changes during positive selection. Calcineurin (Cn) is a calcium-activated serine/threonine phosphatase composed of catalytic (A) and regulatory (B) subunits. Two catalytic subunits, CnAα and CnAβ, are expressed in developing thymocytes. It has been known for more than a decade that positive selection is inhibited by the immunosuppressive agent and potent Cn inhibitor cyclosporin A. More recently, genetic approaches have confirmed that Cn activation is required for positive selection. Although T cell development proceeds normally in mice that are deficient in CnAα, positive selection is inhibited in mice deficient in CnAβ. Conversely, expression of a constitutively active form of Cn in thymocytes has been reported to enhance positive selection. Activated Cn is known to bind and dephosphorylate NFATs in the cytoplasm, resulting in nuclear translocation of these transcriptional regulators. **Calcineurin inhibitors** (CIs) are recommended as first-line treatments in **vitiligo**. Pimecrolimus, is a topical calcineurin inhibitor. It has been shown to induce increased intracellular **tyrosinase activity**, which was consistent with the **elevated content of melanin**, to be effective in facilitating **melanocyte migration** and in increasing **MITF** protein expression thus enhancing melanin synthesis [140]. **Survival of melanocyte stem cells** has been shown to critically depends on B cell lymphoma 2 (BCL2). A basal calcineurin activity is required for maintaining BCL2 expression, and the activation of the calcineurin pathway has been shown to orchestrate the regulation of the intrinsic apoptosis pathway after antigen recognition. Therefore, calcineurin inhibitors might potentiate the pro-apoptotic effect of pharmacological BCL2 inhibitors. *In vitro*, a reduced BCL2 expression in **melanocytes** exposed to **calcineurin inhibitors** increased their sensitivity to the small molecule BCL2 inhibitor ABT-737. This correlated with an augmented pro-apoptotic activity of ABT-737 on melanocytes in combination with cyclosporine A. ABT-737 induced a **fur depigmentation** at the site of injection, and this effect was expanded to a generalized depigmentation in combination with cyclosporine A. Thus, inhibiting calcineurin increases the pro-apoptotic potency of ABT-737 in cells depending on BCL2 for survival [141]. **Melanoma cells** treated with a selective inhibitor for calcineurin/NFATsignaling were reported to show **increased melanin content and tyrosinase activity** [142]. Several reports have revealed the prominent **role of the calcineurin-NFAT2 pathway in melanoma progression and melanogenesis** [143-145]. Regulation of **keratinocyte differentiation** by calcineurin has been reported [146,147].

A high expression of TOX has been reported in **hypopigmented mycosis fungoides**, a variant of cutaneous T‑cell lymphoma [148].

Finally, *TOX* was detected within a selection signature **in cattle with various indicine introgression levels** [10] as well as in a QTL in Korean native Hanwoo cattle for carcass quality traits [149].

***TRNAC-GCA*** (transfer RNA cysteine) tRNA

**REFERENCES**

1. Suzuki, J.; Imanishi, E.; Nagata, S., Exposure of phosphatidylserine by Xk-related protein family members during apoptosis. *J Biol Chem* **2014,** *289* (44), 30257-67.

2. Suzuki, J.; Imanishi, E.; Nagata, S., Xkr8 phospholipid scrambling complex in apoptotic phosphatidylserine exposure. *Proc Natl Acad Sci U S A* **2016,** *113* (34), 9509-14.

3. Sakuragi, T.; Kosako, H.; Nagata, S., Phosphorylation-mediated activation of mouse Xkr8 scramblase for phosphatidylserine exposure. *Proc Natl Acad Sci U S A* **2019,** *116* (8), 2907-2912.

4. Yamashita, Y.; Suzuki, C.; Uchiyama, Y.; Nagata, S., Infertility Caused by Inefficient Apoptotic Germ Cell Clearance in Xkr8-Deficient Male Mice. *Mol Cell Biol* **2020,** *40* (3).

5. Kawano, M.; Nagata, S., Lupus-like autoimmune disease caused by a lack of Xkr8, a caspase-dependent phospholipid scramblase. *Proc Natl Acad Sci U S A* **2018,** *115* (9), 2132-2137.

6. Kim, G. W.; Nam, G. H.; Kim, I. S.; Park, S. Y., Xk-related protein 8 regulates myoblast differentiation and survival. *FEBS J* **2017,** *284* (21), 3575-3588.

7. Chavas, L. M.; Ihara, K.; Kawasaki, M.; Torii, S.; Uejima, T.; Kato, R.; Izumi, T.; Wakatsuki, S., Elucidation of Rab27 recruitment by its effectors: structure of Rab27a bound to Exophilin4/Slp2-a. *Structure* **2008,** *16* (10), 1468-77.

8. Edea, Z.; Jung, K. S.; Shin, S. S.; Yoo, S. W.; Choi, J. W.; Kim, K. S., Signatures of positive selection underlying beef production traits in Korean cattle breeds. *J Anim Sci Technol* **2020,** *62* (3), 293-305.

9. Porto Neto, L. R.; Bunch, R. J.; Harrison, B. E.; Barendse, W., Variation in the XKR4 gene was significantly associated with subcutaneous rump fat thickness in indicine and composite cattle. *Anim Genet* **2012,** *43* (6), 785-9.

10. Cheruiyot, E. K.; Bett, R. C.; Amimo, J. O.; Zhang, Y.; Mrode, R.; Mujibi, F. D. N., Signatures of Selection in Admixed Dairy Cattle in Tanzania. *Front Genet* **2018,** *9*, 607.

11. Sun, J.; Thingholm, T.; Hojrup, P.; Ronnstrand, L., XK-related protein 5 (XKR5) is a novel negative regulator of KIT/D816V-mediated transformation. *Oncogenesis* **2018,** *7* (6), 48.

12. Zhang, D.; Xia, J., Somatic synonymous mutations in regulatory elements contribute to the genetic aetiology of melanoma. *BMC Med Genomics* **2020,** *13* (Suppl 5), 43.

13. Cifola, I.; Pietrelli, A.; Consolandi, C.; Severgnini, M.; Mangano, E.; Russo, V.; De Bellis, G.; Battaglia, C., Comprehensive genomic characterization of cutaneous malignant melanoma cell lines derived from metastatic lesions by whole-exome sequencing and SNP array profiling. *PLoS One* **2013,** *8* (5), e63597.

14. Li, K. G.; Yin, R. X.; Huang, F.; Chen, W. X.; Wu, J. Z.; Cao, X. L., XKR6 rs7014968 SNP Increases Serum Total Cholesterol Levels and the Risk of Coronary Heart Disease and Ischemic Stroke. *Clin Appl Thromb Hemost* **2020,** *26*, 1076029620902844.

15. Zhu, X.; Cho, E. S.; Sha, Q.; Peng, J.; Oksov, Y.; Kam, S. Y.; Ho, M.; Walker, R. H.; Lee, S., Giant axon formation in mice lacking Kell, XK, or Kell and XK: animal models of McLeod neuroacanthocytosis syndrome. *Am J Pathol* **2014,** *184* (3), 800-7.

16. Chang, P.; Heier, C.; Qin, W.; Han, L.; Huang, F.; Sun, Q., Molecular identification of transmembrane protein 68 as an endoplasmic reticulum-anchored and brain-specific protein. *PLoS One* **2017,** *12* (5), e0176980.

17. Melo, T. P.; Fortes, M. R. S.; Bresolin, T.; Mota, L. F. M.; Albuquerque, L. G.; Carvalheiro, R., Multitrait meta-analysis identified genomic regions associated with sexual precocity in tropical beef cattle. *J Anim Sci* **2018,** *96* (10), 4087-4099.

18. Terakado, A. P. N.; Costa, R. B.; de Camargo, G. M. F.; Irano, N.; Bresolin, T.; Takada, L.; Carvalho, C. V. D.; Oliveira, H. N.; Carvalheiro, R.; Baldi, F.; de Albuquerque, L. G., Genome-wide association study for growth traits in Nelore cattle. *Animal* **2018,** *12* (7), 1358-1362.

19. Monecke, T.; Dickmanns, A.; Strasser, A.; Ficner, R., Structure analysis of the conserved methyltransferase domain of human trimethylguanosine synthase TGS1. *Acta Crystallogr D Biol Crystallogr* **2009,** *65* (Pt 4), 332-8.

20. Borg, R. M.; Fenech Salerno, B.; Vassallo, N.; Bordonne, R.; Cauchi, R. J., Disruption of snRNP biogenesis factors Tgs1 and pICln induces phenotypes that mirror aspects of SMN-Gemins complex perturbation in Drosophila, providing new insights into spinal muscular atrophy. *Neurobiol Dis* **2016,** *94*, 245-58.

21. Zhang, Q.; Meng, X.; Qin, G.; Xue, X.; Dang, N., Lyn Kinase Promotes the Proliferation of Malignant Melanoma Cells through Inhibition of Apoptosis and Autophagy via the PI3K/Akt Signaling Pathway. *J Cancer* **2019,** *10* (5), 1197-1208.

22. Su, R.; Zhang, J., Oncogenic role of LYN in human gastric cancer via the Wnt/beta-catenin and AKT/mTOR pathways. *Exp Ther Med* **2020,** *20* (1), 646-654.

23. Palacios-Moreno, J.; Foltz, L.; Guo, A.; Stokes, M. P.; Kuehn, E. D.; George, L.; Comb, M.; Grimes, M. L., Neuroblastoma tyrosine kinase signaling networks involve FYN and LYN in endosomes and lipid rafts. *PLoS Comput Biol* **2015,** *11* (4), e1004130.

24. Shivakrupa, R.; Linnekin, D., Lyn contributes to regulation of multiple Kit-dependent signaling pathways in murine bone marrow mast cells. *Cell Signal* **2005,** *17* (1), 103-9.

25. Xu, Y.; He, Q.; Lu, Y.; Tao, F.; Zhao, L.; Ou, R., MicroRNA-218-5p inhibits cell growth and metastasis in cervical cancer via LYN/NF-kappaB signaling pathway. *Cancer Cell Int* **2018,** *18*, 198.

26. Toubiana, J.; Rossi, A. L.; Belaidouni, N.; Grimaldi, D.; Pene, F.; Chafey, P.; Comba, B.; Camoin, L.; Bismuth, G.; Claessens, Y. E.; Mira, J. P.; Chiche, J. D., Src-family-tyrosine kinase Lyn is critical for TLR2-mediated NF-kappaB activation through the PI 3-kinase signaling pathway. *Innate Immun* **2015,** *21* (7), 685-97.

27. Napolitani, G.; Bortoletto, N.; Racioppi, L.; Lanzavecchia, A.; D'Oro, U., Activation of src-family tyrosine kinases by LPS regulates cytokine production in dendritic cells by controlling AP-1 formation. *Eur J Immunol* **2003,** *33* (10), 2832-41.

28. Morinaga, T.; Abe, K.; Nakayama, Y.; Yamaguchi, N.; Yamaguchi, N., Activation of Lyn tyrosine kinase through decreased membrane cholesterol levels during a change in its membrane distribution upon cell detachment. *J Biol Chem* **2014,** *289* (38), 26327-43.

29. Morinaga, T.; Yamaguchi, N.; Nakayama, Y.; Tagawa, M.; Yamaguchi, N., Role of Membrane Cholesterol Levels in Activation of Lyn upon Cell Detachment. *Int J Mol Sci* **2018,** *19* (6).

30. Vidalain, P. O.; Azocar, O.; Servet-Delprat, C.; Rabourdin-Combe, C.; Gerlier, D.; Manie, S., CD40 signaling in human dendritic cells is initiated within membrane rafts. *EMBO J* **2000,** *19* (13), 3304-13.

31. Iwabuchi, K.; Prinetti, A.; Sonnino, S.; Mauri, L.; Kobayashi, T.; Ishii, K.; Kaga, N.; Murayama, K.; Kurihara, H.; Nakayama, H.; Yoshizaki, F.; Takamori, K.; Ogawa, H.; Nagaoka, I., Involvement of very long fatty acid-containing lactosylceramide in lactosylceramide-mediated superoxide generation and migration in neutrophils. *Glycoconj J* **2008,** *25* (4), 357-74.

32. Wimalachandra, D.; Yang, J. X.; Zhu, L.; Tan, E.; Asada, H.; Chan, J. Y. K.; Lee, Y. H., Long-chain glucosylceramides crosstalk with LYN mediates endometrial cell migration. *Biochim Biophys Acta Mol Cell Biol Lipids* **2018,** *1863* (1), 71-80.

33. Luciano, F.; Ricci, J. E.; Auberger, P., Cleavage of Fyn and Lyn in their N-terminal unique regions during induction of apoptosis: a new mechanism for Src kinase regulation. *Oncogene* **2001,** *20* (36), 4935-41.

34. Marchetti, S.; Gamas, P.; Belhacene, N.; Grosso, S.; Pradelli, L. A.; Colosetti, P.; Johansen, C.; Iversen, L.; Deckert, M.; Luciano, F.; Hofman, P.; Ortonne, N.; Khemis, A.; Mari, B.; Ortonne, J. P.; Ricci, J. E.; Auberger, P., The caspase-cleaved form of LYN mediates a psoriasis-like inflammatory syndrome in mice. *EMBO J* **2009,** *28* (16), 2449-60.

35. Luciano, F.; Herrant, M.; Jacquel, A.; Ricci, J. E.; Auberger, P., The p54 cleaved form of the tyrosine kinase Lyn generated by caspases during BCR-induced cell death in B lymphoma acts as a negative regulator of apoptosis. *FASEB J* **2003,** *17* (6), 711-3.

36. Chudakova, D. A.; Zeidan, Y. H.; Wheeler, B. W.; Yu, J.; Novgorodov, S. A.; Kindy, M. S.; Hannun, Y. A.; Gudz, T. I., Integrin-associated Lyn kinase promotes cell survival by suppressing acid sphingomyelinase activity. *J Biol Chem* **2008,** *283* (43), 28806-16.

37. Choi, Y. L.; Bocanegra, M.; Kwon, M. J.; Shin, Y. K.; Nam, S. J.; Yang, J. H.; Kao, J.; Godwin, A. K.; Pollack, J. R., LYN is a mediator of epithelial-mesenchymal transition and a target of dasatinib in breast cancer. *Cancer Res* **2010,** *70* (6), 2296-306.

38. Liang, X.; He, X.; Li, Y.; Wang, J.; Wu, D.; Yuan, X.; Wang, X.; Li, G., Lyn regulates epithelial-mesenchymal transition in CS-exposed model through Smad2/3 signaling. *Respir Res* **2019,** *20* (1), 201.

39. Yang, C.; Maruyama, S.; Yanagi, S.; Wang, X.; Takata, M.; Kurosaki, T.; Yamamura, H., Syk and Lyn are involved in radiation-induced signaling, but inactivation of Syk or Lyn alone is not sufficient to prevent radiation-induced apoptosis. *J Biochem* **1995,** *118* (1), 33-8.

40. Shangary, S.; Lerner, E. C.; Zhan, Q.; Corey, S. J.; Smithgall, T. E.; Baskaran, R., Lyn regulates the cell death response to ultraviolet radiation through c-Jun N terminal kinase-dependent Fas ligand activation. *Exp Cell Res* **2003,** *289* (1), 67-76.

41. Grishin, A. V.; Azhipa, O.; Semenov, I.; Corey, S. J., Interaction between growth arrest-DNA damage protein 34 and Src kinase Lyn negatively regulates genotoxic apoptosis. *Proc Natl Acad Sci U S A* **2001,** *98* (18), 10172-7.

42. Matsuda, D.; Nakayama, Y.; Horimoto, S.; Kuga, T.; Ikeda, K.; Kasahara, K.; Yamaguchi, N., Involvement of Golgi-associated Lyn tyrosine kinase in the translocation of annexin II to the endoplasmic reticulum under oxidative stress. *Exp Cell Res* **2006,** *312* (7), 1205-17.

43. Helmke, S.; Pfenninger, K. H., Growth cone enrichment and cytoskeletal association of non-receptor tyrosine kinases. *Cell Motil Cytoskeleton* **1995,** *30* (3), 194-207.

44. Dombrosky-Ferlan, P.; Grishin, A.; Botelho, R. J.; Sampson, M.; Wang, L.; Rudert, W. A.; Grinstein, S.; Corey, S. J., Felic (CIP4b), a novel binding partner with the Src kinase Lyn and Cdc42, localizes to the phagocytic cup. *Blood* **2003,** *101* (7), 2804-9.

45. Liu, W. M.; Huang, P.; Kar, N.; Burgett, M.; Muller-Greven, G.; Nowacki, A. S.; Distelhorst, C. W.; Lathia, J. D.; Rich, J. N.; Kappes, J. C.; Gladson, C. L., Lyn facilitates glioblastoma cell survival under conditions of nutrient deprivation by promoting autophagy. *PLoS One* **2013,** *8* (8), e70804.

46. Namba, T.; Kibe, Y.; Funahashi, Y.; Nakamuta, S.; Takano, T.; Ueno, T.; Shimada, A.; Kozawa, S.; Okamoto, M.; Shimoda, Y.; Oda, K.; Wada, Y.; Masuda, T.; Sakakibara, A.; Igarashi, M.; Miyata, T.; Faivre-Sarrailh, C.; Takeuchi, K.; Kaibuchi, K., Pioneering axons regulate neuronal polarization in the developing cerebral cortex. *Neuron* **2014,** *81* (4), 814-29.

47. Umemori, H.; Ogura, H.; Tozawa, N.; Mikoshiba, K.; Nishizumi, H.; Yamamoto, T., Impairment of N-methyl-D-aspartate receptor-controlled motor activity in LYN-deficient mice. *Neuroscience* **2003,** *118* (3), 709-13.

48. Schwarz, L. J.; Fox, E. M.; Balko, J. M.; Garrett, J. T.; Kuba, M. G.; Estrada, M. V.; Gonzalez-Angulo, A. M.; Mills, G. B.; Red-Brewer, M.; Mayer, I. A.; Abramson, V.; Rizzo, M.; Kelley, M. C.; Meszoely, I. M.; Arteaga, C. L., LYN-activating mutations mediate antiestrogen resistance in estrogen receptor-positive breast cancer. *J Clin Invest* **2014,** *124* (12), 5490-502.

49. Gavali, S.; Gupta, M. K.; Daswani, B.; Wani, M. R.; Sirdeshmukh, R.; Khatkhatay, M. I., LYN, a key mediator in estrogen-dependent suppression of osteoclast differentiation, survival, and function. *Biochim Biophys Acta Mol Basis Dis* **2019,** *1865* (3), 547-557.

50. Zardan, A.; Nip, K. M.; Thaper, D.; Toren, P.; Vahid, S.; Beraldi, E.; Fazli, L.; Lamoureux, F.; Gust, K. M.; Cox, M. E.; Bishop, J. L.; Zoubeidi, A., Lyn tyrosine kinase regulates androgen receptor expression and activity in castrate-resistant prostate cancer. *Oncogenesis* **2014,** *3*, e115.

51. Banci, L.; Bertini, I.; Ciofi-Baffoni, S.; Jaiswal, D.; Neri, S.; Peruzzini, R.; Winkelmann, J., Structural characterization of CHCHD5 and CHCHD7: two atypical human twin CX9C proteins. *J Struct Biol* **2012,** *180* (1), 190-200.

52. Barros, M. H.; Johnson, A.; Tzagoloff, A., COX23, a homologue of COX17, is required for cytochrome oxidase assembly. *J Biol Chem* **2004,** *279* (30), 31943-7.

53. Leary, S. C., Redox regulation of SCO protein function: controlling copper at a mitochondrial crossroad. *Antioxid Redox Signal* **2010,** *13* (9), 1403-16.

54. Fink, T.; Tiplady, K.; Lopdell, T.; Johnson, T.; Snell, R. G.; Spelman, R. J.; Davis, S. R.; Littlejohn, M. D., Functional confirmation of PLAG1 as the candidate causative gene underlying major pleiotropic effects on body weight and milk characteristics. *Sci Rep* **2017,** *7*, 44793.

55. Karim, L.; Takeda, H.; Lin, L.; Druet, T.; Arias, J. A.; Baurain, D.; Cambisano, N.; Davis, S. R.; Farnir, F.; Grisart, B.; Harris, B. L.; Keehan, M. D.; Littlejohn, M. D.; Spelman, R. J.; Georges, M.; Coppieters, W., Variants modulating the expression of a chromosome domain encompassing PLAG1 influence bovine stature. *Nat Genet* **2011,** *43* (5), 405-13.

56. Zhang, R.; Miao, J.; Song, Y.; Zhang, W.; Xu, L.; Chen, Y.; Zhang, L.; Gao, H.; Zhu, B.; Li, J.; Gao, X., Genome-wide association study identifies the PLAG1-OXR1 region on BTA14 for carcass meat yield in cattle. *Physiol Genomics* **2019,** *51* (5), 137-144.

57. Nishimura, S.; Watanabe, T.; Mizoshita, K.; Tatsuda, K.; Fujita, T.; Watanabe, N.; Sugimoto, Y.; Takasuga, A., Genome-wide association study identified three major QTL for carcass weight including the PLAG1-CHCHD7 QTN for stature in Japanese Black cattle. *BMC Genet* **2012,** *13*, 40.

58. Hoshiba, H.; Setoguchi, K.; Watanabe, T.; Kinoshita, A.; Mizoshita, K.; Sugimoto, Y.; Takasuga, A., Comparison of the effects explained by variations in the bovine PLAG1 and NCAPG genes on daily body weight gain, linear skeletal measurements and carcass traits in Japanese Black steers from a progeny testing program. *Anim Sci J* **2013,** *84* (7), 529-34.

59. Utsunomiya, Y. T.; do Carmo, A. S.; Carvalheiro, R.; Neves, H. H.; Matos, M. C.; Zavarez, L. B.; Perez O'Brien, A. M.; Solkner, J.; McEwan, J. C.; Cole, J. B.; Van Tassell, C. P.; Schenkel, F. S.; da Silva, M. V.; Porto Neto, L. R.; Sonstegard, T. S.; Garcia, J. F., Genome-wide association study for birth weight in Nellore cattle points to previously described orthologous genes affecting human and bovine height. *BMC Genet* **2013,** *14*, 52.

60. Song, Y.; Xu, L.; Chen, Y.; Zhang, L.; Gao, H.; Zhu, B.; Niu, H.; Zhang, W.; Xia, J.; Gao, X.; Li, J., Genome-Wide Association Study Reveals the PLAG1 Gene for Knuckle, Biceps and Shank Weight in Simmental Beef Cattle. *PLoS One* **2016,** *11* (12), e0168316.

61. Taye, M.; Yoon, J.; Dessie, T.; Cho, S.; Oh, S. J.; Lee, H. K.; Kim, H., Deciphering signature of selection affecting beef quality traits in Angus cattle. *Genes Genomics* **2018,** *40* (1), 63-75.

62. Adams, M. K.; Lee, S. A.; Belyaeva, O. V.; Wu, L.; Kedishvili, N. Y., Characterization of human short chain dehydrogenase/reductase SDR16C family members related to retinol dehydrogenase 10. *Chem Biol Interact* **2017,** *276*, 88-94.

63. Mei, X.; Wu, Z.; Huang, J.; Sun, Y.; Shi, W., Screening and analysis of differentially expressed genes of human melanocytes in skin cells mixed culture. *Am J Transl Res* **2019,** *11* (5), 2657-2667.

64. Wu, L.; Belyaeva, O. V.; Adams, M. K.; Klyuyeva, A. V.; Lee, S. A.; Goggans, K. R.; Kesterson, R. A.; Popov, K. M.; Kedishvili, N. Y., Mice lacking the epidermal retinol dehydrogenases SDR16C5 and SDR16C6 display accelerated hair growth and enlarged meibomian glands. *J Biol Chem* **2019,** *294* (45), 17060-17074.

65. Epping, M. T.; Wang, L.; Edel, M. J.; Carlee, L.; Hernandez, M.; Bernards, R., The human tumor antigen PRAME is a dominant repressor of retinoic acid receptor signaling. *Cell* **2005,** *122* (6), 835-47.

66. Takebayashi-Suzuki, K.; Suzuki, A., Intracellular Communication among Morphogen Signaling Pathways during Vertebrate Body Plan Formation. *Genes (Basel)* **2020,** *11* (3).

67. Lu, Z.; Xie, Y.; Huang, H.; Jiang, K.; Zhou, B.; Wang, F.; Chen, T., Hair follicle stem cells regulate retinoid metabolism to maintain the self-renewal niche for melanocyte stem cells. *Elife* **2020,** *9*.

68. Kawakami, T.; Ohgushi, A.; Hirobe, T.; Soma, Y., Analysis of the effects of all-trans retinoic acid on human melanocytes and melanoblasts in vitro. *J Dermatol* **2017,** *44* (1), 93-94.

69. Sukhov, R. R.; Walker, L. C.; Rance, N. E.; Price, D. L.; Young, W. S., 3rd, Opioid precursor gene expression in the human hypothalamus. *J Comp Neurol* **1995,** *353* (4), 604-22.

70. Rosen, H.; Krichevsky, A.; Polakiewicz, R. D.; Benzakine, S.; Bar-Shavit, Z., Developmental regulation of proenkephalin gene expression in osteoblasts. *Mol Endocrinol* **1995,** *9* (11), 1621-31.

71. Caffrey, J. L.; Boluyt, M. O.; Younes, A.; Barron, B. A.; O'Neill, L.; Crow, M. T.; Lakatta, E. G., Aging, cardiac proenkephalin mRNA and enkephalin peptides in the Fisher 344 rat. *J Mol Cell Cardiol* **1994,** *26* (6), 701-11.

72. Nagui, N. A.; Ezzat, M. A.; Abdel Raheem, H. M.; Rashed, L. A.; Abozaid, N. A., Possible role of proenkephalin in psoriasis. *Clin Exp Dermatol* **2016,** *41* (2), 124-8.

73. Rosenberger, J.; Petrovics, G.; Buzas, B., Oxidative stress induces proorphanin FQ and proenkephalin gene expression in astrocytes through p38- and ERK-MAP kinases and NF-kB. *J Neurochemistry* **2001,** *7*9, 35-44.

74. Vellucci, S. V.; Parrott, R. F., Vasopressin and oxytocin gene expression in the porcine forebrain under basal conditions and following acute stress. *Neuropeptides* **1997,** *31* (5), 431-8.

75. Littlejohn, B. P.; Price, D. M.; Neuendorff, D. A.; Carroll, J. A.; Vann, R. C.; Riggs, P. K.; Riley, D. G.; Long, C. R.; Randel, R. D.; Welsh, T. H., Influence of prenatal transportation stress-induced differential DNA methylation on the physiological control of behavior and stress response in suckling Brahman bull calves. *J Anim Sci* **2020,** *98* (1).

76. Hawkins, P.; Hanson, M.A.; Matthews, S.G., Maternal undernutrition in early gestation alters molecular regulation of the hypothalamic-pituotary-adrenal axis b in the ovine fetus. *J Neuroendocrinol.* **2001** 13 (10), 855-861.

77. Lu, Y.; Starkey, N.; Lei, W.; Li, J.; Cheng, J.; Folk, W. R.; Lubahn, D. B., Inhibition of Hedgehog-Signaling Driven Genes in Prostate Cancer Cells by Sutherlandia frutescens Extract. *PLoS One* **2015,** *10* (12), e0145507.

78. Navarro, S.; Soletto, L.; Puchol, S.; Rotllant, J.; Soengas, J. L.; Cerda-Reverter, J. M., 60 YEARS OF POMC: POMC: an evolutionary perspective. *J Mol Endocrinol* **2016,** *56* (4), T113-8.

79. Yang, X.; Zhu, Z.; Ding, X.; Wang, X.; Cui, G.; Hua, F.; Xiang, J., CaMKII inhibition ameliorated levodopa-induced dyskinesia by downregulating tyrosine hydroxylase activity in an experimental model of Parkinson's disease. *Brain Res* **2018,** *1687*, 66-73.

80. Monnier, D.; Loeffler, J. P., Pituitary adenylate cyclase-activating polypeptide stimulates proenkephalin gene transcription through AP1- and CREB-dependent mechanisms. *DNA Cell Biol* **1998,** *17* (2), 151-9.

81. Just, L.; Morl, F.; Barmann, C.; Olenik, C.; Meyer, D. K., Evidence for cell specific regulation by PACAP38 of the proenkephalin gene expression in neocortical cells. *Glia* **2000,** *30* (3), 242-52.

82. Theodoridu, A.; Olenik, C.; Boeckh, C.; Ziefer, P.; Gebicke-Harter, P.; Meyer, D. K., Interaction of protein kinases A and C in their effects on the proenkephalin gene in astroglial cells. *Neurochem Int* **1994,** *25* (4), 385-93.

83. Just, L.; Olenik, C.; Meyer, D. K., Neocortical projections regulate the neostriatal proenkephalin gene expression. *Cereb Cortex* **1999,** *9* (4), 332-9.

84. Just, L.; Olenik, C.; Heimrich, B.; Meyer, D. K., Glutamatergic control of the expression of the proenkephalin gene in rat frontoparietal cortical slice cultures. *Cereb Cortex* **1998,** *8* (8), 702-9.

85. McTavish, N.; Copeland, L. A.; Saville, M. K.; Perkins, N. D.; Spruce, B. A., Proenkephalin assists stress-activated apoptosis through transcriptional repression of NF-kappaB- and p53-regulated gene targets. *Cell Death Differ* **2007,** *14* (9), 1700-10.

86. Aryee, D. N.; Niedan, S.; Ban, J.; Schwentner, R.; Muehlbacher, K.; Kauer, M.; Kofler, R.; Kovar, H., Variability in functional p53 reactivation by PRIMA-1(Met)/APR-246 in Ewing sarcoma. *Br J Cancer* **2013,** *109* (10), 2696-704.

87. Zhang, H. P.; Yu, Z. L.; Wu, B. B.; Sun, F. R., PENK inhibits osteosarcoma cell migration by activating the PI3K/Akt signaling pathway. *J Orthop Surg Res* **2020,** *15* (1), 162.

88. Giuliani, C.; Cilli, E.; Bacalini, M. G.; Pirazzini, C.; Sazzini, M.; Gruppioni, G.; Franceschi, C.; Garagnani, P.; Luiselli, D., Inferring chronological age from DNA methylation patterns of human teeth. *Am J Phys Anthropol* **2016,** *159* (4), 585-95.

89. Kananen, L.; Marttila, S.; Nevalainen, T.; Jylhava, J.; Mononen, N.; Kahonen, M.; Raitakari, O. T.; Lehtimaki, T.; Hurme, M., Aging-associated DNA methylation changes in middle-aged individuals: the Young Finns study. *BMC Genomics* **2016,** *17*, 103.

90. Steegenga, W. T.; Boekschoten, M. V.; Lute, C.; Hooiveld, G. J.; de Groot, P. J.; Morris, T. J.; Teschendorff, A. E.; Butcher, L. M.; Beck, S.; Muller, M., Genome-wide age-related changes in DNA methylation and gene expression in human PBMCs. *Age (Dordr)* **2014,** *36* (3), 9648.

91. Garagnani, P.; Bacalini, M. G.; Pirazzini, C.; Gori, D.; Giuliani, C.; Mari, D.; Di Blasio, A. M.; Gentilini, D.; Vitale, G.; Collino, S.; Rezzi, S.; Castellani, G.; Capri, M.; Salvioli, S.; Franceschi, C., Methylation of ELOVL2 gene as a new epigenetic marker of age. *Aging Cell* **2012,** *11* (6), 1132-4.

92. Bissonnette, S.; Vaillancourt, M.; Hebert, S. S.; Drolet, G.; Samadi, P., Striatal pre-enkephalin overexpression improves Huntington's disease symptoms in the R6/2 mouse model of Huntington's disease. *PLoS One* **2013,** *8* (9), e75099.

93. Samadi, P.; Bedard, P. J.; Rouillard, C., Opioids and motor complications in Parkinson's disease. *Trends Pharmacol Sci* **2006,** *27* (10), 512-7.

94. Runne, H.; Regulier, E.; Kuhn, A.; Zala, D.; Gokce, O.; Perrin, V.; Sick, B.; Aebischer, P.; Deglon, N.; Luthi-Carter, R., Dysregulation of gene expression in primary neuron models of Huntington's disease shows that polyglutamine-related effects on the striatal transcriptome may not be dependent on brain circuitry. *J Neurosci* **2008,** *28* (39), 9723-31.

95. Tondreau, T.; Dejeneffe, M.; Meuleman, N.; Stamatopoulos, B.; Delforge, A.; Martiat, P.; Bron, D.; Lagneaux, L., Gene expression pattern of functional neuronal cells derived from human bone marrow mesenchymal stromal cells. *BMC Genomics* **2008,** *9*, 166.

96. Lindemeyer, K.; Leemhuis, J.; Loffler, S.; Grass, N.; Norenberg, W.; Meyer, D. K., Metabotropic glutamate receptors modulate the NMDA- and AMPA-induced gene expression in neocortical interneurons. *Cereb Cortex* **2006,** *16* (11), 1662-77.

97. Franklin, S. O.; Jimenez, R., Increases in preproenkephalin mRNA levels in the Syrian hamster: the influence of glucocorticoids is dependent on age and tissue. *Brain Res* **2006,** *1086* (1), 65-75.

98. Hawkins, P.; Hanson, M. A.; Matthews, S. G., Maternal undernutrition in early gestation alters molecular regulation of the hypothalamic-pituitary-adrenal axis in the ovine fetus. *J Neuroendocrinol* **2001,** *13* (10), 855-61.

99. Fraser, M.; Matthews, S. G.; Braems, G.; Jeffray, T.; Challis, J. R., Developmental regulation of preproenkephalin (PENK) gene expression in the adrenal gland of the ovine fetus and newborn lamb: effects of hypoxemia and exogenous cortisol infusion. *J Endocrinol* **1997,** *155* (1), 143-9.

100. Slamberova, R.; Hnatczuk, O. C.; Vathy, I., Expression of proopiomelanocortin and proenkephalin mRNA in sexually dimorphic brain regions are altered in adult male and female rats treated prenatally with morphine. *J Pept Res* **2008,** *63* (5), 399-408.

101. Ace, C. I.; Okulicz, W. C., Microarray profiling of progesterone-regulated endometrial genes during the rhesus monkey secretory phase. *Reprod Biol Endocrinol* **2004,** *2*, 54.

102. Borthwick, J. M.; Charnock-Jones, D. S.; Tom, B. D.; Hull, M. L.; Teirney, R.; Phillips, S. C.; Smith, S. K., Determination of the transcript profile of human endometrium. *Mol Hum Reprod* **2003,** *9* (1), 19-33.

103. Carson, D. D.; Lagow, E.; Thathiah, A.; Al-Shami, R.; Farach-Carson, M. C.; Vernon, M.; Yuan, L.; Fritz, M. A.; Lessey, B., Changes in gene expression during the early to mid-luteal (receptive phase) transition in human endometrium detected by high-density microarray screening. *Mol Hum Reprod* **2002,** *8* (9), 871-9.

104. Simerly, R. B.; Young, B. J.; Carr, A. M., Co-expression of steroid hormone receptors in opioid peptide-containing neurons correlates with patterns of gene expression during the estrous cycle. *Brain Res Mol Brain Res* **1996,** *40* (2), 275-84.

105. Taylor, J. A.; Goubillon, M. L.; Broad, K. D.; Robinson, J. E., Steroid control of gonadotropin-releasing hormone secretion: associated changes in pro-opiomelanocortin and preproenkephalin messenger RNA expression in the ovine hypothalamus. *Biol Reprod* **2007,** *76* (3), 524-31.

106. Cordes, M. A.; Stevenson, S. A.; Driessen, T. M.; Eisinger, B. E.; Riters, L. V., Sexually-motivated song is predicted by androgen-and opioid-related gene expression in the medial preoptic nucleus of male European starlings (Sturnus vulgaris). *Behav Brain Res* **2015,** *278*, 12-20.

107. Tsuneoka, Y.; Yoshida, S.; Takase, K.; Oda, S.; Kuroda, M.; Funato, H., Neurotransmitters and neuropeptides in gonadal steroid receptor-expressing cells in medial preoptic area subregions of the male mouse. *Sci Rep* **2017,** *7* (1), 9809.

108. An, B.; Xia, J.; Chang, T.; Wang, X.; Xu, L.; Zhang, L.; Gao, X.; Chen, Y.; Li, J.; Gao, H., Genome-wide association study reveals candidate genes associated with body measurement traits in Chinese Wagyu beef cattle. *Anim Genet* **2019,** *50* (4), 386-390.

109. Canovas, A.; Reverter, A.; DeAtley, K. L.; Ashley, R. L.; Colgrave, M. L.; Fortes, M. R.; Islas-Trejo, A.; Lehnert, S.; Porto-Neto, L.; Rincon, G.; Silver, G. A.; Snelling, W. M.; Medrano, J. F.; Thomas, M. G., Multi-tissue omics analyses reveal molecular regulatory networks for puberty in composite beef cattle. *PLoS One* **2014,** *9* (7), e102551.

110. Fortes, M. R.; Nguyen, L. T.; Porto Neto, L. R.; Reverter, A.; Moore, S. S.; Lehnert, S. A.; Thomas, M. G., Polymorphisms and genes associated with puberty in heifers. *Theriogenology* **2016,** *86* (1), 333-9.

111. Fortes, M. R.; Reverter, A.; Hawken, R. J.; Bolormaa, S.; Lehnert, S. A., Candidate genes associated with testicular development, sperm quality, and hormone levels of inhibin, luteinizing hormone, and insulin-like growth factor 1 in Brahman bulls. *Biol Reprod* **2012,** *87* (3), 58.

112. Peters, S. O.; Kizilkaya, K.; Garrick, D. J.; Fernando, R. L.; Reecy, J. M.; Weaber, R. L.; Silver, G. A.; Thomas, M. G., Heritability and Bayesian genome-wide association study of first service conception and pregnancy in Brangus heifers. *J Anim Sci* **2013,** *91* (2), 605-12.

113. DeAtley, K. L.; Colgrave, M. L.; Canovas, A.; Wijffels, G.; Ashley, R. L.; Silver, G. A.; Rincon, G.; Medrano, J. F.; Islas-Trejo, A.; Fortes, M. R. S.; Reverter, A.; Porto-Neto, L.; Lehnert, S. A.; Thomas, M. G., Neuropeptidome of the Hypothalamus and Pituitary Gland of Indicine x Taurine Heifers: Evidence of Differential Neuropeptide Processing in the Pituitary Gland before and after Puberty. *J Proteome Res* **2018,** *17* (5), 1852-1865.

114. Dias, M. M.; Canovas, A.; Mantilla-Rojas, C.; Riley, D. G.; Luna-Nevarez, P.; Coleman, S. J.; Speidel, S. E.; Enns, R. M.; Islas-Trejo, A.; Medrano, J. F.; Moore, S. S.; Fortes, M. R.; Nguyen, L. T.; Venus, B.; Diaz, I. S.; Souza, F. R.; Fonseca, L. F.; Baldi, F.; Albuquerque, L. G.; Thomas, M. G.; Oliveira, H. N., SNP detection using RNA-sequences of candidate genes associated with puberty in cattle. *Genet Mol Res* **2017,** *16* (1).

115. Melo, T. P.; Fortes, M. R. S.; Fernandes Junior, G. A.; Albuquerque, L. G.; Carvalheiro, R., RAPID COMMUNICATION: Multi-breed validation study unraveled genomic regions associated with puberty traits segregating across tropically adapted breeds1. *J Anim Sci* **2019,** *97* (7), 3027-3033.

116. Hudson, B. H.; Frederick, J. P.; Drake, L. Y.; Megosh, L. C.; Irving, R. P.; York, J. D., Role for cytoplasmic nucleotide hydrolysis in hepatic function and protein synthesis. *Proc Natl Acad Sci U S A* **2013,** *110* (13), 5040-5.

117. Vissers, L. E.; Lausch, E.; Unger, S.; Campos-Xavier, A. B.; Gilissen, C.; Rossi, A.; Del Rosario, M.; Venselaar, H.; Knoll, U.; Nampoothiri, S.; Nair, M.; Spranger, J.; Brunner, H. G.; Bonafe, L.; Veltman, J. A.; Zabel, B.; Superti-Furga, A., Chondrodysplasia and abnormal joint development associated with mutations in IMPAD1, encoding the Golgi-resident nucleotide phosphatase, gPAPP. *Am J Hum Genet* **2011,** *88* (5), 608-15.

118. Gouignard, N.; Maccarana, M.; Strate, I.; von Stedingk, K.; Malmstrom, A.; Pera, E. M., Musculocontractural Ehlers-Danlos syndrome and neurocristopathies: dermatan sulfate is required for Xenopus neural crest cells to migrate and adhere to fibronectin. *Dis Model Mech* **2016,** *9* (6), 607-20.

119. Glimelius, B.; Pintar, J. E., Analysis of developmentally homogeneous neural crest cell populations in vitro. IV. Cell proliferation and synthesis of glycosaminoglycans. *Cell Differ* **1981,** *10* (3), 173-82.

120. Bhavanandan, V. P., Glycosaminoglycans of cultured human fetal uveal melanocytes and comparison with those produced by cultured human melanoma cells. *Biochemistry* **1981,** *20* (19), 5595-602.

121. Oba-Shinjo, S. M.; Correa, M.; Ricca, T. I.; Molognoni, F.; Pinhal, M. A.; Neves, I. A.; Marie, S. K.; Sampaio, L. O.; Nader, H. B.; Chammas, R.; Jasiulionis, M. G., Melanocyte transformation associated with substrate adhesion impediment. *Neoplasia* **2006,** *8* (3), 231-41.

122. Sifaki, M.; Assouti, M.; Nikitovic, D.; Krasagakis, K.; Karamanos, N. K.; Tzanakakis, G. N., Lumican, a small leucine-rich proteoglycan substituted with keratan sulfate chains is expressed and secreted by human melanoma cells and not normal melanocytes. *IUBMB Life* **2006,** *58* (10), 606-10.

123. Ma, Y. Q.; Geng, J. G., Heparan sulfate-like proteoglycans mediate adhesion of human malignant melanoma A375 cells to P-selectin under flow. *J Immunol* **2000,** *165* (1), 558-65.

124. Smetsers, T. F.; van de Westerlo, E. M.; ten Dam, G. B.; Clarijs, R.; Versteeg, E. M.; van Geloof, W. L.; Veerkamp, J. H.; van Muijen, G. N.; van Kuppevelt, T. H., Localization and characterization of melanoma-associated glycosaminoglycans: differential expression of chondroitin and heparan sulfate epitopes in melanoma. *Cancer Res* **2003,** *63* (11), 2965-70.

125. Chalkiadaki, G.; Nikitovic, D.; Berdiaki, A.; Sifaki, M.; Krasagakis, K.; Katonis, P.; Karamanos, N. K.; Tzanakakis, G. N., Fibroblast growth factor-2 modulates melanoma adhesion and migration through a syndecan-4-dependent mechanism. *Int J Biochem Cell Biol* **2009,** *41* (6), 1323-31.

126. Piepkorn, M.; Hovingh, P.; Dillberger, A.; Linker, A., Divergent regulation of proteoglycan and glycosaminoglycan free chain expression in human keratinocytes and melanocytes. *In Vitro Cell Dev Biol Anim* **1995,** *31* (7), 536-41.

127. Takano, N.; Kawakami, T.; Kawa, Y.; Asano, M.; Watabe, H.; Ito, M.; Soma, Y.; Kubota, Y.; Mizoguchi, M., Fibronectin combined with stem cell factor plays an important role in melanocyte proliferation, differentiation and migration in cultured mouse neural crest cells. *Pigment Cell Res* **2002,** *15* (3), 192-200.

128. Barbucci, R.; Magnani, A.; Lamponi, S.; Pasqui, D.; Bryan, S., The use of hyaluronan and its sulphated derivative patterned with micrometric scale on glass substrate in melanocyte cell behaviour. *Biomaterials* **2003,** *24* (6), 915-26.

129. Takabe, P.; Karna, R.; Rauhala, L.; Tammi, M.; Tammi, R.; Pasonen-Seppanen, S., Melanocyte Hyaluronan Coat Fragmentation Enhances the UVB-Induced TLR-4 Receptor Signaling and Expression of Proinflammatory Mediators IL6, IL8, CXCL1, and CXCL10 via NF-kappaB Activation. *J Invest Dermatol* **2019,** *139* (9), 1993-2003 e4.

130. Makino-Okamura, C.; Niki, Y.; Takeuchi, S.; Nishigori, C.; Declercq, L.; Yaroch, D. B.; Saito, N., Heparin inhibits melanosome uptake and inflammatory response coupled with phagocytosis through blocking PI3k/Akt and MEK/ERK signaling pathways in human epidermal keratinocytes. *Pigment Cell Melanoma Res* **2014,** *27* (6), 1063-74.

131. Bajaj, R.; Kundu, S. T.; Grzeskowiak, C. L.; Fradette, J. J.; Scott, K. L.; Creighton, C. J.; Gibbons, D. L., IMPAD1 and KDELR2 drive invasion and metastasis by enhancing Golgi-mediated secretion. *Oncogene* **2020**.

132. Yang, Y. F.; Wang, Y. Y.; Hsiao, M.; Lo, S.; Chang, Y. C.; Jan, Y. H.; Lai, T. C.; Lee, Y. C.; Hsieh, Y. C.; Yuan, S. F., IMPAD1 functions as mitochondrial electron transport inhibitor that prevents ROS production and promotes lung cancer metastasis through the AMPK-Notch1-HEY1 pathway. *Cancer Lett* **2020,** *485*, 27-37.

133. Li, W.; Jia, H.; Li, Q.; Cui, J.; Li, R.; Zou, Z.; Hong, X., Glycerophosphatidylcholine PC(36:1) absence and 3'-phosphoadenylate (pAp) accumulation are hallmarks of the human glioma metabolome. *Sci Rep* **2018,** *8* (1), 14783.

134. Meisel, J. D.; Kim, D. H., Inhibition of Lithium-Sensitive Phosphatase BPNT-1 Causes Selective Neuronal Dysfunction in C. elegans. *Curr Biol* **2016,** *26* (14), 1922-8.

135. Miki, T. S.; Carl, S. H.; Stadler, M. B.; Grosshans, H., XRN2 Autoregulation and Control of Polycistronic Gene Expresssion in Caenorhabditis elegans. *PLoS Genet* **2016,** *12* (9), e1006313.

136. Hudson, B. H.; Hale, A. T.; Irving, R. P.; Li, S.; York, J. D., Modulation of intestinal sulfur assimilation metabolism regulates iron homeostasis. *Proc Natl Acad Sci U S A* **2018,** *115* (12), 3000-3005.

137. Hale, A. T.; Brown, R. E.; Luka, Z.; Hudson, B. H.; Matta, P.; Williams, C. S.; York, J. D., Modulation of sulfur assimilation metabolic toxicity overcomes anemia and hemochromatosis in mice. *Adv Biol Regul* **2020,** *76*, 100694.

138. Mosca, S.; Cardinali, G.; Flori, E.; Briganti, S.; Bottillo, I.; Mileo, A. M.; Maresca, V., The PI3K pathway induced by alphaMSH exerts a negative feedback on melanogenesis and contributes to the release of pigment. *Pigment Cell Melanoma Res* **2020**.

139. Wilkinson, B.; Chen, J. Y.; Han, P.; Rufner, K. M.; Goularte, O. D.; Kaye, J., TOX: an HMG box protein implicated in the regulation of thymocyte selection. *Nat Immunol* **2002,** *3* (3), 272-80.

140. Xu, P.; Chen, J.; Tan, C.; Lai, R. S.; Min, Z. S., Pimecrolimus increases the melanogenesis and migration of melanocytes in vitro. *Korean J Physiol Pharmacol* **2017,** *21* (3), 287-292.

141. Cippa, P. E.; Kraus, A. K.; Lindenmeyer, M. T.; Chen, J.; Guimezanes, A.; Bardwell, P. D.; Wekerle, T.; Wuthrich, R. P.; Fehr, T., Resistance to ABT-737 in activated T lymphocytes: molecular mechanisms and reversibility by inhibition of the calcineurin-NFAT pathway. *Cell Death Dis* **2012,** *3*, e299.

142. Lee, Y. S.; Kim, D. W.; Kim, S.; Choi, H. I.; Lee, Y.; Kim, C. D.; Lee, J. H.; Lee, S. D.; Lee, Y. H., Downregulation of NFAT2 promotes melanogenesis in B16 melanoma cells. *Anat Cell Biol* **2010,** *43* (4), 303-9.

143. Flockhart, R. J.; Armstrong, J. L.; Reynolds, N. J.; Lovat, P. E., NFAT signalling is a novel target of oncogenic BRAF in metastatic melanoma. *Br J Cancer* **2009,** *101* (8), 1448-55.

144. Perotti, V.; Baldassari, P.; Bersani, I.; Molla, A.; Vegetti, C.; Tassi, E.; Dal Col, J.; Dolcetti, R.; Anichini, A.; Mortarini, R., NFATc2 is a potential therapeutic target in human melanoma. *J Invest Dermatol* **2012,** *132* (11), 2652-60.

145. Yang, Y.; Guo, W.; Ma, J.; Xu, P.; Zhang, W.; Guo, S.; Liu, L.; Ma, J.; Shi, Q.; Jian, Z.; Liu, L.; Wang, G.; Gao, T.; Han, Z.; Li, C., Downregulated TRPV1 Expression Contributes to Melanoma Growth via the Calcineurin-ATF3-p53 Pathway. *J Invest Dermatol* **2018,** *138* (10), 2205-2215.

146. Santini, M. P.; Talora, C.; Seki, T.; Bolgan, L.; Dotto, G. P., Cross talk among calcineurin, Sp1/Sp3, and NFAT in control of p21(WAF1/CIP1) expression in keratinocyte differentiation. *Proc Natl Acad Sci U S A* **2001,** *98* (17), 9575-80.

147. Mammucari, C.; Tommasi di Vignano, A.; Sharov, A. A.; Neilson, J.; Havrda, M. C.; Roop, D. R.; Botchkarev, V. A.; Crabtree, G. R.; Dotto, G. P., Integration of Notch 1 and calcineurin/NFAT signaling pathways in keratinocyte growth and differentiation control. *Dev Cell* **2005,** *8* (5), 665-76.

148. Ibrahim, M. A.; Mohamed, A.; Soltan, M. Y., Thymocyte selection-associated high-mobility group box as a potential diagnostic marker differentiating hypopigmented mycosis fungoides from early vitiligo: A pilot study. *Indian J Dermatol Venereol Leprol* **2019**.

149. Li, Y.; Kim, J. J., Multiple Linkage Disequilibrium Mapping Methods to Validate Additive Quantitative Trait Loci in Korean Native Cattle (Hanwoo). *Asian-Australas J Anim Sci* **2015,** *28* (7), 926-35.
