# Supplementary material for: Fifteen Shades of Grey: Combined Analysis of Genome-Wide SNP Data in Steppe and Mediterranean Grey Cattle Sheds New Light on the Molecular Basis of Coat Color
Source: Genes (Basel). 2020 Aug 13;11(8):932. doi: 10.3390/genes11080932 (PMC7464420; doi:10.3390/genes11080932)
Supplement: Supplementary file 1 [file genes-11-00932-s001.zip › Supplementary file S4.docx]

**Supplementary file S4.** Functions of genes in the ± 250 kbp region upstream and downstream the locus ARS-BFGL-NGS-11271 on BTA26, detected as significant in 13 and 2 pair-wise contrasts performed using Holstein and Angus as reference breeds, respectively.

***LDB1*** (LIM Domain Binding 1 alias CLIM2) is a multi-adaptor protein that mediates interactions between different classes of transcription factors and their co-regulators, and the nature of these complexes determines **cell fate and differentiation**, as well as **cell migration** [1]. There is increasing evidence that LIM-domain proteins and LDB family members can exert both positive and negative regulatory influences on transcription [2]. In mice, it is ubiquitously expressed during development, and a global knockout of *LDB1* caused mid-gestation lethality with severe defects, such as loss of the heart and anterior head. Tissue-specific deletions using a conditional knockout allele revealed that it is important in **neural patterning and development**, hematopoietic system, and limbs at later stages, and that its function in the neural crest-derived palatal mesenchyme is essential for normal morphogenesis of the secondary palate [3]. Studies have demonstrated the role of LIM homeodomain transcription complexes in **maintenance of stem cells** and/or **early progenitors** of several somatic tissues [4,5]. A critical requirement for LDB1 in thymocyte **self-renewal**, as well as thymocyte **radiation resistance**, was reported [6]. In the small intestine, cells prominently expressing LDB1 were **quiescent**, possibly representing a group of **stem-like cells** [7]. A role of *LHX2*, a member of LIM homeodomain transcription complexes, in **specification and maintenance of the hair follicle stem cells**[8-10], in **hair formation** [11] as well as its role as a key factor integrating signaling and transcriptional networks that modulate **activity of the hair follicle stem cells during epidermal regeneration after injury** was demonstrated [12]. LHX2 has been shown to interact with the product of the melanocyte-specific gene-related gene 1 (***MRG1*** alias *CITED2*) [13], a cytokine inducible gene with transcription activation and transformation functions, whose expression appears to be mediated, at least in part, by the **JAK/STAT signaling** pathway [14]. Its paralog, *CITED1* (alias *MSG1*) has been associated with pigmentation of melanocytes and melanoma [15-17] and hypothesized to act as a **regulator of MITF** [18]. Moreover, *CITED1* was found among the best candidate genes falling within one of the QTLs (Quantitative Trait Loci) identified in a F1 hybrid mice population chosen because exhibiting a particularly low level of transgene-mediated ectopically pigmented melanocyte stem cells. This trait was of interest because melanocyte stem cells that are undergoing differentiation produce visible ectopic pigmentation when hairs are observed by light microscopy, and stem cell differentiation is the cellular mechanism responsible for hair graying in these mice. Melanocyte stem cell differentiation indeed precedes hair graying and is positively associated with hair graying severity. Differentiated melanocyte stem cell have been observed also in humans, and their number increases with age, which makes melanocyte stem cell differentiation a relevant cellular phenotype to evaluate for genetic loci that may modify the extent of **age-related hair graying** in both mouse and humans [19]. A prominent role of LDB1 in **Wnt signaling** has been described in several articles[20]. A role of LDB1 complexes in mechanisms of long-range interactions by transcription factors has been also proposed [21,22]. LDB1/CLIM2 has been shown to **interact with estrogen receptors** [23] and to be involved in the regulation of the biological activity of estrogen receptors during the development of human breast cancer [24]. Güngör et al. [25] indicated that the proteasome targets for degradation LIM protein complexes that are not associated with protecting cofactors, and that LDB/CLIM proteins stabilize LIM homeodomain proteins **preventing binding of ubiquitin ligases**. Interestingly, Vaman et al. [26] identified LASP1, a LIM protein, as a yet unknown protein in melanocytes and as novel partner of dynamin in the complex process of **melanosome vesicle release at the dendrite tips**. Bi-allelic loss-of-function variants of the murine LMX1A gene, a LIM homeobox transcription factor, have been reported to cause, among other clinical signs, also **pigmentation abnormalities** [27,28] such as those observed in *dreher* mice [29], showing pigmentation defects in the trunk region varying from a **white spot or patch in the ventral area** to a complete white belt surrounding the whole trunk, or in the *mtl* and *bsd* mice, showing **white belly patches** of variable size [28]. Another LIM domain protein, Hic‐5 (alias ARA55, androgen receptor associated protein55), functions as a focal adhesion adaptor protein as well as a coactivator for steroid receptors such as androgen receptors, glucocorticoid receptors and progesterone receptors [30], may promote either cell senescence or cell growt [31], and has been shown to be involved in melanoma proliferation both in vitro and in vivo[32]. Also worth of interest the fact that knockdown of another LIM domain containing protein, PRICKLE2, in zebrafish caused **shorter antero-posterior axis** during gastrulation and r**etinal neurogenesis defects**, **disrupted morphogenesis of a transient ciliated organ** (anche bb7 fa lo stesso) and **shortened cilia**. (anche bb7 fa lo stesso). Individual knockdown of this gene did not show a retrograde melanosome transport delay in zebrafish. However, the combined knockdown of this gene and of BBS7, a fundamental member of the hetero-octameric BBSome complex, **suppressed the BBS7-induced retrograde melanosome transport delay**, one of the cardinal features of the impairment of the BBSome function [33]. Indeed, the BBSome acts as a cargo adapter in the intra-cellular transport machinery, and mutations at BBS genes have been associated to a severe ciliopathy, the **Bardet-Biedl syndrome** (BBS), characterized, among other features, by pigmentary retinopathy. On the contrary, while BBS7 knockdown did not alter the rate of anterograde melanosome transport [34], a **delay in anterograde melanosome transport** was observed in PRICKLE2-knockdown zebrafish larvae [33]. Alike PRICKLE2 knockdown, BBS7 knockdown was associated with disrupted morphogenesis of a transient ciliated organ and shortened cilia [33]. These results suggest a novel role for PRICKLE2 in intracellular transport, and further support the hypothesis that *BBS7* and *PRICKLE2* modulate distinct pathways leading to partly overlapping phenotypes. The PRICKLE1 LIM domain containing protein was shown to be required in zebrafish for **epithelial-to-mesenchymal transition** of pre-migratory **neural crest cells** and **migration** of cranial neural crest cells [35]. Taken together, the above studies highlight the relevance of LIM-domain proteins and their interactors in pigmentation.

***PPRC1*** (PPARG Related Coactivator 1, alias PGC-1) encodes a protein that it is thought to be a functional relative of PPAR-gamma coactivator 1, which activates **mitochondrial biogenesis** in response to proliferative signals and increased cellular ATP demand [36] playing a central role in integrating mitochondrial biogenesis and energy production with many diverse cellular functions [37]. Interestingly, a close **link between melanosomes and mitochondria** has been highlighted, characterized by **physical connections** between these organelles **through fibrillar bridges** [38]. These contacts are **associated to the melanogenesis process**, as indicated by the fact that they are reduced in a model of aberrant melanogenesis whereas they are enhanced both where melanosome biogenesis takes place in the perinuclear area and when it is actively stimulated by OA1, a G protein-coupled receptor implicated in **ocular albinism** and organellogenesis. In another study, Zhang et al. [39] showed that the disruption of the Ca^2+^ exchanger activity of the mitochondrial NCKX5 protein compromises melanosomal Ca^2+^ homeostasis, melanosomal PMEL expression and melanin production. The gene encoding the NCKX5 protein is *SLC24A5* which has been previously implicated in pigmentation in zebrafish and humans [40] and identified as a causative gene for a nonsyndromic type of OCA, designated as OCA6 [41]. Thus, the authors defined a new class of **hypopigmentation attributable to dysfunctional mitochondria** and to an impairment of mitochondrial Ca^2+^ transfer into melanosomes. Zhang et al. [39] also observed that pharmacological inhibition of mitochondrial ATP synthesis, or of the Ca^2+^ exchanger activity of the mitochondrial NCKX protein, severely reduced mitochondrion–melanosome contact formation. Thus, mitochondrion–melanosome contact sites may have functions in exchanging Ca^2+^ and other molecules such as ATP, required for melanosome biogenesis or melanin biosynthesis. Based on the knowledge that reactive oxygen species (ROS)-induced damage has been suggested as a possible mechanism for graying of hair with aging, and that mitochondrial function is impaired in vitiligo melanocytes [42,43], the authors hypothesized that mitochondrial function could have a role in the **graying of hair** in older people and formation of **hypopigmented lesions in vitiligo** patients. Moreover, inhibitors of the mitochondrial ATPase complex were found to induce pigmentation in melanocytes, further indicating a **role for mitochondrial signal transduction in the induction of melanin production** [44].

***LOC112444554*** Uncharacterized locus

***NOLC1*** (Nucleolar and Coiled-Body Phosphoprotein 1, alias P130) encodes a member of the so-called “pocket proteins”, a family of three tumor suppressor proteins. Their inactivation by cyclin-dependent kinases liberates E2F transcription factors from suppressive complexes that, in turn, induces the continuous expression of target genes whose products promote cell cycle progression. In **normal melanocytes**, external growth factors suppress the activity of all three pocket proteins, allowing E2F activity to accumulate and **sustain transcription of target genes required for cell proliferation**. In **melanoma cells** from advanced lesions, all three pocket proteins are highly phosphorylated and inactive, even in the absence of environmental mitogens, and free E2F activity is **constitutively high** [45-47].

***LOC112444524*** Uncharacterized locus

***LOC101902227*** (vacuolar-sorting protein SNF8 pseudogene)

***LOC785229*** (sulfatase-modifying factor 1 pseudogene)

***ELOVL3*** (ELOVL Fatty Acid Elongase 3) encodes a protein that catalyzes the first and rate-limiting reaction of the four reactions that constitute the long-chain fatty acids elongation cycle. This endoplasmic reticulum-bound enzymatic process allows the addition of 2 carbons to the chain of long- and very long-chain fatty acids per cycle. It is a condensing enzyme that exhibits activity toward saturated and monounsaturated acyl-CoA substrates, with the highest activity towards C22:0 acyl-CoA. May participate in the production of both saturated and monounsaturated very long-chain fatty acids of different chain lengths, that are involved in multiple biological processes as precursors for synthesis of sphingolipids and ceramides and of membrane lipids and lipid mediators. Ceramides and their complex derivatives are important components of mammalian membranes and are key players in intracellular signaling, involved in apoptosis, cell senescence, proliferation, cell growth and differentiation [48]. Ceramide constitutes the lipid backbone of sphingomyelin and other sphingolipids. Sphingolipids are essential for cell proliferation. Ceramide-induced inactivation of Akt/PKB has been associated with **reduced cell proliferation** in a **melanocyte** cell line, as well as **reduced cell pigmentation** by **inhibition of the tyrosinase activity** [49]. The importance of free long chain fatty acids in the regulation of **pigmentation and melanogenesis** has been shown [50]. Linoleic acid or plamitic acid have been shown to **regulate tyrosinase activity** via posttranscriptional events, including the modulation of tyrosinase ubiquitination. In addition, saturated very long chain fatty acids are suggested to be important for the tight packaging of membrane lipids in so-called raft structures, which may have a function as organizers in **intracellular transport** and **signaling** events by creating micro-environments [51,52]. Changes in acyl-chain length of membrane lipid constituents, due to variations in elongase activity, affect membrane-residing enzyme activities, as well as **membrane fusion/budding** events [53], susceptibility to apoptosis [54,55]. *ELOVL3* has been implicated in the **de-differentiation** of primary hepatocytes accompanied with reorganization of lipid metabolism [56], and in the **de-differentiation** in a spheroid-induced **epithelial-mesenchymal transition** model accompanied with poor outcome in breast cancer patients [57]. *ELOVL3* mRNA levels were shown to display diurnal rhythmic changes exclusively in adult male mouse livers, hence in a **sexually dimorphic** manner. Castration markedly reduced *ELOVL3* expression levels in male mouse livers but did not disrupt circadian variation of ELOVL3. Injection of female mice with 5α-dihydrotestosterone induced *ELOVL3* rhythmicity in the liver. In conclusion, *ELOVL3* expression is affected by alteration of the **androgen signaling** [58]. In addition, *ELOVL3*, together with *LDB1* (see above) and *NFKB2* (see below) was located on a putatively selected region in the semi-feral Sasi-Ardi sheep breed from Western Pyrenees, characterized by a **diluted red phenotype** (“cream wool and a fawn face”; “uniform blonde or reddish color”) [59]. *ELOVL3* was significantly **up-regulated in gray coated goats** compared to white and to brown coated goats [60]. Its expression differed significantly between anagen and catagen in hair follicles from five Italian cashmere goats [61]. Together with other members of the elongase family, it was found to be overexpressed in the fine-wool Super Merino sheep compared to the coarse wool Small Tail Han (STH) sheep [62], and it was suggested as a molecular marker for the content of lanolin in fleeces. Other members of the fatty acid elongase family were shown to be **stimulated by 17-β-estradiol** in human breast cancer cell lines [63]. *De novo* mutation in *ELOVL1* was reported to cause, among other symptoms, also acanthosis nigricans, a skin disorder characterized by **darkening (hyperpigmentation**) and thickening (hyperkeratosis) **of the skin**, occurring mainly in the folds of the skin in the armpit, groin and back of the neck.

***PITX3*** (Paired-Like Homeodomain Transcription Factor 3) encodes a member of the RIEG/PITX homeobox family. It is a transcriptional regulator important for terminal **differentiation** and subset-specification of meso-diencephalic dopaminergic (mdDA) **neurons** during development, controlling the size and **rostral/caudal**-identity of the mdDA neuronal population [64]. In addition to its importance during development, it also has roles in the **long-term survival** and maintenance of the mdDA neurons. The Mouse Genome Informatics database (http://www.informatics.jax.org/) reports an annotation for this gene concerning “**abnormal** iris **pigmentation**”. Fusion of this gene with *ETV6* have been associated with the occurrence of a spitzoid neoplasms, the Pigmented spindle cell nevus (PSCN) of Reed [65], characterized by **heavily pigmented** lesional cells and keratinocytes containing abundant **coarse melanin granules** [66]. In mice, *ELOVL3* (see above), another ELOVL member, *ELOVL1*, and *PITX* presents a peculiar tail-to-tail arrangement [67].

***GBF1*** (Golgi Brefeldin A Resistant Guanine Nucleotide Exchange Factor) encodes a member of the Sec7 domain family. The encoded protein is a **guanine nucleotide exchange factor** that regulates the recruitment of proteins to membranes by mediating GDP to GTP exchange. It is localized to the Golgi apparatus and plays a role in **vesicular trafficking** [68-73]. It is involved in the COPI **vesicle-dependent retrograde transport** to the endoplasmic reticulum. The activity of GBF1 is necessary for the Golgi export of ciliary cargo in retinal photoreceptors [74] and is part of the **axonal trafficking** machinery in peripheral axons [75].

***NFKB2*** (Nuclear Factor Kappa B Subunit 2) encodes a subunit of the transcription factor complex nuclear factor-kappa-B (NF-kB) which is a pleiotropic transcription factor present in almost all cell types and is the endpoint of a series of signal transduction events that are initiated by a vast array of stimuli related to many biological processes such as inflammation, immunity, differentiation, cell growth, tumorigenesis and apoptosis. NF-kB was identified as a potential “master regulator” of **melanoma invasion** [76] and, in human melanoma, a number of NF-κB-regulated chemokines are constitutively expressed at high levels [77]. *In vitro* and *in vivo* studies have shown that NF-κB activity is upregulated in dysplastic nevi and lesions of human melanoma when compared with human nevi or melanocytes in normal skin [78-80]. NF-kB was shown to contribute to the promoter activity of the MIA (**melanoma inhibitory activity**) protein in melanoma cells where it exerts autologous growth inhibitory effects [81]. Notably, NF-kB2 has been shown to be the master transcription factor involved in the control of *EZH2* expression in melanoma, with *EZH2* being increasingly expressed during the progression of a broad range of cancers, including **melanoma**, and has been correlated with poor prognosis. *EZH2* can also suppress the **senescence program**, which blocks cellular proliferation and acts as a potent barrier to tumorigenesis [82]. Down-regulation in the activity of the NF-κB pathway, associated with decreased **proliferation, migration** and colony formation, was observed in **melanoma** cell lines with ectopic stable expression of miR-377, which is normally expressed in melanocytes but not in melanoma cell lines [83]. NF-kB modulates Endothelin-1 [84] which has been shown to play a role in the arsenic-mediated **hyperpigmentation** in mice skin [85]. Dey-Rao and Sinha [86] identified, through interactome analysis, NF-kB as one of the key blood- associated transcriptional factors potentially affecting **vitiligo** pathogenesis. Kim et al. [87] showed that tumor necrosis factor superfamily member 14 (*TNFSF14*) inhibits melanogenesis in the primary culture of human **epidermal melanocytes**, where it also activates NF-κB signaling. They provided a link between the two phenomena, demonstrating that inhibition of NF-kB effectively blocks the **hypopigmentation** induced by TNFSF14. Studies have shown that NF-κB signaling may be involved in both the **activation and the inhibition of melanogenesis** [88-90].

***PSD*** (Pleckstrin And Sec7 Domain Containing) encodes a Plekstrin homology and Sec7 domains-containing protein that functions as a **guanine nucleotide exchange factor** (see *GBF1*, above). The encoded protein regulates signal transduction by activating ADP-ribosylation factor 6 (encoded by the gene ***ARF6***). During development of the skin, the canonical **Wnt/β-catenin signaling pathway** guides the **migration and expansion of neural crest-derived melanocytes**. β-catenin signaling determines the fate of melanoblasts through the activation of the micropthalmia transcription factor, ***MITF*** [91]. Grossmann et al. [92] demonstrated that the small guanosine triphosphatase ***ARF6*** plays a critical role in the activation and nuclear translocation of β-catenin. In addition, they showed that ARF6 is necessary for **invasion in melanoma cells**, consistently with the previous findings that stabilized β-catenin promotes metastasis of melanoma. In a study investigating the role of circadian clock and light exposure in **retinal** **pigment regeneration**, it was found, together with its paralogs *PSD2*, *PSD3* and *PSD4*, to be differentially expressed when mice were exposed to daily cycles of different light to dark ratio [93]. Its paralog *PSD3* was also found to be differentially expressed in a comparison of the transcriptional profiles of **melanocytes from dark and light skinned** individuals under basal conditions and following ultraviolet-B irradiation [94]. *PSD3* was also found as differentially expressed in a systematic gene expression profile comparisons of cell lines with different *NF1* status. *NF1* mutations are responsible for neurofibromatosis type I, a genetic disorder characterized, among other clinical signs, by cutaneous neurofibromas and **café-au-lait spots [95]**.

***FBXL15*** (F-Box And Leucine Rich Repeat Protein 15) encodes for a substrate recognition component of a SCF (SKP1-CUL1-F-box protein) E3 ubiquitin-protein ligase complex which mediates the **ubiquitination and subsequent proteasomal degradation** of SMURF1 (Smad ubiquitination regulatory factor 1), an homologous to **HECT-type E3 ubiquitin ligase**, thereby acting as a positive regulator of the **BMP signaling pathway** [96]. Notably, SMURF1 has been suggested as a **pro-mitophagic molecule** [97] based on the evidence provided by Orvedahl et al. [98]. For a role of mitophagy and melanoma/pigmentation, see Supplementary file S2 (*OGDH* gene). *FBXL15* is required for **dorsal/ventral pattern formation.** Pattern formation along the dorsal-ventral (DV) embryonic axis is fundamental in the establishment of the diverse cell types found in vertebrate embryos. The Bone Morphogenetic Protein (BMP) signaling pathway is key in this pattern formation process. BMPs are postulated to act as inductive signaling molecules or morphogens to establish distinct cell fates along the DV axis [99-103]. FBXL15 also mediates ubiquitination of SMURF2 and WWP2. The first protein is the E3 ubiquitin ligase responsible for the polyubiquitination and proteasome-mediated degradation of EZH2, which is required for **neuron differentiation** [104]. In addition, SMURF2 could interact with key proteins in the **Wnt-signaling cascade** (e.g. Dishevelled Segment Polarity Protein 2), [105]. The second protein is responsible for degradation of PTEN (phosphatase and tensin homolog), a well-characterized tumor suppressor. Decreased PTEN expression is observed in 30–50% of melanoma cell lines and in 5–20% of primary melanoma tumors [106]. Recent studies revealed PTEN as a key player in mediating the signaling pathways of both **DNA repair and melanocyte viability in the context of UV exposure** [107]. More importantly, loss of PTEN leads to the onset of **premature melanocyte senescence** presumably by super-physiological activation of the AKT oncoprotein [108], a phenotype termed oncogene-induced senescence (OIS) [109].

***CUEDC2*** (CUE Domain Containing 2) encodes a protein presenting a CUE domain that has ben shown to be a **key regulator of mitosis progression** [110]. Identified as **ubiquitin-binding motifs**, the CUE domains are small domains found in a variety of eukaryotic proteins, able to interact with both mono- and polyubiquitin, and playing a role in mono- and polyubiquitin recognition as well as in facilitating intramolecular mono-ubiquitination [111]. *CUEDC2* has been shown to play an important role in the development and progression of neoplasms and suggested to be involved in promoting **invasion and metastasis** in colorectal cancer [112]. It interacts with progesterone receptors (PR) and promotes **progesterone-induced PR degradation** by the **ubiquitin-proteasome pathway** [113]. CUEDC2 also down-regulates ESR1 (**estrogen receptor** 1) protein levels through the **ubiquitination-proteasome pathway** [114]. Whether CUEDC2 regulates the degradation of steroid hormone receptors other than ERα and PR, and whether its expression is correlated with steroid receptor expression in hormone-dependent cancers are not known. However, CUEDC2 expression is reduced in castrate-recurrent prostate cancer, which is characterized by increased androgen receptor activity, suggesting CUEDC2 may also act to dampen **androgen receptor signaling** [115]. In addition, CUEDC2 interacts with IKKalpha and IKKbeta and **represses activation of the transcription factor** **NF-kB** [116] (see nuclear factor-kappa-B, above). The transcription factor NF-kB is a critical regulator of diverse cytokine-mediated cellular responses and has a key function in cell survival by inducing the expression of genes encoding antiapoptotic molecules. In most cells, NF-kB proteins are normally inactive because they are sequestered in the cytoplasm by the IkB family of inhibitory proteins. Extracellular stimuli, including cytokines such as tumor necrosis factor (TNF) and interleukin 1 (IL-1), lead to activation of IkB kinase (*IKK*) complex, which phosphorylates and ubiquitinates IkB proteins and promotes their subsequent **degradation by the proteasome**. This process allows translocation of NF-kB proteins from the cytoplasm to the nucleus, where they activate expression of NF-kB-regulated genes [117]. Moreover, *CUEDC2* has been proposed as a regulator of ***JAK1*/*STAT3*** **signaling** [118,119]. **In response to UV irradiation**, *CUEDC2* undergoes ERK1/2-dependent phosphorylation and **ubiquitin-dependent degradation**, leading to APC/C(Cdh1)-mediated Cyclin A destruction, Cyclin-dependent kinase 2 inactivation, and **cell cycle arrest**, a mechanism required for providing a time window for DNA repair after **UV-induced DNA damage** [120]. *CUEDC2* was also suggested to be candidate disease biomarker for amyotrophic lateral sclerosis, a **neurodegenerative disease** characterized by **axonal retraction** and subsequent loss of motor neurons [121]. Among the factors associated with the particular vulnerability of these cells, a reduced function of the ubiquitin proteasome system has been suggested [122]. There is now solid evidence that proteostasis dysfunction secondary to **ubiquitin-proteasome system and autophagy** malfunction contributes to the loss of neuronal homeostasis [123].

***LOC112444535*** Uncharacterized locus

**MIR146B** (MicroRNA 146b) was identified among the circulating microRNAs associated with monocytic MDSC activity in melanoma patients. Myeloid-derived suppressor cells (MDSCs) represent a population of immature myeloid cells pathologically associated with cancer and known for their potent inhibitory activity on antitumor T cell immunity. They directly coordinate cancer cell plasticity triggering the **epithelial to mesenchymal transition** phenotype [124]. The microRNA 146b has been indeed shown to promote **migration** of normal thyroid follicular cells and **invasiveness** of papillary thyroid carcinoma cells [125,126] and induce **epithelial-mesenchymal transition** of papillary thyroid carcinoma cells [126], as well as to increase the cell surface levels of the **Wnt receptors** Frizzled-6 and LRP6 and enhance **Wnt/β-catenin signaling** [126]. In endometrial cancer, inhibition of microRNA 146b by **progesterone** has been shown to repress genes in the **Wnt/β‐catenin signaling** pathway [127]. It had been previously shown to be a **hormonally-regulated microRNA** also by Elsarraj et al. [128] who demonstrated how sex hormones (estrogen and progesterone) and prolactin resulted in the upregulation of the microRNA 146b levels in primary mammary epithelial cells. It was found to be differentially expressed between **uveal melanoma** tumors characterized by different type of chromosome 3 aberrations, and gene target prediction revealed **SMAD4**, WISP1, HIPK1, HDAC8 and **KIT** as post-transcriptional regulators of miR-146b [129]. Notably, KIT is a tyrosine-protein kinase that plays an essential role in the regulation of **cell survival** and proliferation, hematopoiesis, **stem cell maintenance**, gametogenesis, mast cell development, **migration** and function, and in **melanogenesis**. It can activate several signaling pathways. It activates the **AKT1 signaling pathway** by phosphorylation of PIK3R1, the regulatory subunit of phosphatidylinositol 3-kinase. Activated KIT also transmits signals via GRB2 and activation of RAS, RAF1 and the **MAP kinases** MAPK1/ERK2 and/or MAPK3/ERK1. Promotes activation of **STAT family members** STAT1, STAT3, STAT5A and STAT5B. Activation of PLCG1 leads to the production of the cellular signaling molecules diacylglycerol and inositol 1,4,5-trisphosphate. It promotes phosphorylation of LYN (see Supplementary file S3). Interestingly, KIT mutations are associated with **piebaldism**, a disorder affecting **melanocyte migration and development** characterized, in humans, by isolated congenital leukoderma (**white skin**) and poliosis (**white hair**) [130]. KIT-related piebald phenotypes are also known in pigs [131,132], cattle [133-136], sheep [137], horses [138], fishes [139] and hooded rats [140,141]. The latter show a pigmentation pattern in which **the entire ventral surface is white,** dorsal pigmentation is limited to the **head and shoulders** (the “hood”) and to a mid-dorsal stripe extending back to the **tip of the tail**. Moreover, miR-146b is increased in skin of patients with psoriasis, a chronic inflammatory skin disease [142,143] and with systemic sclerosis [144], and has been described as a specific psoriasis-associated microRNA that binds ACKR2, a scavenger of proinflammatory chemokines [145]. In addition to its role in inflammatory responses, it has been suggested to be capable of modulating **keratinocyte proliferation** in psoriatic skin [143].

***MFSD13A*** (Major Facilitator Superfamily Domain Containing 13A, alias *TMEM180*) encodes a transmembrane protein that belongs to the glycoside-pentoside-hexuronide (GPH):cation symporter family. Members of this family catalyze symport of a sugar molecule with a monovalent cation (H^+^ or Na^+^). It has been shown to be upregulated under low-oxygen conditions, and to possibly play a role in the uptake or metabolism of glutamine and arginine in **cancer cell proliferation** [146]. Its expression was shown to be not essential for mouse development, as *TMEM180* knockout mice do not exhibit embryonic, neonatal, or post-natal lethality [146]. A SNP (rs2001389) associated with pancreatic cancer risk has been shown to be also associated with lower expression of *TMEM180* and siRNA-mediated knockdown of TMEM180 was shown to promote proliferation of pancreatic cancer cell lines [147], while shRNA-mediated knockdown of *TMEM180* was shown to suppress proliferation of colorectal cancer cells [148]. Another member of the GPH:cation symporter family is *MATP*, (Membrane-Associated Transporter Protein, alias *SLC45A2*) which encodes a transporter protein involved in **melanin synthesis** and considered to be one of the most important genes affecting pigmentation in humans and various animal species [149,150] possibly regulating tyrosinase activity by controlling melanosome pH [151,152]. The protein is expressed in a high percentage of **melanoma** cell lines. Mutations in this gene are a cause of **oculocutaneous albinism** type 4, and polymorphisms in this gene are associated with **variations in skin and hair color** [153,154]. Mutations in another member of the major facilitator superfamily, *MFSD8*, whose transported substrate is yet unknown, are responsible for neuronal ceroid lipofuscinosis, a **lysosomal neurodegenerative disorder** characterized by **accumulation of abnormal storage material** [155]. TMEM180 interacts with ELAVL1 (Embryonic Lethal, Abnormal Vision ELAV- -Like RNA Binding Protein 1, alias HUR) [156].

***ACTR1A*** (Actin Related Protein 1A, alias *ARP1*) encodes a subunit of **dynactin**, a macromolecular complex that binds to both microtubules and cytoplasmic dynein. It is involved in a diverse array of cellular functions, including **ER-to-Golgi transport**, the centripetal **movement of lysosomes** and endosomes, spindle formation, chromosome movement, nuclear positioning, **axonogenesis** and **axonal transport** [157] **Colocalization** experiments in human skin cells *in vitro* highlighted an association of dynactin with **melanosomes** [158]. A study suggested that the dynactin complex links kinesin II to melanosomes mediating frog **melanosomes' motility** [159]. Another study suggested that spectrin may be involved in the dynactin **linkage of motor proteins to melanosomes**, as spectrin was found to interact with two different components of dynactin, including Arp1, in these cells [160]. Dynactin was also suggested to control **pigment localization** in amphibian eggs [161]. In 2012, melanoregulin, a product of the dilute suppressor gene, was shown to regulate microtubule-dependent **retrograde melanosome transport** through the dynein–dynactin motor complex [162].

***SUFU*** (SUFU Negative Regulator Of Hedgehog Signaling) belongs to the **Wnt** / Hedgehog / Notch super-pathway and encodes a negative regulator of the **sonic** **hedgehog signaling pathway** [163] that plays an important role in in **pattern formation** and cellular **proliferation during development**. Notably, SUFU acts by repressing the sonic hedgehog target gene *GLI1*. The repression of GLI1 by SUFU can be removed after SUFU binding with STIL. Recent studies have shown that STIL plays important role in **proliferation, survival, regeneration**, and possibly functions of **neuronal cells** [164-166]. *SUFU* has been associated with a **ciliopathy** (Joubert Syndrome 32). Choi et al. [167] demonstrated that **ciliogenesis has a critical function in melanogenesis**. Indeed, they showed that the induction of primary cilium formation suppresses melanin production by **reducing the expression of melanogenic enzymes**. *SUFU* has been also associated with the basal cell nevus syndrome, a familial cancer syndrome characterized by numerous basal cell carcinomas, along with skeletal, ophthalmologic, and neurologic abnormalities. Elevated SUFU protein levels were positively correlated with the diameter and **invasion** of the tumor in colon cancer [168]. *SUFU* has been also associated with medulloblastoma and meningiomas predisposition [169-171]. Hippocampal expression of *SUFU* was significantly reduced in the 20-month-aged animals vs. 2-months mice due to age-associated DNA methylation so that the gene expression change was suggested as marker for brain **aging** [172].

***TRIM8*** (Tripartite Motif Containing 8) is a member of the tripartite motif (TRIM) protein family. The different domains of TRIM family proteins regulate cellular localization and higher order structures and are involved in several functional activities. Most of the TRIM family proteins, including TRIM8, have also been defined as E3 ubiquitin or ubiquitin like molecule ligases (SUMOylation and ISGylation), suggesting their main role in the regulation of cellular protein stability and tuning. In particular, their RING domain endows the **E3 ubiquitin-protein ligase activity**. TRIM proteins are involved in a **broad range of biological processes**, and have important roles in **differentiation**, **development**, **intracellular signaling, protein quality control, autophagy**, and **immune responses**, by regulating various signaling pathways. Furthermore, many TRIM proteins are induced by type I and type II interferons (IFNs) [173], suggesting that TRIM proteins have an important role in anti-viral and anti-microbial systems. It also plays a role in the TNFalpha and IL-1beta signaling pathways. Mutations in the genes encoding certain TRIMs have been associated with human diseases, classified as immunological diseases, or **developmental disorders**. Moreover, several TRIM members are involved in **cancer** either as tumor suppressors genes or as oncogenes, by controlling a broad range of processes including transcriptional regulation, cell proliferation, apoptosis, **DNA repair**, and **metastasis**. TRIM8 has been shown to be a **p53** direct target gene, and by a positive feedback loop, TRIM8 is able to potentiate the p53 tumor suppressor activity, controlling the molecular switch that directs p53 toward the transcriptional activation of cell cycle arrest [174] and DNA repair genes, leading to the **suppression of cell proliferation** [175]. On the other hand, studies have proposed *TRIM8* as a novel oncogene, because it is involved in the **positive regulation of the NF-κB pathway** via **JAK-STAT signaling pathway** [176-179] or via JNK-MAPK patway [180]. *TRIM8* is expressed in undifferentiated embryonic stem (ES) cells, suggesting that TRIM8, besides regulating stemness in glioblastoma [177], also plays an important role for **maintaining pluripotency** of ES cells. Knock-down of *TRIM8* modestly but significantly upregulated transcription of Nanog and when protein expression of Nanog is upregulated by knock-down of *TRIM8*, ES cells may differentiate [181]. This role for TRIM8 would be consistent with the evidence for a role of STAT3 in self-renewal of pluripotent embryonic stem cells [182]. TRIM8 interacts with KIFC1, and KIF11/Eg5, two master regulators of mitotic spindle assembly and cytoskeleton reorganization [183].

**REFERENCES**

1. Storbeck, C. J.; Wagner, S.; O'Reilly, P.; McKay, M.; Parks, R. J.; Westphal, H.; Sabourin, L. A., The Ldb1 and Ldb2 transcriptional cofactors interact with the Ste20-like kinase SLK and regulate cell migration. *Mol Biol Cell* **2009,** *20* (19), 4174-82.

2. Matthews, J. M.; Visvader, J. E., LIM-domain-binding protein 1: a multifunctional cofactor that interacts with diverse proteins. *EMBO Rep* **2003,** *4* (12), 1132-7.

3. Almaidhan, A.; Cesario, J.; Landin Malt, A.; Zhao, Y.; Sharma, N.; Choi, V.; Jeong, J., Neural crest-specific deletion of Ldb1 leads to cleft secondary palate with impaired palatal shelf elevation. *BMC Dev Biol* **2014,** *14*, 3.

4. Salmans, M. L.; Yu, Z.; Watanabe, K.; Cam, E.; Sun, P.; Smyth, P.; Dai, X.; Andersen, B., The co-factor of LIM domains (CLIM/LDB/NLI) maintains basal mammary epithelial stem cells and promotes breast tumorigenesis. *PLoS Genet* **2014,** *10* (7), e1004520.

5. Li, L.; Jothi, R.; Cui, K.; Lee, J. Y.; Cohen, T.; Gorivodsky, M.; Tzchori, I.; Zhao, Y.; Hayes, S. M.; Bresnick, E. H.; Zhao, K.; Westphal, H.; Love, P. E., Nuclear adaptor Ldb1 regulates a transcriptional program essential for the maintenance of hematopoietic stem cells. *Nat Immunol* **2011,** *12* (2), 129-36.

6. Li, L.; Mitra, A.; Cui, K.; Zhao, B.; Choi, S.; Lee, J. Y.; Stamos, D. B.; El-Khoury, D.; Warzecha, C.; Pfeifer, K.; Hardwick, J.; Zhao, K.; Venters, B.; Dave, U. P.; Love, P. E., Ldb1 is required for Lmo2 oncogene-induced thymocyte self-renewal and T-cell acute lymphoblastic leukemia. *Blood* **2020,** *135* (25), 2252-2265.

7. Makarev, E.; Gorivodsky, M., Islet1 and its co-factor Ldb1 are expressed in quiescent cells of mouse intestinal epithelium. *PLoS One* **2014,** *9* (4), e95256.

8. Xu, X.; Mannik, J.; Kudryavtseva, E.; Lin, K. K.; Flanagan, L. A.; Spencer, J.; Soto, A.; Wang, N.; Lu, Z.; Yu, Z.; Monuki, E. S.; Andersen, B., Co-factors of LIM domains (Clims/Ldb/Nli) regulate corneal homeostasis and maintenance of hair follicle stem cells. *Dev Biol* **2007,** *312* (2), 484-500.

9. Rhee, H.; Polak, L.; Fuchs, E., Lhx2 maintains stem cell character in hair follicles. *Science* **2006,** *312* (5782), 1946-9.

10. Folgueras, A. R.; Guo, X.; Pasolli, H. A.; Stokes, N.; Polak, L.; Zheng, D.; Fuchs, E., Architectural niche organization by LHX2 is linked to hair follicle stem cell function. *Cell Stem Cell* **2013,** *13* (3), 314-27.

11. Tornqvist, G.; Sandberg, A.; Hagglund, A. C.; Carlsson, L., Cyclic expression of lhx2 regulates hair formation. *PLoS Genet* **2010,** *6* (4), e1000904.

12. Mardaryev, A. N.; Meier, N.; Poterlowicz, K.; Sharov, A. A.; Sharova, T. Y.; Ahmed, M. I.; Rapisarda, V.; Lewis, C.; Fessing, M. Y.; Ruenger, T. M.; Bhawan, J.; Werner, S.; Paus, R.; Botchkarev, V. A., Lhx2 differentially regulates Sox9, Tcf4 and Lgr5 in hair follicle stem cells to promote epidermal regeneration after injury. *Development* **2011,** *138* (22), 4843-52.

13. Glenn, D. J.; Maurer, R. A., MRG1 binds to the LIM domain of Lhx2 and may function as a coactivator to stimulate glycoprotein hormone alpha-subunit gene expression. *J Biol Chem* **1999,** *274* (51), 36159-67.

14. Sun, H. B.; Zhu, Y. X.; Yin, T.; Sledge, G.; Yang, Y. C., MRG1, the product of a melanocyte-specific gene related gene, is a cytokine-inducible transcription factor with transformation activity. *Proc Natl Acad Sci U S A* **1998,** *95* (23), 13555-60.

15. Ahmed, N. U.; Shioda, T.; Coser, K. R.; Ichihashi, M.; Ueda, M., Aberrant expression of MSG1 transcriptional activator in human malignant melanoma in vivo. *Pigment Cell Res* **2001,** *14* (2), 140-3.

16. Nair, S. S.; Chaubal, V. A.; Shioda, T.; Coser, K. R.; Mojamdar, M., Over-expression of MSG1 transcriptional co-activator increases melanin in B16 melanoma cells: a possible role for MSG1 in melanogenesis. *Pigment Cell Res* **2001,** *14* (3), 206-9.

17. Li, H.; Ahmed, N. U.; Fenner, M. H.; Ueda, M.; Isselbacher, K. J.; Shioda, T., Regulation of expression of MSG1 melanocyte-specific nuclear protein in human melanocytes and melanoma cells. *Exp Cell Res* **1998,** *242* (2), 478-86.

18. Howlin, J.; Cirenajwis, H.; Lettiero, B.; Staaf, J.; Lauss, M.; Saal, L.; Borg, A.; Gruvberger-Saal, S.; Jonsson, G., Loss of CITED1, an MITF regulator, drives a phenotype switch in vitro and can predict clinical outcome in primary melanoma tumours. *PeerJ* **2015,** *3*, e788.

19. Fialkowski, A. C.; Levy, D. J.; Watkins-Chow, D. E.; Palmer, J. W.; Darji, R.; Tiwari, H. K.; Pavan, W. J.; Harris, M. L., Identification of Gene Variants Associated with Melanocyte Stem Cell Differentiation in Mice Predisposed for Hair Graying. *G3 (Bethesda)* **2019,** *9* (3), 817-827.

20. Garcia, S. A.; Swiersy, A.; Radhakrishnan, P.; Branchi, V.; Kanth Nanduri, L.; Gyorffy, B.; Betzler, A. M.; Bork, U.; Kahlert, C.; Reissfelder, C.; Rahbari, N. N.; Weitz, J.; Scholch, S., LDB1 overexpression is a negative prognostic factor in colorectal cancer. *Oncotarget* **2016,** *7* (51), 84258-84270.

21. Maksimenko, O.; Georgiev, P., Mechanisms and proteins involved in long-distance interactions. *Front Genet* **2014,** *5*, 28.

22. Song, S. H.; Hou, C.; Dean, A., A positive role for NLI/Ldb1 in long-range beta-globin locus control region function. *Mol Cell* **2007,** *28* (5), 810-22.

23. Klein, R. H.; Stephens, D. N.; Ho, H.; Chen, J. K.; Salmans, M. L.; Wang, W.; Yu, Z.; Andersen, B., Cofactors of LIM Domains Associate with Estrogen Receptor alpha to Regulate the Expression of Noncoding RNA H19 and Corneal Epithelial Progenitor Cell Function. *J Biol Chem* **2016,** *291* (25), 13271-85.

24. Johnsen, S. A.; Gungor, C.; Prenzel, T.; Riethdorf, S.; Riethdorf, L.; Taniguchi-Ishigaki, N.; Rau, T.; Tursun, B.; Furlow, J. D.; Sauter, G.; Scheffner, M.; Pantel, K.; Gannon, F.; Bach, I., Regulation of estrogen-dependent transcription by the LIM cofactors CLIM and RLIM in breast cancer. *Cancer Res* **2009,** *69* (1), 128-36.

25. Gungor, C.; Taniguchi-Ishigaki, N.; Ma, H.; Drung, A.; Tursun, B.; Ostendorff, H. P.; Bossenz, M.; Becker, C. G.; Becker, T.; Bach, I., Proteasomal selection of multiprotein complexes recruited by LIM homeodomain transcription factors. *Proc Natl Acad Sci U S A* **2007,** *104* (38), 15000-5.

26. Vaman, V. S. A.; Poppe, H.; Houben, R.; Grunewald, T. G.; Goebeler, M.; Butt, E., LASP1, a Newly Identified Melanocytic Protein with a Possible Role in Melanin Release, but Not in Melanoma Progression. *PLoS One* **2015,** *10* (6), e0129219.

27. Chizhikov, V.; Steshina, E.; Roberts, R.; Ilkin, Y.; Washburn, L.; Millen, K. J., Molecular definition of an allelic series of mutations disrupting the mouse Lmx1a (dreher) gene. *Mamm Genome* **2006,** *17* (10), 1025-32.

28. Steffes, G.; Lorente-Canovas, B.; Pearson, S.; Brooker, R. H.; Spiden, S.; Kiernan, A. E.; Guenet, J. L.; Steel, K. P., Mutanlallemand (mtl) and Belly Spot and Deafness (bsd) are two new mutations of Lmx1a causing severe cochlear and vestibular defects. *PLoS One* **2012,** *7* (11), e51065.

29. Manzanares, M.; Trainor, P. A.; Ariza-McNaughton, L.; Nonchev, S.; Krumlauf, R., Dorsal patterning defects in the hindbrain, roof plate and skeleton in the dreher (dr(J)) mouse mutant. *Mech Dev* **2000,** *94* (1-2), 147-56.

30. Fujimoto, N.; Yeh, S.; Kang, H. Y.; Inui, S.; Chang, H. C.; Mizokami, A.; Chang, C., Cloning and characterization of androgen receptor coactivator, ARA55, in human prostate. *J Biol Chem* **1999,** *274* (12), 8316-21.

31. Wang, H.; Song, K.; Sponseller, T. L.; Danielpour, D., Novel function of androgen receptor-associated protein 55/Hic-5 as a negative regulator of Smad3 signaling. *J Biol Chem* **2005,** *280* (7), 5154-62.

32. Noguchi, F.; Inui, S.; Nakajima, T.; Itami, S., Hic-5 affects proliferation, migration and invasion of B16 murine melanoma cells. *Pigment Cell Melanoma Res* **2012,** *25* (6), 773-82.

33. Mei, X.; Westfall, T. A.; Zhang, Q.; Sheffield, V. C.; Bassuk, A. G.; Slusarski, D. C., Functional characterization of Prickle2 and BBS7 identify overlapping phenotypes yet distinct mechanisms. *Dev Biol* **2014,** *392* (2), 245-55.

34. Yen, H. J.; Tayeh, M. K.; Mullins, R. F.; Stone, E. M.; Sheffield, V. C.; Slusarski, D. C., Bardet-Biedl syndrome genes are important in retrograde intracellular trafficking and Kupffer's vesicle cilia function. *Hum Mol Genet* **2006,** *15* (5), 667-77.

35. Ahsan, K.; Singh, N.; Rocha, M.; Huang, C.; Prince, V. E., Prickle1 is required for EMT and migration of zebrafish cranial neural crest. *Dev Biol* **2019,** *448* (1), 16-35.

36. Mirebeau-Prunier, D.; Le Pennec, S.; Jacques, C.; Gueguen, N.; Poirier, J.; Malthiery, Y.; Savagner, F., Estrogen-related receptor alpha and PGC-1-related coactivator constitute a novel complex mediating the biogenesis of functional mitochondria. *FEBS J* **2010,** *277* (3), 713-25.

37. Scarpulla, R. C., Metabolic control of mitochondrial biogenesis through the PGC-1 family regulatory network. *Biochim Biophys Acta* **2011,** *1813* (7), 1269-78.

38. Daniele, T.; Hurbain, I.; Vago, R.; Casari, G.; Raposo, G.; Tacchetti, C.; Schiaffino, M. V., Mitochondria and melanosomes establish physical contacts modulated by Mfn2 and involved in organelle biogenesis. *Curr Biol* **2014,** *24* (4), 393-403.

39. Zhang, Z.; Gong, J.; Sviderskaya, E. V.; Wei, A.; Li, W., Mitochondrial NCKX5 regulates melanosomal biogenesis and pigment production. *J Cell Sci* **2019,** *132* (14).

40. Lamason, R. L.; Mohideen, M. A.; Mest, J. R.; Wong, A. C.; Norton, H. L.; Aros, M. C.; Jurynec, M. J.; Mao, X.; Humphreville, V. R.; Humbert, J. E.; Sinha, S.; Moore, J. L.; Jagadeeswaran, P.; Zhao, W.; Ning, G.; Makalowska, I.; McKeigue, P. M.; O'Donnell, D.; Kittles, R.; Parra, E. J.; Mangini, N. J.; Grunwald, D. J.; Shriver, M. D.; Canfield, V. A.; Cheng, K. C., SLC24A5, a putative cation exchanger, affects pigmentation in zebrafish and humans. *Science* **2005,** *310* (5755), 1782-6.

41. Wei, A. H.; Zang, D. J.; Zhang, Z.; Liu, X. Z.; He, X.; Yang, L.; Wang, Y.; Zhou, Z. Y.; Zhang, M. R.; Dai, L. L.; Yang, X. M.; Li, W., Exome sequencing identifies SLC24A5 as a candidate gene for nonsyndromic oculocutaneous albinism. *J Invest Dermatol* **2013,** *133* (7), 1834-40.

42. Dell'Anna, M. L.; Maresca, V.; Briganti, S.; Camera, E.; Falchi, M.; Picardo, M., Mitochondrial impairment in peripheral blood mononuclear cells during the active phase of vitiligo. *J Invest Dermatol* **2001,** *117* (4), 908-13.

43. Laddha, N. C.; Dwivedi, M.; Mansuri, M. S.; Gani, A. R.; Ansarullah, M.; Ramachandran, A. V.; Dalai, S.; Begum, R., Vitiligo: interplay between oxidative stress and immune system. *Exp Dermatol* **2013,** *22* (4), 245-50.

44. Williams, D.; Jung, D. W.; Khersonsky, S. M.; Heidary, N.; Chang, Y. T.; Orlow, S. J., Identification of compounds that bind mitochondrial F1F0 ATPase by screening a triazine library for correction of albinism. *Chem Biol* **2004,** *11* (9), 1251-9.

45. Tonks, I. D.; Mukhopadhyay, P.; Schroder, W. A.; Sorolla, A.; Mould, A. W.; Handoko, H. Y.; Ferguson, B.; Muller, H. K.; Keith, P.; Hayward, N. K.; Walker, G. J.; Kay, G. F., Melanocyte transformation requires complete loss of all pocket protein function via a mechanism that mitigates the need for MAPK pathway activation. *Oncogene* **2017,** *36* (26), 3789-3795.

46. Halaban, R.; Cheng, E.; Smicun, Y.; Germino, J., Deregulated E2F transcriptional activity in autonomously growing melanoma cells. *J Exp Med* **2000,** *191* (6), 1005-16.

47. Halaban, R., Melanoma cell autonomous growth: the Rb/E2F pathway. *Cancer Metastasis Rev* **1999,** *18* (3), 333-43.

48. Pettus, B. J.; Chalfant, C. E.; Hannun, Y. A., Ceramide in apoptosis: an overview and current perspectives. *Biochim Biophys Acta* **2002,** *1585* (2-3), 114-25.

49. Kim, D. S.; Kim, S. Y.; Moon, S. J.; Chung, J. H.; Kim, K. H.; Cho, K. H.; Park, K. C., Ceramide inhibits cell proliferation through Akt/PKB inactivation and decreases melanin synthesis in Mel-Ab cells. *Pigment Cell Res* **2001,** *14* (2), 110-5.

50. Ando, H.; Watabe, H.; Valencia, J. C.; Yasumoto, K.; Furumura, M.; Funasaka, Y.; Oka, M.; Ichihashi, M.; Hearing, V. J., Fatty acids regulate pigmentation via proteasomal degradation of tyrosinase: a new aspect of ubiquitin-proteasome function. *J Biol Chem* **2004,** *279* (15), 15427-33.

51. Simons, K.; Ikonen, E., Functional rafts in cell membranes. *Nature* **1997,** *387* (6633), 569-72.

52. Simons, K.; Toomre, D., Lipid rafts and signal transduction. *Nat Rev Mol Cell Biol* **2000,** *1* (1), 31-9.

53. Jakobsson, A.; Westerberg, R.; Jacobsson, A., Fatty acid elongases in mammals: their regulation and roles in metabolism. *Prog Lipid Res* **2006,** *45* (3), 237-49.

54. Hartmann, D.; Wegner, M. S.; Wanger, R. A.; Ferreiros, N.; Schreiber, Y.; Lucks, J.; Schiffmann, S.; Geisslinger, G.; Grosch, S., The equilibrium between long and very long chain ceramides is important for the fate of the cell and can be influenced by co-expression of CerS. *Int J Biochem Cell Biol* **2013,** *45* (7), 1195-203.

55. Sassa, T.; Suto, S.; Okayasu, Y.; Kihara, A., A shift in sphingolipid composition from C24 to C16 increases susceptibility to apoptosis in HeLa cells. *Biochim Biophys Acta* **2012,** *1821* (7), 1031-7.

56. Kiamehr, M.; Heiskanen, L.; Laufer, T.; Dusterloh, A.; Kahraman, M.; Kakela, R.; Laaksonen, R.; Aalto-Setala, K., Dedifferentiation of Primary Hepatocytes is Accompanied with Reorganization of Lipid Metabolism Indicated by Altered Molecular Lipid and miRNA Profiles. *Int J Mol Sci* **2019,** *20* (12).

57. Kang, Y. P.; Yoon, J. H.; Long, N. P.; Koo, G. B.; Noh, H. J.; Oh, S. J.; Lee, S. B.; Kim, H. M.; Hong, J. Y.; Lee, W. J.; Lee, S. J.; Hong, S. S.; Kwon, S. W.; Kim, Y. S., Spheroid-Induced Epithelial-Mesenchymal Transition Provokes Global Alterations of Breast Cancer Lipidome: A Multi-Layered Omics Analysis. *Front Oncol* **2019,** *9*, 145.

58. Chen, H.; Gao, L.; Yang, D.; Xiao, Y.; Zhang, M.; Li, C.; Wang, A.; Jin, Y., Coordination between the circadian clock and androgen signaling is required to sustain rhythmic expression of Elovl3 in mouse liver. *J Biol Chem* **2019,** *294* (17), 7046-7056.

59. Ruiz-Larranaga, O.; Langa, J.; Rendo, F.; Manzano, C.; Iriondo, M.; Estonba, A., Genomic selection signatures in sheep from the Western Pyrenees. *Genet Sel Evol* **2018,** *50* (1), 9.

60. Peng, J.; He, M. H.; Duan, Y. M.; Liu, Y. T.; Zhou, J. Q., Inhibition of telomere recombination by inactivation of KEOPS subunit Cgi121 promotes cell longevity. *PLoS Genet* **2015,** *11* (3), e1005071.

61. Nocelli, C.; Cappelli, K.; Capomaccio, S.; Pascucci, L.; Mercati, F.; Pazzaglia, I.; Mecocci, S.; Antonini, M.; Renieri, C., Shedding light on cashmere goat hair follicle biology: from morphology analyses to transcriptomic landascape. *BMC Genomics* **2020,** *21* (1), 458.

62. Zhang, L.; Sun, F.; Jin, H.; Dalrymple, B. P.; Cao, Y.; Wei, T.; Vuocolo, T.; Zhang, M.; Piao, Q.; Ingham, A. B., A comparison of transcriptomic patterns measured in the skin of Chinese fine and coarse wool sheep breeds. *Sci Rep* **2017,** *7* (1), 14301.

63. Gonzalez-Bengtsson, A.; Asadi, A.; Gao, H.; Dahlman-Wright, K.; Jacobsson, A., Estrogen Enhances the Expression of the Polyunsaturated Fatty Acid Elongase Elovl2 via ERalpha in Breast Cancer Cells. *PLoS One* **2016,** *11* (10), e0164241.

64. Kouwenhoven, W. M.; von Oerthel, L.; Smidt, M. P., Pitx3 and En1 determine the size and molecular programming of the dopaminergic neuronal pool. *PLoS One* **2017,** *12* (8), e0182421.

65. VandenBoom, T.; Quan, V. L.; Zhang, B.; Garfield, E. M.; Kong, B. Y.; Isales, M. C.; Panah, E.; Igartua, C.; Taxter, T.; Beaubier, N.; White, K.; Gerami, P., Genomic Fusions in Pigmented Spindle Cell Nevus of Reed. *Am J Surg Pathol* **2018,** *42* (8), 1042-1051.

66. Morkin, M. I.; Kapadia, M. K.; Laver, N. V., Pigmented Spindle Cell Nevus of Reed of the Eyelid. *Ocul Oncol Pathol* **2017,** *3* (3), 176-180.

67. Asadi, A.; Jorgensen, J.; Jacobsson, A., Elovl1 and p55Cdc genes are localized in a tail-to-tail array and are co-expressed in proliferating cells. *J Biol Chem* **2002,** *277* (21), 18494-500.

68. Kawamoto, K.; Yoshida, Y.; Tamaki, H.; Torii, S.; Shinotsuka, C.; Yamashina, S.; Nakayama, K., GBF1, a guanine nucleotide exchange factor for ADP-ribosylation factors, is localized to the cis-Golgi and involved in membrane association of the COPI coat. *Traffic* **2002,** *3* (7), 483-95.

69. Garcia-Mata, R.; Szul, T.; Alvarez, C.; Sztul, E., ADP-ribosylation factor/COPI-dependent events at the endoplasmic reticulum-Golgi interface are regulated by the guanine nucleotide exchange factor GBF1. *Mol Biol Cell* **2003,** *14* (6), 2250-61.

70. Zhao, X.; Claude, A.; Chun, J.; Shields, D. J.; Presley, J. F.; Melancon, P., GBF1, a cis-Golgi and VTCs-localized ARF-GEF, is implicated in ER-to-Golgi protein traffic. *J Cell Sci* **2006,** *119* (Pt 18), 3743-53.

71. Szul, T.; Grabski, R.; Lyons, S.; Morohashi, Y.; Shestopal, S.; Lowe, M.; Sztul, E., Dissecting the role of the ARF guanine nucleotide exchange factor GBF1 in Golgi biogenesis and protein trafficking. *J Cell Sci* **2007,** *120* (Pt 22), 3929-40.

72. Manolea, F.; Claude, A.; Chun, J.; Rosas, J.; Melancon, P., Distinct functions for Arf guanine nucleotide exchange factors at the Golgi complex: GBF1 and BIGs are required for assembly and maintenance of the Golgi stack and trans-Golgi network, respectively. *Mol Biol Cell* **2008,** *19* (2), 523-35.

73. Quilty, D.; Gray, F.; Summerfeldt, N.; Cassel, D.; Melancon, P., Arf activation at the Golgi is modulated by feed-forward stimulation of the exchange factor GBF1. *J Cell Sci* **2014,** *127* (Pt 2), 354-64.

74. Wang, J.; Fresquez, T.; Kandachar, V.; Deretic, D., The Arf GEF GBF1 and Arf4 synergize with the sensory receptor cargo, rhodopsin, to regulate ciliary membrane trafficking. *J Cell Sci* **2017,** *130* (23), 3975-3987.

75. Cornejo, V. H.; Gonzalez, C.; Campos, M.; Vargas-Saturno, L.; Juricic, M. L. A.; Miserey-Lenkei, S.; Pertusa, M.; Madrid, R.; Couve, A., Non-conventional Axonal Organelles Control TRPM8 Ion Channel Trafficking and Peripheral Cold Sensing. *Cell Rep* **2020,** *30* (13), 4505-4517 e5.

76. Ryu, B.; Kim, D. S.; Deluca, A. M.; Alani, R. M., Comprehensive expression profiling of tumor cell lines identifies molecular signatures of melanoma progression. *PLoS One* **2007,** *2* (7), e594.

77. Ueda, Y.; Richmond, A., NF-kappaB activation in melanoma. *Pigment Cell Res* **2006,** *19* (2), 112-24.

78. Dhawan, P.; Richmond, A., Role of CXCL1 in tumorigenesis of melanoma. *J Leukoc Biol* **2002,** *72* (1), 9-18.

79. McNulty, S. E.; del Rosario, R.; Cen, D.; Meyskens, F. L., Jr.; Yang, S., Comparative expression of NFkappaB proteins in melanocytes of normal skin vs. benign intradermal naevus and human metastatic melanoma biopsies. *Pigment Cell Res* **2004,** *17* (2), 173-80.

80. McNulty, S. E.; Tohidian, N. B.; Meyskens, F. L., Jr., RelA, p50 and inhibitor of kappa B alpha are elevated in human metastatic melanoma cells and respond aberrantly to ultraviolet light B. *Pigment Cell Res* **2001,** *14* (6), 456-65.

81. Bosserhoff, A. K.; Hein, R.; Bogdahn, U.; Buettner, R., Structure and promoter analysis of the gene encoding the human melanoma-inhibiting protein MIA. *J Biol Chem* **1996,** *271* (1), 490-5.

82. De Donatis, G. M.; Le Pape, E.; Pierron, A.; Cheli, Y.; Hofman, V.; Hofman, P.; Allegra, M.; Zahaf, K.; Bahadoran, P.; Rocchi, S.; Bertolotto, C.; Ballotti, R.; Passeron, T., NF-kB2 induces senescence bypass in melanoma via a direct transcriptional activation of EZH2. *Oncogene* **2016,** *35* (21), 2735-45.

83. Zehavi, L.; Schayek, H.; Jacob-Hirsch, J.; Sidi, Y.; Leibowitz-Amit, R.; Avni, D., MiR-377 targets E2F3 and alters the NF-kB signaling pathway through MAP3K7 in malignant melanoma. *Mol Cancer* **2015,** *14*, 68.

84. Quehenberger, P.; Bierhaus, A.; Fasching, P.; Muellner, C.; Klevesath, M.; Hong, M.; Stier, G.; Sattler, M.; Schleicher, E.; Speiser, W.; Nawroth, P. P., Endothelin 1 transcription is controlled by nuclear factor-kappaB in AGE-stimulated cultured endothelial cells. *Diabetes* **2000,** *49* (9), 1561-70.

85. Yajima, I.; Kumasaka, M. Y.; Iida, M.; Oshino, R.; Tanihata, H.; Al Hossain, A.; Ohgami, N.; Kato, M., Arsenic-mediated hyperpigmentation in skin via NF-kappa B/endothelin-1 signaling in an originally developed hairless mouse model. *Arch Toxicol* **2017,** *91* (11), 3507-3516.

86. Dey-Rao, R.; Sinha, A. A., Vitiligo blood transcriptomics provides new insights into disease mechanisms and identifies potential novel therapeutic targets. *BMC Genomics* **2017,** *18* (1), 109.

87. Kim, K. H.; Choi, H.; Kim, H. J.; Lee, T. R., TNFSF14 inhibits melanogenesis via NF-kB signaling in melanocytes. *Cytokine* **2018,** *110*, 126-130.

88. Zhou, J.; Shang, J.; Song, J.; Ping, F., Interleukin-18 augments growth ability of primary human melanocytes by PTEN inactivation through the AKT/NF-kappaB pathway. *Int J Biochem Cell Biol* **2013,** *45* (2), 308-16.

89. Sun, L.; Pan, S.; Yang, Y.; Sun, J.; Liang, D.; Wang, X.; Xie, X.; Hu, J., Toll-like receptor 9 regulates melanogenesis through NF-kappaB activation. *Exp Biol Med (Maywood)* **2016,** *241* (14), 1497-504.

90. Englaro, W.; Bahadoran, P.; Bertolotto, C.; Busca, R.; Derijard, B.; Livolsi, A.; Peyron, J. F.; Ortonne, J. P.; Ballotti, R., Tumor necrosis factor alpha-mediated inhibition of melanogenesis is dependent on nuclear factor kappa B activation. *Oncogene* **1999,** *18* (8), 1553-9.

91. Webster, M. R.; Weeraratna, A. T., A Wnt-er migration: the confusing role of beta-catenin in melanoma metastasis. *Sci Signal* **2013,** *6* (268), pe11.

92. Grossmann, A. H.; Yoo, J. H.; Clancy, J.; Sorensen, L. K.; Sedgwick, A.; Tong, Z.; Ostanin, K.; Rogers, A.; Grossmann, K. F.; Tripp, S. R.; Thomas, K. R.; D'Souza-Schorey, C.; Odelberg, S. J.; Li, D. Y., The small GTPase ARF6 stimulates beta-catenin transcriptional activity during WNT5A-mediated melanoma invasion and metastasis. *Sci Signal* **2013,** *6* (265), ra14.

93. Xue, Y.; Shen, S. Q.; Corbo, J. C.; Kefalov, V. J., Circadian and light-driven regulation of rod dark adaptation. *Sci Rep* **2015,** *5*, 17616.

94. Lopez, S.; Smith-Zubiaga, I.; Garcia de Galdeano, A.; Boyano, M. D.; Garcia, O.; Gardeazabal, J.; Martinez-Cadenas, C.; Izagirre, N.; de la Rua, C.; Alonso, S., Comparison of the Transcriptional Profiles of Melanocytes from Dark and Light Skinned Individuals under Basal Conditions and Following Ultraviolet-B Irradiation. *PLoS One* **2015,** *10* (8), e0134911.

95. Sun, D., Identification of transcriptional mechanisms downstream of nf1 gene defeciency in malignant peripheral nerve sheath tumors. (Doctoral dissertation) **2012**. http://digitalcommons.wayne.edu/oa_dissertations

96. Cui, Y.; He, S.; Xing, C.; Lu, K.; Wang, J.; Xing, G.; Meng, A.; Jia, S.; He, F.; Zhang, L., SCFFBXL(1)(5) regulates BMP signalling by directing the degradation of HECT-type ubiquitin ligase Smurf1. *EMBO J* **2011,** *30* (13), 2675-89.

97. Maes, H.; Agostinis, P., Autophagy and mitophagy interplay in melanoma progression. *Mitochondrion* **2014,** *19 Pt A*, 58-68.

98. Orvedahl, A.; Sumpter, R., Jr.; Xiao, G.; Ng, A.; Zou, Z.; Tang, Y.; Narimatsu, M.; Gilpin, C.; Sun, Q.; Roth, M.; Forst, C. V.; Wrana, J. L.; Zhang, Y. E.; Luby-Phelps, K.; Xavier, R. J.; Xie, Y.; Levine, B., Image-based genome-wide siRNA screen identifies selective autophagy factors. *Nature* **2011,** *480* (7375), 113-7.

99. Ma, T. C.; Vong, K. I.; Kwan, K. M., Spatiotemporal Decline of BMP Signaling Activity in Neural Progenitors Mediates Fate Transition and Safeguards Neurogenesis. *Cell Rep* **2020,** *30* (11), 3616-3624 e4.

100. Takebayashi-Suzuki, K.; Suzuki, A., Intracellular Communication among Morphogen Signaling Pathways during Vertebrate Body Plan Formation. *Genes (Basel)* **2020,** *11* (3).

101. Wu, M. Y.; Hill, C. S., Tgf-beta superfamily signaling in embryonic development and homeostasis. *Dev Cell* **2009,** *16* (3), 329-43.

102. Kishigami, S.; Mishina, Y., BMP signaling and early embryonic patterning. *Cytokine Growth Factor Rev* **2005,** *16* (3), 265-78.

103. Munoz-Sanjuan, I.; A, H. B., Early posterior/ventral fate specification in the vertebrate embryo. *Dev Biol* **2001,** *237* (1), 1-17.

104. Yu, Y. L.; Chou, R. H.; Shyu, W. C.; Hsieh, S. C.; Wu, C. S.; Chiang, S. Y.; Chang, W. J.; Chen, J. N.; Tseng, Y. J.; Lin, Y. H.; Lee, W.; Yeh, S. P.; Hsu, J. L.; Yang, C. C.; Hung, S. C.; Hung, M. C., Smurf2-mediated degradation of EZH2 enhances neuron differentiation and improves functional recovery after ischaemic stroke. *EMBO Mol Med* **2013,** *5* (4), 531-47.

105. Lian, G.; Chenn, A.; Ekuta, V.; Kanaujia, S.; Sheen, V., Formin 2 Regulates Lysosomal Degradation of Wnt-Associated beta-Catenin in Neural Progenitors. *Cereb Cortex* **2019,** *29* (5), 1938-1952.

106. Wu, H.; Goel, V.; Haluska, F. G., PTEN signaling pathways in melanoma. *Oncogene* **2003,** *22* (20), 3113-22.

107. Ming, M.; Feng, L.; Shea, C. R.; Soltani, K.; Zhao, B.; Han, W.; Smart, R. C.; Trempus, C. S.; He, Y. Y., PTEN positively regulates UVB-induced DNA damage repair. *Cancer Res* **2011,** *71* (15), 5287-95.

108. Chen, Z.; Trotman, L. C.; Shaffer, D.; Lin, H. K.; Dotan, Z. A.; Niki, M.; Koutcher, J. A.; Scher, H. I.; Ludwig, T.; Gerald, W.; Cordon-Cardo, C.; Pandolfi, P. P., Crucial role of p53-dependent cellular senescence in suppression of Pten-deficient tumorigenesis. *Nature* **2005,** *436* (7051), 725-30.

109. Braig, M.; Lee, S.; Loddenkemper, C.; Rudolph, C.; Peters, A. H.; Schlegelberger, B.; Stein, H.; Dorken, B.; Jenuwein, T.; Schmitt, C. A., Oncogene-induced senescence as an initial barrier in lymphoma development. *Nature* **2005,** *436* (7051), 660-5.

110. Gao, Y. F.; Li, T.; Chang, Y.; Wang, Y. B.; Zhang, W. N.; Li, W. H.; He, K.; Mu, R.; Zhen, C.; Man, J. H.; Pan, X.; Li, T.; Chen, L.; Yu, M.; Liang, B.; Chen, Y.; Xia, Q.; Zhou, T.; Gong, W. L.; Li, A. L.; Li, H. Y.; Zhang, X. M., Cdk1-phosphorylated CUEDC2 promotes spindle checkpoint inactivation and chromosomal instability. *Nat Cell Biol* **2011,** *13* (8), 924-33.

111. Shih, S. C.; Prag, G.; Francis, S. A.; Sutanto, M. A.; Hurley, J. H.; Hicke, L., A ubiquitin-binding motif required for intramolecular monoubiquitylation, the CUE domain. *EMBO J* **2003,** *22* (6), 1273-81.

112. Hu, S.; Hu, Z.; Li, S.; He, C.; Wu, Y.; Teng, D.; Yan, Y.; Li, H.; Xing, X.; Zou, G.; Li, Y.; Yang, Y.; Wang, Y.; Du, X., Expression of CUEDC2 in colorectal cancer with different invasion and migration abilities. *J Int Med Res* **2019,** *47* (2), 905-914.

113. Zhang, P. J.; Zhao, J.; Li, H. Y.; Man, J. H.; He, K.; Zhou, T.; Pan, X.; Li, A. L.; Gong, W. L.; Jin, B. F.; Xia, Q.; Yu, M.; Shen, B. F.; Zhang, X. M., CUE domain containing 2 regulates degradation of progesterone receptor by ubiquitin-proteasome. *EMBO J* **2007,** *26* (7), 1831-42.

114. Pan, X.; Zhou, T.; Tai, Y. H.; Wang, C.; Zhao, J.; Cao, Y.; Chen, Y.; Zhang, P. J.; Yu, M.; Zhen, C.; Mu, R.; Bai, Z. F.; Li, H. Y.; Li, A. L.; Liang, B.; Jian, Z.; Zhang, W. N.; Man, J. H.; Gao, Y. F.; Gong, W. L.; Wei, L. X.; Zhang, X. M., Elevated expression of CUEDC2 protein confers endocrine resistance in breast cancer. *Nat Med* **2011,** *17* (6), 708-14.

115. Musgrove, E. A., Estrogen receptor degradation: a CUE for endocrine resistance? *Breast Cancer Res* **2011,** *13* (4), 312.

116. Li, F.; Tang, C.; Jin, D.; Guan, L.; Wu, Y.; Liu, X.; Wu, X.; Wu, Q. Y.; Gao, D., CUEDC2 suppresses glioma tumorigenicity by inhibiting the activation of STAT3 and NF-kappaB signaling pathway. *Int J Oncol* **2017,** *51* (1), 115-127.

117. Li, H. Y.; Liu, H.; Wang, C. H.; Zhang, J. Y.; Man, J. H.; Gao, Y. F.; Zhang, P. J.; Li, W. H.; Zhao, J.; Pan, X.; Zhou, T.; Gong, W. L.; Li, A. L.; Zhang, X. M., Deactivation of the kinase IKK by CUEDC2 through recruitment of the phosphatase PP1. *Nat Immunol* **2008,** *9* (5), 533-41.

118. Kumar, A.; Kumar, A.; Ingle, H.; Kumar, S.; Mishra, R.; Verma, M. K.; Biswas, D.; Kumar, N. S.; Mishra, A.; Raut, A. A.; Takaoka, A.; Kumar, H., MicroRNA hsa-miR-324-5p Suppresses H5N1 Virus Replication by Targeting the Viral PB1 and Host CUEDC2. *J Virol* **2018,** *92* (19).

119. Zhang, W. N.; Wang, L.; Wang, Q.; Luo, X.; Fang, D. F.; Chen, Y.; Pan, X.; Man, J. H.; Xia, Q.; Jin, B. F.; Li, W. H.; Li, T.; Liang, B.; Chen, L.; Gong, W. L.; Yu, M.; Li, A. L.; Zhou, T.; Li, H. Y., CUEDC2 (CUE domain-containing 2) and SOCS3 (suppressors of cytokine signaling 3) cooperate to negatively regulate Janus kinase 1/signal transducers and activators of transcription 3 signaling. *J Biol Chem* **2012,** *287* (1), 382-92.

120. Zhang, W. N.; Zhou, J.; Zhou, T.; Li, A. L.; Wang, N.; Xu, J. J.; Chang, Y.; Man, J. H.; Pan, X.; Li, T.; Li, W. H.; Mu, R.; Liang, B.; Chen, L.; Jin, B. F.; Xia, Q.; Gong, W. L.; Zhang, X. M.; Wang, L.; Li, H. Y., Phosphorylation-triggered CUEDC2 degradation promotes UV-induced G1 arrest through APC/C(Cdh1) regulation. *Proc Natl Acad Sci U S A* **2013,** *110* (27), 11017-22.

121. Otake, K.; Kamiguchi, H.; Hirozane, Y., Identification of biomarkers for amyotrophic lateral sclerosis by comprehensive analysis of exosomal mRNAs in human cerebrospinal fluid. *BMC Med Genomics* **2019,** *12* (1), 7.

122. Ferraiuolo, L.; Kirby, J.; Grierson, A. J.; Sendtner, M.; Shaw, P. J., Molecular pathways of motor neuron injury in amyotrophic lateral sclerosis. *Nat Rev Neurol* **2011,** *7* (11), 616-30.

123. Riancho, J.; Gonzalo, I.; Ruiz-Soto, M.; Berciano, J., Why do motor neurons degenerate? Actualization in the pathogenesis of amyotrophic lateral sclerosis. *Neurologia* **2019,** *34* (1), 27-37.

124. Huber, V.; Vallacchi, V.; Fleming, V.; Hu, X.; Cova, A.; Dugo, M.; Shahaj, E.; Sulsenti, R.; Vergani, E.; Filipazzi, P.; De Laurentiis, A.; Lalli, L.; Di Guardo, L.; Patuzzo, R.; Vergani, B.; Casiraghi, E.; Cossa, M.; Gualeni, A.; Bollati, V.; Arienti, F.; De Braud, F.; Mariani, L.; Villa, A.; Altevogt, P.; Umansky, V.; Rodolfo, M.; Rivoltini, L., Tumor-derived microRNAs induce myeloid suppressor cells and predict immunotherapy resistance in melanoma. *J Clin Invest* **2018,** *128* (12), 5505-5516.

125. Lima, C. R.; Geraldo, M. V.; Fuziwara, C. S.; Kimura, E. T.; Santos, M. F., MiRNA-146b-5p upregulates migration and invasion of different Papillary Thyroid Carcinoma cells. *BMC Cancer* **2016,** *16*, 108.

126. Deng, X.; Wu, B.; Xiao, K.; Kang, J.; Xie, J.; Zhang, X.; Fan, Y., MiR-146b-5p promotes metastasis and induces epithelial-mesenchymal transition in thyroid cancer by targeting ZNRF3. *Cell Physiol Biochem* **2015,** *35* (1), 71-82.

127. Huang, X.; Zhong, R.; He, X.; Deng, Q.; Peng, X.; Li, J.; Luo, X., Investigations on the mechanism of progesterone in inhibiting endometrial cancer cell cycle and viability via regulation of long noncoding RNA NEAT1/microRNA-146b-5p mediated Wnt/beta-catenin signaling. *IUBMB Life* **2019,** *71* (2), 223-234.

128. Elsarraj, H. S.; Hong, Y.; Valdez, K.; Carletti, M.; Salah, S. M.; Raimo, M.; Taverna, D.; Prochasson, P.; Bharadwaj, U.; Tweardy, D. J.; Christenson, L. K.; Behbod, F., A novel role of microRNA146b in promoting mammary alveolar progenitor cell maintenance. *J Cell Sci* **2013,** *126* (Pt 11), 2446-58.

129. Venkatesan, N.; Kanwar, J.; Deepa, P. R.; Khetan, V.; Crowley, T. M.; Raguraman, R.; Sugneswari, G.; Rishi, P.; Natarajan, V.; Biswas, J.; Krishnakumar, S., Clinico-Pathological Association of Delineated miRNAs in Uveal Melanoma with Monosomy 3/Disomy 3 Chromosomal Aberrations. *PLoS One* **2016,** *11* (1), e0146128.

130. Shah, M.; Zedek, D., Piebaldism. In *StatPearls*, Treasure Island (FL), 2020.

131. Sun, G.; Liang, X.; Qin, K.; Qin, Y.; Shi, X.; Cong, P.; Mo, D.; Liu, X.; Chen, Y.; He, Z., Functional Analysis of KIT Gene Structural Mutations Causing the Porcine Dominant White Phenotype Using Genome Edited Mouse Models. *Front Genet* **2020,** *11*, 138.

132. Johansson Moller, M.; Chaudhary, R.; Hellmen, E.; Hoyheim, B.; Chowdhary, B.; Andersson, L., Pigs with the dominant white coat color phenotype carry a duplication of the KIT gene encoding the mast/stem cell growth factor receptor. *Mamm Genome* **1996,** *7* (11), 822-30.

133. Hafliger, I. M.; Hirter, N.; Paris, J. M.; Wolf Hofstetter, S.; Seefried, F. R.; Drogemuller, C., A de novo germline mutation of KIT in a white-spotted Brown Swiss cow. *Anim Genet* **2020,** *51* (3), 449-452.

134. Kommadath, A.; Grant, J. R.; Krivushin, K.; Butty, A. M.; Baes, C. F.; Carthy, T. R.; Berry, D. P.; Stothard, P., A large interactive visual database of copy number variants discovered in taurine cattle. *Gigascience* **2019,** *8* (6).

135. Keel, B. N.; Keele, J. W.; Snelling, W. M., Genome-wide copy number variation in the bovine genome detected using low coverage sequence of popular beef breeds. *Anim Genet* **2017,** *48* (2), 141-150.

136. Fontanesi, L.; Tazzoli, M.; Russo, V.; Beever, J., Genetic heterogeneity at the bovine KIT gene in cattle breeds carrying different putative alleles at the spotting locus. *Anim Genet* **2010,** *41* (3), 295-303.

137. Norris, B. J.; Whan, V. A., A gene duplication affecting expression of the ovine ASIP gene is responsible for white and black sheep. *Genome Res* **2008,** *18* (8), 1282-93.

138. Hauswirth, R.; Jude, R.; Haase, B.; Bellone, R. R.; Archer, S.; Holl, H.; Brooks, S. A.; Tozaki, T.; Penedo, M. C.; Rieder, S.; Leeb, T., Novel variants in the KIT and PAX3 genes in horses with white-spotted coat colour phenotypes. *Anim Genet* **2013,** *44* (6), 763-5.

139. Otsuki, Y.; Okuda, Y.; Naruse, K.; Saya, H., Identification of kit-ligand a as the Gene Responsible for the Medaka Pigment Cell Mutant few melanophore. *G3 (Bethesda)* **2020,** *10* (1), 311-319.

140. Torigoe, D.; Ichii, O.; Dang, R.; Ohnaka, T.; Okano, S.; Sasaki, N.; Kon, Y.; Agui, T., High-resolution linkage mapping of the rat hooded locus. *J Vet Med Sci* **2011,** *73* (5), 707-10.

141. Kuramoto, T.; Nakanishi, S.; Ochiai, M.; Nakagama, H.; Voigt, B.; Serikawa, T., Origins of albino and hooded rats: implications from molecular genetic analysis across modern laboratory rat strains. *PLoS One* **2012,** *7* (8), e43059.

142. Vaher, H.; Kivihall, A.; Runnel, T.; Raam, L.; Prans, E.; Maslovskaja, J.; Abram, K.; Kaldvee, B.; Mrowietz, U.; Weidinger, S.; Kingo, K.; Rebane, A., SERPINB2 and miR-146a/b are coordinately regulated and act in the suppression of psoriasis-associated inflammatory responses in keratinocytes. *Exp Dermatol* **2020,** *29* (1), 51-60.

143. Hermann, H.; Runnel, T.; Aab, A.; Baurecht, H.; Rodriguez, E.; Magilnick, N.; Urgard, E.; Sahmatova, L.; Prans, E.; Maslovskaja, J.; Abram, K.; Karelson, M.; Kaldvee, B.; Reemann, P.; Haljasorg, U.; Ruckert, B.; Wawrzyniak, P.; Weichenthal, M.; Mrowietz, U.; Franke, A.; Gieger, C.; Barker, J.; Trembath, R.; Tsoi, L. C.; Elder, J. T.; Tkaczyk, E. R.; Kisand, K.; Peterson, P.; Kingo, K.; Boldin, M.; Weidinger, S.; Akdis, C. A.; Rebane, A., miR-146b Probably Assists miRNA-146a in the Suppression of Keratinocyte Proliferation and Inflammatory Responses in Psoriasis. *J Invest Dermatol* **2017,** *137* (9), 1945-1954.

144. Zhou, B.; Zuo, X. X.; Li, Y. S.; Gao, S. M.; Dai, X. D.; Zhu, H. L.; Luo, H., Integration of microRNA and mRNA expression profiles in the skin of systemic sclerosis patients. *Sci Rep* **2017,** *7*, 42899.

145. Shams, K.; Kurowska-Stolarska, M.; Schutte, F.; Burden, A. D.; McKimmie, C. S.; Graham, G. J., MicroRNA-146 and cell trauma down-regulate expression of the psoriasis-associated atypical chemokine receptor ACKR2. *J Biol Chem* **2018,** *293* (8), 3003-3012.

146. Yasunaga, M.; Saijou, S.; Hanaoka, S.; Anzai, T.; Tsumura, R.; Matsumura, Y., Significant antitumor effect of an antibody against TMEM180, a new colorectal cancer-specific molecule. *Cancer Sci* **2019,** *110* (2), 761-770.

147. Mei, S.; Ke, J.; Tian, J.; Ying, P.; Yang, N.; Wang, X.; Zou, D.; Peng, X.; Yang, Y.; Zhu, Y.; Gong, Y.; Zhong, R.; Chang, J.; Miao, X., A functional variant in the boundary of a topological association domain is associated with pancreatic cancer risk. *Mol Carcinog* **2019,** *58* (10), 1855-1862.

148. Anzai, T.; Matsumura, Y., Topological analysis of TMEM180, a newly identified membrane protein that is highly expressed in colorectal cancer cells. *Biochem Biophys Res Commun* **2019,** *520* (3), 566-572.

149. Gunnarsson, U.; Hellstrom, A. R.; Tixier-Boichard, M.; Minvielle, F.; Bed'hom, B.; Ito, S.; Jensen, P.; Rattink, A.; Vereijken, A.; Andersson, L., Mutations in SLC45A2 cause plumage color variation in chicken and Japanese quail. *Genetics* **2007,** *175* (2), 867-77.

150. Schmutz, S. M.; Berryere, T. G., The genetics of cream coat color in dogs. *J Hered* **2007,** *98* (5), 544-8.

151. Reinders, A.; Ward, J. M., Investigating polymorphisms in membrane-associated transporter protein SLC45A2, using sucrose transporters as a model. *Mol Med Rep* **2015,** *12* (1), 1393-8.

152. Dooley, C. M.; Schwarz, H.; Mueller, K. P.; Mongera, A.; Konantz, M.; Neuhauss, S. C.; Nusslein-Volhard, C.; Geisler, R., Slc45a2 and V-ATPase are regulators of melanosomal pH homeostasis in zebrafish, providing a mechanism for human pigment evolution and disease. *Pigment Cell Melanoma Res* **2013,** *26* (2), 205-17.

153. Newton, J. M.; Cohen-Barak, O.; Hagiwara, N.; Gardner, J. M.; Davisson, M. T.; King, R. A.; Brilliant, M. H., Mutations in the human orthologue of the mouse underwhite gene (uw) underlie a new form of oculocutaneous albinism, OCA4. *Am J Hum Genet* **2001,** *69* (5), 981-8.

154. Graf, J.; Voisey, J.; Hughes, I.; van Daal, A., Promoter polymorphisms in the MATP (SLC45A2) gene are associated with normal human skin color variation. *Hum Mutat* **2007,** *28* (7), 710-7.

155. Aldahmesh, M. A.; Al-Hassnan, Z. N.; Aldosari, M.; Alkuraya, F. S., Neuronal ceroid lipofuscinosis caused by MFSD8 mutations: a common theme emerging. *Neurogenetics* **2009,** *10* (4), 307-11.

156. Abdelmohsen, K.; Srikantan, S.; Yang, X.; Lal, A.; Kim, H. H.; Kuwano, Y.; Galban, S.; Becker, K. G.; Kamara, D.; de Cabo, R.; Gorospe, M., Ubiquitin-mediated proteolysis of HuR by heat shock. *EMBO J* **2009,** *28* (9), 1271-82.

157. Moughamian, A. J.; Osborn, G. E.; Lazarus, J. E.; Maday, S.; Holzbaur, E. L., Ordered recruitment of dynactin to the microtubule plus-end is required for efficient initiation of retrograde axonal transport. *J Neurosci* **2013,** *33* (32), 13190-203.

158. Vancoillie, G.; Lambert, J.; Haeghen, Y. V.; Westbroek, W.; Mulder, A.; Koerten, H. K.; Mommaas, A. M.; Van Oostveldt, P.; Naeyaert, J. M., Colocalization of dynactin subunits P150Glued and P50 with melanosomes in normal human melanocytes. *Pigment Cell Res* **2000,** *13* (6), 449-57.

159. Deacon, S. W.; Serpinskaya, A. S.; Vaughan, P. S.; Lopez Fanarraga, M.; Vernos, I.; Vaughan, K. T.; Gelfand, V. I., Dynactin is required for bidirectional organelle transport. *J Cell Biol* **2003,** *160* (3), 297-301.

160. Aspengren, S.; Wallin, M., A role for spectrin in dynactin-dependent melanosome transport in Xenopus laevis melanophores. *Pigment Cell Res* **2004,** *17* (3), 295-301.

161. Lee, C.; Le, M. P.; Cannatella, D.; Wallingford, J. B., Changes in localization and expression levels of Shroom2 and spectrin contribute to variation in amphibian egg pigmentation patterns. *Dev Genes Evol* **2009,** *219* (6), 319-30.

162. Ohbayashi, N.; Maruta, Y.; Ishida, M.; Fukuda, M., Melanoregulin regulates retrograde melanosome transport through interaction with the RILP-p150Glued complex in melanocytes. *J Cell Sci* **2012,** *125* (Pt 6), 1508-18.

163. Skoda, A. M.; Simovic, D.; Karin, V.; Kardum, V.; Vranic, S.; Serman, L., The role of the Hedgehog signaling pathway in cancer: A comprehensive review. *Bosn J Basic Med Sci* **2018,** *18* (1), 8-20.

164. Li, J.; Li, P.; Carr, A.; Wang, X.; DeLaPaz, A.; Sun, L.; Lee, E.; Tomei, E.; Li, L., Functional expression of SCL/TAL1 interrupting locus (Stil) protects retinal dopaminergic cells from neurotoxin-induced degeneration. *J Biol Chem* **2013,** *288* (2), 886-93.

165. Li, P.; He, Y.; Cai, G.; Xiao, F.; Yang, J.; Li, Q.; Chen, X., CCDC114 is mutated in patient with a complex phenotype combining primary ciliary dyskinesia, sensorineural deafness, and renal disease. *J Hum Genet* **2019,** *64* (1), 39-48.

166. Carr, A. L.; Sun, L.; Lee, E.; Li, P.; Antonacci, C.; Gorbea, E.; Finlay, C.; Li, L., The human oncogene SCL/TAL1 interrupting locus is required for mammalian dopaminergic cell proliferation through the Sonic hedgehog pathway. *Cell Signal* **2014,** *26* (2), 306-12.

167. Choi, H.; Shin, J. H.; Kim, E. S.; Park, S. J.; Bae, I. H.; Jo, Y. K.; Jeong, I. Y.; Kim, H. J.; Lee, Y.; Park, H. C.; Jeon, H. B.; Kim, K. W.; Lee, T. R.; Cho, D. H., Primary Cilia Negatively Regulate Melanogenesis in Melanocytes and Pigmentation in a Human Skin Model. *PLoS One* **2016,** *11* (12), e0168025.

168. Wang, Z. C.; Gao, J.; Zi, S. M.; Yang, M.; Du, P.; Cui, L., Aberrant expression of sonic hedgehog pathway in colon cancer and melanosis coli. *J Dig Dis* **2013,** *14* (8), 417-24.

169. Guerrini-Rousseau, L.; Dufour, C.; Varlet, P.; Masliah-Planchon, J.; Bourdeaut, F.; Guillaud-Bataille, M.; Abbas, R.; Bertozzi, A. I.; Fouyssac, F.; Huybrechts, S.; Puget, S.; Bressac-De Paillerets, B.; Caron, O.; Sevenet, N.; Dimaria, M.; Villebasse, S.; Delattre, O.; Valteau-Couanet, D.; Grill, J.; Brugieres, L., Germline SUFU mutation carriers and medulloblastoma: clinical characteristics, cancer risk, and prognosis. *Neuro Oncol* **2018,** *20* (8), 1122-1132.

170. Aavikko, M.; Li, S. P.; Saarinen, S.; Alhopuro, P.; Kaasinen, E.; Morgunova, E.; Li, Y.; Vesanen, K.; Smith, M. J.; Evans, D. G.; Poyhonen, M.; Kiuru, A.; Auvinen, A.; Aaltonen, L. A.; Taipale, J.; Vahteristo, P., Loss of SUFU function in familial multiple meningioma. *Am J Hum Genet* **2012,** *91* (3), 520-6.

171. Taylor, M. D.; Liu, L.; Raffel, C.; Hui, C. C.; Mainprize, T. G.; Zhang, X.; Agatep, R.; Chiappa, S.; Gao, L.; Lowrance, A.; Hao, A.; Goldstein, A. M.; Stavrou, T.; Scherer, S. W.; Dura, W. T.; Wainwright, B.; Squire, J. A.; Rutka, J. T.; Hogg, D., Mutations in SUFU predispose to medulloblastoma. *Nat Genet* **2002,** *31* (3), 306-10.

172. Harris, C. J.; Davis, B. A.; Zweig, J. A.; Nevonen, K. A.; Quinn, J. F.; Carbone, L.; Gray, N. E., Age-Associated DNA Methylation Patterns Are Shared Between the Hippocampus and Peripheral Blood Cells. *Front Genet* **2020,** *11*, 111.

173. Toniato, E.; Chen, X. P.; Losman, J.; Flati, V.; Donahue, L.; Rothman, P., TRIM8/GERP RING finger protein interacts with SOCS-1. *J Biol Chem* **2002,** *277* (40), 37315-22.

174. Caratozzolo, M. F.; Micale, L.; Turturo, M. G.; Cornacchia, S.; Fusco, C.; Marzano, F.; Augello, B.; D'Erchia, A. M.; Guerrini, L.; Pesole, G.; Sbisa, E.; Merla, G.; Tullo, A., TRIM8 modulates p53 activity to dictate cell cycle arrest. *Cell Cycle* **2012,** *11* (3), 511-23.

175. Caratozzolo, M. F.; Marzano, F.; Mastropasqua, F.; Sbisa, E.; Tullo, A., TRIM8: Making the Right Decision between the Oncogene and Tumour Suppressor Role. *Genes (Basel)* **2017,** *8* (12).

176. Venuto, S.; Castellana, S.; Monti, M.; Appolloni, I.; Fusilli, C.; Fusco, C.; Pucci, P.; Malatesta, P.; Mazza, T.; Merla, G.; Micale, L., TRIM8-driven transcriptomic profile of neural stem cells identified glioma-related nodal genes and pathways. *Biochim Biophys Acta Gen Subj* **2019,** *1863* (2), 491-501.

177. Zhang, C.; Mukherjee, S.; Tucker-Burden, C.; Ross, J. L.; Chau, M. J.; Kong, J.; Brat, D. J., TRIM8 regulates stemness in glioblastoma through PIAS3-STAT3. *Mol Oncol* **2017,** *11* (3), 280-294.

178. Tomar, D.; Sripada, L.; Prajapati, P.; Singh, R.; Singh, A. K.; Singh, R., Nucleo-cytoplasmic trafficking of TRIM8, a novel oncogene, is involved in positive regulation of TNF induced NF-kappaB pathway. *PLoS One* **2012,** *7* (11), e48662.

179. Okumura, F.; Matsunaga, Y.; Katayama, Y.; Nakayama, K. I.; Hatakeyama, S., TRIM8 modulates STAT3 activity through negative regulation of PIAS3. *J Cell Sci* **2010,** *123* (Pt 13), 2238-45.

180. Li, Q.; Yan, J.; Mao, A. P.; Li, C.; Ran, Y.; Shu, H. B.; Wang, Y. Y., Tripartite motif 8 (TRIM8) modulates TNFalpha- and IL-1beta-triggered NF-kappaB activation by targeting TAK1 for K63-linked polyubiquitination. *Proc Natl Acad Sci U S A* **2011,** *108* (48), 19341-6.

181. Okumura, F.; Okumura, A. J.; Matsumoto, M.; Nakayama, K. I.; Hatakeyama, S., TRIM8 regulates Nanog via Hsp90beta-mediated nuclear translocation of STAT3 in embryonic stem cells. *Biochim Biophys Acta* **2011,** *1813* (10), 1784-92.

182. Niwa, H.; Burdon, T.; Chambers, I.; Smith, A., Self-renewal of pluripotent embryonic stem cells is mediated via activation of STAT3. *Genes Dev* **1998,** *12* (13), 2048-60.

183. Venuto, S.; Monteonofrio, L.; Cozzolino, F.; Monti, M.; Appolloni, I.; Mazza, T.; Canetti, D.; Giambra, V.; Panelli, P.; Fusco, C.; Squeo, G. M.; Croce, A. I.; Pucci, P.; Malatesta, P.; Soddu, S.; Merla, G.; Micale, L., TRIM8 interacts with KIF11 and KIFC1 and controls bipolar spindle formation and chromosomal stability. *Cancer Lett* **2020,** *473*, 98-106.
