# Supplementary material for: Fifteen Shades of Grey: Combined Analysis of Genome-Wide SNP Data in Steppe and Mediterranean Grey Cattle Sheds New Light on the Molecular Basis of Coat Color
Source: Genes (Basel). 2020 Aug 13;11(8):932. doi: 10.3390/genes11080932 (PMC7464420; doi:10.3390/genes11080932)
Supplement: Supplementary file 1 [file genes-11-00932-s001.zip › Supplementary file S5.docx]

**Supplementary file S5.** Functions of genes located in the interval delimited by the SNPs BTB-01532239 and Hapmap27934-BTC-065223 on BTA14, in addition to the genes mentioned in Supplementary file S3.

***RPS20*** (Ribosomal Protein S20) his gene encodes a ribosomal protein that is a component of the 40S subunit. The protein belongs to the S10P family of ribosomal proteins. It is located in the cytoplasm. This gene is co-transcribed with the small nucleolar RNA gene U54, which is located in its second intron. Krishnan et al. [1] showed that interplay between GNL1 and RPS20 promotes phosphorylation of retinoblastoma protein (Rb) which in-turn modulate G1/S phase of the **cell division** cycle. RPS20 was detected in a global **ubiquitination** screening of primary human cells undergoing oncogene-induced **senescence** (OIS). Pathway analysis revealed that the OIS-induced ubiquitinome alterations mainly affected 3 signaling networks: eIF2 signaling, eIF4/p70S6K signaling, and mTOR signaling. Interestingly, the majority of the changed ubiquitinated proteins in these pathways belong to the **translation machinery**. This included several translation initiation factors (eIF2C2, eIF2B4, eIF3I, eIF3L, eIF4A1) and elongation factors (eEF1G, eEF1A) as well as 40S (RPS4X, RPS7, RPS11 and RPS20) and 60S ribosomal subunits (RPL10, RPL11, RPL18 and RPL35a). These results suggest that ubiquitination affects key components of the translation machinery to regulate protein synthesis during OIS, pointing toward ubiquitination as a hitherto unappreciated regulatory mechanism during OIS [2]. **Ubiquitination** of RPS20 was also found to trigger the **ribosome-associated quality control** (RQC) pathway, which senses the state of translation (e.g., when translation stalls), targets collided ribosomes and leads to subunit dissociation, followed by proteasomal degradation of the nascent peptide [3-6]. Daftuar et al. [7] identified RPS20 as a ribosomal protein able to bind to and **regulate** Mdm2 and MdmX, and thereby **p53**. p53 is an important **tumor suppressor** in cells, and its loss or mutation has been implicated in at least half of all human **cancers**. Molecularly, p53 is a **transcription factor** that stimulates expression of numerous target genes **in response to stress**. Levels of p53 are tightly regulated by Mdm2, a RING-type **E3 ubiquitin ligase** that binds to the N-terminal transactivation domain of p53 via sequences within its own N-terminal region. Mdm2 both inhibits p53 transactivation of its target genes and ubiquitinates lysines within the p53 C-terminus. Mdm2-mediated ubiquitination targets p53 for nuclear export and degradation by the **proteasome**. p53 activity is also regulated by MdmX, a homolog of Mdm2 that also contains a N-terminal p53-binding domain and a C-terminal RING domain. Just as with Mdm2, binding of the MdmX N-terminus to p53 inhibits its transactivation activity, but in the case of MdmX, its RING domain does not function to ubiquitinate p53. Rather, MdmX forms hetero-oligomers with Mdm2 in cells and likely directs Mdm2 RING activity towards p53 ubiquitination and away from Mdm2 auto-ubiquitination. Upon some forms of cellular stress, MdmX is degraded, thus releasing p53 from inhibition, and Mdm2 and p53 are modified so that Mdm2 cannot bind to p53 and target it for degradation. Both mechanisms allow for a buildup of active p53 and **arrest of the cell cycle** or, depending on the extent of the damage or cellular context, **apoptosis**. The accumulation of p53 also stimulates expression of Mdm2, thus completing an important negative feedback loop whereby p53 is eventually degraded once the stress has passed. p53 can be **stabilized** in an Mdm2-dependent manner by ectopic expression of various **ribosomal proteins** These RPs all bind to the central region of Mdm2 and inhibit its E3 ubiquitin ligase activity, leading to the activation of p53 [7].

Interestingly, McGowan et al. [8] reported two mouse **dark skin** loci caused by mutations in Rps19 (ribosomal protein S19) and Rps20 (ribosomal protein S20) and identified a common pathophysiologic program in which **p53 stabilization stimulates Kit ligand expression, and, consequently, epidermal melanocytosis** via a paracrine mechanism.

Besides MDM2, two critical proteins that are associated with **stress resistance**—superoxide dismutase (SOD1) and ubiquitin C (UBC)-, are also linked with the RPS20 protein network (S2B Fig). **SOD1** is a vital detoxifying enzyme and a major **antioxidant** system, and is considered an **anti-aging** factor ^9^. Likewise, **UBC** is typically referred to as a **stress-inducible gene** that can adequately increase expression levels when cells are challenged with different types of stress; presumably **to correct protein misfolding or to degrade damaged proteins** allowing them to survive under toxic stress conditions. These findings suggest that upregulation of RPS20 contributes to an **anti-stress phenotype** by enhancing genomic and cellular stability (discussed by Yong et al. [10] who also reported RPS20 as a stress-response marker of **glioblastoma stem cells**, and predictor of poor prognosis in glioblastoma patients).

Goldstone and Lavin [11] described the RPS20 cDNA as downregulated fivefold in association with the induction of **apoptosis** in a human leukaemic cell line and showed that the expression of the S20 mRNA was downregulated early during the induction of apoptosis in their model system, prior to the onset of DNA fragmentation and other morphological changes associated with cell death, suggesting some degree of involvement of RPS20 in the biochemical events that occur during the onset of cell death.

A polymorphism ablating a polyadenylation signal of the gene encoding ribosomal protein S20 was found to be highly significantly associated with **calving ease** in a *Bos taurus* German Fleckvieh population [12]. RPS20 was also detected within a GWAS for age at first calving under different environmental conditions in Nellore (***B. indicus***) heifers [13], within a GWAS for birth weight in Nellore (***B. indicus***) cattle [14], as well as within a selection signature for stature and body size in Angus cattle [15].

***LOC112449628*** Uncharacterized locus

***LOC112449630*** Uncharacterized locus

***MOS*** (MOS Proto-Oncogene, Serine/Threonine Kinase) encodes a serine/threonine kinase that activates the **MAP kinase** cascade through direct phosphorylation of the MAP kinase activator **MEK**. Indeed, MOS has been shown to cause persistent activation of the extracellular signal-regulated protein kinase (ERK) both in and in vitro. ERK is a member of a mitogen-activated protein kinase (MAPK) family, a group of serine/threonine protein kinases that includes **c-Jun** amino-terminal kinase (**JNK**) and p38 subgroups as other members. In response to a variety of mitogenic stimuli, ERKI (p44MAPK) and ERK2 (p42MAPK), the best characterized ERK isoforms in vertebrate cells, are rapidly and transiently activated, translocated to the nucleus, and play a crucial role in entry of quiescent cells into the cell cycle. This rapid rise of ERK activity is due to the activation of MAPK/ERK-activating kinases (**MEKs**), a group of dual specificity protein kinases that directly phosphorylate ERKs on both threonine and tyrosine residues. MEKs themselves are activated through direct phosphorylation on two serine residues by MAP kinase kinase kinases (MAPKKKs), which include the proto-oncogene product c-Raf. Consistent with the persistent ERK activation in Mos-transformed cells, Mos also functions as MAPKKK and activates Mekl, one of the MEK isoforms, suggesting that Mos may utilize the Mekl/ERK pathway to transform cells (discussed by Okazaki and Sagata [16], who also showed that **c-Fos** can be metabolically stabilized, and mediate, at least in part, the oncogenic signalling by the Mos/ERK pathway). Mitogen-activated protein kinase (MAPK) pathways are involved in diverse cellular processes including **proliferation**, **cell death**, **differentiation** and **development**.

Besides germ cells, MOS has been shown to be expressed in various somatic cell lineages and tissues [17,18]. Constitutive expression of MOS has been shown to induce **oncogenic transformation** *in vitro* possibly via activation of **c-fos, c-jun** and **c-myc**, and MOS overexpression has been associated with **chromosomal instability** [19]. Also, **inappropriate microtubule reorganization** mediated by MOS during interphase could explain both the neoplastic morphological alterations observed at low MOS levels and the dramatic impairment of cell cycle and cell morphology resulting from high concentrations of MOS [20].
Paulino et al. [21] evaluate the effects of EGF, P4, and both EGF and P4 on growth, viability and ultrastructure of bovine secondary follicles as well as on the levels of mRNA expression for GDF9, MOS, H1foo, cyclin B1, PARN and eIF4E in oocytes of secondary follicles cultured *in vitro*. They found that EGF, but not progesterone, promoted the development of secondary follicles cultured *in vitro* and increased the expression of MOS and GDF9, indicative of oocyte competence to assure oocyte maturation and embryo development. The MOS kinase has been shown to play a crucial role in the **control of meiosis in oocytes** in addition to regulating cell survival and apoptosis [22].

Leibovitch et al. previously showed that the c‐mos proto‐oncogene product, serine/threonine kinase Mos, is up‐regulated during **skeletal muscle development** [23,24]. Further, they showed that MOS increases the ability of **MyoD** to transactivate both muscle‐specific genes and its own promoter and could therefore participate in the positive autoregulation loop of MyoD and **muscle differentiation** [25].

Several evidences highlight the **relevance of the MAPK pathway in pigment biology and pathology**, some of which are not-exhaustively presented in what follows.

Syntenin has been shown to regulate **melanogenesis** via the **p38 MAPK** pathway [26]. The **anti-melanogenic** effect of *Dendropanax morbiferus* and its active components was shown to act via protein kinase a/cyclic adenosine monophosphate-responsive binding protein- and **p38** **mitogen-activated protein kinase**-mediated microphthalmia-associated transcription factor downregulation [27]. **Anti-melanogenic** effects of *Annona squamosa* L. leaves were shown to occur through inhibition of alpha-melanocyte-stimulating hormone (α-MSH) stimulated melanogenesis via **p38** signaling pathway in melanoma cells [28]. **Melanogenic** effects of maclurin have been shown to be mediated through the activation of cAMP/PKA/CREB and **p38 MAPK**/CREB signaling pathways. [29]. Fargesin has been shown to **inhibit melanin synthesis** in murine malignant and immortalized melanocytes by regulating PKA/CREB and **P38/MAPK** signaling pathways [30]. **P44/42 MAPK** signaling is a prime target activated by phenylethyl resorcinol in its **anti-melanogenic** action [31]. Kazinol U was shown to **inhibit melanogenesis** through the inhibition of tyrosinase-related proteins via phosphorylation of AMPK and **MAPK** proteins. Inhibition of Src kinase family (SKF) induces **melanogenesis** via the **MAPK** and CREB pathways [32]. The *Ganoderma lucidum* polysaccharide has been shown to **inhibit** **UVB-induced melanogenesis** by antagonizing cAMP/PKA and ROS/**MAPK** signaling pathways [33]. The mycotoxin beauvericin has been shown to **inhibit melanogenesis** by regulating cAMP/PKA/CREB and LXR-α/**p38 MAPK**-mediated pathways [34]. R-Fluoxetine has been shown to **increase melanin synthesis** through a 5-ht1a/2a receptor and **p38 MAPK** signaling pathways [35].

***PLAG1*** (PLAG1 Zinc Finger) encodes a **transcription factor** whose activation results in up-regulation of target genes, such as IGFII, leading to uncontrolled **cell proliferation**: when overexpressed in cultured cells, higher proliferation rate and transformation are observed. Other target genes such as CRLF1, CRABP2, CRIP2, PIGF are strongly induced in cells with PLAG1 induction. It is a proto-oncogene whose ectopic expression can trigger the development of pleomorphic adenomas of the salivary gland and lipoblastomas. Its overexpression, associated with up-regulation of IGFII, is frequently observed in hepatoblastoma, common primary liver tumor in childhood. Cooperates with CBFB-MYH11, a fusion gene important for myeloid leukemia. The transcription factor PLAG1 has been shown to play an important role in the **regulation of neurogenic potential** in mouse neocortical **neural progenitor cells** [36]. Over-expression of PLAG1 promoted and knockdown of PLAG1suppressed **neuronal differentiation** of neocortical neural progenitor cells *in vivo*. Transcriptomic analysis showed that PLAG1 increased the **expression of** a set of **neuronal genes** in neural progenitor cells [37,38] suggested that altered miR-218-1 regulation of SLIT2, RET and PLAG1 might be involved in the pathogenesis of Hirschsprung's disease, a rare congenital disease caused by **impaired proliferation and migration of neural crest cells**.

Mutation at *PLAG1* have been associated with **Silver-Russell syndrome** [39], an heterogenous syndrome characterized by dysmorphic features and, in rare instances, by **alteration of pigmentation** [40]. Notably, mutations of genes controlling IGF2 expression, such as PLAG1 and HMGA2, have been shown to be able to cause Silver-Russell syndrome [41].

Involvement of WNT pathway in PLAG1-related salivary gland adenomas has been shown [42,43].

PLAG1 has been shown to enhance the **stemness** profiles in the acinar cells of normal human salivary glands [44].

NFYA and PLAG1 were shown to be essential for basal transcriptional regulation of OSBPL2, an intracellular **transport protein** that binds **sterols** and **phospholipids** and mediates **lipid transport between intracellular compartments**. OSBPL2 has been shown to increase plasma membrane **cholesterol** levels and decreases **phosphatidylinositol-4,5-bisphosphate** levels in the cell membrane [45].

To generate energy and biomass for **tumor growth**, cancer cells are well documented to have **enhanced metabolic requirements**, including elevated aerobic glycolysis and **glutaminolysis.** PLAG1 has been shown to contribute to expression of a glutaminolytic enzyme, **glutamate dehydrogenase 1** (GDH1), whose product, α-ketoglutarate, has been shown to promote tumor growth by regulating **redox homeostasis** by activating a reactive oxygen species (**ROS) scavenging enzyme**, glutathione peroxidase 1. Mechanistically, the GDH1 product, α-ketoglutarate, was shown to activate the calcium/calmodulin-dependent protein kinase kinase 2 (**CamKK2**) involved in the calcium/calmodulin-dependent (CaM) kinase cascade, by enhancing its substrate **AMPK** binding, which contributes to **energy production** [46].

*PLAG1* was found in a signature of positive selection underlying beef production traits in Korean cattle breeds [47], in a signature of selection underlying morphological differences across 18 French cattle breeds [48], in a signature of selection underlying stature in a European cohort of cattle breeds [49]. Also, it was found in a peculiar selection signature for the Reggiana cattle breed [50], for Tanzanian crossbred cattle [51], for Angus and Holstein [52], for Limousin [53]. It was found in a QTL for direct calving difficulty in a Holstein Friesian cattle population [54] as well as within a GWAS for age at first calving under different environmental conditions in Nellore (***B. indicus***) heifers [13], a GWAS for *Longissimus* muscle area in a tropical composite (taurine x **indicine**) beef cattle population [9]. A 19-bp insertion/deletion (indel) in intron 1 of the *PLAG1* gene was shown to be related to the height of Chinese cattle breeds [55,56] and to growth traits of Chinese cattle breeds [57]. The gene was also shown to be associated with growth traits in Chinese cattle breeds [58,59] with stature in a U.S. Gelbvieh beef cattle population [60], with stature in Montbeliarde cattle breed [61], with stature and body size in Angus [15], with stature in a cross Holstein-Friesian × Jersey [62], with body measurement traits in Chinese Wagyu beef cattle [63], with carcass meat yield for both Chinese Simmental and Wagyu cattle breeds [64], for carcass traits in a Chinese Simmental cattle population [65], for carcass weight and intramuscular fat in Hanwoo Korean cattle [66], for carcass traits in ***Bos indicus*** Nellore cattle [67], for birth weight in Peranakan Ongole Indonesian ***Bos indicus*** cattle breed [68], for various body weights in **composite** Brangus, and in Gelbvieh and Simmental breeds [69], for scrotal circumference in ***Bos indicus*** Nellore cattle [70], for hip height, weight, net food intake, age at puberty in males and females, IGF-I concentration in blood and fat depth in *Bos taurus*, ***Bos indicus*** and tropical composite cattle in Australia [71], for birth weight in ***Bos indicus*** Nellore cattle [14], for bovine stature in Japanese Black cattle [72] for early life body weight and peripubertal weight and growth in Holstein Friesian, for body weight at 250 days of age in Australian and New Zealand cattle populations [73], and was shown to harbor an eQTL for growth and body weight as well as milk production traits, including milk fat, volume, and protein yield [74].

The bovine PLAG1 mutation with major effects on body size, weight and reproduction has been shown to be a >1,000 years old derived allele that increased rapidly in frequency in Northwestern European *B. taurus* between the 16th and 18th centuries. Towards the 19th and 20th centuries, the allele was introgressed into non-European *B. taurus* and *Bos indicus* breeds. These data implicate a major role of this allele in recent changes in body size in modern cattle, and represent one of the first examples of a genomic sweep in livestock that was driven by selection on a complex trait [75,76].

Signature of selection involving PLAG1 was also observed in eight indigenous Swiss sheep breeds [77].

***FAM110B*** (Family with Sequence Similarity 110 Member B) Also known as *C8orf72*, the gene is the founder member of the FAM110 family that colocalize with the centrosome and the Micro Tubule Organizing Center (MTOC) in interphase and at the spindle poles in mitosis. They have been implicated in biological functions related to the cell cycle [78] Naouar et al [79] found that transient downregulation of FAM110B was associated with downregulated Wnt, TGF-β and the Notch pathways, increased E-cadherin and decreased N-cadherin expression. Since loss of E-cadherin and activation of signaling pathways including Wnt/beta-catenin, TGF-β, and Notch are considered fundamental events in **epithelial to mesenchymal transition** (EMT), FAM110B seems to be a player in this process that, in vertebrates, drives the formation of migratory neural crest cells during embryonic development from the epithelial cells of the neuroectoderm. As a result, these cells dissociate from neural folds, gain motility, and disseminate to various parts of the embryo, where they differentiate to many other cell types [80], such as melanocytes. [81] reported FAM110B among the top 100 genes whose expression was found to be increased in human umbilical mesenchymal stem cells grown in a neuronal conditioned medium compared to those grown in a classical DMEM medium, suggesting a possible role in **neuronal differentiation**. Consistently with the above study, Xi and Zhang [82] found that FAM110B was associated with pancreatic adenocarcinoma tumor differentiation. Furthermore, FAM110B was highlighted as an **estradiol and androgen responsive gene** [83, 84] In Hanwoo cattle, Lee et al. [85] reported as associated with carcass weight a major chromosome region ranging from 23 Mb to 25 Mb on chromosome 14. Genes in that region that were closer to the most significant SNPs were, among other, *SCDBP* and *FAM110B*. Interestingly, the authors mention that the association study had been performed on an overall sample set composed by animals from three **Hanwoo breeds displaying different coat colors** (Korean brown, Korean brindle, and Jeju black) and that different allele frequencies and different LD patterns were observed among the three breeds in the mentioned chromosomic region.

***LOC101902490*** (60S ribosomal protein L39)

***UBXN2B*** (UBX Domain Protein 2B) The gene, also known as *P37*, codes for the UBX Domain Protein 2B, a cofactor of NSFL1 (also known as P97 or VSP, valosin-containing protein) responsible for its functional specificity. P97, one of the most abundant cytosolic ATPases, shows indeed a wide diversity of substrates; it couples ATP hydrolysis to segregation of polypeptides from immobile cellular structures such as protein assemblies, membranes, ribosome, and chromatin. This often results in proteasomal degradation of extracted polypeptides, with profound influence on various aspects of cellular physiology [86] such as **protein homeostasis** (protein quality control processes, like the endoplasmic reticulum-associated degradation, ERAD; extraction of polypeptides from the mitochondrial outer membrane to facilitate mitochondria-associated degradation; extraction of defective translation products stalled on ribosome; shuttling aberrant polypeptides to the proteasome for degradation; autophagy, a process of cellular protein turnover characterized by engulfment of cytosol into the autophagosome), **chromatin-associated functions** (linking P97 to diverse nuclear events ranging from gene transcription to DNA replication and repair), **membrane fusion and vesicular trafficking**. Notably, Ramanathan and Ye [87] demonstrated how p97 associates with EEA1 to regulate the size of early endosomes, that are known to contribute to the development of melanosomes [88]. Additional evidences point to implication of P97/P37 in fusion of vesicles that lead to the re-formation of the Golgi and endoplasmic reticulum apparatus at the end of mitosis [89]. In another study [90] P97/P37 was identified to play a role in ensuring proper spindle orientation by preventing the excessive recruitment of the NuMA (nuclear/mitotic apparatus protein) complex to the cortex in metaphase [91].

***CYP7A1*** (Cytochrome P450 Family 7 Subfamily A Member 1) The gene codes for **cholesterol 7α-hydroxylase**, which catalyzes the first and rate-limiting step in the classical **bile acid synthetic pathway**. It is mainly expressed in liver, in a circadian-dependent fashion [92, 93]. Together with other CYP family members, 7-hydroxylases, including CYP7A1, have been shown to be expressed in skin [94, 95] where they may produce 7-hydroxy/oxy-steroids/sterols. Oxysterols act as ligands for LXRs [96]. LXRs (liver X receptors) are expressed in skin [96,97] in hair follicles [98, 99] and in melanocytes [100]. In the melanocytes from perilesional skin of vitiligo patients, LXRs were upregulated suggesting that they might be involved in the **regulation of melanin production** [100]. Indeed, activation of LXR was shown to potently inhibits melanogenesis by lowering the expression of three key enzymes for melanin synthesis (TYR, TYRP1, DCT) associated to **accelerated MITF protein degradation**, in response to the activation of the MEK/ERK/RSK-1 signaling pathway [101]. LXR activation was also shown to induces **keratinocyte differentiation** [96, 102, 103] In addition, LXRs are known to heterodimerize with retinoid X receptors (RXRs) [104] and regulate a number of genes involved in controlling intracellular cholesterol trafficking, metabolism and balance in liver [105] and adrenal [106]. Since RXRs have been shown to be functionally expressed in skin [107], it could be assumed that a similar mechanism of homeostasis may be active in this compartment, where **cholesterol represents the precursor for local synthesis of vitamin D, androgens, estrogens, progesterone, gluco- and mineral-corticosteroids** [108]. Interestingly, studies have shown that both the precursor and the steroid derivatives may play a role in melanocyte biology. Schallreuter et al. [109] demonstrated that cholesterol increases **melanogenesis** in epidermal melanocytes, and this phenotype is associated with a cholesterol-dependent increase in cAMP levels and protein expression of ERß, CREB, MITF, THI and tyrosinase. In a recent paper, Cario [110] reviewed the ability of melanocytes to respond to **stimulation by estrogen and progesterone** with **altered patterns of pigmentation**. They highlighted how these hormones may act through both classical nuclear receptors (ER and PR) and membrane receptors, the latter respectively stimulating (GPER) and inhibiting (PAQR7) cAMP signaling [111]. Studies have shown that also **androgens may affect pigmentation** via reduction in the expression of MC1R (melanocortin 1 receptor, Scott et al. [112], or through decreased intracellular cAMP levels due to the SHBG-modulated steroid-activated signal transduction pathway [113]. It is noteworthy to mention that, **in skin, all major components of the hypothalamic–pituitary–adrenal (HPA) axis are produced** [114]. Pang et al. [115] provided evidence that chronic stress can **suppress the expression of skin HPA axis-related genes**, associated to **reduced pigmentation**, through a negative feedback control via high glucocorticoid concentrations induced by stress. Taken together, the above evidences suggest a complex scenario characterized by **close interconnections between the steroidogenic and melanogenic activities in skin**, with CYP7A1 possibly playing a role in modulating cholesterol/oxysterols availability.

***TRNAG-CCC*** (Transfer RNA Glycine) transfer RNA

***LOC112449629*** (U1 spliceosomal RNA) small nuclear RNA, component of the spliceosome.

***SDCBP*** (Syndecan Binding Protein) The Syndecan Binding Protein gene, also known as *MDA9* (melanoma differentiation associated gene 9), codes for Syntenin-1, a cytoplasmic scaffold protein that contains tandemly repeated PDZ domains, involved in **cytoskeletal-membrane organization, cell adhesion, protein and vesicular trafficking, and the activation of transcription factors**. SDCBP has been widely shown to contribute to **metastatic progression of melanoma** [116,117]. *SDCBP* is reported to be one of the genes that are highly **upregulated in the reservoir of stem cells in the mouse hair follicle bulge** [118]. It was first described in mouse embryonic melanoblasts by Colombo et al. [119] who reported a marked expression level. It was also observed displaying a tight regulation of its temporal and spatial expression in mouse embryonic skin by Jeon et al. [120], who suggested its involvement in **differentiation during fetal development**. More recently, the expression of *SDCBP*, and other genes known to affect melanocyte differentiation and melanogenesis, was shown to be **inhibited** by miR-155 **in melanocytes from patients with vitiligo** [121]. Bian et al. [122] identified *SDCBP* as one of the **dysregulated** genes **in grey compared to black hair follicles in human premature hair graying patients**. SDCBP interacts with the actin cytoskeleton and with cell adhesion receptors, including integrins and syndecans [123,124]. Syndecans (members 1 to 4) are cell surface heparan sulfate proteoglycans, playing a crucial role in binding extracellular ligands and in cell-matrix adhesion, cell–cell adhesion, migration and proliferation [125]. Syndecan-1 has been shown to participate in **melanin transport** [126]. Knockdown of syndecan-2 expression decreased MC1R-mediated melanoma cell migration [127]. Overexpression of syndecan-2 was shown to increase **melanin synthesis** both in melanoma cells and in human primary epidermal melanocytes via changes in the localization of PKCbII, known to phosphorylate, upon translocation from the cytosol to the plasma membrane, serine residues in the cytoplasmic domain of tyrosinase, a key enzyme in the melanogenesis process, thereby regulating its activity [128, 129].

***LOC112449508*** Uncharacterized locus

***NSMAF*** (Neutral Sphingomyelinase Activation Associated Factor) The gene, also known as *FAN* (factor associated with neutral sphingomyelinase activation), encodes a WD-repeat protein that binds the cytoplasmic neutral sphingomyelinase activation domain (NSD) of the tumor necrosis factor receptor 1 (TNFR1). The latter, upon activation by TNF-a, triggers several NSMAF-dependent signaling pathways, such as neutral sphingomyelinase activation (**sphingomyelin‐ceramide pathway**), **apoptosis signaling, inflammation and immune response, and cytoskeleton remodeling**. Ceramide-induced inactivation of Akt/PKB has been associated with **reduced cell proliferation in a melanocyte cell line, as well as reduced cell pigmentation** by inhibition of the tyrosinase activity [130]. Cytoskeletal events underlie both **melanoblast migration and dendrite formation by melanocytes**. During the embryonic life, melanoblasts migrate actively using a classical migration machinery that include the tubulin network, actin filaments and associated proteins [131], including Rac, Rho and Cdc42. TNF‐α‐induced activation of Rac and Rho was not altered by NSMAF deficiency, while Cdc42 was not activated in TNF‐α‐treated NSMAF‐deficient mouse embryonic fibroblasts, indicating the critical role of NSMAF in Cdc42 activation in response to TNF‐α [132]. Cdc42-null melanoblasts were shown to fail in fully populating the developing mouse embryo, and presented defects in cell-cycle progression and cytokinesis, likely contributing to the paucity of melanoblasts in Cdc42-null embryos and to the **coat color defects** of adults (i.e., a white patch running down the ventral midline, covering half to most of the underside, hypopigmented paws and tails) [133]. Loss of Cdc42 in the melanocyte lineage gave a phenotype characterized by defects in the organization and coordination of actin dynamics, contractile activity, and adhesion that revealed Cdc42 as a complex organizer of melanocyte motility systems [133]. Early evidence of the involvement of cytoskeleton in dendrite formation by melanocytes was produced by Lacour et al. [134]. Moreover, Scott et al. [135] highlighted that the expression of constitutively active Cdc42 protein in melanocytes is responsible for a marked **increase in melanosome-containing dendrite filopodia** and in **filopodia attachment to keratinocyte**. Interestingly, NSMAF also possesses a BEACH domain (named after ‘Beige and Chediak-Higashi’, where *beige* is the name of the **Chediak-Higashi Syndrome** mouse model [136], which is a conserved ~280 residue domain, present in a family of proteins conserved throughout eukaryotes, often described generically as playing a role in **membrane dynamics and/or intracellular trafficking of endosome or lysosome-related proteins and vesicles** [137]. This family includes the lysosomal trafficking regulator (*LYST*), the gene mutated in Chediak-Higashi syndrome (CHS), which is a rare, autosomal recessive disorder that can cause severe immunodeficiency, prolonged bleeding, neurological symptoms and **hypopigmentation** of the eyes and skin [138]. In particular, in the human species, the childhood occurrence of **silvery grey colored hair** has been reported [139,140]. In mice, an additional murine mutant Lyst allele, named grey because of the **grey coat color** of affected mice, was described by Runkel et al. [141]. Besides humans and mice, CHS has been also described in rat [142], mink [143], cats [144], and cattle [145], and, for all the above species, the involvement of the LYST gene is supported. The CHS phenotype has been additionally described in killer whale, fox, tiger, and bison, but no molecular data where generated to confirm the underlying causal mutation. CHS is distinguishable from other hypopigmentation with immunodeficiency syndromes as only CHS is characterized by the presence of enlarged lysosomes in various cell types, as well as the **presence of giant melanosomes in melanocytes that prevent the even distribution of melanin** [146]. Möhlig et al. [147] showed that NSMAF-deficient cells have enlarged lysosomes, like LYST-deficient cells, thus allowing to formulate the hypothesis that **NSMAF may be involved in the occurrence of cattle phenotypes characterized by hair hypopigmentation/greying similarly to those described in CHS**. CHS is characterized by a large phenotypic variability; noteworthy, **hyperpigmentation of extremities and sun-exposed areas** (namely the face, external ears, upper and lower extremities) have been widely documented in Asian CHS patients [148-150].

**REFERENCES**

1. Krishnan, R.; Boddapati, N.; Mahalingam, S., Interplay between human nucleolar GNL1 and RPS20 is critical to modulate cell proliferation. *Sci Rep* **2018,** *8* (1), 11421.

2. Bengsch, F.; Tu, Z.; Tang, H. Y.; Zhu, H.; Speicher, D. W.; Zhang, R., Comprehensive analysis of the ubiquitinome during oncogene-induced senescence in human fibroblasts. *Cell Cycle* **2015,** *14* (10), 1540-7.

3. Hashimoto, S.; Sugiyama, T.; Yamazaki, R.; Nobuta, R.; Inada, T., Identification of a novel trigger complex that facilitates ribosome-associated quality control in mammalian cells. *Sci Rep* **2020,** *10* (1), 3422.

4. Garshott, D. M.; Sundaramoorthy, E.; Leonard, M.; Bennett, E. J., Distinct regulatory ribosomal ubiquitylation events are reversible and hierarchically organized. *Elife* **2020,** *9*.

5. Ikeuchi, K.; Tesina, P.; Matsuo, Y.; Sugiyama, T.; Cheng, J.; Saeki, Y.; Tanaka, K.; Becker, T.; Beckmann, R.; Inada, T., Collided ribosomes form a unique structural interface to induce Hel2-driven quality control pathways. *EMBO J* **2019,** *38* (5).

6. Matsuo, Y.; Ikeuchi, K.; Saeki, Y.; Iwasaki, S.; Schmidt, C.; Udagawa, T.; Sato, F.; Tsuchiya, H.; Becker, T.; Tanaka, K.; Ingolia, N. T.; Beckmann, R.; Inada, T., Ubiquitination of stalled ribosome triggers ribosome-associated quality control. *Nat Commun* **2017,** *8* (1), 159.

7. Daftuar, L.; Zhu, Y.; Jacq, X.; Prives, C., Ribosomal proteins RPL37, RPS15 and RPS20 regulate the Mdm2-p53-MdmX network. *PLoS One* **2013,** *8* (7), e68667.

8. McGowan, K. A.; Li, J. Z.; Park, C. Y.; Beaudry, V.; Tabor, H. K.; Sabnis, A. J.; Zhang, W.; Fuchs, H.; de Angelis, M. H.; Myers, R. M.; Attardi, L. D.; Barsh, G. S., Ribosomal mutations cause p53-mediated dark skin and pleiotropic effects. *Nat Genet* **2008,** *40* (8), 963-70.

9. Grigoletto, L.; Ferraz, J. B. S.; Oliveira, H. R.; Eler, J. P.; Bussiman, F. O.; Abreu Silva, B. C.; Baldi, F.; Brito, L. F., Genetic Architecture of Carcass and Meat Quality Traits in Montana Tropical((R)) Composite Beef Cattle. *Front Genet* **2020,** *11*, 123.

10. Yong, W. H.; Shabihkhani, M.; Telesca, D.; Yang, S.; Tso, J. L.; Menjivar, J. C.; Wei, B.; Lucey, G. M.; Mareninov, S.; Chen, Z.; Liau, L. M.; Lai, A.; Nelson, S. F.; Cloughesy, T. F.; Tso, C. L., Ribosomal Proteins RPS11 and RPS20, Two Stress-Response Markers of Glioblastoma Stem Cells, Are Novel Predictors of Poor Prognosis in Glioblastoma Patients. *PLoS One* **2015,** *10* (10), e0141334.

11. Goldstone, S. D.; Lavin, M. F., Isolation of a cDNA clone, encoding the ribosomal protein S20, downregulated during the onset of apoptosis in a human leukaemic cell line. *Biochem Biophys Res Commun* **1993,** *196* (2), 619-23.

12. Pausch, H.; Flisikowski, K.; Jung, S.; Emmerling, R.; Edel, C.; Gotz, K. U.; Fries, R., Genome-wide association study identifies two major loci affecting calving ease and growth-related traits in cattle. *Genetics* **2011,** *187* (1), 289-97.

13. Mota, L. F. M.; Lopes, F. B.; Fernandes Junior, G. A.; Rosa, G. J. M.; Magalhaes, A. F. B.; Carvalheiro, R.; Albuquerque, L. G., Genome-wide scan highlights the role of candidate genes on phenotypic plasticity for age at first calving in Nellore heifers. *Sci Rep* **2020,** *10* (1), 6481.

14. Utsunomiya, Y. T.; do Carmo, A. S.; Carvalheiro, R.; Neves, H. H.; Matos, M. C.; Zavarez, L. B.; Perez O'Brien, A. M.; Solkner, J.; McEwan, J. C.; Cole, J. B.; Van Tassell, C. P.; Schenkel, F. S.; da Silva, M. V.; Porto Neto, L. R.; Sonstegard, T. S.; Garcia, J. F., Genome-wide association study for birth weight in Nellore cattle points to previously described orthologous genes affecting human and bovine height. *BMC Genet* **2013,** *14*, 52.

15. Taye, M.; Yoon, J.; Dessie, T.; Cho, S.; Oh, S. J.; Lee, H. K.; Kim, H., Deciphering signature of selection affecting beef quality traits in Angus cattle. *Genes Genomics* **2018,** *40* (1), 63-75.

16. Okazaki, K.; Sagata, N., The Mos/MAP kinase pathway stabilizes c-Fos by phosphorylation and augments its transforming activity in NIH 3T3 cells. *EMBO J* **1995,** *14* (20), 5048-59.

17. Herzog, N. K.; Ramagli, L. S.; Khorana, S.; Arlinghaus, R. B., Evidence for somatic cell expression of the c-mos protein [corrected]. *Oncogene* **1989,** *4* (11), 1307-15.

18. Leibovitch, S. A.; Guillier, M.; Lenormand, J. L.; Leibovitch, M. P., p34cdc2 protein is complexed with the c-mos protein in rat skeletal muscle. *Oncogene* **1993,** *8* (9), 2361-9.

19. Evangelou, K.; Balaskas, C.; Marinos, E.; Dosios, T.; Kittas, C.; Gorgoulis, V. G., Immunohistochemical localization of c-mos at the light and electron microscope level in non-small cell lung carcinomas. *Biotech Histochem* **2002,** *77* (2), 85-91.

20. Yew, N.; Strobel, M.; Vande Woude, G. F., Mos and the cell cycle: the molecular basis of the transformed phenotype. *Curr Opin Genet Dev* **1993,** *3* (1), 19-25.

21. Paulino, L.; Barroso, P. A. A.; Silva, A. W. B.; Souza, A. L. P.; Bezerra, F. T. G.; Silva, B. R.; Donato, M. M. A.; Peixoto, C. A.; Silva, J. R. V., Effects of epidermal growth factor and progesterone on development, ultrastructure and gene expression of bovine secondary follicles cultured in vitro. *Theriogenology* **2020,** *142*, 284-290.

22. Jalocha, I.; Gabrys, M. S.; Bal, J., [The crucial role of the proto-oncogene c-mos in regulation of oocyte maturation]. *Postepy Hig Med Dosw (Online)* **2010,** *64*, 636-41.

23. Leibovitch, M. P.; Solhonne, B.; Guillier, M.; Verrelle, P.; Leibovitch, S. A.; Verelle, P., Direct relationship between the expression of tumor suppressor H19 mRNA and c-mos proto-oncogene during myogenesis. *Oncogene* **1995,** *10* (2), 251-60.

24. Leibovitch, S. A.; Guillier, M.; Lenormand, J. L.; Leibovitch, M. P., Accumulation of the c-mos protein is correlated with post-natal development of skeletal muscle. *Oncogene* **1991,** *6* (9), 1617-22.

25. Benayoun, B.; Pelpel, K.; Solhonne, B.; Guillier, M.; Leibovitch, S. A., Overexpression of Mos(rat) proto-oncogene product enhances the positive autoregulatory loop of MyoD. *FEBS Lett* **1998,** *437* (1-2), 39-43.

26. Sun, L.; Guo, C.; Yan, L.; Li, H.; Sun, J.; Huo, X.; Xie, X.; Hu, J., Syntenin regulates melanogenesis via the p38 MAPK pathway. *Mol Med Rep* **2020,** *22* (2), 733-738.

27. Park, J. U.; Yang, S. Y.; Guo, R. H.; Li, H. X.; Kim, Y. H.; Kim, Y. R., Anti-Melanogenic Effect of Dendropanax morbiferus and Its Active Components via Protein Kinase A/Cyclic Adenosine Monophosphate-Responsive Binding Protein- and p38 Mitogen-Activated Protein Kinase-Mediated Microphthalmia-Associated Transcription Factor Downregulation. *Front Pharmacol* **2020,** *11*, 507.

28. Ko, G. A.; Kang, H. R.; Moon, J. Y.; Ediriweera, M. K.; Eum, S.; Bach, T. T.; Cho, S. K., Annona squamosa L. leaves inhibit alpha-melanocyte-stimulating hormone (alpha-MSH) stimulated melanogenesis via p38 signaling pathway in B16F10 melanoma cells. *J Cosmet Dermatol* **2020,** *19* (7), 1785-1792.

29. Hwang, Y. S.; Oh, S. W.; Park, S. H.; Lee, J.; Yoo, J. A.; Kwon, K.; Park, S. J.; Kim, J.; Yu, E.; Cho, J. Y.; Lee, J., Melanogenic Effects of Maclurin Are Mediated through the Activation of cAMP/PKA/CREB and p38 MAPK/CREB Signaling Pathways. *Oxid Med Cell Longev* **2019,** *2019*, 9827519.

30. Fu, T.; Chai, B.; Shi, Y.; Dang, Y.; Ye, X., Fargesin inhibits melanin synthesis in murine malignant and immortalized melanocytes by regulating PKA/CREB and P38/MAPK signaling pathways. *J Dermatol Sci* **2019,** *94* (1), 213-219.

31. Kang, M.; Park, S. H.; Park, S. J.; Oh, S. W.; Yoo, J. A.; Kwon, K.; Kim, J.; Yu, E.; Cho, J. Y.; Lee, J., p44/42 MAPK signaling is a prime target activated by phenylethyl resorcinol in its anti-melanogenic action. *Phytomedicine* **2019,** *58*, 152877.

32. Ku, K. E.; Choi, N.; Oh, S. H.; Kim, W. S.; Suh, W.; Sung, J. H., Src inhibition induces melanogenesis in human G361 cells. *Mol Med Rep* **2019,** *19* (4), 3061-3070.

33. Hu, S.; Huang, J.; Pei, S.; Ouyang, Y.; Ding, Y.; Jiang, L.; Lu, J.; Kang, L.; Huang, L.; Xiang, H.; Xiao, R.; Zeng, Q.; Chen, J., Ganoderma lucidum polysaccharide inhibits UVB-induced melanogenesis by antagonizing cAMP/PKA and ROS/MAPK signaling pathways. *J Cell Physiol* **2019,** *234* (5), 7330-7340.

34. Lee, S. E.; Park, S. H.; Oh, S. W.; Yoo, J. A.; Kwon, K.; Park, S. J.; Kim, J.; Lee, H. S.; Cho, J. Y.; Lee, J., Beauvericin inhibits melanogenesis by regulating cAMP/PKA/CREB and LXR-alpha/p38 MAPK-mediated pathways. *Sci Rep* **2018,** *8* (1), 14958.

35. Liu, L.; Fu, M.; Pei, S.; Zhou, L.; Shang, J., R-Fluoxetine Increases Melanin Synthesis Through a 5-HT1A/2A Receptor and p38 MAPK Signaling Pathways. *Int J Mol Sci* **2018,** *20* (1).

36. Adnani, L.; Dixit, R.; Chen, X.; Balakrishnan, A.; Modi, H.; Touahri, Y.; Logan, C.; Schuurmans, C., Plag1 and Plagl2 have overlapping and distinct functions in telencephalic development. *Biol Open* **2018,** *7* (11).

37. Sakai, H.; Fujii, Y.; Kuwayama, N.; Kawaji, K.; Gotoh, Y.; Kishi, Y., Plag1 regulates neuronal gene expression and neuronal differentiation of neocortical neural progenitor cells. *Genes Cells* **2019,** *24* (10), 650-666.

38. Tang, W.; Tang, J.; He, J.; Zhou, Z.; Qin, Y.; Qin, J.; Li, B.; Xu, X.; Geng, Q.; Jiang, W.; Wu, W.; Wang, X.; Xia, Y., SLIT2/ROBO1-miR-218-1-RET/PLAG1: a new disease pathway involved in Hirschsprung's disease. *J Cell Mol Med* **2015,** *19* (6), 1197-207.

39. Inoue, T.; Nakamura, A.; Iwahashi-Odano, M.; Tanase-Nakao, K.; Matsubara, K.; Nishioka, J.; Maruo, Y.; Hasegawa, Y.; Suzumura, H.; Sato, S.; Kobayashi, Y.; Murakami, N.; Nakabayashi, K.; Yamazawa, K.; Fuke, T.; Narumi, S.; Oka, A.; Ogata, T.; Fukami, M.; Kagami, M., Contribution of gene mutations to Silver-Russell syndrome phenotype: multigene sequencing analysis in 92 etiology-unknown patients. *Clin Epigenetics* **2020,** *12* (1), 86.

40. Partington, M. W., X-linked short stature with skin pigmentation: evidence for heterogeneity of the Russell-Silver syndrome. *Clin Genet* **1986,** *29* (2), 151-6.

41. Abi Habib, W.; Brioude, F.; Edouard, T.; Bennett, J. T.; Lienhardt-Roussie, A.; Tixier, F.; Salem, J.; Yuen, T.; Azzi, S.; Le Bouc, Y.; Harbison, M. D.; Netchine, I., Genetic disruption of the oncogenic HMGA2-PLAG1-IGF2 pathway causes fetal growth restriction. *Genet Med* **2018,** *20* (2), 250-258.

42. Declercq, J.; Van Dyck, F.; Van Damme, B.; Van de Ven, W. J., Upregulation of Igf and Wnt signalling associated genes in pleomorphic adenomas of the salivary glands in PLAG1 transgenic mice. *Int J Oncol* **2008,** *32* (5), 1041-7.

43. Zhao, X.; Ren, W.; Yang, W.; Wang, Y.; Kong, H.; Wang, L.; Yan, L.; Xu, G.; Fei, J.; Fu, J.; Zhang, C.; Wang, Z., Wnt pathway is involved in pleomorphic adenomas induced by overexpression of PLAG1 in transgenic mice. *Int J Cancer* **2006,** *118* (3), 643-8.

44. Goto, Y.; Ibi, M.; Sato, H.; Tanaka, J.; Yasuhara, R.; Aota, K.; Azuma, M.; Fukada, T.; Mishima, K.; Irie, T., PLAG1 enhances the stemness profiles of acinar cells in normal human salivary glands in a cell type-specific manner. *J Oral Biosci* **2020,** *62* (1), 99-106.

45. Wang, Q.; Lin, C.; Zhang, C.; Wang, H.; Lu, Y.; Yao, J.; Wei, Q.; Xing, G.; Cao, X., 25-hydroxycholesterol down-regulates oxysterol binding protein like 2 (OSBPL2) via the p53/SREBF2/NFYA signaling pathway. *J Steroid Biochem Mol Biol* **2019,** *187*, 17-26.

46. Jin, L.; Chun, J.; Pan, C.; Kumar, A.; Zhang, G.; Ha, Y.; Li, D.; Alesi, G. N.; Kang, Y.; Zhou, L.; Yu, W. M.; Magliocca, K. R.; Khuri, F. R.; Qu, C. K.; Metallo, C.; Owonikoko, T. K.; Kang, S., The PLAG1-GDH1 Axis Promotes Anoikis Resistance and Tumor Metastasis through CamKK2-AMPK Signaling in LKB1-Deficient Lung Cancer. *Mol Cell* **2018,** *69* (1), 87-99 e7.

47. Edea, Z.; Jung, K. S.; Shin, S. S.; Yoo, S. W.; Choi, J. W.; Kim, K. S., Signatures of positive selection underlying beef production traits in Korean cattle breeds. *J Anim Sci Technol* **2020,** *62* (3), 293-305.

48. Gautier, M., Genome-Wide Scan for Adaptive Divergence and Association with Population-Specific Covariates. *Genetics* **2015,** *201* (4), 1555-79.

49. Randhawa, I. A.; Khatkar, M. S.; Thomson, P. C.; Raadsma, H. W., Composite Selection Signals for Complex Traits Exemplified Through Bovine Stature Using Multibreed Cohorts of European and African Bos taurus. *G3 (Bethesda)* **2015,** *5* (7), 1391-401.

50. Bertolini, F.; Schiavo, G.; Bovo, S.; Sardina, M. T.; Mastrangelo, S.; Dall'Olio, S.; Portolano, B.; Fontanesi, L., Comparative selection signature analyses identify genomic footprints in Reggiana cattle, the traditional breed of the Parmigiano-Reggiano cheese production system. *Animal* **2020,** *14* (5), 921-932.

51. Cheruiyot, E. K.; Bett, R. C.; Amimo, J. O.; Zhang, Y.; Mrode, R.; Mujibi, F. D. N., Signatures of Selection in Admixed Dairy Cattle in Tanzania. *Front Genet* **2018,** *9*, 607.

52. Boitard, S.; Boussaha, M.; Capitan, A.; Rocha, D.; Servin, B., Uncovering Adaptation from Sequence Data: Lessons from Genome Resequencing of Four Cattle Breeds. *Genetics* **2016,** *203* (1), 433-50.

53. Zhao, F.; McParland, S.; Kearney, F.; Du, L.; Berry, D. P., Detection of selection signatures in dairy and beef cattle using high-density genomic information. *Genet Sel Evol* **2015,** *47*, 49.

54. Purfield, D. C.; Evans, R. D.; Berry, D. P., Breed- and trait-specific associations define the genetic architecture of calving performance traits in cattle. *J Anim Sci* **2020,** *98* (5).

55. Hou, J.; Qu, K.; Jia, P.; Hanif, Q.; Zhang, J.; Chen, N.; Dang, R.; Chen, H.; Huang, B.; Lei, C., A SNP in PLAG1 is associated with body height trait in Chinese cattle. *Anim Genet* **2020,** *51* (1), 87-90.

56. Zhou, Z.; Huang, B.; Lai, Z.; Li, S.; Wu, F.; Qu, K.; Jia, Y.; Hou, J.; Liu, J.; Lei, C.; Dang, R., The Distribution Characteristics of a 19-bp Indel of the PLAG1 Gene in Chinese Cattle. *Animals (Basel)* **2019,** *9* (12).

57. Xu, W.; He, H.; Zheng, L.; Xu, J. W.; Lei, C. Z.; Zhang, G. M.; Dang, R. H.; Niu, H.; Qi, X. L.; Chen, H.; Huang, Y. Z., Detection of 19-bp deletion within PLAG1 gene and its effect on growth traits in cattle. *Gene* **2018,** *675*, 144-149.

58. Zhong, J. L.; Xu, J. W.; Wang, J.; Wen, Y. F.; Niu, H.; Zheng, L.; He, H.; Peng, K.; He, P.; Shi, S. Y.; Huang, Y. Q.; Lei, C. Z.; Dang, R. H.; Lan, X. Y.; Qi, X. L.; Chen, H.; Huang, Y. Z., A novel SNP of PLAG1 gene and its association with growth traits in Chinese cattle. *Gene* **2019,** *689*, 166-171.

59. Li, Z.; Wu, M.; Zhao, H.; Fan, L.; Zhang, Y.; Yuan, T.; He, S.; Wang, P.; Zhang, Y.; Sun, X.; Wang, S., The PLAG1 mRNA expression analysis among genetic variants and relevance to growth traits in Chinese cattle. *Anim Biotechnol* **2019**, 1-8.

60. Smith, J. L.; Wilson, M. L.; Nilson, S. M.; Rowan, T. N.; Oldeschulte, D. L.; Schnabel, R. D.; Decker, J. E.; Seabury, C. M., Genome-wide association and genotype by environment interactions for growth traits in U.S. Gelbvieh cattle. *BMC Genomics* **2019,** *20* (1), 926.

61. Marete, A. G.; Guldbrandtsen, B.; Lund, M. S.; Fritz, S.; Sahana, G.; Boichard, D., A Meta-Analysis Including Pre-selected Sequence Variants Associated With Seven Traits in Three French Dairy Cattle Populations. *Front Genet* **2018,** *9*, 522.

62. Karim, L.; Takeda, H.; Lin, L.; Druet, T.; Arias, J. A.; Baurain, D.; Cambisano, N.; Davis, S. R.; Farnir, F.; Grisart, B.; Harris, B. L.; Keehan, M. D.; Littlejohn, M. D.; Spelman, R. J.; Georges, M.; Coppieters, W., Variants modulating the expression of a chromosome domain encompassing PLAG1 influence bovine stature. *Nat Genet* **2011,** *43* (5), 405-13.

63. An, B.; Xia, J.; Chang, T.; Wang, X.; Xu, L.; Zhang, L.; Gao, X.; Chen, Y.; Li, J.; Gao, H., Genome-wide association study reveals candidate genes associated with body measurement traits in Chinese Wagyu beef cattle. *Anim Genet* **2019,** *50* (4), 386-390.

64. Zhang, R.; Miao, J.; Song, Y.; Zhang, W.; Xu, L.; Chen, Y.; Zhang, L.; Gao, H.; Zhu, B.; Li, J.; Gao, X., Genome-wide association study identifies the PLAG1-OXR1 region on BTA14 for carcass meat yield in cattle. *Physiol Genomics* **2019,** *51* (5), 137-144.

65. Song, Y.; Xu, L.; Chen, Y.; Zhang, L.; Gao, H.; Zhu, B.; Niu, H.; Zhang, W.; Xia, J.; Gao, X.; Li, J., Genome-Wide Association Study Reveals the PLAG1 Gene for Knuckle, Biceps and Shank Weight in Simmental Beef Cattle. *PLoS One* **2016,** *11* (12), e0168316.

66. Kim, H. J.; Sharma, A.; Lee, S. H.; Lee, D. H.; Lim, D. J.; Cho, Y. M.; Yang, B. S.; Lee, S. H., Genetic association of PLAG1, SCD, CYP7B1 and FASN SNPs and their effects on carcass weight, intramuscular fat and fatty acid composition in Hanwoo steers (Korean cattle). *Anim Genet* **2017,** *48* (2), 251-252.

67. A, G. T. P.; Utsunomiya, Y. T.; Milanesi, M.; Torrecilha, R. B.; Carmo, A. S.; Neves, H. H.; Carvalheiro, R.; Ajmone-Marsan, P.; Sonstegard, T. S.; Solkner, J.; Contreras-Castillo, C. J.; Garcia, J. F., Pleiotropic Genes Affecting Carcass Traits in Bos indicus (Nellore) Cattle Are Modulators of Growth. *PLoS One* **2016,** *11* (7), e0158165.

68. Hartati, H.; Utsunomiya, Y. T.; Sonstegard, T. S.; Garcia, J. F.; Jakaria, J.; Muladno, M., Evidence of Bos javanicus x Bos indicus hybridization and major QTLs for birth weight in Indonesian Peranakan Ongole cattle. *BMC Genet* **2015,** *16*, 75.

69. Saatchi, M.; Schnabel, R. D.; Taylor, J. F.; Garrick, D. J., Large-effect pleiotropic or closely linked QTL segregate within and across ten US cattle breeds. *BMC Genomics* **2014,** *15*, 442.

70. Utsunomiya, Y. T.; Carmo, A. S.; Neves, H. H.; Carvalheiro, R.; Matos, M. C.; Zavarez, L. B.; Ito, P. K.; Perez O'Brien, A. M.; Solkner, J.; Porto-Neto, L. R.; Schenkel, F. S.; McEwan, J.; Cole, J. B.; da Silva, M. V.; Van Tassell, C. P.; Sonstegard, T. S.; Garcia, J. F., Genome-wide mapping of loci explaining variance in scrotal circumference in Nellore cattle. *PLoS One* **2014,** *9* (2), e88561.

71. Fortes, M. R.; Kemper, K.; Sasazaki, S.; Reverter, A.; Pryce, J. E.; Barendse, W.; Bunch, R.; McCulloch, R.; Harrison, B.; Bolormaa, S.; Zhang, Y. D.; Hawken, R. J.; Goddard, M. E.; Lehnert, S. A., Evidence for pleiotropism and recent selection in the PLAG1 region in Australian Beef cattle. *Anim Genet* **2013,** *44* (6), 636-47.

72. Nishimura, S.; Watanabe, T.; Mizoshita, K.; Tatsuda, K.; Fujita, T.; Watanabe, N.; Sugimoto, Y.; Takasuga, A., Genome-wide association study identified three major QTL for carcass weight including the PLAG1-CHCHD7 QTN for stature in Japanese Black cattle. *BMC Genet* **2012,** *13*, 40.

73. Pryce, J. E.; Arias, J.; Bowman, P. J.; Davis, S. R.; Macdonald, K. A.; Waghorn, G. C.; Wales, W. J.; Williams, Y. J.; Spelman, R. J.; Hayes, B. J., Accuracy of genomic predictions of residual feed intake and 250-day body weight in growing heifers using 625,000 single nucleotide polymorphism markers. *J Dairy Sci* **2012,** *95* (4), 2108-19.

74. Fink, T.; Tiplady, K.; Lopdell, T.; Johnson, T.; Snell, R. G.; Spelman, R. J.; Davis, S. R.; Littlejohn, M. D., Functional confirmation of PLAG1 as the candidate causative gene underlying major pleiotropic effects on body weight and milk characteristics. *Sci Rep* **2017,** *7*, 44793.

75. Utsunomiya, Y. T.; Milanesi, M.; Utsunomiya, A. T. H.; Torrecilha, R. B. P.; Kim, E. S.; Costa, M. S.; Aguiar, T. S.; Schroeder, S.; do Carmo, A. S.; Carvalheiro, R.; Neves, H. H. R.; Padula, R. C. M.; Sussai, T. S.; Zavarez, L. B.; Cipriano, R. S.; Caminhas, M. M. T.; Hambrecht, G.; Colli, L.; Eufemi, E.; Ajmone-Marsan, P.; Cesana, D.; Sannazaro, M.; Buora, M.; Morgante, M.; Liu, G.; Bickhart, D.; Van Tassell, C. P.; Solkner, J.; Sonstegard, T. S.; Garcia, J. F., A PLAG1 mutation contributed to stature recovery in modern cattle. *Sci Rep* **2017,** *7* (1), 17140.

76. Koufariotis, L.; Hayes, B. J.; Kelly, M.; Burns, B. M.; Lyons, R.; Stothard, P.; Chamberlain, A. J.; Moore, S., Sequencing the mosaic genome of Brahman cattle identifies historic and recent introgression including polled. *Sci Rep* **2018,** *8* (1), 17761.

77. Signer-Hasler, H.; Burren, A.; Ammann, P.; Drogemuller, C.; Flury, C., Runs of homozygosity and signatures of selection: a comparison among eight local Swiss sheep breeds. *Anim Genet* **2019,** *50* (5), 512-525.

78. Hauge, H.; Patzke, S.; Aasheim, H. C., Characterization of the FAM110 gene family. *Genomics* **2007,** *90* (1), 14-27.

79. Martinez-Morales, J. R.; Henrich, T.; Ramialison, M.; Wittbrodt, J., New genes in the evolution of the neural crest differentiation program. *Genome Biol* **2007,** *8* (3), R36.

80. Hay, E. D., The mesenchymal cell, its role in the embryo, and the remarkable signaling mechanisms that create it. *Dev Dyn* **2005,** *233* (3), 706-20.

81. Chou, S. C.; Ko, T. L.; Fu, Y. Y.; Wang, H. W.; Fu, Y. S., Identification of genetic networks during mesenchymal stem cell transformation into neurons. *Chin J Physiol* **2008,** *51* (4), 230-46.

82. Xi, T.; Zhang, G., Integrated analysis of tumor differentiation genes in pancreatic adenocarcinoma. *PLoS One* **2018,** *13* (3), e0193427.

83. Dutertre, M.; Gratadou, L.; Dardenne, E.; Germann, S.; Samaan, S.; Lidereau, R.; Driouch, K.; de la Grange, P.; Auboeuf, D., Estrogen regulation and physiopathologic significance of alternative promoters in breast cancer. *Cancer Res* **2010,** *70* (9), 3760-70.

84. Vainio, P.; Wolf, M.; Edgren, H.; He, T.; Kohonen, P.; Mpindi, J. P.; Smit, F.; Verhaegh, G.; Schalken, J.; Perala, M.; Iljin, K.; Kallioniemi, O., Integrative genomic, transcriptomic, and RNAi analysis indicates a potential oncogenic role for FAM110B in castration-resistant prostate cancer. *Prostate* **2012,** *72* (7), 789-802.

85. Lee, S. H.; Choi, B. H.; Lim, D.; Gondro, C.; Cho, Y. M.; Dang, C. G.; Sharma, A.; Jang, G. W.; Lee, K. T.; Yoon, D.; Lee, H. K.; Yeon, S. H.; Yang, B. S.; Kang, H. S.; Hong, S. K., Genome-wide association study identifies major loci for carcass weight on BTA14 in Hanwoo (Korean cattle). *PLoS One* **2013,** *8* (10), e74677.

86. Ye, Y.; Tang, W. K.; Zhang, T.; Xia, D., A Mighty "Protein Extractor" of the Cell: Structure and Function of the p97/CDC48 ATPase. *Front Mol Biosci* **2017,** *4*, 39.

87. Ramanathan, H. N.; Ye, Y., The p97 ATPase associates with EEA1 to regulate the size of early endosomes. *Cell Res* **2012,** *22* (2), 346-59.

88. Raposo, G.; Marks, M. S., Melanosomes--dark organelles enlighten endosomal membrane transport. *Nat Rev Mol Cell Biol* **2007,** *8* (10), 786-97.

89. Uchiyama, K.; Totsukawa, G.; Puhka, M.; Kaneko, Y.; Jokitalo, E.; Dreveny, I.; Beuron, F.; Zhang, X.; Freemont, P.; Kondo, H., p37 is a p97 adaptor required for Golgi and ER biogenesis in interphase and at the end of mitosis. *Dev Cell* **2006,** *11* (6), 803-16.

90. Kress, E.; Schwager, F.; Holtackers, R.; Seiler, J.; Prodon, F.; Zanin, E.; Eiteneuer, A.; Toya, M.; Sugimoto, A.; Meyer, H.; Meraldi, P.; Gotta, M., The UBXN-2/p37/p47 adaptors of CDC-48/p97 regulate mitosis by limiting the centrosomal recruitment of Aurora A. *J Cell Biol* **2013,** *201* (4), 559-75.

91. Lee, B. H.; Schwager, F.; Meraldi, P.; Gotta, M., p37/UBXN2B regulates spindle orientation by limiting cortical NuMA recruitment via PP1/Repo-Man. *J Cell Biol* **2018,** *217* (2), 483-493.

92. Noshiro, M.; Nishimoto, M.; Okuda, K., Rat liver cholesterol 7 alpha-hydroxylase. Pretranslational regulation for circadian rhythm. *J Biol Chem* **1990,** *265* (17), 10036-41.

93. Russell, D. W.; Setchell, K. D., Bile acid biosynthesis. *Biochemistry* **1992,** *31* (20), 4737-49.

94. Janikowska, G.; Pyka-Pajak, A.; Janikowski, T.; Adamska, J.; Mazurek, U.; Jedrusik, P., Potential Mechanism of Action of Cyclosporin A in Human Dermal Fibroblasts-Transcriptomic Analysis of CYPs. *Molecules* **2018,** *23* (7).

95. Slominski, A. T.; Zmijewski, M. A.; Semak, I.; Zbytek, B.; Pisarchik, A.; Li, W.; Zjawiony, J.; Tuckey, R. C., Cytochromes p450 and skin cancer: role of local endocrine pathways. *Anticancer Agents Med Chem* **2014,** *14* (1), 77-96.

96. Hanley, K.; Ng, D. C.; He, S. S.; Lau, P.; Min, K.; Elias, P. M.; Bikle, D. D.; Mangelsdorf, D. J.; Williams, M. L.; Feingold, K. R., Oxysterols induce differentiation in human keratinocytes and increase Ap-1-dependent involucrin transcription. *J Invest Dermatol* **2000,** *114* (3), 545-53.

97. Schmuth, M.; Jiang, Y. J.; Dubrac, S.; Elias, P. M.; Feingold, K. R., Thematic review series: skin lipids. Peroxisome proliferator-activated receptors and liver X receptors in epidermal biology. *J Lipid Res* **2008,** *49* (3), 499-509.

98. Russell, L. E.; Harrison, W. J.; Bahta, A. W.; Zouboulis, C. C.; Burrin, J. M.; Philpott, M. P., Characterization of liver X receptor expression and function in human skin and the pilosebaceous unit. *Exp Dermatol* **2007,** *16* (10), 844-52.

99. Billoni, N.; Buan, B.; Gautier, B.; Collin, C.; Gaillard, O.; Mahe, Y. F.; Bernard, B. A., Expression of peroxisome proliferator activated receptors (PPARs) in human hair follicles and PPAR alpha involvement in hair growth. *Acta Derm Venereol* **2000,** *80* (5), 329-34.

100. Kumar, R.; Parsad, D.; Kaul, D.; Kanwar, A. J., Liver X receptor expression in human melanocytes, does it have a role in the pathogenesis of vitiligo? *Exp Dermatol* **2010,** *19* (1), 62-4.

101. Lee, C. S.; Park, M.; Han, J.; Lee, J. H.; Bae, I. H.; Choi, H.; Son, E. D.; Park, Y. H.; Lim, K. M., Liver X receptor activation inhibits melanogenesis through the acceleration of ERK-mediated MITF degradation. *J Invest Dermatol* **2013,** *133* (4), 1063-71.

102. Komuves, L. G.; Schmuth, M.; Fowler, A. J.; Elias, P. M.; Hanley, K.; Man, M. Q.; Moser, A. H.; Lobaccaro, J. M.; Williams, M. L.; Mangelsdorf, D. J.; Feingold, K. R., Oxysterol stimulation of epidermal differentiation is mediated by liver X receptor-beta in murine epidermis. *J Invest Dermatol* **2002,** *118* (1), 25-34.

103. Schmuth, M.; Elias, P. M.; Hanley, K.; Lau, P.; Moser, A.; Willson, T. M.; Bikle, D. D.; Feingold, K. R., The effect of LXR activators on AP-1 proteins in keratinocytes. *J Invest Dermatol* **2004,** *123* (1), 41-8.

104. Willy, P. J.; Mangelsdorf, D. J., Unique requirements for retinoid-dependent transcriptional activation by the orphan receptor LXR. *Genes Dev* **1997,** *11* (3), 289-98.

105. Lu, T. T.; Repa, J. J.; Mangelsdorf, D. J., Orphan nuclear receptors as eLiXiRs and FiXeRs of sterol metabolism. *J Biol Chem* **2001,** *276* (41), 37735-8.

106. Cummins, C. L.; Volle, D. H.; Zhang, Y.; McDonald, J. G.; Sion, B.; Lefrancois-Martinez, A. M.; Caira, F.; Veyssiere, G.; Mangelsdorf, D. J.; Lobaccaro, J. M., Liver X receptors regulate adrenal cholesterol balance. *J Clin Invest* **2006,** *116* (7), 1902-12.

107. Gericke, J.; Ittensohn, J.; Mihaly, J.; Alvarez, S.; Alvarez, R.; Torocsik, D.; de Lera, A. R.; Ruhl, R., Regulation of retinoid-mediated signaling involved in skin homeostasis by RAR and RXR agonists/antagonists in mouse skin. *PLoS One* **2013,** *8* (4), e62643.

108. Slominski, A. T.; Manna, P. R.; Tuckey, R. C., On the role of skin in the regulation of local and systemic steroidogenic activities. *Steroids* **2015,** *103*, 72-88.

109. Schallreuter, K. U.; Hasse, S.; Rokos, H.; Chavan, B.; Shalbaf, M.; Spencer, J. D.; Wood, J. M., Cholesterol regulates melanogenesis in human epidermal melanocytes and melanoma cells. *Exp Dermatol* **2009,** *18* (8), 680-8.

110. Cario, M., How hormones may modulate human skin pigmentation in melasma: An in vitro perspective. *Exp Dermatol* **2019,** *28* (6), 709-718.

111. Natale, C. A.; Duperret, E. K.; Zhang, J.; Sadeghi, R.; Dahal, A.; O'Brien, K. T.; Cookson, R.; Winkler, J. D.; Ridky, T. W., Sex steroids regulate skin pigmentation through nonclassical membrane-bound receptors. *Elife* **2016,** *5*.

112. Scott, M. C.; Suzuki, I.; Abdel-Malek, Z. A., Regulation of the human melanocortin 1 receptor expression in epidermal melanocytes by paracrine and endocrine factors and by ultraviolet radiation. *Pigment Cell Res* **2002,** *15* (6), 433-9.

113. Tadokoro, T.; Rouzaud, F.; Itami, S.; Hearing, V. J.; Yoshikawa, K., The inhibitory effect of androgen and sex-hormone-binding globulin on the intracellular cAMP level and tyrosinase activity of normal human melanocytes. *Pigment Cell Res* **2003,** *16* (3), 190-7.

114. Slominski, A.; Wortsman, J.; Paus, R.; Elias, P. M.; Tobin, D. J.; Feingold, K. R., Skin as an endocrine organ: implications for its function. *Drug Discov Today Dis Mech* **2008,** *5* (2), 137-144.

115. Pang, S.; Wu, H.; Wang, Q.; Cai, M.; Shi, W.; Shang, J., Chronic stress suppresses the expression of cutaneous hypothalamic-pituitary-adrenocortical axis elements and melanogenesis. *PLoS One* **2014,** *9* (5), e98283.

116. Helmke, B. M.; Polychronidis, M.; Benner, A.; Thome, M.; Arribas, J.; Deichmann, M., Melanoma metastasis is associated with enhanced expression of the syntenin gene. *Oncol Rep* **2004,** *12* (2), 221-8.

117. Das, S. K.; Guo, C.; Pradhan, A. K.; Bhoopathi, P.; Talukdar, S.; Shen, X. N.; Emdad, L.; Subler, M. A.; Windle, J. J.; Sarkar, D.; Wang, X. Y.; Fisher, P. B., Knockout of MDA-9/Syntenin (SDCBP) expression in the microenvironment dampens tumor-supporting inflammation and inhibits melanoma metastasis. *Oncotarget* **2016,** *7* (30), 46848-46861.

118. Tumbar, T.; Guasch, G.; Greco, V.; Blanpain, C.; Lowry, W. E.; Rendl, M.; Fuchs, E., Defining the epithelial stem cell niche in skin. *Science* **2004,** *303* (5656), 359-63.

119. Colombo, S.; Champeval, D.; Rambow, F.; Larue, L., Transcriptomic analysis of mouse embryonic skin cells reveals previously unreported genes expressed in melanoblasts. *J Invest Dermatol* **2012,** *132* (1), 170-8.

120. Jeon, H. Y.; Das, S. K.; Dasgupta, S.; Emdad, L.; Sarkar, D.; Kim, S. H.; Lee, S. G.; Fisher, P. B., Expression patterns of MDA-9/syntenin during development of the mouse embryo. *J Mol Histol* **2013,** *44* (2), 159-66.

121. Sahmatova, L.; Tankov, S.; Prans, E.; Aab, A.; Hermann, H.; Reemann, P.; Pihlap, M.; Karelson, M.; Abram, K.; Kisand, K.; Kingo, K.; Rebane, A., MicroRNA-155 is Dysregulated in the Skin of Patients with Vitiligo and Inhibits Melanogenesis-associated Genes in Melanocytes and Keratinocytes. *Acta Derm Venereol* **2016,** *96* (6), 742-7.

122. Bian, Y.; Wei, G.; Song, X.; Yuan, L.; Chen, H.; Ni, T.; Lu, D., Global downregulation of pigmentation-associated genes in human premature hair graying. *Exp Ther Med* **2019,** *18* (2), 1155-1163.

123. Gao, Y.; Li, M.; Chen, W.; Simons, M., Synectin, syndecan-4 cytoplasmic domain binding PDZ protein, inhibits cell migration. *J Cell Physiol* **2000,** *184* (3), 373-9.

124. Zimmermann, P.; Tomatis, D.; Rosas, M.; Grootjans, J.; Leenaerts, I.; Degeest, G.; Reekmans, G.; Coomans, C.; David, G., Characterization of syntenin, a syndecan-binding PDZ protein, as a component of cell adhesion sites and microfilaments. *Mol Biol Cell* **2001,** *12* (2), 339-50.

125. Bernfield, M.; Gotte, M.; Park, P. W.; Reizes, O.; Fitzgerald, M. L.; Lincecum, J.; Zako, M., Functions of cell surface heparan sulfate proteoglycans. *Annu Rev Biochem* **1999,** *68*, 729-77.

126. Maatta, A.; Jaakkola, P.; Jalkanen, M., Extracellular matrix-dependent activation of syndecan-1 expression in keratinocyte growth factor-treated keratinocytes. *J Biol Chem* **1999,** *274* (14), 9891-8.

127. Chung, H.; Lee, J. H.; Jeong, D.; Han, I. O.; Oh, E. S., Melanocortin 1 receptor regulates melanoma cell migration by controlling syndecan-2 expression. *J Biol Chem* **2012,** *287* (23), 19326-35.

128. Jung, H.; Oh, E. S., FK506 positively regulates the migratory potential of melanocyte-derived cells by enhancing syndecan-2 expression. *Pigment Cell Melanoma Res* **2016,** *29* (4), 434-43.

129. Jung, H.; Chung, H.; Chang, S. E.; Choi, S.; Han, I. O.; Kang, D. H.; Oh, E. S., Syndecan-2 regulates melanin synthesis via protein kinase C betaII-mediated tyrosinase activation. *Pigment Cell Melanoma Res* **2014,** *27* (3), 387-97.

130. Kim, D. S.; Kim, S. Y.; Moon, S. J.; Chung, J. H.; Kim, K. H.; Cho, K. H.; Park, K. C., Ceramide inhibits cell proliferation through Akt/PKB inactivation and decreases melanin synthesis in Mel-Ab cells. *Pigment Cell Res* **2001,** *14* (2), 110-5.

131. Laurent-Gengoux, P.; Petit, V.; Aktary, Z.; Gallagher, S.; Tweedy, L.; Machesky, L.; Larue, L., Simulation of melanoblast displacements reveals new features of developmental migration. *Development* **2018,** *145* (12).

132. Haubert, D.; Gharib, N.; Rivero, F.; Wiegmann, K.; Hosel, M.; Kronke, M.; Kashkar, H., PtdIns(4,5)P-restricted plasma membrane localization of FAN is involved in TNF-induced actin reorganization. *EMBO J* **2007,** *26* (14), 3308-21.

133. Woodham, E. F.; Paul, N. R.; Tyrrell, B.; Spence, H. J.; Swaminathan, K.; Scribner, M. R.; Giampazolias, E.; Hedley, A.; Clark, W.; Kage, F.; Marston, D. J.; Hahn, K. M.; Tait, S. W.; Larue, L.; Brakebusch, C. H.; Insall, R. H.; Machesky, L. M., Coordination by Cdc42 of Actin, Contractility, and Adhesion for Melanoblast Movement in Mouse Skin. *Curr Biol* **2017,** *27* (5), 624-637.

134. Lacour, J. P.; Gordon, P. R.; Eller, M.; Bhawan, J.; Gilchrest, B. A., Cytoskeletal events underlying dendrite formation by cultured pigment cells. *J Cell Physiol* **1992,** *151* (2), 287-99.

135. Scott, G.; Leopardi, S.; Printup, S.; Madden, B. C., Filopodia are conduits for melanosome transfer to keratinocytes. *J Cell Sci* **2002,** *115* (Pt 7), 1441-51.

136. Perou, C. M.; Moore, K. J.; Nagle, D. L.; Misumi, D. J.; Woolf, E. A.; McGrail, S. H.; Holmgren, L.; Brody, T. H.; Dussault, B. J., Jr.; Monroe, C. A.; Duyk, G. M.; Pryor, R. J.; Li, L.; Justice, M. J.; Kaplan, J., Identification of the murine beige gene by YAC complementation and positional cloning. *Nat Genet* **1996,** *13* (3), 303-8.

137. Cullinane, A. R.; Schaffer, A. A.; Huizing, M., The BEACH is hot: a LYST of emerging roles for BEACH-domain containing proteins in human disease. *Traffic* **2013,** *14* (7), 749-66.

138. Ward, D. M.; Shiflett, S. L.; Kaplan, J., Chediak-Higashi syndrome: a clinical and molecular view of a rare lysosomal storage disorder. *Curr Mol Med* **2002,** *2* (5), 469-77.

139. Jin, Y.; Zhang, L.; Wang, S.; Chen, F.; Gu, Y.; Hong, E.; Yu, Y.; Ni, X.; Guo, Y.; Shi, T.; Xu, Z., Whole Genome Sequencing Identifies Novel Compound Heterozygous Lysosomal Trafficking Regulator Gene Mutations Associated with Autosomal Recessive Chediak-Higashi Syndrome. *Sci Rep* **2017,** *7*, 41308.

140. Patne, S. C.; Kumar, S.; Bagri, N. K.; Kumar, A.; Shukla, J., Chediak-higashi syndrome: a case report. *Indian J Hematol Blood Transfus* **2013,** *29* (2), 80-3.

141. Runkel, F.; Bussow, H.; Seburn, K. L.; Cox, G. A.; Ward, D. M.; Kaplan, J.; Franz, T., Grey, a novel mutation in the murine Lyst gene, causes the beige phenotype by skipping of exon 25. *Mamm Genome* **2006,** *17* (3), 203-10.

142. Mori, M.; Yamasaki, K.; Nakanishi, S.; Kitada, K.; Higuchi, K.; Namiki, C.; Hamada, S.; Serikawa, T., A new beige mutant rat ACI/N-Lystbg-Kyo. *Exp Anim* **2003,** *52* (1), 31-6.

143. Anistoroaei, R.; Krogh, A. K.; Christensen, K., A frameshift mutation in the LYST gene is responsible for the Aleutian color and the associated Chediak-Higashi syndrome in American mink. *Anim Genet* **2013,** *44* (2), 178-83.

144. Buckley, R. M.; Grahn, R. A.; Gandolfi, B.; Herrick, J. R.; Kittleson, M. D.; Bateman, H. L.; Newsom, J.; Swanson, W. F.; Prieur, D. J.; Lyons, L. A., Assisted reproduction mediated resurrection of a feline model for Chediak-Higashi syndrome caused by a large duplication in LYST. *Sci Rep* **2020,** *10* (1), 64.

145. Kunieda, T.; Nakagiri, M.; Takami, M.; Ide, H.; Ogawa, H., Cloning of bovine LYST gene and identification of a missense mutation associated with Chediak-Higashi syndrome of cattle. *Mamm Genome* **1999,** *10* (12), 1146-9.

146. Westbroek, W.; Adams, D.; Huizing, M.; Koshoffer, A.; Dorward, H.; Tinloy, B.; Parkes, J.; Helip-Wooley, A.; Kleta, R.; Tsilou, E.; Duvernay, P.; Digre, K. B.; Creel, D. J.; White, J. G.; Boissy, R. E.; Gahl, W. A., Cellular defects in Chediak-Higashi syndrome correlate with the molecular genotype and clinical phenotype. *J Invest Dermatol* **2007,** *127* (11), 2674-7.

147. Mohlig, H.; Mathieu, S.; Thon, L.; Frederiksen, M. C.; Ward, D. M.; Kaplan, J.; Schutze, S.; Kabelitz, D.; Adam, D., The WD repeat protein FAN regulates lysosome size independent from abnormal downregulation/membrane recruitment of protein kinase C. *Exp Cell Res* **2007,** *313* (12), 2703-18.

148. Ho, M. C.; Hsieh, Y. T., Mixed hyperpigmentation and hypopigmentation of iris and choroid in Chediak-Higashi syndrome. *J AAPOS* **2013,** *17* (5), 558-60.

149. Fukuchi, K.; Tatsuno, K.; Sakaguchi, K.; Sano, S.; Sasaki, T.; Aoki, S.; Kubo, A.; Tokura, Y., Novel gene mutations in Chediak-Higashi syndrome with hyperpigmentation. *J Dermatol* **2019,** *46* (11), e416-e418.

150. Usha, H. N.; Prabhu, P. D.; Sridevi, M.; Baindur, K.; Balakrishnan, C. M., Chediak-Higashi syndrome. *Indian Pediatr* **1994,** *31* (9), 1115-9.
