# Supplementary material for: Fifteen Shades of Grey: Combined Analysis of Genome-Wide SNP Data in Steppe and Mediterranean Grey Cattle Sheds New Light on the Molecular Basis of Coat Color
Source: Genes (Basel). 2020 Aug 13;11(8):932. doi: 10.3390/genes11080932 (PMC7464420; doi:10.3390/genes11080932)
Supplement: Supplementary file 1 [file genes-11-00932-s001.zip › Supplementary file S8.docx]

**Supplementary file S8.** Non-exhaustive list of the online material screened in order to collect information on coat color phenotypes for the considered breeds.

| **Asiatic zebuine cattle breeds** |
| --- |
| - https://alchetron.com/Tharparkar-(cattle) - http://dadf.gov.in/sites/default/filess/Central%20Breeding%20Farm%20Suratgarh_0_0.pdf - https://books.google.it/books?id=Y-VfAwAAQBAJ&pg=PA422&lpg=PA422&dq=Tharparkar+calf+color&source=bl&ots=tAW4XbMC1c&sig=ACfU3U00eIa5JCNi6TPauHLlzFjQEMXq8w&hl=it&sa=X&ved=2ahUKEwi34PC-sKzqAhUlQxUIHaKsDgoQ6AEwE3oECAoQAQ#v=onepage&q=Tharparkar%20&f=false - https://www.dairyknowledge.in/section/cattle-breeds |
| **Latin American imported breeds of zebuine origin** |
| - https://it.123rf.com/archivio-fotografico/nelore.html?sti=n5jqxkcqvjas85vh4s\| - http://afs.okstate.edu/breeds/cattle/nelore/index.html/ |
| **Asiatic grey Steppe cattle breeds** |
| - https://www.alamy.com/hungarian-grey-cattle-with-calf-image281058209.html - https://www.learnnaturalfarming.com/complete-details-on-ukrainian-grey-cattle/ |
| **European breeds of the Podolian group** |
| - https://www.arabasilicata.it/page/?s=59 - https://www.passaturi.it/it/blog/29-piante-e-animali/297-i-bovini-podolici-in-puglia.html - https://agronotizie.imagelinenetwork.com/zootecnia/2017/04/04/linea-vacca-vitello-si-puo-fare-di-piu-e-meglio/53563 - https://agronotizie.imagelinenetwork.com/zootecnia/2020/04/22/chianina-asta-online-in-tempo-di-quarantena/66579 - https://www.likesx.com/vacca-vitelle-razza-marchigiana/ - http://www.aziendaagricolasargenti.it/service/allevamento-carni-bovine/ - https://appuntidizootecnia.blogspot.com/p/bovine-italiane-da-carne.html - https://www.braciamiancora.com/la-carne-italiana-le-dieci-migliori-razze/ - https://agronotizie.imagelinenetwork.com/zootecnia/2019/02/04/il-debutto-in-fiera-della-romagnola/61651 - https://www.dreamstime.com/royalty-free-stock-photography-maremma-cow-calf-breeding-meremmana-as-breastfeeds-her-image31409557 - https://www.agefotostock.com/age/en/Stock-Images/Rights-Managed/IBR-2095796 - https://turismoinmaremma.wordpress.com/2014/03/17/la-vacca-maremmana/ - https://informatorezootecnico.edagricole.it/aia-informa/maremmana-leffetto-di-35-anni-di-selezione/ |
| **Other local breeds from Mediterranean countries** |
| - https://www.alamy.it/fotos-immagini/gasconne.html - https://www.ariege.com/en/discover-ariege/agro-pastoralism/gascony-cow |
| **Alpine cattle breeds** |
| - https://www.taccuinigastrosofici.it/ita/news/contemporanea/cibi-tradizionali/Razza-bovina-grigio-alpina.html - https://www.flickr.com/photos/146684860@N07/25054226117 - http://www.arche-austria.at/index.php?id=96 |
